# Ursolic Acid Regulates Intestinal Microbiota and Inflammatory Cell Infiltration to Prevent Ulcerative Colitis

**DOI:** 10.1155/2021/6679316

**Published:** 2021-04-30

**Authors:** Qinsong Sheng, Fei Li, Guanping Chen, Jiacheng Li, Jing Li, YiFan Wang, Yingyan Lu, Qun Li, Mingqian Li, Kequn Chai

**Affiliations:** ^1^Department of Colorectal and Anal Surgery, The First Affiliated Hospital of College of Medicine, Zhejiang University, China; ^2^College of Life Science, Sichuan Normal University, Chengdu, Sichuan 610101, China; ^3^Cancer Institute of Integrated Tradition Chinese and Western Medicine, Zhejiang Academy of Traditional Chinese Medicine, Tongde Hospital of Zhejiang Province, Hangzhou, Zhejiang 310012, China

## Abstract

Ulcerative colitis (UC) is a chronic and relapsing inflammatory bowel disorder in the colon and rectum leading to low life-quality and high societal costs. Ursolic acid (UA) is a natural product with pharmacological and biological activities. The studies are aimed at investigating the protective and treatment effects of UA against the dextran sulfate sodium- (DSS-) induced UC mouse model and its underlying mechanism. UA was orally administered at different time points before and after the DSS-induced model. Mice body weight, colon length, and histological analysis were used to evaluate colon tissue damage and therapeutic evaluation. Intestinal transcriptome and microbe 16 s sequencing was used to analyze the mechanisms of UA in the prevention and treatment of UC. The early prevention effect of UA could effectively delay mouse weight loss and colon length shorten. UA alleviated UC inflammation and lowered serum and colon IL-6 levels. Three classical inflammatory pathways: MAPKs, IL-6/STAT3, and PI3K were downregulated by UA treatment. The proportion of macrophages and neutrophils in inflammatory cell infiltration was reduced in UA treatment groups. UA could significantly reduce the richness of intestinal flora to avoid the inflammatory response due to the destruction of the intestinal epithelial barrier. The function of UA against UC was through reducing intestinal flora abundance and regulating inflammatory and fatty acid metabolism signaling pathways to affect immune cell infiltration and cytokine expression.

## 1. Introduction

Ulcerative colitis (UC) is a chronic and relapsing inflammatory bowel disorder in the colon and rectum, which can induce recurrent episodes of bloody diarrhea, abdominal pain, and even colorectal cancer [1]. The unhealthy lifestyle, gut microbiota, and genetic factors may be the main cause of the pathogenesis of UC [2]. At present, many drugs are used to treat UC including aminosalicylates, corticosteroids, immunosuppressants, and biological reagents; however, a large of patients are still ineffective or have more side effects [3]. Therefore, new therapeutic strategies for UC need to be developed.

Ursolic acid (UA), purified from medicinal plants and foods such as lavender and apple peel, is a natural pentacyclic triterpenoid carboxylic acid with pharmacological and biological activities [4]. The UA may possess broad-spectrum anticancer effects by promoting the apoptosis and autophagy of cancer cells to inhibit cell growth [5–9]. The anti-inflammation mechanisms of UA are reported to inhibit the production of proinflammatory cytokines such as IL6, IL1*β*, and TNF and reduced the high nuclear level of NF*κ*B p65 [10–13]. UA can reduce transaminase (AST and ALT) levels and fat accumulation to protect against liver diseases [14–16]. Moreover, UA showed antibacterial activity to reduce bacterial biofilm mass of Streptococcus mutants, Pseudomonas aeruginosa, Actinomyces viscosus, etc. and has a synergistic effect against both Staphylococcus aureus and Bacillus cereus with ampicillin and tetracycline [17, 18]. The antibacterial activities of UA are realized by destroying the integrity of the bacterial membrane and inhibiting the metabolic and protein synthesis pathway [19]. Meanwhile, UA can improve intestinal flora imbalance and play a protective role in the intestinal tract of liver fibrosis mice [20–22]. The character of UC is intestinal immune imbalance and intestinal microbial disorder [23, 24]. The infiltration of macrophage, dendritic cells, and T cells plays crucially important roles in the dextran sulfate sodium- (DSS-) induced mouse UC model [25–27]. The commensal bacteria diversity in UC patient pattern decreases, particularly in Firmicutes and Bacteroides, but some bacterial species are a relative increase like Enterobacteriaceae [28–30].

In our study, we hypothesized that UA may improve the microenvironment of intestinal flora and regulate the infiltration of immune cells to prevent ulcerative colitis. So, we used the DSS-induced mouse UC model to investigate the protective effects of UA against ulcerative colitis.

## 2. Materials and Methods

### 2.1. Reagents

Reagent-grade DSS salt (MW, 36-50 kDa, MP Biomedicals); UA (purity, 99.27%, MedChemExpress); Fast DNA Spin Kit for feces (6570200, MP Biomedicals); Qiagen RNeasy Kit (74104, Qiagen); BD™ Cytometric Beads Array (BD).

### 2.2. DSS Induced the Mouse UC Model and UA Treatment

**Figure 1:**
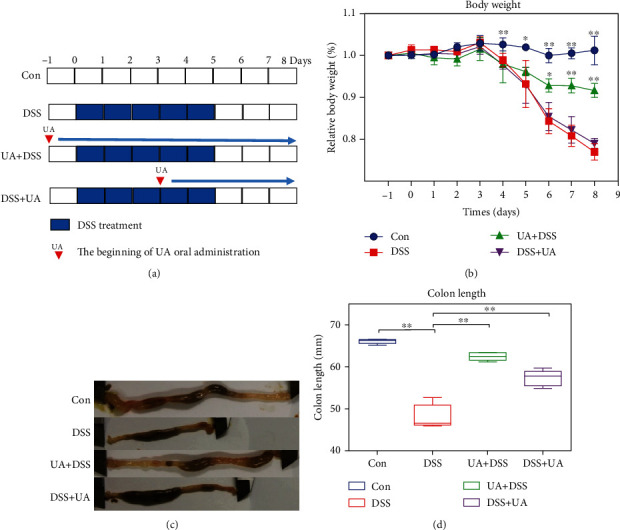
Effects of the UA on DSS-induced UC model. (a) Flow chart and time point of the DSS-induced UC model and UA treatment. The blue frame represented a 3% DSS drinking solution for one day. (b) The daily record of body weight in the DSS-induced UC model and UA treatment groups (*n* = 8). (c) The morphology and length of the colon in the DSS-induced UC model and UA treatment groups. (d) The box plot of colon length in the DSS-induced UC model and UA treatment groups (*n* = 8). Data represent the mean ± SD of values per group. Statistically significant results in different groups are marked by ^∗^*P* < 0.05 and ^∗∗^*P* < 0.01. There was no significant difference in the unmarked group.

The 6-week-old C57/BL6 (male, 18-20 g) were provided and fed a basal diet at 24°C and 55% humidity with 14 : 10 light-dark cycle in SPF laboratory animal facility according to the approval of the Animal Ethics Committee of Zhejiang Academy of Traditional Chinese Medicine (KTSC20200058). After a week of adaptation, mice were divided into 4 groups (*n* = 8) according to the experimental plan. In our experiment, all mice were randomly divided into four groups: control group (Con group), model group (DSS group), preventive treatment group (UA + DSS group), and treatment group (DSS + UA group) (Figure 1(a)). The DSS-induced UC model was given 3% (w/v) DSS in the drinking water for 5 days ad libitum. UA (200 mg/kg body weight) was administered by oral gavage once a day for 7 days, commencing 24 hours before changing 3% (w/v) DSS of the drinking water in the UA + DSS group. In the DSS + UA group, UA (200 mg/kg body weight) was administered by oral gavage once a day for 4 days, commencing 3 days after changing 3%(w/v) DSS of the drinking water. The vehicle was administered by oral gavage in the Con group and DSS group. The mice were weighed daily. The mice were anesthetized to collect whole blood from the hearts of mice and sacrificed to collect rectal feces and colonic tissues for follow-up experiments.

### 2.3. Histopathological Analysis and Serum Cytokine Measurement

The colonic tissue of mice was fixed with 4% formalin, embedded in paraffin, and then stained with hematoxylin and eosin (HE). The score of Nancy index was performed according to the Marchal-Bressenot method [31]. The Nancy index is defined by 5 level classification ranging from grade 0 (no significant disease activity) to grade 4 (severely active disease). The whole blood in the EP tube was left at room temperature for more than one hour and centrifuged at 1500 g for 20 minutes, and the supernatant was the serum. The 9 cytokine (IL-6, IL-10, MCP-1, TNF, IFN-*γ*, IL-17, IL-2, GM-CSF, and IL-4) levels were measured using the BD™ Cytometric Beads Array (CBA) according to manufacturers' protocol.

### 2.4. Transcriptome Analysis of Colonic Tissues

Total RNA was isolated from 3 colonic tissues of each group using the Qiagen RNeasy kit following the manufacturers' protocol. RNA samples with good quality control (RIN values > 8) were sequenced using Hiseq-2500 by Novogene. The raw data of RNA-seq was inspected using FastQC and mapped to the reference genome (GRCm38). The read count of genes was calculated by Hisat2 [32]. The expression of genes was analyzed by principal component analysis (PCA). According to a different group, colon length, and Nancy index, all genes were clustered by weighted gene coexpression network analysis (WGCNA) [33]. The clusters of genes were annotated by GO and KEGG by clusterProfiler [34]. Based on the RNA-seq data, the immune cell inflation was analyzed by seq-ImmuCC, which is a tool of tissue transcriptome measuring cellular compositions of the immune microenvironment from mouse RNA-seq data [35].

### 2.5. Intestinal Microbiota 16S rRNA Sequence

The total DNA of the rectal feces was isolated using the Fast DNA Spin Kit for feces. The V4 region of the 16S rRNA gene was amplified and sequenced with the 515F/806R primer set by Illumina MiSeq platforms at Novogene. The operational taxonomic units (OTUs) and representative sequences for each OTU were obtained at 97% similarity by FLASH, QIIME, and UPARSE software [36–38]. The species of OTUs sequence were annotated at setting a threshold of 0.8 ~ 1 by the Mothur method and SILVA132 SSUrRNA database (http://www.arb-silva.de/) [39]. And then, the abundance of species, Alpha diversity of each group, was calculated by *R* software.

### 2.6. Real-Time RT-PCR

Briefly, 1 *μ*g of extracted RNA was reverse transcribed (Applied Biosystems) and amplified using the SYBR green PCR master mix (Roche 480). The relative quantification of the gene expression was calculated with the 2 − *ΔΔ*Ct method referring to Gapdh.

### 2.7. Immunoblot Analysis

The colonic tissues were washed with ice-cold PBS and lysed with lysis buffer (20 mM Tris at pH 7.5, 1 mM PMSF, 0.1% Triton X-100, and 10 *μ*g/ml aprotinin). The concentration of protein was determined using a BCA assay (Sangon Biotech), and 20 *μ*g of protein per lane was added on an 8-12% SDS-polyacrylamide gel. The protein electrophoretically transferred to a nitrocellulose membrane (0.1-*μ*M pore size). The proteins were detected using rabbit polyclonal antibodies against mouse Tgfb-1, Col1a1, Itga5, and Gapdh (Proteintech Group) as primary antibodies and peroxidase-conjugated anti-rabbit IgG (Proteintech Group) as a secondary antibody. Protein was detected by an enhanced chemiluminescence system (ECL) and exposure to X-ray film.

### 2.8. Statistical Analysis

The Image-Pro Plus software was used to calculate the score of Nancy index. Image production and data analysis were performed using GraphPad Prism and *R* software. All data are presented as mean ± SD or SEM. The number of each experimental group was at least 3 samples to ensure confidence in the results. Student's *t*-test and Kruskal-Wallis *H* test were used to analyze the significant differences between groups. ^∗^*P* < 0.05 was considered significant. ^∗∗^*P* < 0.01 was considered extremely significant.

## 3. Results

### 3.1. Ursolic Acid Attenuated DSS-Induced Ulcerative Colitis of Mice

**Figure 2:**
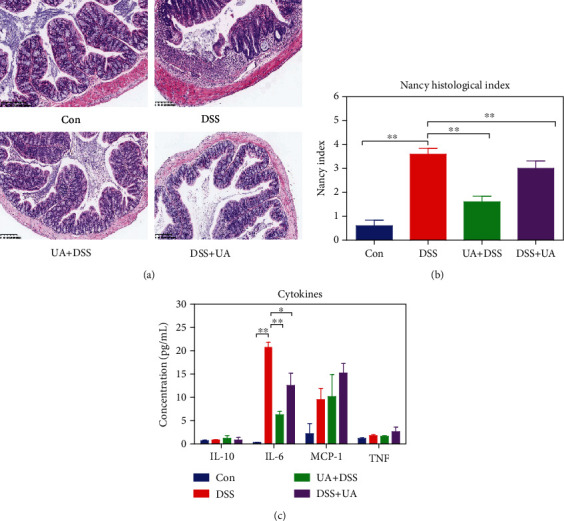
Effects of the UA against colon inflammatory and injure. (a) The histopathology of the colon by HE stain in the DSS-induced UC model and UA treatment groups. (b) Nancy index of each group according to the Marchal-Bressenot method in each group (*n* = 8). (c) Effects of UA on serum levels of four cytokines in the DSS-induced UC model and UA treatment groups (*n* = 8). Data represent the mean ± SEM of values per group. Statistically significant results in different groups are marked by ^∗^*P* < 0.05 and ^∗∗^*P* < 0.01. There was no significant difference in the unmarked group.

To investigate the effect of UA on prevention and treatment for UC, the DSS-induced mouse UC model was performed by oral administration of UA before and after DSS treatment. Early intervention with UA (UA + DSS group) was able to alleviate DSS-induced weight loss and shortening the colon in mice (Figure 1). However, the DSS + UA group only reduced DSS-induced shortening of the colon in mice for UA treatment five days (Figure 1). The histopathology of the colon was evaluated by the score of Nancy index and HE. The Nancy index of the UA + DSS group and DSS + UA was significantly lower than the DSS group (Figure 2(b)). The 3% DSS induced severe mucosal and epithelial cell structural damage and inflammation response (Figure 2). However, the mucosal epithelium was more intact and regularly arranged between the UA + DSS and DSS + UA groups than the DSS group (Figure 2(a)). The UA also reduced the level of proinflammatory factor IL6 in serum (Figure 2(c)). So, our results implied that UA had protective and therapeutic effects on colon damage and inflammation.

### 3.2. Core Gene Expression Characteristics of UA Attenuated DSS-Induced Mouse UC

**Figure 3:**
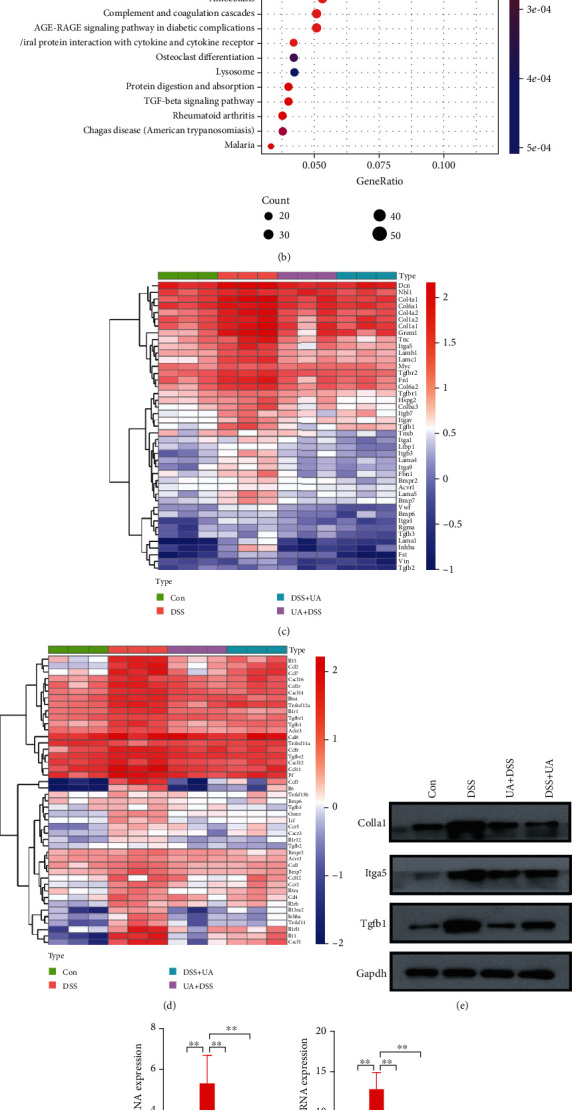
Transcriptome analysis and protein expression of the prevention and treatment of the DSS-induced UC model with UA treatment. (a) PCA analysis results of transcriptome under different treatments. (b) The KEGG signaling pathway enrichment results of black module genes by WGCNA. (c) Heatmap of ECM receptor interaction and TGF-*β* signaling pathway gene expression. (d) Heatmap of the cytokine-cytokine receptor signaling pathway gene expression. (e) Immunoblot analysis of Col1a1, Tgfb-1, and Itag5 in four groups of colonic tissues. (f) The relative mRNA expression of Il6, Tgfb-1, Ccr-2, and Csf-1 gene in four groups of colonic tissues.

**Figure 4:**
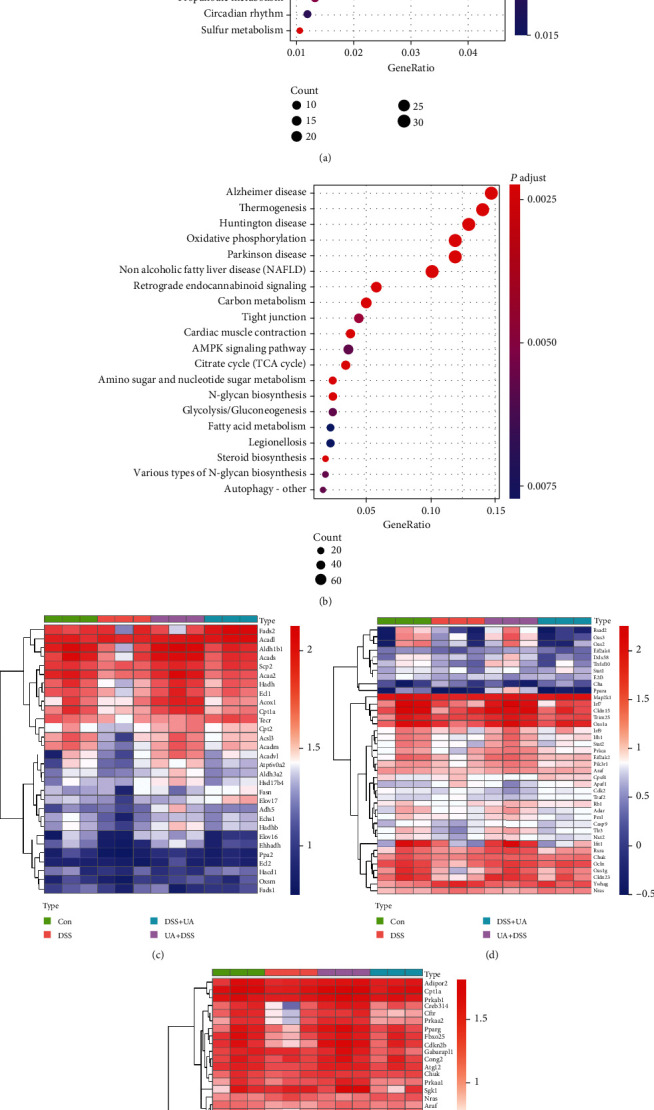
Gene expression modules associated with UA therapy and prevention. (a) The KEGG signaling pathway enrichment results of blue module genes associated with the phenotype of the UA + DSS group. (b) The KEGG signaling pathway enrichment results of red module genes associated with the phenotype of the DSS + UA group. (c) Heatmap of fatty acid metabolism signaling pathway gene expression. (d) Heatmap of the virus infection signaling pathway gene expression. (e) Heatmap of the AMPK and FoxO signaling pathway gene expression.

To state the molecular mechanism of UA treatment on UC, we analyzed the transcriptome of four groups (Con, DSS, UA + DSS, and DSS + UA) by high throughput sequencing (GSE150688). The Con and UA + DSS groups were clustered, while DSS and DSS + UA groups were individually separated according to transcriptomic data by PCA analysis (Figure 3(a)). The specific gene coexpression modules of four different treatment groups were analyzed by WGCNA. The 15 gene coexpression modules were found using calculating parameter (power = 14 and mergecutheight = 0.25) (Figure 1S and Table 1S). The gene expression profile of the black module was significantly positively correlated with the DSS group (correlation = 0.97, *P* value = 2*e* − 07). The genes of the black module were clustered into inflammation signaling pathways such as PI3K-Akt, MAPK, and cytokine interaction (Figure 3(b)). The ECM (extracellular matrix) and TGF-*β* signaling pathway were activated by DSS (Figure 3(c)). The blue module was significantly positively correlated with the UA + DSS group (correlation = 0.64, *P* value =0.02) and negatively correlated with the DSS group (correlation = -0.61, *P* value =0.04) (Figure 1S). The genes of the blue module were mapped into fatty acid metabolism, bile secretion, and virus infection pathways by KEGG analysis (Figures 4(a) and 4(c)–4(e)). The red module was significantly positively correlated with the DSS + UA group (correlation = 0.59, *P* value = 0.05) and negatively correlated with the DSS group (correlation = −0.65, *P* value = 0.02) (Figure 1S). The genes of the red module were clustered into neurological disease, oxidative phosphorylation, and fatty acid metabolism in (Figure 4(b)). Interestingly, We found gene modules (blue and red) associated with phenotypes that can be enriched to fatty acid metabolism in both the preventive treatment group (UA + DSS group) and treatment group (DSS + UA group) (Figures 4(a) and 4(b)). We also found that the gene expression of fatty acid metabolism and fatty acid degradation in the UA-treated group and the normal control group was higher than that in the DSS model group (Figure 4(c)). Meanwhile, AMPK and FOXO signaling pathways were downregulated in the DSS group compared with UA treatment and Con groups (Figure 4(d)). These results suggested that the protection and treatment effect of UA on UC is mainly through the regulation of fatty acid metabolism.

### 3.3. UA Regulates Immune Cell Infiltration of DSS-Induced Mouse UC

**Figure 5:**
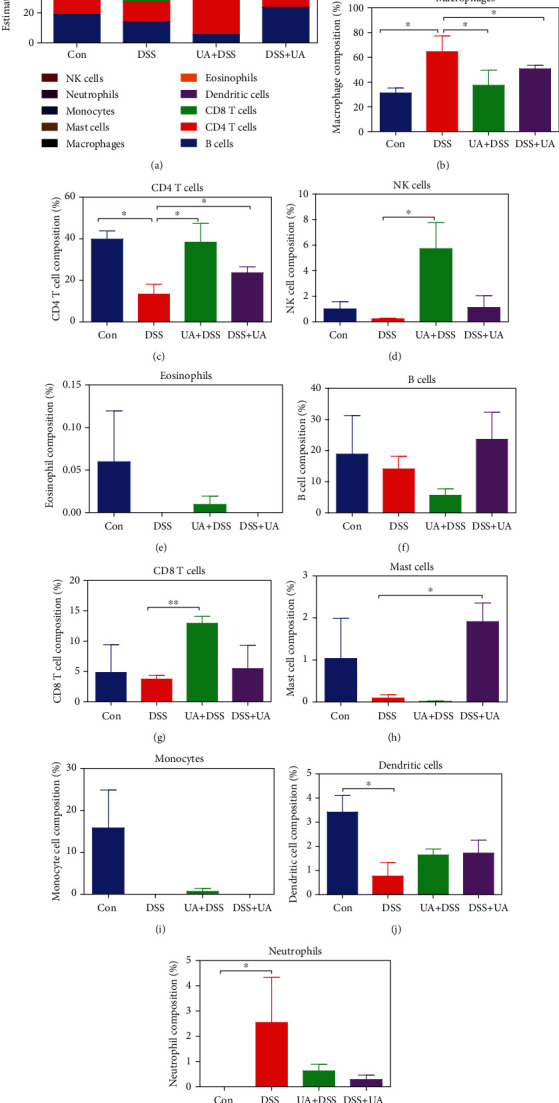
Composition of infiltrated immune cells among four UC experiment groups. (a) Fractions of 10 immune cells in Con, DSS, UA + DSS, and DSS + UA groups (*n* = 3 each group). (b) The proportion of macrophage change in four experimental groups. (c) The proportion of CD4 T cells change in four experimental groups. (d) The proportion of NK cell change in four experimental groups. (e) The proportion of eosinophil change in four experimental groups. (f) The proportion of B cell change in four experimental groups. (g) The proportion of CD8 T cell change in four experimental groups. (h) The proportion of mast cell change in four experimental groups. (i) The proportion of monocyte cell change in four experimental groups. (j) The proportion of dendritic cell change in four experimental groups. (k) The proportion of neutrophil change in four experimental groups. Data represent the mean ± SEM of values per group. Statistically significant results in different groups are marked by ^∗^*P* < 0.05 and ^∗∗^*P* < 0.01. There was no significant difference in the unmarked group.

To understand the composition of infiltrated immune cells in inflammatory microenvironments Figure 5(h) of different treatment groups, the transcriptomic data were analyzed by seq-ImmuCC. Compared with the Con group, the proportion of macrophages and neutrophils in the DSS group was significantly increased, while the proportion of CD4 T and dendritic cells was decreased significantly (Figures 5(a)–5(c)). The proportion of macrophages and neutrophils was decreased, and the proportion of CD4 T and dendritic cells was increased in the UA treatment group including UA + DSS and DSS + UA (Figures 5(a), 5(b), and 5(k)). The proportion of NK and CD8 T cells was significantly increased in the UA + DSS group (Figures 5(d) and 5(g)). The proportion of mast cells was increased in the DSS + UA group (Figure 5(h)). These results suggested that UA could regulated immune cell infiltration in the UC mouse model.

### 3.4. UA Regulates the Microenvironment of the Intestinal Flora of DSS-Induced Mouse UC

**Figure 6:**
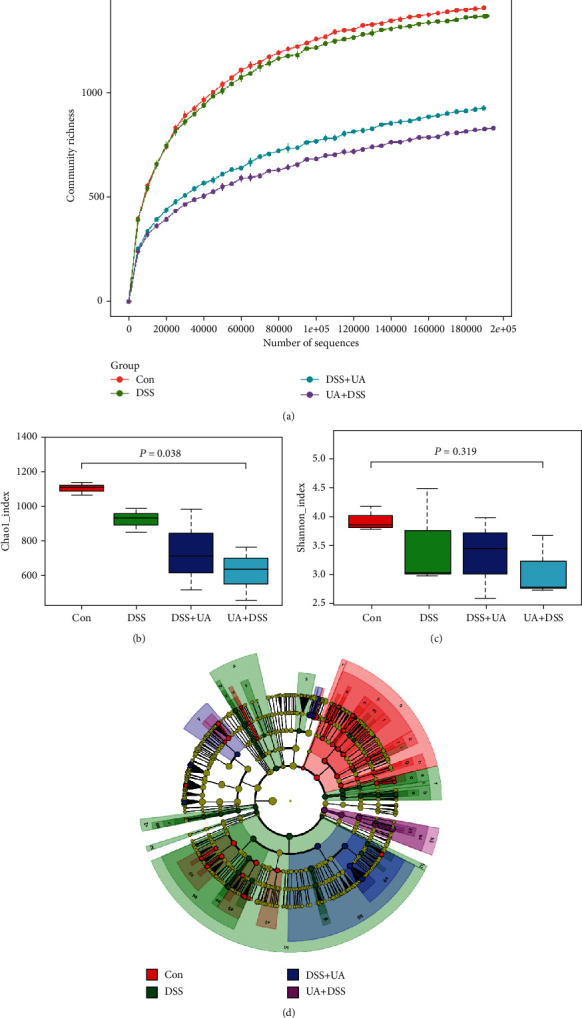
Analysis of the differential microbial community among the group. (a) Rarefaction curve of microbial community richness of Con, DSS, UA + DSS, and DSS + UA groups. (b) Alpha diversity index (Chao1_index) of intestinal within groups. (c) Alpha diversity index (Shannon_index) of intestinal within groups. (d) Cladogram of the LDA value from the Con, DSS, UA + DSS, and DSS + UA groups. The Kruskal-Wallis *H* test was used to analyze the significant differences between groups by *R*.

To investigate the regulatory effect of UA on intestinal microorganisms, the observed species and diversity were analyzed by 16S rRNA amplicon sequencing. We found that UA could significantly reduce the community richness of bacteria and the Chao index (*P* value = 0.038) in the gut (Figure 6(a) and 6(b)). However, there was no significant difference in the Shannon index (*P* value = 0.319) (Figure 6(c)) and the Beta diversity index (data not shown). To determine the bacteria biomarkers of each treatment group, the LDA (Linear Discriminant Analysis) effect size of four groups was analyzed by LEfSe software. The Con and DSS groups owned more biomarkers, and the biomarkers were reduced by UA treatment (Figure 6(d)). The major biomarker of UA + DSS was Verrucomicrobia, while the major biomarker of the DSS + UA group was Gammaproteobacteria (Figure 6(d)).

## 4. Discussion

Inflammatory bowel disease (IBD) has been a global disease leading to low life-quality and high societal costs [40]. UC and Crohn's disease (CD) are the two main IBD pathological subtypes. In contrast to CD, UC lesions occur mainly in the mucosa of the colon due to genetic immune, environmental factors, and intestinal microbes [2]. In this study, the early prevention (UA + DSS group) and late treatment (DSS + UA group) effects of UA were analyzed by the DSS-induced UC mouse model. The early prevention effect of UA could effectively delay mouse weight loss and colon length shorten (Figure 1). However, the late treatment effect of UA only delayed colon length shorten, and there was no significant difference in body weight between the DSS + UA group and the DSS group (Figure 1), but the no difference in body weight might be caused due to short UA administration time (only 5 days). However, in previous studies, UA treatment for 7 days could improve SOD activity and reduce malondialdehyde (MDA) and myeloperoxidase (MPO) activity to relieve the reduction of body weight and stool blood [12]. Meanwhile, UA treatment for 9 days in the UA + DSS group showed significant weight recovery (Figure 1(b)). So, prolonged administration of UA may contribute to weight recovery in UC mice.

To further elaborate the mechanism of UA in the prevention and treatment of UC, the correlation between the transcriptome and the phenotypes of each treatment group was analyzed by WGCNA. Three classical inflammatory pathways: MAPKs, IL-6/STAT3, and PI3K were enriched into the black module which was significantly positively correlated with the DSS group (Figure 3(b) and 1S). Mitogen-activated protein kinases (MAPKs) involve in the regulation of the synthesis of inflammation mediators by transcription and translation [41]. PI3K isoforms (PI3K*α*, *β*, *γ*, *δ*) play a particularly important role in chemokine-mediated recruitment and activation of innate immune cells at sites of inflammation and B and T cell development, differentiation, and function [42]. UA could also significantly reduce the IL-6 level in serum and downregulate the expression of inflammation-related genes (Figures 2(d), 3(c), and 3(d)). IL-6 regulates various cells including epithelial cells, macrophages, neutrophils, and T cells to activate early immune responses [43]. In IBD patients, multiple aberrancies in lipid metabolism have been found, and fatty acids may affect cytokine production and inflammation response [44–46]. AMP-activated protein kinase (AMPK) plays a key role as a master regulator of cellular energy homeostasis and is also thought to be important for regulating fatty acid metabolism [47]. The activation of the AMPK-FOXO3 pathway reduces the fatty acid-induced increase in intracellular reactive oxygen species [48]. In our results, the prevention and treatment effects of UA on UC are achieved by activating AMPK/FOXO signaling pathways that upregulate fat acid metabolism (Figure 4).

Immune cell infiltration is an important biomarker of inflammation. DSS feeding resulted in an increased production of macrophage-derived cytokines in BALB/c mice [26]. We also found macrophages accounted for the highest proportion of immune infiltrating cells in the DSS-induced UC model (Figures 5(a) and 5(b)). The proportion of macrophages was significantly reduced in two UA treatment groups (Figure 5(b)). UA inhibits NF-*κ*B activation in both intestinal epithelial cells and macrophages and attenuates experimental murine colitis [13]. However, the infiltration of neutrophils in IBD leads to loss of barrier function and apoptosis of epithelial cells [49]. UA treatment reduced the proportion of neutrophils in the colonic mucosa of DSS-induced UC models (Figure 5(k)). CD4 T cells, also known as T helper (Th) lymphocytes, differentiate into a variety of Th cell types and are key in mediating inflammation [50]. In our model, the proportion of CD4 T cells was downregulated in the DSS-induced UC model, while UA could mitigate this decline in the ratio (Figure 5(c)). However, the types of these CD4 T cells still need further analysis.

**Figure 7:**
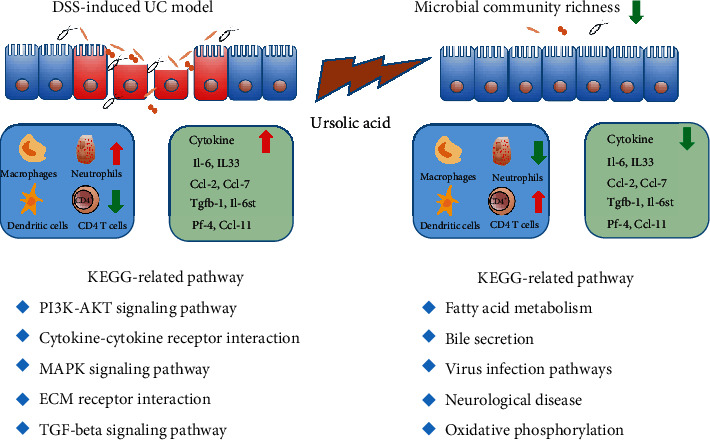
Schematic representation of UA function on the prevention and treatment of UC. DSS induced injury to the intestinal epithelial immune barrier, leading to the transfer of antigens into gut lamina propria. The red arrow means upregulation, and the green means downregulation.

At present, there is no single agent that has been proven to cause IBD. The role of gut microbes has been suspected because of potential infectious, particularly when the intestinal epithelial barrier is destroyed [51]. UA has a potential antibacterial effect by inhibition of protein synthesis and the metabolic pathway [18]. In our studies, the community richness of bacteria, Chao index, and bacteria biomarkers were markedly decreased in two UA treatment groups (Figure 6). This reduction of community richness of bacteria would reduce the innate immune response and inflammation due to the destruction of the epithelial barrier (Figure 7). In studies on liver fibrosis, it has been found that UA could prevent intestinal damage caused by carbon tetrachloride by improving intestinal flora disturbance [19–21]. In our research, the major biomarker of the UA + DSS group was Verrucomicrobia by LEfSe (Figure 6(d)). Meanwhile, Verrucomicrobia has been recently proposed as a hallmark of a healthy gut due to its anti-inflammatory and immunostimulant properties and its ability to improve gut barrier function, insulin sensitivity, and endotoxemia [52].

In conclusion, we demonstrated that UA could prevent and ameliorate the DSS-induced UC mouse model. The function of UA against UC was through reducing intestinal flora abundance, regulating inflammatory and fatty acid metabolism signaling pathways to affect immune cell infiltration and cytokine expression (Figure 7). These results suggested that IBD susceptible populations would eat some foods or drink herb tea rich in UA such as apple, berries, and mulberry leaf tea to prevent and treat IBD. Of course, how much UA content through diet and tea per day is still to be further studied to prevent UC.

## Data Availability

The data used to support the findings of this study are available from the corresponding author upon request. The transcriptome data of this research have been submitted GEO database (Accession: GSE150688).

## Supplementary Materials

Supplementary 1 Figure 1S. Relationship between gene expression modules and phenotype of experiment groups. The 15 gene expression modules were found by WGCNA. The upper number of each frame represented the correlation value. The lower number of each frame standard for significance *P* value. The abscissa represents different groups and phenotypes, and the ordinate represents related gene expression modules.

Supplementary 2 Table 1S. The table of the related gene in 15 gene expression modules by WGCNA.
