# Supplementary figures and images for: Ursolic Acid Regulates Intestinal Microbiota and Inflammatory Cell Infiltration to Prevent Ulcerative Colitis
Source: J Immunol Res. 2021 Apr 30;2021:6679316. doi: 10.1155/2021/6679316 (PMC8111854; doi:10.1155/2021/6679316)

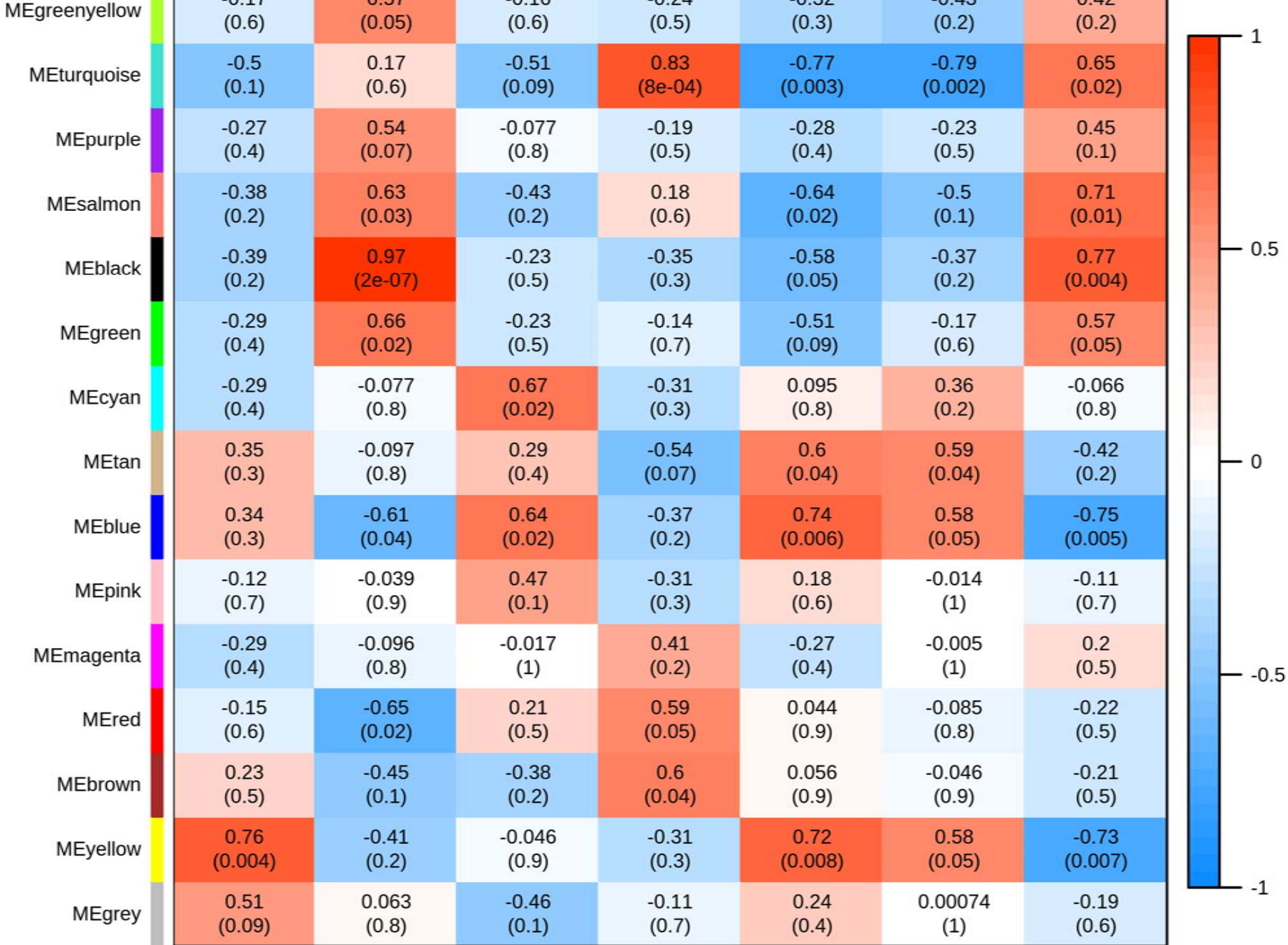

Supplement: Supplementary 1 — Figure 1S. Relationship between gene expression modules and phenotype of experiment groups. The 15 gene expression modules were found by WGCNA. The upper number of each frame represented the correlation value. The lower number of each frame standard for significance P value. The abscissa represents different groups and phenotypes, and the ordinate represents related gene expression modules. [file 6679316.f1.pdf]
