# Supplementary material for: Ursolic Acid Regulates Intestinal Microbiota and Inflammatory Cell Infiltration to Prevent Ulcerative Colitis
Source: J Immunol Res. 2021 Apr 30;2021:6679316. doi: 10.1155/2021/6679316 (PMC8111854; doi:10.1155/2021/6679316)
Supplement: Supplementary 2 — Table 1S. The table of the related gene in 15 gene expression modules by WGCNA. [file 6679316.f2.docx]

| **Black** | **Blue** | **Brown** | **Cyan** | **Green** | **Greenyellow** | **Magenta** | **Pink** | **Purple** | **Red** | **Salmon** | **Tan** | **Turquoise** | **Yellow** |
| --- | --- | --- | --- | --- | --- | --- | --- | --- | --- | --- | --- | --- | --- |
| Igha | Pigr | Eef1a1 | Reg3b | Fth1 | Ctrb1 | H2-D1 | S100a6 | Apoe | Lypd8 | Mfge8 | Tpm1 | Hspa8 | mt-Co1 |
| Igkc | Dmbt1 | Tpt1 | Reg3g | Actb | Cela1 | Plac8 | Nedd4 | Gsn | Krt8 | Cxcl13 | Myh11 | Actg1 | mt-Cytb |
| Jchain | Car1 | Rplp0 | Fgl2 | Zg16 | Zfp36l2 | Sprr2a1 | Csnk1a1 | Mgam | Lgals4 | Clu | Acta2 | Calm1 | mt-Nd1 |
| Col3a1 | Muc2 | Rpl4 | 2210407C18Rik | Tmsb4x | 2010109I03Rik | Cd74 | Fam120a | Iglv1 | Eef2 | Ighm | Actg2 | Hsp90ab1 | mt-Nd4 |
| Psap | Ceacam1 | Rpsa | Ighv1-82 | Fcgbp | Cela2a | Ly6e | Gnb2 | Iglc1 | Krt19 | Mgp | Des | Ldha | mt-Nd5 |
| Ctsb | Atp1a1 | Rpl41 | Car3 | S100a9 | Pnlip | Tmsb10 | Eif1 | C4b | Atp5b | Cerk | Myl9 | Scd2 | mt-Nd2 |
| Tax1bp1 | H2-K1 | Rps2 | Prr5 | S100a8 | Igkv1-117 | Cpd | Btg1 | 9530053A07Rik | Epcam | Ptp4a3 | Mylk | Pkm | mt-Rnr2 |
| Myh9 | Agr2 | Rplp1 | Nlrc5 | Ezr | Cpb1 | Ctss | Sptan1 | Ighg2b | Muc13 | Pou2af1 | Tpm2 | Ptma | P4hb |
| Flna | B2m | Rps3a1 | Tph1 | Mxd1 | Cpa1 | Cd177 | Kif5b | Man1a | Atp5a1 | Bin1 | Cnn1 | Ybx1 | mt-Nd6 |
| Dcn | Spint2 | Prdx6 | Igkv2-109 | Mcl1 | Prss2 | Rpl17-ps10 | Eif4ebp2 | Gcg | Lgals3 | Cd79a | Cpe | Hnrnpa2b1 | Car2 |
| Eif4b | Emp1 | Rpl13 | Gzma | Saa3 | Cel | Smpdl3a | Tcf25 | Iglc2 | Dstn | Ppp2r5b | Dmpk | Eif4g2 | Ahnak |
| Lyz2 | Gfpt1 | Rpl7 | Asb2 | Anxa1 | 2210010C04Rik | Huwe1 | Dag1 | Ighv7-3 | Slc25a5 | Ighd | Igkv6-23 | Pdia3 | Txnip |
| Sparc | Anxa2 | Rack1 | Cx3cr1 | Ehf | Sfrp1 | H2-Ab1 | Sat1 | Wdr33 | Tspan1 | Cd79b | 5-Mar | Gpx2 | mt-Rnr1 |
| Igfbp5 | Atp1b1 | Rps3 | Casp12 | Slc12a2 | Ighv1-81 | H2-Aa | Rhob | Ighv1-9 | Myl6 | Ccdc137 | Cfl2 | Itm2b | Arhgdia |
| Col4a1 | Tgoln1 | Rps6 | Ighv5-16 | Pdxdc1 | Try4 | Clta | Arfgef1 | Igkv10-94 | Krt20 | Spib | Smtn | Pabpc1 | Neat1 |
| Col1a2 | Cyp2c55 | Rps24 | Apol7c | Iqgap1 | Pnliprp1 | Neurl3 | Cnot1 | Pbsn | Tmbim6 | Cdc14b | Ppp1r12b | H3f3b | Rac1 |
| Igfbp4 | Guca2a | Rpl10 | Slc46a3 | Gnai2 | Try5 | H2-Eb1 | Zfp91 | Igkv6-15 | Aldoa | Ess2 | Synpo2 | Calr | Ivns1abp |
| Tspan3 | Atp2a3 | Muc3 | Igkv5-39 | Ube2d3 | Col12a1 | Hk2 | Hook1 | Hba-a1 | Slc25a3 | 6-Sep | Atp2b4 | Ly6a | Gnb1 |
| Cd2ap | Cdh1 | Rps9 | Ighv5-6 | Slc35a3 | Reg2 | Plet1 | Mbd2 | Cfd | St6galnac6 | Cd22 | Synm | Eif5a | Lasp1 |
| Csrp1 | Cfl1 | Rpl8 | Nphp1 | S100a11 | Igkv1-110 | Chmp2a | Ncor1 | Fmo2 | Ddx5 | Snx8 | Pdlim3 | Eef1g | Tagln2 |
| Golph3l | Clca1 | Rps18 | Gulp1 | Il1b | Cela3b | Duox2 | Raly | Gpnmb | Cox4i1 | Ly6d | Pgm5 | Hspa5 | Eif4h |
| Plcb3 | Bsg | Rpl27a | Art2a-ps | Picalm | Clps | Herpud1 | Acp5 | Igkv12-44 | Cldn7 | Ms4a1 | Igkv3-2 | Serinc3 | Taldo1 |
| C3 | Pfn1 | Rpl3 | Igkv8-19 | Cd14 | Lum | Carhsp1 | Rps6ka3 | Igkv4-91 | Actn4 | Nle1 | Marveld1 | Arf1 | Gas6 |
| Serpina3n | Klk1 | Rpl5 | Itgae | Ddx6 | Ighv6-3 | Psmb10 | Map1lc3b | Gapdh | Hnf4a | Tsr2 | Gm12751 | Ftl1 | Ccni |
| Lrp1 | Ctnnb1 | Txn1 | Rab37 | Rab7 | Ctrl | Irf1 | Lpp | Tgm4 | B3galt5 | H2-Ob | Angptl2 | Prdx1 | Xbp1 |
| Mt1 | 1810065E05Rik | Rps14 | Pdgfc | Sypl | Igkv5-48 | Rab8a | Cyb5r3 | Igkv1-135 | Cox6a1 | Cd19 | Flnc | Tpi1 | Tmed2 |
| Calu | Maoa | Oit1 | Gbp8 | Thbs1 | Rnase1 | Prrc2c | Celf1 | P2rx7 | Stard10 | Timeless | Zfp280b | Eno1 | Ddx17 |
| Col1a1 | Selenbp1 | Rps4x | Cd7 | Ets2 | Adgrl2 | Psme1 | Ctsa | Igkv9-120 | Tspan8 | Snn | Rabep2 | Eif4a1 | Eif4a2 |
| Fn1 | Cmpk1 | Rps8 | Ceacam12 | Cxcl2 | Reg1 | Stx7 | Slmap | Apod | Tmprss2 | Cd3eap | Eml1 | Actr3 | Mvp |
| Mboat1 | Hmgcs2 | Rps5 | Mapkapk5 | Prdx5 | Cdc42ep1 | Nsd1 | Ist1 | Igkv4-55 | Cdh17 | Diexf | Mrvi1 | Akr1a1 | H1f0 |
| Igfbp7 | Aes | Rps7 | Plet1os | Tpm4 | Amy2b | Snx1 | Atp6v1g1 | Ighv5-17 | Atp5g3 | Ikzf3 | Ang4 | Pgk1 | Slc16a1 |
| Esrp1 | Aqp4 | Rpl19 | Ctsw | Sqstm1 | Cpa2 | Ensa | Top2b | Igkv19-93 | Golm1 | Zfp251 | Jph2 | Tpd52 | Srrm2 |
| Pmm2 | Txndc5 | Ckmt1 | Adipoq | Vim | Gp2 | Upp1 | Cep170b | Hfe | Tm9sf2 | Ptprcap | Armcx3 | Ncl | Mat2a |
| Ctsd | Klf4 | Rpl12 | Thrsp | Gsr | Igkv8-30 | AW112010 | Fam91a1 | Cd83 | Tm9sf3 | H2-DMb2 | Id4 | Mlec | Ankrd13a |
| Col4a2 | Cd24a | Rps26 | Abhd3 | Slfn4 | Amy2a3 | Psme2 | Trir | Igkv6-32 | Ucp2 | Nfatc2 | Klhl23 | Tubb4b | Srsf5 |
| Ctsc | Myo15b | Rpl6 | Klrd1 | Glud1 | Ctgf | Irf8 | Nf2 | Ighv2-2 | Calm2 | Pax5 | Klc2 | Hsp90b1 | Uba1 |
| Zfp706 | Rrbp1 | Rpl18 | Apol10c-ps | Junb | Rfx1 | Atp6v0d1 | Rap1a | Ighv2-9-1 | Vil1 | Tnfrsf13b | Tspan2 | Arpc1b | Akt1 |
| Kmt5a | Slc26a2 | Rps23 | Tcrg-C2 | Hp | Fndc1 | Rabac1 | Atrnl1 | Igkv8-24 | Gpa33 | Hvcn1 | Stum | Eif4g1 | Capza2 |
| Tmem30b | Nudt4 | Rpl18a | Wif1 | Fhl1 | Ighv8-12 | Ctsh | Ankrd17 | Hbb-bs | Cox8a | Kri1 | Ttll7 | Hnrnpk | Gnai3 |
| Tmem176b | Slc6a14 | Rpl11 | Ighv1-37 | Myo6 | Amy2a1 | Id3 | Cul1 | Igkv13-84 | Cs | Map4k1 | Rab23 | Ywhaz | Nadk |
| Ctsl | Rfk | Rps20 | Cd96 | Adipor1 | Iffo2 | Rnf130 | Ankfy1 | Cyp2e1 | Calm3 | Rasgrp3 | Tmtc1 | Npm1 | Eif3c |
| Col18a1 | Dsp | Rpl10a | Retn | Lcn2 | Mfap1a | Tifa | Osbp | Ttyh1 | Serpinb6a | Pigb | Plekhh2 | Cdc42 | Postn |
| Lgmn | Slc26a3 | Ppia | Gbgt1 | Rap1b | Ighv8-8 | Tap1 | Prkci | 9530002B09Rik | Gdi2 | Card11 | Ighv2-6 | Vasp | Rbm39 |
| Gpx3 | Myh14 | Rps15 | Gm10509 | Lamp2 | Amy2a5 | Egln2 | Dpp8 | Ighv7-1 | Lamp1 | Wdr91 | Mtss1l | Hnrnpu | Crk |
| Kdelr2 | Cdhr5 | Rpl9 | AA414768 | Chd4 | Maged2 | Necap2 | Ier2 | Ighv14-3 | Chchd2 | Hspa2 | Myom1 | Serp1 | Trak1 |
| Bgn | Glul | Rps16 | Igkv1-122 | Creb3l1 | Cyr61 | Sin3b | Sec16a | Ighv2-5 | Spink4 | Slc6a9 | Sspn | Cltc | Gprc5a |
| Tgfbr2 | Cd63 | Rps19 | Tcrg-C1 | Gda | Lama2 | Psmb8 | Sec63 | Ighv1-78 | Ptp4a2 | Cr2 | Cryab | Serbp1 | Selenot |
| Gss | Sptbn1 | Rpl32 | Igkv12-89 | Lcp1 | Fbln5 | Tax1bp3 | Apc | Ighv9-2 | Gpi1 | P2ry10 | Ptn | Oaz1 | Sult1a1 |
| Cald1 | Ahcyl2 | Fau | Tcrg-C4 | Ppp1r15b | Pnliprp2 | Ost4 | Stk38 | Ccl21a | Myo1a | Bank1 | Ighv1-52 | Gpx1 | Dnajc10 |
| Col6a1 | Atp8b1 | Rpl26 | Ighv1-47 | Brd2 | Vangl1 | Igkv10-96 | Bnip3l | Gm8116 | Serpinb1a | Cxcr5 | Mturn | Selenop | Plscr1 |
| Kras | Sppl2a | Rpl28 | Gm12396 | Atf4 | Igkv3-12 | Edem2 | Larp4 | Tcaf3 | Ms4a8a | Lyl1 | Dact3 | Arpc5 | Mtpn |
| Grem1 | Ppp1cb | Rpl14 | Adig | Ganab | Ighv1-50 | Etv3 | Selenow | Igkv4-68 | Vdac2 | Tnfrsf13c | Igkv4-74 | Tpm3 | Nus1 |
| Ighg2c | Abcb1a | Rpl31 | Gm6451 | Tln1 | Bmp5 | Zc3h12a | Epb41 | Mmp7 | Hdlbp | Them6 | Fbxl22 | Flnb | Adcy6 |
| Pebp1 | Mgat4c | Rpl15 | Trgv2 | Litaf | Zbtb2 | Arrdc3 | Usp9x | Susd1 | Ctnna1 | Pus7l | Rhobtb1 | Lmna | Ptgfrn |
| Mmp14 | Ugt2b34 | Rps25 | Gm44867 | Rab3d | Igkv4-57-1 | Zc3h4 | Dnajc13 | Sfrp2 | Atp5f1 | Fcmr | Chrm2 | Clca4b | Edem1 |
| Tmem176a | Chp1 | Rps11 | Gm25777 | Tgfbi | Ighv1-22 | Med28 | Kdm5c | Ighg3 | Sqor | Fcrla | Six5 | Tkt | Nfe2l1 |
| Ifitm3 | Cdhr2 | Rps17 | Mir8103 | Cldn2 | Ighv1-7 | Ubap1 | Mapk1ip1l | Ighv1-12 | Mgst3 | Trmt112-ps2 | Cap2 | Cnbp | Clptm1l |
| Wnk1 | Dsg2 | Rps12 | Gm17241 | Fosl2 | Fgf10 | H2-Q6 | Arfgef2 | Cldn1 | Sri | Fcrl1 | Fzd2 | Hnrnpab | Slk |
| Epas1 | Ethe1 | Eef1b2 | Trbj1-1 | Sh3glb1 | Ighv1-19 | Apobec1 | Opa1 | Gm3531 | Vdac1 | H2-Eb2 | Pacsin1 | Arf4 | Nisch |
| Tnc | Tmem59 | Ubb |  | Cmtm6 | Ighv9-4 | Psmb9 | Fnbp1l | Ighv5-4 | Cldn3 | Hhex | Tmem200b | Hspa9 | Mprip |
| Col6a2 | Tfcp2l1 | Rpl23 |  | Srgn | 5830417I10Rik | Gbp2 | H2-Q4 | Clec10a | Sprr2a2 | Blk | Tyw5 | Ywhae | Coro1c |
| Akr1c14 | Slc44a1 | Rplp2 |  | Atp6ap2 | Mfap5 | Dirc2 | Kcmf1 | Nutf2-ps1 | Prss32 | Fcer2a | Fibin | Cap1 | Pafah1b1 |
| Serpinh1 | Erbin | Rpl34 |  | Rtn4 | Itm2a | Ncoa6 | Usp34 | Ighv1-4 | Atp5c1 | Pla2g2d | Igkv4-50 | Hspd1 | Pfkp |
| Il6st | Cd164 | S100a10 |  | Pxn | Ighv1-58 | Casp4 | Pbrm1 | Hba-a2 | Krt18 | H2-Oa | Sntg2 | Bzw1 | Mob1a |
| Grn | Iqgap2 | Rpl30 |  | Pygb | Ighv8-5 | Ifi47 | Ap1g1 | Hbb-bt | Gsto1 | Ccr6 | Rbpms2 | Sdcbp | Ap2m1 |
| Cxcl12 | H2-T23 | Rpl36 |  | Vmp1 | Ighv4-1 | H2-Q7 | Kif1b | Pianp | Arl1 | Vpreb3 | Gdf10 | Prelid1 | Sh3bgrl |
| Ugcg | Pmp22 | Rps27 |  | Tmod3 | Reg3a | Gba | Maged1 | Cytl1 | Atp5d | Haao | Ctxn3 | Sprr2a3 | Csnk1d |
| Idh2 | Car4 | Rps15a |  | Mapk6 | Igkv12-38 | Arid2 | Eea1 | Iglj1 | Cdx1 | BC035044 | Popdc2 | Tubb5 | D17Wsu92e |
| Ndufb10 | Papss2 | Saa1 |  | Ahcy | Cuzd1 | Ak4 | Ash1l | Alas2 | Sdha | Mrpl48-ps | Angptl7 | Actr2 | Mptx1 |
| Lypla1 | Rnasel | Rpl27 |  | Clic4 | Ighv1-61 | Gnl1 | Wtap | Igkv1-88 | Clec2h | Gm7335 | Tacr2 | Pgd | Gstm1 |
| Man2a1 | Ugdh | Rpl23a |  | Mapkapk2 | Amy2a4 | Creb3 | Tnfrsf21 | Gm10591 | Misp |  | Ighv1-66 | Rab1a | Srpr |
| Ddit4 | Cab39 | Rpl37a |  | Acat1 | Tmem45a | Dhx36 | Cand1 | Azgp1 | Plxnb2 |  | Gm527 | Ssr3 | Mapre1 |
| Ecpas | Fam83e | Nans |  | Msn | Ighv1-56 | Duoxa2 | Arl6ip5 | Gm5615 | Cox7c |  | Igkv4-61 | Arpc2 | 9130208D14Rik |
| Lgals1 | Fabp2 | Rpl39 |  | Abi1 | Reg3d | Trim40 | Usp24 | Gsdma | Cyb5b |  | Gm3604 | Arcn1 | Vps4b |
| Mpeg1 | Aqp8 | Rpl37 |  | Sorl1 | Ighv1-62-2 | Gan | Lats1 | Gpihbp1 | Ghitm |  | 1700007L15Rik | S100a16 | Snrnp70 |
| Hspg2 | C1galt1 | Rpl22 |  | Il1rn | Tff2 | Abhd12 | Trappc8 | Ighv5-2 | Aco2 |  | B130055M24Rik | Tfrc | Arf3 |
| Tug1 | Slc9a3 | Aldh2 |  | Nfkbia | Pla2g1b | Iigp1 | Skiv2l | Rps24-ps3 | Mapk3 |  | Hand1 | Clic1 | Gm8979 |
| Abhd17c | 2610507B11Rik | Rpl29 |  | Actn1 | Tmed6 | Med1 | Ppip5k2 | Inmt | Elf3 |  | Nmu | Capzb | Actr1a |
| Dpt | Sort1 | Rpl17 |  | Socs3 | Gamt | Rexo1 | G3bp2 | Ighv7-4 | Uqcrc1 |  | Oaz2-ps | Ppp1r1b | Slc1a5 |
| Timp2 | Adamdec1 | Tff3 |  | Wdr26 | Igkv5-45 | Mpzl3 | Tcf12 | Igkv2-112 | Slc44a4 |  | Igkv11-125 | Pglyrp1 | Sar1a |
| Fstl1 | 2200002D01Rik | Itgb1 |  | Retnlg | Serpini2 | Spats2l | Rbm7 | Ighv13-2 | Coro1b |  | Gm24060 | Chmp4b | Hnrnpdl |
| Lamb1 | Endod1 | Rpl35 |  | Acod1 | Crlf1 | Rab3gap2 | Alg10b | Igkv4-63 | Mdh1 |  | Mir1968 | Ywhah | Myo1c |
| Sel1l | Slc25a24 | App |  | Slpi | Igkv4-90 | Igtp | Sart1 | Ighv5-15 | Kif1c |  | Gm23205 | Rpn1 | Sycn |
| Serping1 | Ogdh | Ddx3x |  | Lrrfip1 | Igkv3-9 | Ino80 | Ube4a | Ighv1-31 | Lima1 |  | Mir143 | Txnrd1 | Hk1 |
| Anxa5 | Prom1 | Adh1 |  | Ppp2r5a | 1810028F09Rik | Ifi27l2a | Gtf3c1 | Igkv5-37 | Uqcrfs1 |  | Gm26280 | Eif5 | Dnajc5 |
| Gns | Tsc22d1 | Crip1 |  | Qsox1 | Prss3 | Atxn3 | Trip11 | Ighv3-8 | Cyc1 |  | Mir7211 | Selenof | Arf6 |
| Cebpd | Clca4a | Rpl36a |  | Plek | Igkv3-3 | Trim15 | Pum1 | Gzmk | Plekhb2 |  |  | Canx | Flii |
| Col5a2 | Lmo7 | Rps13 |  | Coro1a | Gm15526 | Htatip2 | Arhgef12 | Il22 | Ifit1bl1 |  |  | Capns1 | Ykt6 |
| Adam9 | Sult1d1 | Rps27a |  | Lrig1 | Gm25835 | Tmem128 | Soat1 | Igkv9-123 | Eif2s2 |  |  | Mdh2 | Ddx39b |
| Ppif | Slc9a3r1 | Btf3 |  | Btg2 | Snora47 | Rprd2 | Pja2 | Apoc1 | Nucb1 |  |  | Hnrnpul2 | Alas1 |
| Ltbp4 | Acox1 | Naca |  | Acsl5 |  | Mllt10 | Sytl2 | Scgb3a1 | Anxa4 |  |  | Atp2a2 | Appl2 |
| Wasl | Pls1 | Hint1 |  | Spop |  | R3hdm1 | Klhdc10 | Igkv9-129 | Gmds |  |  | Caprin1 | 9530068E07Rik |
| Coa3 | Hadh | Gne |  | Grk2 |  | Ubl7 | Gm16286 | Ighv1-49 | St14 |  |  | Fkbp4 | Hist1h1c |
| C1qa | Erbb3 | Rpl35a |  | Rab11fip1 |  | H2-DMb1 | Ipo8 | Gm43442 | Cycs |  |  | Rsrp1 | Pak2 |
| Lamc1 | Ctdsp2 | Plec |  | Sod2 |  | Gm10177 | Dedd2 | Ighv2-6-8 | 2610528A11Rik |  |  | Laptm4a | Ogt |
| Col5a1 | Abhd2 | Cox6c |  | Slc2a1 |  | Slc20a2 | Tulp4 | Gm4596 | Adgrg7 |  |  | Hsp90aa1 | Pcbp2 |
| Mmp2 | Sft2d2 | Tmem45b |  | Zfp36 |  | Atf3 | Sirt7 | Ighv3-4 | Uqcrh |  |  | Cd38 | Hp1bp3 |
| Strbp | Phgr1 | Dazap2 |  | Deptor |  | Zfp746 | Pcm1 | Igkv4-69 | Sdhd |  |  | B3gnt3 | Ap2b1 |
| Tmem123 | Lgals3bp | Rps29 |  | Diaph1 |  | Zbtb42 | Gxylt1 | Snord49b | Ppp2ca |  |  | Pcbp1 | Srxn1 |
| Ndufs7 | Stard4 | Rps21 |  | Mtmr6 |  | Tm2d1 | Ptk2 | Ighd1-1 | Ndufs2 |  |  | Clca3b | Mark2 |
| Man2b1 | Cbr1 | Rps12-ps3 |  | Sbno2 |  | Gskip | Dock1 |  | Cenpb |  |  | Ywhab | Ube2z |
| Csnk2a1 | Cnnm4 | Cd9 |  | 7-Sep |  | 2010003K11Rik | Iws1 |  | Atp5j2 |  |  | Surf4 | Hjurp |
| Nek9 | Lin7c | Acly |  | Myadm |  | Tat | Akap11 |  | Cox7b |  |  | Kpnb1 | Hnrnpd |
| Cavin1 | Add1 | Klf5 |  | Trappc10 |  | H2-DMa | Ube4b |  | Sf3b1 |  |  | Hnrnpa1 | Slc39a1 |
| Col6a3 | Prr15l | Gabarap |  | Tnfrsf1b |  | Igkv12-46 | Gde1 |  | Rpn2 |  |  | Rbm3 | Ccnl2 |
| Rnf141 | Vipr1 | B3gnt7 |  | Bdh1 |  | Edrf1 | Vps13c |  | Hmgcs1 |  |  | Ddb1 | Acin1 |
| Impa1 | St3gal4 | Eif3h |  | Riok3 |  | Ighv1-55 | Abcb7 |  | Uqcrc2 |  |  | Ybx3 | Glyr1 |
| Aup1 | Pank3 | Eif3f |  | Tnfrsf1a |  | Tmem209 | Pcif1 |  | Copa |  |  | Ran | Yy1 |
| Pdgfra | Idh3b | Ptbp3 |  | Ostf1 |  | Vars2 | Zc3h7b |  | Cystm1 |  |  | Itga6 | Casp8 |
| Nek7 | Fkbp5 | Sema4g |  | Cdc42se1 |  | Batf2 | Eloc |  | Perp |  |  | Rhoa | AC149090.1 |
| C1qb | Atp11b | Ptms |  | Il13ra1 |  | Igkv15-103 | Mif4gd |  | Cant1 |  |  | Efhd2 | Tnfaip1 |
| Twsg1 | Vdac3 | Ddost |  | Tgm2 |  | Ighv1-76 | Sirt2 |  | Fuca1 |  |  | Malat1 | Wfdc2 |
| Ndufab1 | Zbtb7a | Sdc1 |  | Slc5a1 |  | Prr12 | 8-Sep |  | Cmas |  |  | Pdia6 | Ppp6r1 |
| Tbrg4 | Shisa5 | Mgat4a |  | Marcks |  | Nat8f4 | Ypel5 |  | Gna11 |  |  | Wdr1 | Tinagl1 |
| Slc3a2 | Mkrn1 | Plekha6 |  | Mbtps1 |  | Galk1 | N4bp2l2 |  | Uqcrq |  |  | Eif3a | Srsf11 |
| Smim10l1 | Dsc2 | Rpl38 |  | Fut8 |  | Jkamp | Rasa1 |  | Rnf10 |  |  | Hnrnpa0 | Alkbh5 |
| Vat1 | Ptprf | Atp5h |  | Il4ra |  | Usp42 | Fam210a |  | Cox6b1 |  |  | Vcp | Slc38a10 |
| Chchd10 | Irf7 | Sod1 |  | Nfkbiz |  | Prkdc | Zfp384 |  | Tmed10 |  |  | Ppp1ca | Rbbp4 |
| Slc25a4 | Vdr | Cox7a2l |  | Bhlhe40 |  | Igkv1-99 | Sec24a |  | Net1 |  |  | Slc6a8 | Matr3 |
| Tnfrsf11a | Ipmk | Gsdmc4 |  | Grina |  | Fut11 | Plbd1 |  | Pdcd6ip |  |  | S100a14 | Tbc1d20 |
| C1s1 | Scp2 | Rps10-ps2 |  | Ehd1 |  | Akt3 | Usp6nl |  | Gng12 |  |  | Csde1 | Slc35e1 |
| Spin1 | Entpd5 | Id1 |  | Samhd1 |  | Cenpc1 | Nfix |  | Cox5b |  |  | H2afz | Nacc1 |
| Bach1 | Hif1a | Rpl17-ps3 |  | Cd44 |  | Igkv3-10 | Gopc |  | Mal2 |  |  | Srsf1 | Wls |
| Cpped1 | Cpt1a | BC005537 |  | Ifitm1 |  | Bid | Usf1 |  | Coro2a |  |  | Prrc2a | Cggbp1 |
| C1qc | Fkbp1a | Gm9794 |  | Dusp1 |  | Ubd | Ltn1 |  | Mgat4b |  |  | Slc35c1 | Dbnl |
| Unc93b1 | Suclg2 | Npc2 |  | Prdx3 |  | Gm6206 | Kif21a |  | Myo1d |  |  | Odc1 | Ralbp1 |
| Nid1 | Ildr1 | Jpt1 |  | Cotl1 |  | Abhd8 | Dlgap4 |  | C77080 |  |  | Son | Clk1 |
| Uhrf2 | Aldh1b1 | Bag1 |  | Iars2 |  | Gzmb | Dmxl1 |  | Cttn |  |  | Gm10123 | Ythdf3 |
| Zfr | Slc9a2 | Anxa7 |  | Fbxl5 |  | Ighv1-80 | Cnot8 |  | Rab2a |  |  | Prr13 | Fam102b |
| Klhl9 | Hsd11b2 | Rpl21 |  | Xdh |  | Igkv13-85 | Ehmt1 |  | Gale |  |  | Srsf3 | Trpc4ap |
| Klf13 | Mep1a | Rpl7a |  | Ube2h |  | Qtrt2 | Scaf8 |  | Suclg1 |  |  | Srebf2 | Ndfip1 |
| Crispld2 | Atp2b1 | Rps28 |  | Cd47 |  | Igkv4-57 | Arhgap17 |  | Lad1 |  |  | Hnrnpf | Ccdc71l |
| Hook2 | Tor1aip2 | Atp5e |  | Syk |  | Gbp4 | Bax |  | Fkbp8 |  |  | Ccl6 | Luc7l3 |
| Rammet | Dynll2 | Ndufa6 |  | Tnks1bp1 |  | Gm3788 | Eif2ak3 |  | Ifngr1 |  |  | Ak2 | Eif1a |
| Cic | Fat1 | Eif3e |  | Lmnb1 |  | Ido1 | Ascc3 |  | Ppp2r1a |  |  | Hnrnpl | Rnf4 |
| Zfp598 | Nupr1 | Prkar1a |  | Tnfaip3 |  | L3mbtl3 | Ago1 |  | Rtn3 |  |  | Lman2 | Foxp1 |
| Ctnnd1 | Sectm1b | Cst3 |  | Marcksl1 |  | Mppe1 | Sgpp1 |  | Clint1 |  |  | Lrrc59 | Zc3h7a |
| Sugt1 | Pdlim1 | M6pr |  | Aldob |  | Als2 | Rgl2 |  | Idh1 |  |  | Ptbp1 | Fbxl3 |
| Ndst1 | Map2k1 | Azin1 |  | Apob |  | Tbck | Zcrb1 |  | Tjp3 |  |  | Set | Usp4 |
| Fkbp9 | F11r | Nfe2l2 |  | Midn |  | Usp2 | Rptor |  | Mknk2 |  |  | Rps6ka1 | Sbds |
| Capn7 | Retnlb | Oxct1 |  | Hnrnph2 |  | Aptx | Strn3 |  | Fut2 |  |  | Mki67 | Rbm25 |
| Plekha7 | Etf1 | Psmb1 |  | Arhgef1 |  | Il18bp | Golga1 |  | Dbi |  |  | Hdgf | Nxf1 |
| Ereg | Trim25 | Cct4 |  | Jun |  | Cxcl9 | Ptprg |  | Uqcr10 |  |  | Ppa1 | Stk11 |
| Cisd2 | Mfsd6 | Stat3 |  | Tpcn1 |  | Sprr1a | Ids |  | Lrp10 |  |  | Nono | Sipa1l1 |
| Fcgr2b | Slc5a8 | Hoxb13 |  | Lamc2 |  | Gm9791 | Stxbp5 |  | Epb41l4b |  |  | Dync1h1 | Clock |
| Itgav | Adipor2 | Gng5 |  | Furin |  | Cbll1 | Yeats4 |  | Chmp1a |  |  | Klf6 | Gm26917 |
| Itga5 | Galnt4 | Csnk1g2 |  | Nrd1 |  | Mlh3 | Sdhaf2 |  | Copb2 |  |  | Cct2 | Itm2c |
| Dgcr2 | Golga4 | Scarb2 |  | Atp6v1e1 |  | Aif1 | Zfp146 |  | Arl6ip1 |  |  | Hnrnpc | Ehd4 |
| Oxa1l | Dpep1 | Csrp2 |  | Lrba |  | Igkv14-100 | Spast |  | AA467197 |  |  | Hmgb2 | Styk1 |
| Fndc3b | Dnm2 | Trip12 |  | Ptprc |  | Emid1 | Pex5 |  | Jup |  |  | Ctbp1 | Atp6v1b2 |
| Bmpr2 | Far1 | Idh3a |  | Zyx |  | Igkv3-5 | Dcaf1 |  | Hadha |  |  | Arpc3 | Lman1 |
| Emilin1 | Pttg1ip | Nop53 |  | Npepps |  | Socs1 | Gemin7 |  | Stt3b |  |  | Hnrnpm | Rbm5 |
| Csf1r | Clstn1 | Psma7 |  | Jak2 |  | Mcpt1 | Mrpl33 |  | Sptlc2 |  |  | Sfpq | Shc1 |
| Dusp6 | Parm1 | Reg4 |  | Atp6v1a |  | Igkv12-41 | Ufl1 |  | Gucy2c |  |  | Skp1a | Man1b1 |
| B4galt6 | Rnf213 | Gm6136 |  | Preb |  | Igkv9-124 | Paip2b |  | Stk25 |  |  | H3f3a | Retreg1 |
| Esyt1 | Galnt7 | Rock2 |  | Ptpn1 |  | Cd300c2 | Tap2 |  | Atp5j |  |  | Cct5 | Cdc42bpb |
| Rapgef1 | Golgb1 | Ubr5 |  | Pten |  | Gm14137 | Helz |  | Sdhb |  |  | Tuba1b | Ppp3r1 |
| Fbln1 | Slc35a1 | Elob |  | Ephb4 |  | Gm9844 | Ddx19b |  | Mapk13 |  |  | Dynll1 | Stk24 |
| Axl | Lgals9 | Eif3i |  | Ap1b1 |  | Tgtp2 | Tbc1d8b |  | Nckap1 |  |  | Hnrnph1 | Leng8 |
| Gja1 | Trp53inp2 | Ggh |  | Lsm14a |  | Ighv1-42 | Supt4a |  | Cox7a2 |  |  | Rab5c | Pdcd4 |
| Dlg3 | Nlrp6 | Trp53i11 |  | Arap1 |  | Cd3g | Fmn1 |  | Fasn |  |  | Pitpna | Gmfb |
| Ptpn3 | Trim2 | Eif3l |  | Zfand5 |  | Igkv6-14 | Surf6 |  | Epn1 |  |  | Mrfap1 | Zc3hav1 |
| Plpp3 | Hpgd | Tgm3 |  | Mfsd14b |  | Ppfia4 | Setd1b |  | Ak3 |  |  | Eif6 | Dcaf7 |
| Sephs2 | Atp13a3 | Arf5 |  | Tut7 |  | Xylb | Fto |  | Sdf4 |  |  | Ssr1 | Vsig10 |
| Pecam1 | Prkcd | Amfr |  | Stk17b |  | Mcpt2 | Vamp4 |  | Sh3bgrl2 |  |  | Esd | Morf4l1 |
| Mmp3 | Myo18a | Rab25 |  | Stat6 |  | Slc7a9 | Sdccag3 |  | Atp5l |  |  | Psmd2 | Dnajb6 |
| Ncor2 | Acads | Ehmt2 |  | Etl4 |  | Gchfr | Wbp4 |  | Cmtm4 |  |  | Pde9a | Rab21 |
| Rasa3 | Slc35g1 | 2900097C17Rik |  | Cflar |  | Tgtp1 | Ints4 |  | Sdhc |  |  | Klf3 | Hist1h2bc |
| Synj2bp | Clcn2 | Tnpo1 |  | Nfkb1 |  | Tlr12 | Glce |  | Syngr2 |  |  | Morf4l2 | Rab11b |
| Anxa6 | B4galnt2 | Pum2 |  | Clcn3 |  | Igkv3-1 | Cnep1r1 |  | Eps8 |  |  | Ywhaq | Slc20a1 |
| Stab1 | Abcd3 | Gm10275 |  | Sbf1 |  | Ccr10 | Dlg5 |  | Bspry |  |  | Nars | Zfand6 |
| Rabgap1l | Cdx2 | Wasf2 |  | Pim3 |  | Nkg7 | 6030458C11Rik |  | Spint1 |  |  | Rad21 | Ppp3ca |
| Itga1 | Nov | Jak1 |  | Wbp1l |  | Wnt5b | Vma21 |  | Pdha1 |  |  | Hspa4 | Cers2 |
| Rbm6 | Mtch2 | Itih5 |  | Plvap |  | Ccl20 | Evi5 |  | Cox5a |  |  | Fus | mt-Co3 |
| Fbxw2 | Clmn | Lgr4 |  | Ifitm2 |  | Slamf9 | Cog7 |  | Usmg5 |  |  | G3bp1 | Acbd3 |
| Poldip2 | Actr1b | Rps27l |  | Tra2a |  | Cxcr6 | Cuedc2 |  | Tm4sf20 |  |  | Purb | Cdc42se2 |
| Plpp5 | Galnt10 | Serinc1 |  | Zfand3 |  | Pipox | Nbas |  | Sdc4 |  |  | Tardbp | Akirin1 |
| Il1r1 | Ifi27l2b | Ppp2r5c |  | Col4a3bp |  | Slamf8 | Prelp |  | Tmed7 |  |  | Sec61a1 | Chtf8 |
| Tnxb | Apol10a | Plp2 |  | Cndp2 |  | Apol10b | Dnajb14 |  | Uqcrb |  |  | Papola | Agpat2 |
| Ighv1-53 | Sema4a | Gpx4 |  | Akr1b8 |  | Ighv1-84 | Usf2 |  | Atp5g1 |  |  | Ccnd2 | Senp6 |
| Tgfbr1 | Higd1a | Rpl14-ps1 |  | Mical2 |  | Ighv5-12 | Mtmr1 |  | Esrra |  |  | Wars | Ckap4 |
| Fzd1 | Ech1 | Hipk1 |  | Msl1 |  | Calhm6 | Rad54l2 |  | Mlf2 |  |  | Phb2 | Gosr2 |
| Igkv14-111 | Tapbp | Fam168b |  | Ehbp1l1 |  | Vps37d | Nacc2 |  | Ugp2 |  |  | Aplp2 | Naip6 |
| Slc39a14 | Afdn | Gipc2 |  | Map3k11 |  | Tmem273 | Rnf166 |  | Copb1 |  |  | Dnaja2 | mt-Tp |
| Eln | Hao2 | Gna13 |  | Sgsm3 |  | Scimp | Slc25a28 |  | Sdcbp2 |  |  | Eif3b | Tmx1 |
| Sod3 | Prss30 | Akap13 |  | Rnf19b |  | Gm18584 | Zbtb33 |  | Cyp51 |  |  | Hmgcr | Mdm4 |
| Synpo | Ssfa2 | Hectd1 |  | Sidt2 |  | Gm4482 | Gps2 |  | Atp10b |  |  | Cct7 | Stx12 |
| Ccl9 | Abcc3 | Tnks2 |  | Atp1b3 |  | Ak3l2-ps | Zfp609 |  | Tmbim1 |  |  | Psmb4 | Prpf38b |
| Spred1 | Esyt2 | Kars |  | Fos |  | Ighv11-2 | Pdpr |  | Prdx2 |  |  | Slc6a6 | Efr3a |
| Fbn1 | Golph3 | Larp4b |  | Tnip1 |  | Gm12891 | Atf7 |  | Ndufv2 |  |  | Sfn | Ubap2l |
| Tmem119 | Ablim1 | Eif3k |  | Kpna4 |  | Trbv13-2 | Katna1 |  | Dlst |  |  | Srsf2 | Pck1 |
| Dnajc22 | Chmp2b | Gsdmd |  | Stk40 |  | Trbv16 | Ercc6l2 |  | Plpp2 |  |  | Tmed9 | Irf2bp2 |
| Nbl1 | Papss1 | Lnpep |  | Hibadh |  | Mir7030 | Blvrb |  | Slc38a2 |  |  | Ctsz | Hid1 |
| Loxl2 | Gcnt3 | Gsdmc2 |  | Osbpl9 |  | Trbd1 | Ttc37 |  | Macf1 |  |  | Ewsr1 | Zfp445 |
| Mrps35 | Myo5b | Limk2 |  | Sbno1 |  | Gm25432 | Abtb1 |  | Dld |  |  | Tspo | Ddah1 |
| Extl3 | Fam234a | Ranbp2 |  | Plin2 |  |  | Qser1 |  | Msmo1 |  |  | Anp32b | Pkp4 |
| Wdfy3 | Ndfip2 | Rpl21-ps15 |  | Birc3 |  |  | Epg5 |  | Rnf128 |  |  | Mapk1 | Fbxw11 |
| Pea15a | Cluh | Susd6 |  | Otud5 |  |  | 1810058I24Rik |  | Mtch1 |  |  | Srsf6 | Add3 |
| Timp3 | Impad1 | Mgst1 |  | Bcl10 |  |  | Rmc1 |  | Acadl |  |  | Cct8 | Dcp2 |
| Scpep1 | Myo7b | Smdt1 |  | Wfdc17 |  |  | Smarcad1 |  | Ssr4 |  |  | Sf3b2 | Tm9sf1 |
| Il1rl1 | 1700020I14Rik | Pnrc2 |  | Ecm1 |  |  | Naaa |  | Cds1 |  |  | Ssr2 | Chpt1 |
| Lbh | Slc39a9 | Sval1 |  | Psd4 |  |  | Hsdl1 |  | Cib1 |  |  | Stt3a | Scaf1 |
| Mrc1 | Ppp6r3 | Scamp1 |  | Hdc |  |  | Pmaip1 |  | Zbtb7b |  |  | Thbs4 | Aldh9a1 |
| Heg1 | Fam129b | Dctn2 |  | Mapk14 |  |  | Prkag1 |  | Cdkn1a |  |  | Prkar2a | Mfsd1 |
| Rdh10 | Tmc4 | Abat |  | Akt2 |  |  | Rmnd5b |  | Immt |  |  | Snx5 | Nrbp1 |
| Vcam1 | Ces2e | Tmem234 |  | Laptm5 |  |  | Ctnnal1 |  | Edf1 |  |  | Asah1 | Ccnd1 |
| Spon1 | Clic5 | Smim24 |  | Kif13a |  |  | Slc45a4 |  | Naip1 |  |  | Krt7 | Ttc7 |
| Vcan | Rmnd5a | Kcne3 |  | Rnf149 |  |  | Thsd4 |  | Gak |  |  | Dst | Relch |
| Nfic | Serinc2 | Nsa2 |  | Tsc22d3 |  |  | Pggt1b |  | Uap1 |  |  | Metap2 | Nomo1 |
| Mmp10 | Arhgap5 | Smim6 |  | Msrb1 |  |  | Mars2 |  | Chmp3 |  |  | Tes | Rab35 |
| Ehd2 | Ndrg1 | Xiap |  | Nos2 |  |  | Slc25a32 |  | Atp5g2 |  |  | Tcp1 | Ginm1 |
| Ighv1-64 | Akr1c19 | Aff4 |  | Pnrc1 |  |  | Dpy19l4 |  | Tmem183a |  |  | Kpna2 | Utp20 |
| Ano6 | Abr | H2afj |  | Mrs2 |  |  | C2cd3 |  | Akr1c13 |  |  | Pgam1 | Taok3 |
| C1ra | Aldh1a1 | Dnajc7 |  | Dgat2 |  |  | Fam174a |  | Rab10 |  |  | Ubr4 | Mgat2 |
| Ppt1 | Os9 | Sgpl1 |  | Dhcr7 |  |  | Lig3 |  | Cd82 |  |  | Prpf8 | Ankrd10 |
| Gaa | Dram2 | Map4 |  | Dynlt3 |  |  | Socs7 |  | Atp9a |  |  | Sec61b | Kcnk6 |
| Lama5 | 4931406C07Rik | Fam129a |  | Slfn2 |  |  | Atm |  | Atp5mpl |  |  | Smg1 | Plekha5 |
| Snx27 | Chgb | Polr1d |  | Kdm7a |  |  | Heatr5b |  | Rab14 |  |  | Rnh1 | Pnisr |
| Arnt | Hdac1 | Minos1 |  | Apoa1 |  |  | Manea |  | Slc39a4 |  |  | Ppp1cc | Atxn7l3 |
| Wipf1 | Rnase4 | Baz2a |  | Arfip2 |  |  | Wdr6 |  | Ndufa4 |  |  | Dhx9 | Dcaf12 |
| Eng | Trpm4 | Glg1 |  | Cux1 |  |  | Ctdspl2 |  | Atp5o |  |  | Ipo7 | Ube2j1 |
| Tuba1a | Mier1 | Myo5c |  | Lrg1 |  |  | Wrn |  | 4833439L19Rik |  |  | Cltb | Ap2a2 |
| Gm3336 | Samd9l | Psmb6 |  | Lbr |  |  | Fktn |  | Qars |  |  | Rbbp7 | Usp25 |
| Ppfibp1 | Pon2 | Rtraf |  | Smox |  |  | Zbtb17 |  | Scamp2 |  |  | Pam | Gpat4 |
| Acsl4 | Cxadr | Gm6472 |  | Cebpb |  |  | Pik3r4 |  | Rab1b |  |  | Tmpo | Tnrc6a |
| Dhrs9 | Nbr1 | Aoc1 |  | Arih2 |  |  | Nsrp1 |  | Rer1 |  |  | Rgs5 | Ppp4r3a |
| Ighv1-26 | Zdhhc5 | Ap2s1 |  | Brd4 |  |  | Sox4 |  | Chchd3 |  |  | Mbnl2 | Arglu1 |
| Mafb | Fam20b | Rnf145 |  | Skil |  |  | Rabggta |  | Itpr3 |  |  | Ahcyl1 | Nxpe2 |
| Baz1a | Lurap1l | Swi5 |  | Slc27a4 |  |  | Gon4l |  | Dhcr24 |  |  | Cdv3 | 2010111I01Rik |
| Rbfox2 | Smim14 | Sem1 |  | Gdi1 |  |  | Bcl7c |  | Casp7 |  |  | Dnajc3 | Supt5 |
| Pofut2 | Arfgap3 | Atp6v0b |  | Nsd3 |  |  | Rgs10 |  | Rps27rt |  |  | Dnaja1 | Klc1 |
| Col15a1 | Tspan13 | Pyy |  | Neo1 |  |  | Alg11 |  | Etfa |  |  | Tmem30a | Kdm3b |
| Hspb1 | Acsl3 | Eif3m |  | Pcnx3 |  |  | Tfeb |  | Ldlr |  |  | Rad23b | Sike1 |
| 2310061I04Rik | Usp47 | Uso1 |  | Cog3 |  |  | AI597479 |  | Itpk1 |  |  | Ap1s1 | Cisd3 |
| Sh3pxd2a | Tst | Stag2 |  | Slc16a3 |  |  | Wtip |  | Shroom3 |  |  | Sec31a | Marf1 |
| Rbpj | Atp6v0a2 | Ociad1 |  | Pfkfb3 |  |  | Fbxl17 |  | Pfkl |  |  | Zfp106 | Vps26a |
| Tead1 | Ube2i | Tma7 |  | Cnn2 |  |  | Ptpn4 |  | Prelid3b |  |  | Ostc | Anxa11 |
| Dkk3 | Slc4a2 | Rps16-ps2 |  | Stk38l |  |  | Tesk1 |  | Afg3l2 |  |  | Smpd3 | Kctd10 |
| Gata6 | Slc25a20 | Arpc1a |  | Mfn1 |  |  | Tbc1d5 |  | Rnf44 |  |  | Ly6g | Rsrc2 |
| Ubac2 | Mtus1 | Birc6 |  | Pmepa1 |  |  | Rnf25 |  | Clptm1 |  |  | Map2k2 | Rnf14 |
| Cfh | Neu1 | Ppp5c |  | Lyn |  |  | Usp21 |  | Ifngr2 |  |  | Tuba1c | Akap8 |
| Napg | Fam162a | Ndrg2 |  | Cdc42ep4 |  |  | Gm3054 |  | Vps35 |  |  | Usp7 | Fcho2 |
| Mcam | Chmp4c | Pfdn5 |  | Akap1 |  |  | Trove2 |  | Rhoc |  |  | Oat | Gm5485 |
| Egfr | Pacsin2 | Srp9 |  | Atp6v0a1 |  |  | Bace1 |  | Ndufa10 |  |  | Adam10 | Nploc4 |
| Pls3 | Echs1 | Pycard |  | Ppp1r12c |  |  | Tbc1d4 |  | Slc25a39 |  |  | Rab6a | Pnn |
| Ap1g2 | Hsd17b11 | Tmem259 |  | Cyth1 |  |  | Sorbs1 |  | Aph1a |  |  | Txn2 | Phldb1 |
| Spon2 | Slc35f5 | Tnrc18 |  | Sgms2 |  |  | Rps6kc1 |  | Rab18 |  |  | Cct3 | mt-Co2 |
| Hgsnat | Pof1b | Arih1 |  | Ubp1 |  |  | Ammecr1 |  | Twf1 |  |  | Cast | Ddx42 |
| Ext1 | Tmcc3 | Nt5c |  | N4bp1 |  |  | Npat |  | Slc31a1 |  |  | Cdc37 | Oxsr1 |
| Rcan1 | Slc4a7 | Dgka |  | Rac2 |  |  | Gpam |  | Setd3 |  |  | Ankrd11 | Foxp4 |
| Fbln2 | Max | Tab2 |  | Ccnl1 |  |  | Cpne8 |  | Sult1b1 |  |  | Strap | Colgalt1 |
| Sh3pxd2b | Erbb2 | Ptpn11 |  | Tle3 |  |  | Smim13 |  | Mbnl1 |  |  | Thrap3 | Pla2g4a |
| Acadsb | 5330417C22Rik | St3gal6 |  | Tsnax |  |  | Lcor |  | Llgl2 |  |  | Larp1 | Khdc4 |
| Thbd | Btnl5-ps | Uhmk1 |  | Prrc1 |  |  | Arhgef26 |  | Sgpp2 |  |  | Capza1 | Shoc2 |
| Slc48a1 | Mpst | Brk1 |  | Map2k4 |  |  | Zfp507 |  | Ndufv1 |  |  | Cct6a | Ccdc9 |
| Abcc9 | Mettl7a1 | Ago2 |  | Ndel1 |  |  | Wee1 |  | Psen1 |  |  | Anxa3 | Ppp4r2 |
| Odr4 | Cgn | Spink1 |  | Pim1 |  |  | Rnf215 |  | Rtcb |  |  | Hnrnpul1 | Ddx50 |
| Stam | Ywhag | Drap1 |  | Flot1 |  |  | Borcs6 |  | Slc37a1 |  |  | Sf1 | Ppp4r1 |
| Svep1 | Parva | Tpt1-ps3 |  | Cxcl5 |  |  | Acbd4 |  | Tm9sf4 |  |  | Mtdh | Mbd1 |
| Aktip | Vps13d | Ubxn1 |  | Bcl3 |  |  | Dapp1 |  | Polr2m |  |  | Top2a | Zkscan3 |
| Tgfbr3 | Tent5a | Stmn1 |  | Rnf40 |  |  | Dolk |  | Dgkz |  |  | Zfp207 | Cat |
| Ptgs2 | Akap9 | Lsm4 |  | Trib1 |  |  | Lynx1 |  | Sec11c |  |  | Gars | Gpd2 |
| Pias4 | Pi4k2b | Ube2r2 |  | Hexim1 |  |  | Mfap4 |  | Tmprss4 |  |  | Psmc3 | Hnrnpa3 |
| Kcnj8 | Ociad2 | Rps2-ps13 |  | Spata13 |  |  | Igsf9b |  | Hexa |  |  | Dhx15 | Nr1d2 |
| Akr7a5 | Ppp1r2 | 6-Mar |  | Mmp8 |  |  | Dip2c |  | Uqcr11 |  |  | Itgb4 | Wipi2 |
| Traf3 | Cyp3a13 | Hsbp1 |  | Oaz2 |  |  | Nr1h3 |  | Akr1c12 |  |  | Gstp1 | Bfar |
| Tmem2 | Acadm | Etnk1 |  | Antxr2 |  |  | Faim |  | Letm1 |  |  | Smad4 | Cbfb |
| Lama4 | Ppm1a | Srebf1 |  | Fbxo3 |  |  | Zbtb24 |  | Kdelr1 |  |  | H13 | Zranb2 |
| Pld3 | Dab2ip | Dnm1l |  | Snap23 |  |  | Epha4 |  | Cd151 |  |  | Pcna | 6430548M08Rik |
| Cp | Pccb | Cul3 |  | Gk |  |  | Hlcs |  | Slc25a10 |  |  | Pa2g4 | Eloa |
| F2r | Hadhb | Snx2 |  | Arhgef2 |  |  | Btd |  | Mall |  |  | Dnajb11 | Thap12 |
| Mtif2 | Edem3 | Fut4 |  | Tcn2 |  |  | B3glct |  | Clrn3 |  |  | Psma1 | Tcea1 |
| Tgfb1 | Setd5 | Atxn2l |  | Entpd1 |  |  | Pomt2 |  | Bcap31 |  |  | Api5 | Fam135a |
| Grhl2 | Ccng2 | Mrpl51 |  | Map4k4 |  |  | Nudt18 |  | Tspan15 |  |  | Tra2b | Baiap2l2 |
| Bcr | Baiap2l1 | Pde5a |  | Fcgr3 |  |  | Gpank1 |  | Micu1 |  |  | Ubqln1 | Icmt |
| Tacc1 | Zzef1 | Nedd8 |  | C530008M17Rik |  |  | Sfxn5 |  | Rab15 |  |  | Hsd17b12 | Rgmb |
| Hpse | Rflnb | Sp1 |  | Mllt6 |  |  | Fam213b |  | Ndufb11 |  |  | Cope | Tnpo2 |
| Atp13a2 | Sgk1 | Cryl1 |  | Ell2 |  |  | Tmco6 |  | Man1a2 |  |  | Rab11a | Wdr13 |
| Pkd2 | Oasl2 | Cds2 |  | Ncf2 |  |  | Ulk3 |  | Ppard |  |  | Rcc2 | Gnaq |
| Pros1 | Chmp1b | Ufc1 |  | Csf3r |  |  | AI413582 |  | Tmem54 |  |  | Manf | Ddx54 |
| Cenpv | Specc1l | Ddi2 |  | Clec4e |  |  | Lrrc8a |  | R3hdm4 |  |  | Sec24c | Pcf11 |
| Fdx1 | Tns4 | Ndufa8 |  | Plagl2 |  |  | Plcxd2 |  | Paqr4 |  |  | Clec2d | Zmynd8 |
| Asap1 | Luzp1 | Rnf186 |  | Plaur |  |  | Uprt |  | Me2 |  |  | Psma2 | Agpat3 |
| Egr1 | Gtpbp2 | Ndufa2 |  | Tbc1d10b |  |  | Sik2 |  | Tmem8 |  |  | Pdia4 | Sowahc |
| Tubb6 | Yipf6 | Ralgds |  | Ier5 |  |  | Fbxw4 |  | Jund |  |  | Copz1 | Rnf6 |
| Mzb1 | Leprot | 2-Mar |  | Itgb2 |  |  | Pkn3 |  | Ndufs1 |  |  | Ctdsp1 | Taok2 |
| Scarb1 | Cyp4f14 | S100a1 |  | Ccnd3 |  |  | Tasp1 |  | Sqle |  |  | Spcs2 | Nsf |
| Mxra8 | Azi2 | Sumo1 |  | Nfkb2 |  |  | Nectin1 |  | Fdft1 |  |  | Mta2 | Usp48 |
| Slc43a3 | Rbm47 | Ifi30 |  | Fam49b |  |  | Rprd1a |  | Klc4 |  |  | Capg | Nfx1 |
| Iglv2 | Fryl | Mindy1 |  | Rhog |  |  | Lrch3 |  | Luc7l2 |  |  | Naa50 | Rragc |
| Piezo1 | Irf6 | Fis1 |  | Magi3 |  |  | Zscan25 |  | Psme4 |  |  | Vapa | Tial1 |
| Marveld2 | Ces2c | Aamp |  | Lsp1 |  |  | Abcg5 |  | Fh1 |  |  | Vamp3 | Cacul1 |
| Acp2 | Ap3d1 | Ndufa13 |  | Chd7 |  |  | H3f3a-ps1 |  | Casp1 |  |  | Anapc5 | Nr1i2 |
| Ddr2 | Pcsk7 | Fam8a1 |  | Asprv1 |  |  | Gm45884 |  | Capn5 |  |  | Numa1 | Chd8 |
| Adamts1 | Stard7 | Sra1 |  | Atp11a |  |  | Lrrc49 |  | Cdk16 |  |  | Psma6 | Itgb5 |
| Zfp330 | Phyh | Kdm2a |  | Tnfaip2 |  |  | 1810043G02Rik |  | Smagp |  |  | Cd81 | Tbl1xr1 |
| Antxr1 | Satb2 | Map7 |  | Cdk11b |  |  | Cklf |  | Saraf |  |  | Eef1d | Smad5 |
| Zcchc24 | Heph | Cops2 |  | Mcf2l |  |  | Adcy9 |  | Ergic3 |  |  | Prnp | Crebzf |
| Washc4 | Dyrk2 | Ndufb7 |  | Cd53 |  |  | Med31 |  | Slc40a1 |  |  | Hspe1 | Zfc3h1 |
| Myc | Fndc3a | Ncaph2 |  | Csf2rb |  |  | Lsm10 |  | Ei24 |  |  | Ubtf | Peli1 |
| Rhot2 | P2rx4 | Tns1 |  | Rbms1 |  |  | 2700049A03Rik |  | Ncstn |  |  | Gorasp2 | Kpna1 |
| Kremen1 | Helz2 | Mrpl52 |  | Sun2 |  |  | Cntnap1 |  | Bak1 |  |  | Psma4 | Kat7 |
| Ap3s2 | Ush1c | Ubald2 |  | Dgat1 |  |  | Zfp526 |  | Dhx32 |  |  | Fam107b | Eif2s3y |
| Tbccd1 | Tmem127 | Atl3 |  | U2surp |  |  | Tlcd2 |  | Degs2 |  |  | Ipo5 | Ccdc88c |
| Fermt2 | Coa5 | Cop1 |  | Igfbp3 |  |  | Unc5a |  | Bag6 |  |  | Itga3 | Pkn1 |
| Trim35 | 2900026A02Rik | Serf2 |  | Xpo6 |  |  | Kctd13 |  | Yme1l1 |  |  | B4galt1 | Slc25a36 |
| Ngef | Sh3bgrl3 | Ppp4r3b |  | Sec14l1 |  |  | Ceacam18 |  | Rhpn2 |  |  | U2af2 | Nudcd3 |
| Rras | Mgst2 | Igf2r |  | 1810013L24Rik |  |  | Vipr2 |  | Lamb3 |  |  | Psmc1 | Rbm33 |
| Ccl11 | Tob1 | Ndufa5 |  | Myo9b |  |  | Zscan22 |  | Ick |  |  | Nptn | Firre |
| Itpripl2 | Tep1 | Acsm3 |  | Ikbkb |  |  | Mmp17 |  | Mia2 |  |  | Gm6793 | Lrig3 |
| Foxf1 | Fam102a | Hipk3 |  | Samd4b |  |  | 1500011B03Rik |  | Anks4b |  |  | Tmem131 | Ap2a1 |
| Map3k4 | Gpd1l | Spag9 |  | Acaca |  |  | Stk26 |  | Arhgef16 |  |  | Tuba4a | Fam160a2 |
| Pdgfrb | Arhgap21 | Ndufa7 |  | B4galt5 |  |  | Phldb3 |  | Tsta3 |  |  | Syncrip | Cntrl |
| Lox | Adk | Cyb5a |  | Pip5k1c |  |  | Cfb |  | Fam32a |  |  | Ptpa | Coq2 |
| Sdc3 | Cyp2c65 | Cbl |  | Cd52 |  |  | Zfp691 |  | Ppp1r14d |  |  | Sparcl1 | Tef |
| Plau | Rere | Rbck1 |  | Tox4 |  |  | Gm15675 |  | Ttc39a |  |  | Hmgn1 | Naip5 |
| Ccdc91 | Camk2n1 | Sptssa |  | Ifnar1 |  |  | Pard6a |  | Tmem14c |  |  | Cep85 | Kif3b |
| Plk2 | Prkab1 | Nipbl |  | F3 |  |  | Abcg8 |  | Idh3g |  |  | Psmd3 | Angel2 |
| Man2a2 | Dync1li2 | Arid1a |  | Bcas1 |  |  | Fbxo44 |  | Tmem63a |  |  | Galnt1 | Gstm2 |
| Usp43 | Sord | Arpp19 |  | Frmd4b |  |  | Nectin4 |  | Smim7 |  |  | Szrd1 | Qdpr |
| Hdac4 | Cftr | Ppp1r9b |  | Adgre5 |  |  | Gm1818 |  | Fam3b |  |  | Snd1 | Gm53 |
| Epn2 | Pgrmc1 | Pef1 |  | Adam17 |  |  | Ptar1 |  | Ppib |  |  | Samm50 | Ogn |
| Clmp | Cept1 | Nt5c3 |  | Pgs1 |  |  | Acyp2 |  | Cyb561 |  |  | Copg1 | Mapk9 |
| Igkv6-17 | Ubc | Eif4e2 |  | Fam174b |  |  | Arv1 |  | 2510039O18Rik |  |  | BC030870 | Mex3c |
| Pxdn | Tceal9 | Dock5 |  | Fbxo11 |  |  | Gm9938 |  | Ap1m2 |  |  | Dusp11 | Ccnt2 |
| Sntb2 | Acsf2 | Prrc2b |  | Clip1 |  |  | Gm6877 |  | Dpp3 |  |  | Psmd8 | Atf6b |
| Inf2 | Prkaa2 | Itsn2 |  | Kdm6b |  |  | Gm10734 |  | Ndufa9 |  |  | Ppp2cb | 11-Sep |
| Qk | Cldn15 | Dynlrb1 |  | Gpcpd1 |  |  | Fam89a |  | Txndc17 |  |  | Arpc4 | Ascc2 |
| F13a1 | Arhgef5 | Taok1 |  | Cldn4 |  |  | Gm12657 |  | Zdhhc13 |  |  | Chtop | Zyg11b |
| Uck1 | Gucd1 | Gabpb2 |  | G0s2 |  |  | Gm5905 |  | Mcu |  |  | Prkacb | Sh2b1 |
| Dab2 | Chp2 | Taf10 |  | Ivd |  |  | Igkv1-133 |  | Ubl5 |  |  | Trim28 | Adck5 |
| Tanc1 | Grk6 | Plgrkt |  | Il10rb |  |  | 4930524J08Rik |  | Erlin2 |  |  | Nap1l1 | Irf2 |
| Col16a1 | Scaf11 | Lpgat1 |  | Esrp2 |  |  | Gm14438 |  | Pdhb |  |  | Pbxip1 | Tatdn2 |
| Ptprb | Lims1 | Ssbp3 |  | Mmp13 |  |  | Gm33051 |  | Suds3 |  |  | Tjp2 | Dvl1 |
| Tns2 | Lrp6 | Acss2 |  | Abca1 |  |  | Gm3219 |  | Ccl28 |  |  | Tpr | Plekha2 |
| Matn2 | Rnf114 | Rpl22l1 |  | Ssh2 |  |  | Rpl15-ps2 |  | Tbrg1 |  |  | Tufm | Brd8 |
| Cbx6 | Capn1 | Herc2 |  | Vsir |  |  | AF357399 |  | Aacs |  |  | Psmb3 | Eaf1 |
| Ighv3-6 | Capn2 | Rab5b |  | Tbc1d2b |  |  | AC151284.2 |  | Ube3b |  |  | Anp32a | Isoc1 |
| Usp37 | Ddr1 | Chd3 |  | Cldn12 |  |  | Gm7931 |  | Naga |  |  | Wapl | Gsdmc3 |
| Igkv4-53 | Mgat3 | Supt6 |  | Dusp16 |  |  | Snord58b |  | Slc35a2 |  |  | Hyou1 | Luc7l |
| Vwf | Fermt1 | Aurkaip1 |  | Fech |  |  | Snora78 |  | Insig1 |  |  | Stom | Taf1d |
| Plxnd1 | Ggnbp2 | Polr2a |  | Bcl2l1 |  |  | Mir8094 |  | Vamp8 |  |  | Snrpb | Gsk3a |
| Tm4sf1 | Krcc1 | Pdlim5 |  | Cxcl3 |  |  | Gm10180 |  | Src |  |  | Ddx21 | Rexo2 |
| Fgfr1 | Usf3 | Dlg1 |  | Tspan14 |  |  |  |  | Il17rc |  |  | Spcs3 | Pheta1 |
| Itpkb | Ptprh | Rpsa-ps10 |  | Senp2 |  |  |  |  | Slc25a1 |  |  | Ccng1 | Scfd1 |
| Ccl8 | Tjp1 | Erp29 |  | Rela |  |  |  |  | Pigt |  |  | Khdrbs1 | Wdr45b |
| Fam53c | Usp12 | Gas5 |  | Tmem43 |  |  |  |  | Tmem164 |  |  | Ltbr | Tecpr1 |
| Ms4a6d | Slc30a4 | Mef2d |  | Ncf1 |  |  |  |  | Bclaf1 |  |  | Tram1 | Ppme1 |
| Lyve1 | Dgkd | B230219D22Rik |  | Wdr11 |  |  |  |  | Cox14 |  |  | Tomm70a | Zranb1 |
| Cyth3 | Reep3 | Aldh1l1 |  | Snx18 |  |  |  |  | Fbp2 |  |  | Ly6c1 | Scyl2 |
| Lhfp | Mettl7b | Ndufb9 |  | Dot1l |  |  |  |  | Dok4 |  |  | Tomm22 | Zfp217 |
| Rassf2 | Mxd4 | Igbp1 |  | Trem1 |  |  |  |  | Atpif1 |  |  | Rrp1 | Hes1 |
| Ccdc80 | Sh3d19 | Ano1 |  | Wdr48 |  |  |  |  | Ndufb8 |  |  | C1qbp | Tspyl1 |
| Rhoj | Atad1 | Wac |  | Zdhhc18 |  |  |  |  | Dctn1 |  |  | Cnot6 | Kdm3a |
| Braf | Parp12 | Use1 |  | Cpne2 |  |  |  |  | Ndufs8 |  |  | AI506816 | Gm10925 |
| Serpina3g | Mia3 | Lzts2 |  | Smap2 |  |  |  |  | Mfn2 |  |  | Derl1 | Paip1 |
| Dpysl3 | Dcaf11 | Otud4 |  | Ncoa3 |  |  |  |  | Chmp5 |  |  | Cdk4 | Dpp9 |
| Mrpl44 | Aldh3a2 | Casd1 |  | Lilr4b |  |  |  |  | Kcnk1 |  |  | Atp6ap1 | Trim24 |
| Lars2 | Galnt12 | Rab5a |  | Abca7 |  |  |  |  | Fads2 |  |  | Pomp | Srf |
| Cd93 | Pik3r1 | B3gnt5 |  | C1qtnf6 |  |  |  |  | Prkcsh |  |  | Hprt | Arhgef7 |
| Pik3r3 | Elf1 | Pgls |  | Sirpa |  |  |  |  | Ndufb5 |  |  | Por | Adam15 |
| Maf | Atl2 | Gm15427 |  | Numb |  |  |  |  | Mlx |  |  | Smarca4 | Pck2 |
| Cxcl16 | Brpf3 | Aff1 |  | Map7d1 |  |  |  |  | Sel1l3 |  |  | Prpf40a | Cdk8 |
| Tgfb1i1 | Retreg3 | Mettl9 |  | Mdm2 |  |  |  |  | Agpat1 |  |  | Gm43305 | Cldnd1 |
| Sec22a | Fblim1 | Rbx1 |  | Slc25a37 |  |  |  |  | Camk2d |  |  | Vapb | Vgll4 |
| Cln6 | Mon2 | 4932438A13Rik |  | Mmp9 |  |  |  |  | Higd2a |  |  | Agps | Nemf |
| Gpr180 | Pgm1 | Plk1 |  | Flot2 |  |  |  |  | Tlnrd1 |  |  | Tomm20 | Nfib |
| Ttpal | Hgfac | Mrpl30 |  | Ptpn6 |  |  |  |  | Tbc1d13 |  |  | Phb | Pcmtd2 |
| Hivep2 | Sec62 | Lamtor2 |  | Kctd20 |  |  |  |  | Arl2bp |  |  | Ube2l3 | Stx16 |
| Bdkrb2 | Fa2h | Ep300 |  | Dpf2 |  |  |  |  | Ap3m1 |  |  | Ppm1g | C130074G19Rik |
| Uvrag | Tprgl | Rpl3-ps1 |  | Rab8b |  |  |  |  | Fuca2 |  |  | Grb2 | Nipa2 |
| Il33 | Arhgap27 | Ski |  | Arhgef3 |  |  |  |  | Sucla2 |  |  | Ssrp1 | Taz |
| Ighv1-18 | Hepacam2 | Coq9 |  | Dhx38 |  |  |  |  | Naip2 |  |  | Uggt1 | Fnbp4 |
| Commd6 | Vps54 | D8Ertd738e |  | Shmt1 |  |  |  |  | Galnt3 |  |  | Nap1l4 | Nup153 |
| Tent5c | Sema3c | Acp1 |  | Nup98 |  |  |  |  | Rala |  |  | Eprs | Fbxo32 |
| Adgrf5 | Cdc42bpg | Ptprj |  | Tbk1 |  |  |  |  | Eps8l3 |  |  | Mgea5 | Dync1li1 |
| Fabp5 | Dcaf8 | Eif2a |  | Zfp36l1 |  |  |  |  | Casp3 |  |  | Gclm | Zbtb18 |
| Ly6c2 | Nab1 | Gsk3b |  | Lama3 |  |  |  |  | Micu2 |  |  | Smarcd2 | Tacc2 |
| Pcolce | Acaa2 | Ep400 |  | Steap4 |  |  |  |  | Ndufs3 |  |  | Aprt | Kdm1a |
| Sh3bp4 | Slc39a11 | Tmem134 |  | Pla2g7 |  |  |  |  | Ndufs4 |  |  | Cyba | Ripk1 |
| Zfp672 | Ermp1 | Errfi1 |  | Resf1 |  |  |  |  | Lta4h |  |  | Srp72 | Fahd1 |
| Bcl9l | Mep1b | Dennd2d |  | Casc3 |  |  |  |  | Stxbp2 |  |  | Vars | 1110038B12Rik |
| Cygb | Sp3 | Atp6v1f |  | Klf2 |  |  |  |  | Slc25a11 |  |  | Glo1 | Ccdc25 |
| Cep164 | Pnpla2 | Sec11a |  | Nr4a1 |  |  |  |  | Rnpepl1 |  |  | Glrx | mt-Atp6 |
| Abl2 | Zdhhc20 | Mapkapk3 |  | Sec24b |  |  |  |  | Pip4k2c |  |  | Pabpc4 | Pts |
| Tusc2 | Dnmbp | Pcyt2 |  | Tmem181a |  |  |  |  | F2rl1 |  |  | Fgfr1op2 | Slc35b3 |
| Pip5k1a | Mgat5 | Gm6863 |  | Relb |  |  |  |  | Dlat |  |  | Pds5a | Klf10 |
| Cdh5 | Gcc2 | Tollip |  | Tcirg1 |  |  |  |  | Bpnt1 |  |  | Snx3 | Washc5 |
| Aebp1 | Mmp15 | Ndufb4 |  | Fcer1g |  |  |  |  | 1810037I17Rik |  |  | Cyfip1 | Sfswap |
| Rbpms | Ppm1b | Atp2c1 |  | Mboat7 |  |  |  |  | Emc10 |  |  | Smarca5 | Atp9b |
| Crybg3 | Parp14 | Atp12a |  | Trim8 |  |  |  |  | 2210016F16Rik |  |  | Nucks1 | Slc9a8 |
| Bicd2 | Wwp2 | Atf6 |  | Phf20l1 |  |  |  |  | Tex261 |  |  | Ptges3 | Rab5if |
| Mrps36 | Ergic1 | Setd2 |  | Tlr2 |  |  |  |  | Rcor1 |  |  | Abce1 | Rapgefl1 |
| Trmt1l | Arhgap32 | Gm15500 |  | Ptpn12 |  |  |  |  | Tmem9b |  |  | Hmga1 | Zfp219 |
| Calcrl | Kitl | Mrpl9 |  | Cbr3 |  |  |  |  | Arl8b |  |  | Hmgb1 | Atg4b |
| Ltbp1 | Kif13b | Pkdcc |  | Ets1 |  |  |  |  | Lypla2 |  |  | Smim15 | Gtpbp1 |
| Rundc1 | H2-Q2 | Mib1 |  | Arhgap45 |  |  |  |  | Tmbim4 |  |  | Erh | Slc35f6 |
| Sema7a | Smarcc2 | Nt5e |  | Atf1 |  |  |  |  | Golga2 |  |  | Acvr1b | Elk4 |
| Cfp | 8-Mar | Nedd9 |  | Atg3 |  |  |  |  | Tecr |  |  | H2afy | 2010300C02Rik |
| Msrb3 | Pkn2 | Herc1 |  | Ikbke |  |  |  |  | Vps28 |  |  | Galnt6 | Fbxl14 |
| Cemip | Muc4 | Kat6a |  | Agpat5 |  |  |  |  | Lpcat3 |  |  | St13 | Mfsd14a |
| Areg | Rnf103 | Tlr1 |  | Entpd6 |  |  |  |  | Spcs1 |  |  | Mcfd2 | Tor1b |
| Serpine2 | Ireb2 | Hes6 |  | Rbp2 |  |  |  |  | Btbd1 |  |  | Ppp1r12a | Desi2 |
| 1700017B05Rik | Ptprk | Tmc6 |  | Mef2a |  |  |  |  | Syap1 |  |  | Cpne3 | Lipa |
| Sema3f | Ppp1r37 | Hacd3 |  | Tyrobp |  |  |  |  | Stim1 |  |  | Map2k3 | Pan3 |
| Slit3 | Tmem236 | Pafah1b3 |  | Ifrd1 |  |  |  |  | Tradd |  |  | Arhgap1 | Zwint |
| Ptk7 | Ralgapb | Cep350 |  | Fabp4 |  |  |  |  | Becn1 |  |  | Ppp4c | Zmym5 |
| Fhl2 | Mast2 | Comt |  | Chst4 |  |  |  |  | Mgrn1 |  |  | Acaa1a | Sorbs2 |
| Creb3l2 | Bmpr1a | Hnrnpll |  | Myd88 |  |  |  |  | Gdpd1 |  |  | Maz | Ezh1 |
| Plod1 | Eif4g3 | Ap3s1 |  | Ppp1r3b |  |  |  |  | Nectin2 |  |  | Zdhhc3 | Snap29 |
| Kdf1 | Vwa8 | Nfatc3 |  | Fbrs |  |  |  |  | Cdc42ep5 |  |  | Trabd | Prpf18 |
| Celf2 | Ralgapa2 | Tomm7 |  | Il1r2 |  |  |  |  | Trap1 |  |  | Ube2n | Zan |
| Sulf1 | Cyhr1 | Tbca |  | Gcnt2 |  |  |  |  | Mapre2 |  |  | Dad1 | Ints6l |
| Sigirr | Acap2 | Nudt19 |  | Sema4d |  |  |  |  | Selenos |  |  | Nsun2 | Srek1 |
| Sipa1l2 | Ocln | Ebp |  | Mafg |  |  |  |  | Fam83h |  |  | Abcf1 | Ttc14 |
| Arhgap29 | Atxn7l3b | Cwc15 |  | Arf2 |  |  |  |  | Efnb1 |  |  | Psmc6 | Supt20 |
| Lgi2 | Cebpg | Usp8 |  | Anpep |  |  |  |  | Rab22a |  |  | Ddx1 | Tc2n |
| Actr8 | Gclc | Elof1 |  | Klhl2 |  |  |  |  | Zdhhc7 |  |  | Prpf19 | Ilkap |
| Lix1l | Itch | Stx5a |  | Gtf2ird1 |  |  |  |  | Erp44 |  |  | Top1 | Rbmx |
| Tnfrsf12a | Acss1 | Pdzd8 |  | Fxyd5 |  |  |  |  | Yipf3 |  |  | Ece1 | Zfp646 |
| Tspan4 | Rap1gap | Abhd11os |  | Rab32 |  |  |  |  | Ube2q1 |  |  | Baz1b | Car13 |
| Jade2 | Tmco3 | Ndufa1 |  | E330009J07Rik |  |  |  |  | Tprn |  |  | Srsf7 | Gm28661 |
| Smim3 | Ap3b1 | Btbd7 |  | Synrg |  |  |  |  | Crybg1 |  |  | Psmd13 | Pnpla6 |
| Oaf | Pld1 | Trak2 |  | Cog1 |  |  |  |  | Sc5d |  |  | Sars | Cirbp |
| Phospho2 | H2-T22 | Ubn1 |  | Tmx2 |  |  |  |  | Itfg1 |  |  | Rangap1 | Rtf1 |
| Pigo | Cyp2d34 | Kmt2e |  | Mansc1 |  |  |  |  | Glmp |  |  | Pdap1 | Nudcd1 |
| Tmem186 | Tstd1 | Phf3 |  | Vbp1 |  |  |  |  | Ccny |  |  | Psme3 | Chd6 |
| Wwtr1 | Hmgcl | Slc38a1 |  | Ace |  |  |  |  | 0610040J01Rik |  |  | Ssb | Tgfbrap1 |
| Clasp2 | Tbl1x | Naa20 |  | Selplg |  |  |  |  | Blnk |  |  | Ube2b | 2410002F23Rik |
| Rbms2 | Dnajc14 | Elf4 |  | Emd |  |  |  |  | Ephb2 |  |  | Sumo3 | Tubgcp3 |
| Prr15 | Ccdc47 | Ndufv3 |  | Uri1 |  |  |  |  | Cul4a |  |  | Mybbp1a | Kmt5c |
| Slc41a1 | Aftph | Appbp2 |  | Sox9 |  |  |  |  | Mpc2 |  |  | Anp32e | Atp6v1c1 |
| Apoo | Prpsap1 | Ankhd1 |  | Cadps2 |  |  |  |  | Cnpy2 |  |  | Stip1 | Farp1 |
| Plekhg2 | Ubr3 | Sun1 |  | Bcat2 |  |  |  |  | Sar1b |  |  | Acot7 | Naa60 |
| Timp1 | Epb41l2 | Filip1l |  | Atg2a |  |  |  |  | Fgfbp1 |  |  | Maea | Rab27a |
| Tmem102 | Tmem98 | Grsf1 |  | D10Jhu81e |  |  |  |  | D1Ertd622e |  |  | Cers6 | Sepsecs |
| Elmo2 | Mlxip | D630039A03Rik |  | Phf23 |  |  |  |  | Nkiras2 |  |  | Gltp | Fam193b |
| Osbpl8 | Tmc5 | Anapc2 |  | Prr14 |  |  |  |  | BC031181 |  |  | Ppp1r14b | Rbm26 |
| Olfml3 | Mat2b | Rpl23a-ps3 |  | Suco |  |  |  |  | Pak4 |  |  | Tns3 | Pxylp1 |
| Tpst2 | Snx13 | Git1 |  | Usp32 |  |  |  |  | Rock1 |  |  | Hcfc1 | Pias2 |
| Rcbtb2 | Ube2d2a | 0610012G03Rik |  | Ube2f |  |  |  |  | Pak1 |  |  | Slc35a4 | Gpalpp1 |
| Arhgap31 | Mob1b | Tmem168 |  | Sh3gl1 |  |  |  |  | Slc35b2 |  |  | Tfdp1 | Tcf7l2 |
| Rcn1 | Skap2 | Cdk13 |  | Ing1 |  |  |  |  | Ndufa3 |  |  | Sec13 | Hoxa7 |
| Serpine1 | Yipf1 | Rnf220 |  | Pbx2 |  |  |  |  | Adgra3 |  |  | Mrpl12 | Tspan31 |
| Bmp1 | Hsd3b3 | Chd1 |  | Gyg |  |  |  |  | Ndufs5 |  |  | Sf3b3 | Slc12a6 |
| Camk2g | Tlk1 | Mpnd |  | Mpp1 |  |  |  |  | Kmt2d |  |  | Paics | Aaed1 |
| Rad51d | Reep5 | Bptf |  | Brpf1 |  |  |  |  | Elovl7 |  |  | Ubl3 | Gpkow |
| Elmo1 | Mecom | Arl5a |  | Lactb2 |  |  |  |  | Atp5k |  |  | Srsf10 | Zfp704 |
| Adamts4 | 9-Sep | Plcl2 |  | Slc44a2 |  |  |  |  | Cldn8 |  |  | Tars | Raf1 |
| Pdik1l | Rxra | Med13 |  | Adprh |  |  |  |  | Cr1l |  |  | Srrm1 | Galm |
| Tmem126b | Slc22a23 | Stk16 |  | Hcls1 |  |  |  |  | Grpel1 |  |  | Magt1 | Hoxa11os |
| Rcn3 | Chuk | Atox1 |  | Vip |  |  |  |  | Pdcd10 |  |  | Wsb1 | Fam76b |
| Ostm1 | Gpbp1l1 | Srp14 |  | Pitpnm1 |  |  |  |  | Ptdss1 |  |  | Mydgf | Rcn2 |
| Kdr | Tet3 | Nsmce4a |  | Adam8 |  |  |  |  | 2410015M20Rik |  |  | Zc3h15 | Sacm1l |
| Mob3b | Sptssb | Stk4 |  | Ninj1 |  |  |  |  | Npdc1 |  |  | Nampt | Cenpf |
| Tepsin | Irgm1 | Ptk2b |  | Spi1 |  |  |  |  | Chdh |  |  | Psmc5 | Rasa4 |
| Amotl1 | Prkca | R3hdm2 |  | Dennd5a |  |  |  |  | Osbpl2 |  |  | Snrpd2 | 2310022B05Rik |
| Loxl1 | Brd1 | Rap2c |  | Stk10 |  |  |  |  | Crb3 |  |  | Taf15 | Tia1 |
| Runx1 | Tmem106b | Npepl1 |  | Tank |  |  |  |  | Gbf1 |  |  | Zmiz1 | Imp4 |
| Slc43a2 | Foxa1 | Mvd |  | Svil |  |  |  |  | Etfdh |  |  | Psmd7 | Snx12 |
| Zfp180 | Cipc | Rpl6l |  | Cd33 |  |  |  |  | Mdp1 |  |  | Pgp | Plbd2 |
| Pdpn | Spdef | Med15 |  | Abcc1 |  |  |  |  | Rnf181 |  |  | Nmt1 | Gm42047 |
| Osmr | Oas1a | Polr2e |  | Madd |  |  |  |  | Strn4 |  |  | Lap3 | Dock9 |
| Msr1 | Ddrgk1 | Tmem87a |  | Prex1 |  |  |  |  | Med25 |  |  | Nr3c1 | Irak4 |
| Htra3 | Efnb2 | Tnrc6b |  | Agap3 |  |  |  |  | Rbbp6 |  |  | Dek | Spryd3 |
| Nrg1 | Adh5 | Eny2 |  | Rhou |  |  |  |  | Tom1l1 |  |  | Eif5b | Eps15l1 |
| Dcun1d2 | Zadh2 | H6pd |  | Sik3 |  |  |  |  | Cisd1 |  |  | Eif4a-ps4 | Eppk1 |
| Mcrip1 | Tmigd1 | Zmynd11 |  | Htra1 |  |  |  |  | Hif1an |  |  | Got2 | Fam120b |
| Ltbp3 | Mgam2-ps | Snf8 |  | Uqcc1 |  |  |  |  | Selenoi |  |  | Anxa8 | Sumf1 |
| Nrp1 | Rap1gap2 | Map3k7 |  | Fkbp15 |  |  |  |  | Fmr1 |  |  | Psmd1 | Pigm |
| Igf1 | Calml4 | Rc3h1 |  | Bnip2 |  |  |  |  | Fbxo18 |  |  | Rnf187 | Epm2aip1 |
| Myo5a | Tmem171 | Mrps24 |  | Ccr1 |  |  |  |  | Ccz1 |  |  | Syne2 | Fbrsl1 |
| S1pr1 | G6pdx | Atp13a1 |  | Rab31 |  |  |  |  | Grtp1 |  |  | Kidins220 | Rnf185 |
| Scara5 | Nipsnap2 | Agfg1 |  | Anks1 |  |  |  |  | Chmp6 |  |  | Ufm1 | BC005624 |
| Wnt5a | Slc13a2 | Usp22 |  | Fmnl1 |  |  |  |  | Msl2 |  |  | Xpo1 | Exoc1 |
| Mdfic | AU040320 | Ric8a |  | Cybc1 |  |  |  |  | Gid8 |  |  | Puf60 | Cwh43 |
| Itga9 | Frk | Cmip |  | Ccl3 |  |  |  |  | Nelfb |  |  | Paip2 | Epha2 |
| Chl1 | Foxo3 | Ppdpf |  | Orai2 |  |  |  |  | Ndufs6 |  |  | Ralb | Ncbp3 |
| Igkv4-72 | Inppl1 | Pde7a |  | Cln5 |  |  |  |  | Tmco1 |  |  | Amd1 | Ccdc107 |
| Hspb8 | Myo1e | Dctn3 |  | Arhgdib |  |  |  |  | Pla2g10 |  |  | Gls | Nr1d1 |
| Scube1 | Safb | Liph |  | Chil3 |  |  |  |  | Atg4d |  |  | Dync1i2 | Paxbp1 |
| Acsf3 | Rasef | Pigs |  | Eif4ebp1 |  |  |  |  | Gipc1 |  |  | Cops7a | Rspry1 |
| Zfp617 | Sipa1l3 | Scand1 |  | Lmo4 |  |  |  |  | Aifm1 |  |  | Sae1 | Tfip11 |
| Ppic | Myof | Myb |  | Pirb |  |  |  |  | Stap2 |  |  | Psma3 | Mta1 |
| Olfml2b | Gpr107 | Tspan7 |  | Birc2 |  |  |  |  | Spr |  |  | Abl1 | Vps16 |
| Colec12 | Ces2a | Fkbp2 |  | Sp100 |  |  |  |  | Mpi |  |  | Rpl7l1 | Prpf39 |
| Nin | Tmed3 | Pkd1 |  | Nfya |  |  |  |  | Marveld3 |  |  | Pink1 | Tpm3-rs7 |
| Ccdc58 | Lrrfip2 | Thoc2 |  | Ncoa1 |  |  |  |  | Psenen |  |  | Anapc1 | Clk4 |
| Immp1l | Elovl6 | Smad1 |  | Card19 |  |  |  |  | Emc7 |  |  | Fam96a | Mum1 |
| Ripor1 | Nr1h4 | Mrpl43 |  | Blcap |  |  |  |  | Ceacam20 |  |  | Adss | Setdb1 |
| Col5a3 | Myo10 | Plekhm1 |  | Usp38 |  |  |  |  | Lrrc42 |  |  | Pkp3 | Gtf3c4 |
| Csf1 | Golim4 | Rp9 |  | Degs1 |  |  |  |  | Pmpcb |  |  | Trp53inp1 | Dock6 |
| A4galt | Entpd8 | Timm44 |  | Hmox1 |  |  |  |  | Nostrin |  |  | Dap | Gsta4 |
| Mterf3 | Trim16 | Aagab |  | Rabgef1 |  |  |  |  | Tmem97 |  |  | Cdkn1b | Atxn2 |
| Ighv9-3 | Fzd5 | Pcyt1a |  | Ier3 |  |  |  |  | Guk1 |  |  | Cops6 | Atg7 |
| Arhgef40 | Selenom | Snrpe |  | Ksr1 |  |  |  |  | Ttc22 |  |  | Gspt1 | Lpcat4 |
| Ighg1 | Clcn4 | Rpl9-ps6 |  | Prps2 |  |  |  |  | Dnpep |  |  | Aqp1 | Alcam |
| Ackr3 | Tmem94 | Spen |  | Synj1 |  |  |  |  | Slc30a7 |  |  | Cdk2ap2 | Smg6 |
| Ydjc | Ap1ar | Slc36a1 |  | Ulk1 |  |  |  |  | Trim31 |  |  | Psmc2 | Man2b2 |
| Elk3 | Ifih1 | 5031439G07Rik |  | Iqsec1 |  |  |  |  | Isca1 |  |  | Atrx | Gsdmcl2 |
| Itga4 | Cdcp1 | Herc4 |  | Aldh1a3 |  |  |  |  | Faf2 |  |  | Psmb5 | Arfgap2 |
| Nkx2-3 | Znrf2 | Tcf20 |  | Arhgap42 |  |  |  |  | Ccdc93 |  |  | Selenok | Scyl3 |
| Cd163 | Sec23a | Slc50a1 |  | Dhrs7 |  |  |  |  | Apeh |  |  | Rassf3 | Zfp397 |
| Crtap | Spg7 | Ahctf1 |  | Stx6 |  |  |  |  | Ndufc2 |  |  | Dazap1 | Top3b |
| Chst15 | Nectin3 | Tmem242 |  | Zeb2 |  |  |  |  | Gpsm2 |  |  | 7-Mar | Zcchc7 |
| Pcdh7 | Slc12a7 | Mrpl54 |  | Dbt |  |  |  |  | Mcmbp |  |  | Pdcd6 | St6galnac2 |
| Pvr | Cgref1 | Ptov1 |  | Slc7a11 |  |  |  |  | Cblc |  |  | Atp6v0e | Uxs1 |
| Mknk1 | Pde3a | Nxpe4 |  | Map3k5 |  |  |  |  | Ngly1 |  |  | Ube2s | Taf4 |
| Btg3 | Pcdh1 | Mrpl24 |  | Exoc7 |  |  |  |  | Mob4 |  |  | Secisbp2l | Gm29216 |
| Ighv14-4 | Trpm7 | Chd2 |  | Gramd1a |  |  |  |  | Ssx2ip |  |  | Txnl1 | Casp6 |
| Atp8b2 | Nbeal2 | Otub1 |  | Cd300lf |  |  |  |  | Fam114a2 |  |  | Ola1 | Acp6 |
| Zfp322a | Hsd17b2 | H2afv |  | Cyth4 |  |  |  |  | Mycbp2 |  |  | Hmgn2 | Cdo1 |
| Pappa | Gpr108 | Lmnb2 |  | Per1 |  |  |  |  | Ssh3 |  |  | Psmd4 | Brf1 |
| Cd68 | Uck2 | Smg7 |  | Chic2 |  |  |  |  | Tm2d2 |  |  | Kctd5 | Erf |
| Phf20 | Parp9 | Jtb |  | Notch2 |  |  |  |  | Safb2 |  |  | Csk | Ankrd27 |
| Ednrb | Scin | Mrps21 |  | Mta3 |  |  |  |  | Eef2k |  |  | Myl12a | Lpo |
| Syt13 | Insl5 | Mzt1 |  | Kctd12 |  |  |  |  | Plekhh1 |  |  | Jpt2 | Eml2 |
| Adgra2 | Dennd4c | Pdcl3 |  | Snx10 |  |  |  |  | Pla2g16 |  |  | Mif | 4833420G17Rik |
| Ntn1 | Inava | Tmem250-ps |  | Ppp1r18 |  |  |  |  | Cpt2 |  |  | Uba2 | Mtmr9 |
| Adamts2 | Xpr1 | Cxxc1 |  | Fbxo28 |  |  |  |  | Abhd16a |  |  | Impdh2 | Gm15501 |
| Thy1 | Ddx60 | Rapgef6 |  | Foxa3 |  |  |  |  | Ccdc85c |  |  | Ccar1 | Cdadc1 |
| Cacna2d1 | Mcur1 | Slc25a17 |  | Pdss1 |  |  |  |  | Metrnl |  |  | Rnf11 | Llgl1 |
| Igkv5-43 | Slc12a8 | Arfgap1 |  | Acvrl1 |  |  |  |  | Mff |  |  | Nol7 | Efemp1 |
| Mmrn1 | Ugt1a7c | Trip10 |  | Gadd45b |  |  |  |  | Mlph |  |  | Utrn | Nrbp2 |
| Samd10 | Nrip1 | Polr2f |  | Coq10b |  |  |  |  | Blmh |  |  | Eif2s1 | Akirin2 |
| Ednra | Pde4dip | Tmx3 |  | Sil1 |  |  |  |  | Mvb12a |  |  | Slc25a51 | Nsmce1 |
| Stxbp1 | Tmed5 | Arrdc1 |  | Cxcr2 |  |  |  |  | Ube3c |  |  | Eif4a3 | Chka |
| Tspan9 | Sub1 | Emc6 |  | Ifitm6 |  |  |  |  | Prkaca |  |  | Upf1 | Zfp266 |
| Wdfy2 | Btnl6 | Aim2 |  | Dhx40 |  |  |  |  | Pgm2 |  |  | Limd1 | Engase |
| Mrps30 | Fastk | Krtcap3 |  | Pag1 |  |  |  |  | Mapkap1 |  |  | Zmiz2 | Snhg1 |
| Ms4a4a | Tbc1d15 | Ndrg3 |  | Cybb |  |  |  |  | Brip1os |  |  | Park7 | Gmeb2 |
| Ccr2 | Tmem63b | Emp2 |  | AU020206 |  |  |  |  | Tsg101 |  |  | Rars | Gm45912 |
| Cxcl14 | Arap2 | Htt |  | Lrrc8d |  |  |  |  | Pex19 |  |  | Wsb2 | Ccdc127 |
| Nxn | Mink1 | Syf2 |  | Fam76a |  |  |  |  | Arpin |  |  | Smarce1 | Ntan1 |
| Dock10 | Rreb1 | Wdr89 |  | Tfe3 |  |  |  |  | Rep15 |  |  | Ube2m | Gm13509 |
| Gem | Golga3 | Sh3glb2 |  | Lyst |  |  |  |  | Acadvl |  |  | Fubp1 | Btbd9 |
| Tmem41a | Gramd3 | Inpp5a |  | Stat5b |  |  |  |  | Ndufc1 |  |  | Aars | Cul4b |
| Arhgef17 | Pcgf3 | Ttyh3 |  | Pygl |  |  |  |  | Stx3 |  |  | Sympk | Pan2 |
| Thap2 | Aebp2 | Ccdc12 |  | Atxn10 |  |  |  |  | Pex2 |  |  | Cdipt | Arfrp1 |
| Gpm6b | Lhfpl2 | Kdm5a |  | Malt1 |  |  |  |  | Ankrd13c |  |  | Banf1 | Rnf168 |
| Rtn4ip1 | Hectd3 | Acat2 |  | Gripap1 |  |  |  |  | Pkp2 |  |  | Ddx46 | Dmac2 |
| Pctp | Abcg2 | Trpm6 |  | Ell |  |  |  |  | Pip5k1b |  |  | Ncln | Prag1 |
| Pltp | Fam114a1 | Atraid |  | Znrf1 |  |  |  |  | Keap1 |  |  | Ddx39 | Mtf2 |
| Pde3b | Usp3 | Ndufb1-ps |  | Clec4d |  |  |  |  | Hmg20b |  |  | Lamtor1 | Pard6b |
| Dlc1 | Plekha8 | Hmgn5 |  | Rdh11 |  |  |  |  | Pigq |  |  | Sgta | Lrrc47 |
| Tmx4 | Cth | Elf2 |  | Lysmd3 |  |  |  |  | Sdf2 |  |  | Sap18 | Zfp467 |
| Adgre1 | Prkaa1 | Pdzk1ip1 |  | Ica1 |  |  |  |  | Decr1 |  |  | Vti1b | Ubxn7 |
| Cd302 | Lmbrd1 | Ddc |  | Plod3 |  |  |  |  | Cnih4 |  |  | Eml4 | Zfp950 |
| Dcaf17 | Apobec3 | Hivep1 |  | Il17ra |  |  |  |  | Csnk1g3 |  |  | Napa | Ampd2 |
| 0610009B22Rik | Uaca | Elmsan1 |  | C5ar1 |  |  |  |  | Plekhg3 |  |  | Eif3g | Gm28437 |
| Phldb2 | Camta2 | Ankrd28 |  | Swap70 |  |  |  |  | Tcf4 |  |  | Ik | Gpd1 |
| Adcy7 | Agrn | Cyld |  | Cytip |  |  |  |  | Cog6 |  |  | Eif1ax | Snrnp48 |
| Fzd4 | Grb7 | Ccser2 |  | Nckap1l |  |  |  |  | Gmppa |  |  | Snrnp200 | G2e3 |
| Ldb3 | Hsd17b4 | Clns1a |  | Rnf19a |  |  |  |  | 1110059E24Rik |  |  | Glrx3 | Gnas |
| Tie1 | Asph | Mrpl2 |  | Noct |  |  |  |  | Psme2b |  |  | Aco1 | Acsl1 |
| Ms4a6b | Araf | Hoxa10 |  | Fuk |  |  |  |  | Mob2 |  |  | Tomm5 | Pabpn1 |
| Csgalnact2 | Nedd4l | Timm8b |  | Glipr2 |  |  |  |  | Stxbp3 |  |  | Smc4 | Tspan12 |
| Pf4 | Brox | Nudt3 |  | Tmcc1 |  |  |  |  | Dicer1 |  |  | Snx17 | Cdip1 |
| Pik3cg | Dnajc8 | Dctn5 |  | Mir22hg |  |  |  |  | Ddx3y |  |  | Ibtk | Man2c1 |
| Kirrel | Cnn3 | Abracl |  | Rgs3 |  |  |  |  | Pcmt1 |  |  | Serinc5 | C2cd5 |
| Lrrc32 | Ogfr | Cops9 |  | Pde4b |  |  |  |  | Pafah2 |  |  | Ip6k1 | Sugp1 |
| Itgb3 | Pcyox1 | Prdm2 |  | Cpeb2 |  |  |  |  | Setx |  |  | Rrm1 | Abhd5 |
| Eogt | L1cam | Uhrf1bp1l |  | Notch1 |  |  |  |  | Cog2 |  |  | Snx9 | Mcee |
| Sik1 | Dhrs11 | St3gal1 |  | Irak2 |  |  |  |  | Ythdc1 |  |  | Psmb7 | Orc3 |
| Plod2 | Lzts3 | Irf2bpl |  | Fpr1 |  |  |  |  | Erg28 |  |  | Mbd3 | Rnf43 |
| Cdh11 | Cdk9 | Gm14303 |  | Klhdc4 |  |  |  |  | Mrpl42 |  |  | Arrb1 | Pex13 |
| Efemp2 | Ihh | Socs4 |  | Pdlim7 |  |  |  |  | Cdca7 |  |  | Eif4e | Rpl28-ps1 |
| Lrrc8c | Gm1123 | Slc44a3 |  | Map3k3 |  |  |  |  | Aldh16a1 |  |  | Vwa5a | Zbtb44 |
| Akap12 | Gpbp1 | Gm4332 |  | Dennd4a |  |  |  |  | Fam84b |  |  | Gtf2i | Fhod1 |
| Adam23 | Chga | Irf3 |  | Arl8a |  |  |  |  | Gm10221 |  |  | Nr1h2 | Bmt2 |
| Clec4n | Trim26 | Arid1b |  | Lpcat2 |  |  |  |  | Nipsnap3b |  |  | Abhd17a | Exoc6 |
| Prdm10 | Retreg2 | Hsd17b10 |  | Rlf |  |  |  |  | Ldb1 |  |  | Sigmar1 | Hnf1b |
| Malsu1 | Ifit1 | Imp3 |  | Arhgap30 |  |  |  |  | Dcun1d1 |  |  | Psmb2 | Bmi1 |
| Ppm1f | Pdia5 | Stx4a |  | Gmfg |  |  |  |  | Pklr |  |  | Prpf4b | Ythdc2 |
| Islr | Arhgap12 | Mrpl57 |  | Maff |  |  |  |  | Asxl1 |  |  | Ccna2 | Upf3b |
| Zfp574 | Nr3c2 | Gm9616 |  | Rhbdf2 |  |  |  |  | Bcl2l13 |  |  | Psma5 | Trappc9 |
| Ggt5 | Bsdc1 | Aak1 |  | Gadd45a |  |  |  |  | Lss |  |  | Traf7 | Mtf1 |
| Acvr1 | Rap1gds1 | Map3k2 |  | 2610002M06Rik |  |  |  |  | Lrrc19 |  |  | Hs6st1 | Als2cl |
| Fyn | Nck2 | Fbxo38 |  | Limd2 |  |  |  |  | Mtfr1 |  |  | Mgat1 | Gtf3c2 |
| Fmnl2 | Ankib1 | Trmt1 |  | Id2 |  |  |  |  | Cog4 |  |  | Mcm6 | Hmg20a |
| Frmd6 | Cyp2s1 | Hoxd13 |  | Fam3a |  |  |  |  | Stau1 |  |  | Smc1a | Gstm3 |
| Igkv17-127 | Avl9 | Otud7b |  | Emilin2 |  |  |  |  | BC004004 |  |  | Serpinb5 | Yeats2 |
| Itgb7 | Map4k3 | Vcl |  | Lipg |  |  |  |  | Sptlc1 |  |  | Usp19 | Snapin |
| Ero1lb | Nhsl1 | Polr3k |  | Nfkbib |  |  |  |  | Copg2 |  |  | Tmem50a | Ttll6 |
| Gng2 | Eps15 | Bri3 |  | Csf2ra |  |  |  |  | Leprotl1 |  |  | Rnpep | Guf1 |
| Jam2 | Dip2a | Cers4 |  | Ptpn23 |  |  |  |  | Mul1 |  |  | Akr1b3 | Ankrd16 |
| Rgl1 | Lgals8 | Naxd |  | Gpr35 |  |  |  |  | Gabarapl2 |  |  | Cyb5r4 | Ttll4 |
| Bmp7 | Sbf2 | Zzz3 |  | Gm8995 |  |  |  |  | Chfr |  |  | Smg5 | Clk2 |
| Grk5 | Ppa2 | Sft2d1 |  | Sh3rf1 |  |  |  |  | Bcar1 |  |  | Nop58 | Zscan26 |
| Zfp961 | Slain2 | Gm2000 |  | Clasp1 |  |  |  |  | Lztr1 |  |  | Ubxn4 | Aspg |
| Col14a1 | Atp8a1 | Plpbp |  | Rest |  |  |  |  | Ndufb3 |  |  | Arrdc4 | Zfp68 |
| Pyurf | Pparg | Mecr |  | Wdr41 |  |  |  |  | Eif2ak1 |  |  | Cbx5 | 2010315B03Rik |
| Glipr1 | Nras | Tnks |  | Zfp131 |  |  |  |  | Nr2f6 |  |  | Mfsd4a | Mms19 |
| Ighv1-54 | Gigyf2 | Tmem70 |  | Git2 |  |  |  |  | Mpdu1 |  |  | Timm13 | Cyp4f13 |
| Gga2 | Agpat4 | Ube3a |  | Stxbp6 |  |  |  |  | Taf1 |  |  | Arhgap35 | Coq8a |
| Chpf2 | C1galt1c1 | Brap |  | Itgal |  |  |  |  | Ndufa11 |  |  | Nek6 | Smc5 |
| Mmaa | Tmed4 | Phip |  | Fbxo42 |  |  |  |  | Cx3cl1 |  |  | Tmem50b | Map2k7 |
| Glt8d1 | Clip2 | Foxk1 |  | Tiparp |  |  |  |  | Vps36 |  |  | Tmem87b | Hmgxb3 |
| Sufu | Abhd13 | Mtfr1l |  | Krit1 |  |  |  |  | Unc119b |  |  | Scd1 | Kat6b |
| Zfp516 | Fbxo25 | Pgap2 |  | Guca2b |  |  |  |  | Naa35 |  |  | Psmd6 | Tbc1d17 |
| Fbxo7 | Hpcal1 | Fxr2 |  | Sde2 |  |  |  |  | Cyth2 |  |  | Pafah1b2 | Gstz1 |
| Hip1 | Dtx3l | Gm11808 |  | Pura |  |  |  |  | Ext2 |  |  | Lonp1 | Frat2 |
| Dstyk | Lclat1 | Rhbdl2 |  | Pnkp |  |  |  |  | Pja1 |  |  | Eif2s3x | Mcoln1 |
| Arl4c | Ubxn2a | Lamtor3 |  | Aida |  |  |  |  | 2610528J11Rik |  |  | Mid1ip1 | Pgghg |
| Serpinb9 | Ktn1 | Fem1b |  | Bin3 |  |  |  |  | Polr3c |  |  | Xpnpep1 | Zscan29 |
| Amotl2 | Gusb | Pfdn1 |  | Ccl4 |  |  |  |  | Car9 |  |  | Prmt1 | Spry2 |
| Zeb1 | St3gal3 | Strn |  | Dock8 |  |  |  |  | Dyrk1a |  |  | Ctcf | Trip4 |
| Il11 | Ccdc186 | Lamtor5 |  | Atg16l1 |  |  |  |  | Tm7sf3 |  |  | Hip1r | Dqx1 |
| Hgf | Rp2 | Bola2 |  | Kdm1b |  |  |  |  | Specc1 |  |  | Wdr82 | Dnajb2 |
| Tonsl | Tbc1d9b | Mrpl14 |  | Bcl2l11 |  |  |  |  | Tbcd |  |  | Srrt | Pdk3 |
| Vps8 | Efcab14 | Slc8b1 |  | Crtc2 |  |  |  |  | Atp2c2 |  |  | Psmc4 | Alpk1 |
| Nt5dc3 | Acbd5 | Rasa2 |  | Chil1 |  |  |  |  | Trappc11 |  |  | Pmpca | Cryzl1 |
| Cmtm3 | Tom1l2 | Pcx |  | Flt1 |  |  |  |  | Cpsf7 |  |  | Nrp2 | Gigyf1 |
| Ighv14-2 | Usp53 | Utp11 |  | Hck |  |  |  |  | Mcrs1 |  |  | Csnk2b | Uimc1 |
| Bicc1 | Mpp5 | Tmem258 |  | Mkl1 |  |  |  |  | Mea1 |  |  | Naa15 | Klhl18 |
| Mmrn2 | Wwp1 | Zfas1 |  | Ptafr |  |  |  |  | Cdc25a |  |  | Sh2d4a | Rnf139 |
| Mmp19 | Lonp2 | 1600012H06Rik |  | Smo |  |  |  |  | Spire2 |  |  | Emg1 | Slc27a1 |
| Slc29a3 | Ankrd40 | Lamtor4 |  | Rffl |  |  |  |  | Bcl2l14 |  |  | Elovl1 | Tpra1 |
| Pld4 | Tcim | Atf2 |  | Sell |  |  |  |  | Glod4 |  |  | Sec24d | Plpp1 |
| Tpst1 | Daam1 | Il18 |  | Zswim4 |  |  |  |  | Gabpa |  |  | Yap1 | Gm44985 |
| Ms4a7 | Vps41 | 2610001J05Rik |  | Zfp263 |  |  |  |  | Pik3r2 |  |  | Rheb | Fgfr3 |
| Pla2g15 | Rps6ka4 | Hoxa9 |  | Pfkfb4 |  |  |  |  | Exoc3 |  |  | Rin2 | Csad |
| Fkbp10 | Cyp2j6 | Mtor |  | Apoa4 |  |  |  |  | Cox15 |  |  | Ahsa1 | Nadsyn1 |
| Irf5 | Cttnbp2nl | Mrps9 |  | Crip2 |  |  |  |  | Cab39l |  |  | Snu13 | Zfp946 |
| Hic1 | Ppip5k1 | Gjb3 |  | Sipa1 |  |  |  |  | Tfam |  |  | Glrx5 | Smap1 |
| Gpc1 | Stam2 | Tmem184b |  | Alox5ap |  |  |  |  | Tyk2 |  |  | Ppig | Slc25a25 |
| Selp | Rgp1 | Vcpip1 |  | Dtx4 |  |  |  |  | Cdk5rap3 |  |  | Sec61g | Tmem159 |
| Lats2 | Trafd1 | Rpl31-ps8 |  | Trappc12 |  |  |  |  | Ppp2r5d |  |  | Cdc34 | Nfia |
| Otulinl | Isg15 | Bscl2 |  | Clcn7 |  |  |  |  | Cript |  |  | Kmt2c | Tdp2 |
| Hsd11b1 | Mark3 | Ccdc32 |  | Fgr |  |  |  |  | Rmdn3 |  |  | Msi2 | Tmem161a |
| Fgf7 | Abhd4 | Mrpl27 |  | Mocs1 |  |  |  |  | Emc2 |  |  | Jmjd1c | Sec16b |
| Hspb7 | Abhd17b | Pgap1 |  | Slc11a1 |  |  |  |  | Ppil2 |  |  | Nhp2 | Zfp654 |
| Ighv1-75 | Lmbrd2 | Hoxa13 |  | Adpgk |  |  |  |  | Abcb8 |  |  | Pcnp | Cep104 |
| Nid2 | Nbeal1 | Spg21 |  | Coro7 |  |  |  |  | Gas2l1 |  |  | Sulf2 | Scamp3 |
| Uhrf1bp1 | Tmem165 | Rabggtb |  | Slc18b1 |  |  |  |  | S100a13 |  |  | Atic | Tada2b |
| Spsb1 | Ccdc6 | Crkl |  | Dennd1c |  |  |  |  | Stard3 |  |  | Insr | Ankra2 |
| Nrros | Plekhf2 | Dctn6 |  | Slc15a3 |  |  |  |  | Clpx |  |  | Idi1 | Ctse |
| Podn | Eif2ak2 | Gfer |  | Tet2 |  |  |  |  | Thap4 |  |  | Nudc | Ndst2 |
| Irf4 | Mtm1 | 4933434E20Rik |  | Ngp |  |  |  |  | Yif1a |  |  | Zfp740 | Mri1 |
| Fxyd6 | Slc22a18 | Thoc7 |  | Hibch |  |  |  |  | Fyttd1 |  |  | Celsr1 | Parp6 |
| Ms4a4d | Gosr1 | Tle4 |  | Tinf2 |  |  |  |  | Vps26b |  |  | Xrn2 | Lrp4 |
| Adamts9 | Ssbp4 | Nipsnap1 |  | Akna |  |  |  |  | Galk2 |  |  | Tnk2 | Tdrd3 |
| Cxcl1 | Zbp1 | Smad3 |  | Pias1 |  |  |  |  | Unc50 |  |  | Eif2b1 | Zfp386 |
| Abcd4 | Nipal3 | Armc10 |  | Fbxo33 |  |  |  |  | Bcar3 |  |  | Ube2j2 | Gys1 |
| Mrc2 | Ern2 | Virma |  | Icam1 |  |  |  |  | Mkrn2os |  |  | Cstb | Aar2 |
| Sema4c | Vps13b | Kif21b |  | Rilpl2 |  |  |  |  | Epsti1 |  |  | Pxk | Kdsr |
| Pi15 | Grpel2 | Trim33 |  | Pik3cd |  |  |  |  | Lrrc1 |  |  | Gatad1 | Gtpbp8 |
| Tmem86a | Chd9 | Tor1aip1 |  | Cd274 |  |  |  |  | Nufip2 |  |  | Mtmr3 | Letmd1 |
| Ptprm | Ctdspl | Phf2 |  | Igf1r |  |  |  |  | Elmo3 |  |  | Appl1 | Gm37494 |
| Clec7a | Scnn1a | Rnaseh2c |  | Ap1m1 |  |  |  |  | Vkorc1l1 |  |  | Snx4 | Chpf |
| Exo5 | Muc3a | Anapc7 |  | Krt90 |  |  |  |  | Alg5 |  |  | Cpeb4 | Med23 |
| Cilp | Casz1 | Slc7a1 |  | Fam3c |  |  |  |  | Mrpl10 |  |  | Irak1 | Deaf1 |
| Dnajb5 | Zfp622 | Hspa13 |  | Gas7 |  |  |  |  | Rchy1 |  |  | Camk1d | Slc29a1 |
| Chsy1 | Ano10 | Nosip |  | Jak3 |  |  |  |  | Mllt1 |  |  | Rsl1d1 | Ube2e1 |
| Pid1 | Znfx1 | Nagk |  | Mark4 |  |  |  |  | Pycrl |  |  | Tmem167 | Il11ra1 |
| Cdr2l | Golga5 | Asxl2 |  | Csrnp1 |  |  |  |  | Polr1c |  |  | Poldip3 | Tceal8 |
| Srpx2 | Mut | Anapc13 |  | Gimap6 |  |  |  |  | Rnf121 |  |  | Ube2c | Ttf1 |
| P3h3 | Iscu | Tmem256 |  | Itgam |  |  |  |  | Faf1 |  |  | Cpsf6 | Ocel1 |
| Fbxo46 | Adar | Jarid2 |  | Cd200 |  |  |  |  | Pithd1 |  |  | Zfp664 | Mib2 |
| 2700081O15Rik | Rab3gap1 | Scml4 |  | Cavin3 |  |  |  |  | Vps4a |  |  | Ttc39b | Gdap2 |
| Col27a1 | Rap2a | Prss8 |  | Ldlrap1 |  |  |  |  | Atg5 |  |  | Opa3 | Hnrnph3 |
| Sorbs3 | Wbp1 | Abhd11 |  | Plk3 |  |  |  |  | Pawr |  |  | Usp15 | Gm20559 |
| Cd4 | Ube2g1 | Zcchc14 |  | Adam19 |  |  |  |  | Pigk |  |  | Ckb | Clasrp |
| Ttl | Acad11 | Kxd1 |  | Plekho2 |  |  |  |  | Mrpl50 |  |  | Lrp5 | Med20 |
| Pde8a | Yipf4 | Tbc1d1 |  | Gpr137b-ps |  |  |  |  | Fnbp1 |  |  | Eif3d | Fbxl6 |
| Pitpnc1 | Unc5b | Ccnt1 |  | P4ha1 |  |  |  |  | Tbc1d22a |  |  | Psmd14 | Mrgbp |
| Kcne4 | Efna1 | Bod1l |  | BC037034 |  |  |  |  | Dscr3 |  |  | Isyna1 | Zfp865 |
| Zfp748 | Sema3b | Gfm2 |  | Plat |  |  |  |  | Ptcd2 |  |  | Khsrp | Adnp2 |
| Rbp1 | Rab27b | B3gat3 |  | Ripk4 |  |  |  |  | Tapbpl |  |  | Nup50 | Afap1l1 |
| Tnfsf13b | Arl4a | Med13l |  | Ric1 |  |  |  |  | Pqlc1 |  |  | Cav1 | Lrrc41 |
| Scarf2 | Slc17a9 | Bad |  | Tmem38b |  |  |  |  | Mpc1-ps |  |  | Rtf2 | Igsf3 |
| Prdm1 | Cnot6l | Fem1c |  | Alpi |  |  |  |  | Fxyd3 |  |  | Srp68 | Tomm34 |
| Cdyl | Eps8l2 | Reep4 |  | Ccdc88b |  |  |  |  | Nlrc4 |  |  | Exoc5 | Spopl |
| Ccdc51 | Cebpa | Ppp2r2d |  | Cables2 |  |  |  |  | Pank2 |  |  | Adrm1 | Lpar6 |
| Tram2 | Spata2 | Rex1bd |  | Inpp5d |  |  |  |  | Ice1 |  |  | Maf1 | BC016579 |
| Mkks | Cbs | Bod1 |  | Prkd3 |  |  |  |  | Vipas39 |  |  | Cdc123 | Gm10698 |
| Abca2 | Mkln1 | Cenpx |  | Rara |  |  |  |  | Dolpp1 |  |  | Txndc9 | Gt(ROSA)26Sor |
| Grb10 | Mbp | Gtf2a2 |  | Dck |  |  |  |  | Crat |  |  | Snrpd3 | Tprkb |
| Rftn1 | St6gal1 | Atxn1l |  | Nt5c2 |  |  |  |  | Rassf6 |  |  | Emc3 | Clec3b |
| Man1c1 | Rassf7 | Mga |  | Phf1 |  |  |  |  | Wdtc1 |  |  | Ddx23 | Ghr |
| Fam198b | Erlin1 | Sin3a |  | Tbpl1 |  |  |  |  | Phf12 |  |  | Tsc22d4 | Nfrkb |
| Dse | Irgm2 | Oxr1 |  | Fes |  |  |  |  | Sco1 |  |  | Rlim | Tbce |
| Bloc1s4 | Slc25a46 | Morc3 |  | Selenon |  |  |  |  | Nln |  |  | Erap1 | Arhgap26 |
| Olfm1 | Noxa1 | Eno3 |  | Ankrd44 |  |  |  |  | Syne1 |  |  | Palld | Endov |
| H2-M3 | Yes1 | Tmem238 |  | Foxn2 |  |  |  |  | Msl3 |  |  | Tfg | Zfand1 |
| Sphk1 | Etv6 | Fam173a |  | Lilrb4a |  |  |  |  | Cops3 |  |  | Ssu72 | Fastkd1 |
| Crem | Pik3c2b | Mrpl23 |  | Samsn1 |  |  |  |  | Rnf5 |  |  | Smu1 | Dpysl2 |
| Nlgn2 | Pcmtd1 | Lpl |  | Sh3bp5 |  |  |  |  | Pqlc3 |  |  | Cops5 | Lgals12 |
| Pcsk6 | Zfp148 | Atrn |  | Adi1 |  |  |  |  | Nipal2 |  |  | Phc2 | Zfp777 |
| Camkk2 | Tpd52l2 | Prcc |  | Rbm15 |  |  |  |  | Hyal2 |  |  | Slc30a5 | BC017158 |
| Scrn2 | Rnf115 | Mrps17 |  | Tnf |  |  |  |  | Sgk2 |  |  | Hdac3 | Anks3 |
| Ccl2 | Lgalsl | Pfdn6 |  | Fermt3 |  |  |  |  | Esco1 |  |  | Khnyn | Bora |
| Ctsk | Caskin2 | Sytl1 |  | Zfyve26 |  |  |  |  | Arhgef18 |  |  | Zdhhc21 | Gga3 |
| Arhgef25 | Wwc1 | Vrk3 |  | Ap1s3 |  |  |  |  | Nsdhl |  |  | Txlna | Asb7 |
| Txnrd2 | Sphk2 | Bdp1 |  | Arrb2 |  |  |  |  | Zfp592 |  |  | Pdpk1 | Ppp1r9a |
| Cd276 | Nhlrc3 | Pacs2 |  | Dennd3 |  |  |  |  | Tmco4 |  |  | Heatr5a | Capn15 |
| Scn1b | Tmf1 | Fibp |  | Fnip2 |  |  |  |  | Pdcl |  |  | Fbl | Rad17 |
| Ctu1 | Lmtk2 | Mrps14 |  | Mmab |  |  |  |  | Mllt3 |  |  | Kansl3 | Miip |
| Itgax | Bcl2l2 | Gm10443 |  | Ephx2 |  |  |  |  | Tmem19 |  |  | Tpp2 | Gm8989 |
| Ighv2-3 | Lrrk1 | Zfp703 |  | Vps18 |  |  |  |  | Nfs1 |  |  | Zmat2 | Zfp513 |
| Rgs1 | Surf1 | Emc4 |  | Ptges |  |  |  |  | Apmap |  |  | Usp5 | Gm7072 |
| Pxdc1 | Bst2 | Pla2g2f |  | Gimap4 |  |  |  |  | Nfu1 |  |  | Ctdnep1 | Plekhn1 |
| Cul7 | Kcnq1 | Rpl7a-ps5 |  | Isg20 |  |  |  |  | Vps37c |  |  | Ss18 | Dgcr8 |
| Susd2 | Ppp2r5e | Fundc2 |  | Nabp1 |  |  |  |  | Necap1 |  |  | Ryk | Terf2 |
| Dbn1 | Slc9a1 | Rpl38-ps2 |  | Dock2 |  |  |  |  | Papd4 |  |  | Plaa | Zmym3 |
| Rab11fip5 | Nipal1 | Mettl26 |  | Parn |  |  |  |  | Cyp4f40 |  |  | Pdk1 | Hdac11 |
| Coprs | Tmem167b | Smurf1 |  | Vav1 |  |  |  |  | Nub1 |  |  | Fam57a | Slc37a2 |
| Gm38910 | Slu7 | Ssna1 |  | Rnf169 |  |  |  |  | Fbxl19 |  |  | Gga1 | Mcc |
| Trip6 | Trio | Gstt2 |  | Vhl |  |  |  |  | Dcxr |  |  | Kmt2a | Pank4 |
| Slc12a4 | Kdm4a | Hexb |  | Cd37 |  |  |  |  | Alg9 |  |  | Suz12 | Mtx3 |
| Alpl | Ncoa2 | Nudt9 |  | Sla |  |  |  |  | Lpar1 |  |  | Dnaja3 | Dhx57 |
| Myh10 | Ppp1r10 | Fopnl |  | Themis2 |  |  |  |  | Armc8 |  |  | Scrib | Etv4 |
| Notch3 | Cd55 | Dcaf15 |  | Srpk2 |  |  |  |  | Stub1 |  |  | Triobp | Pigp |
| St3gal2 | Rnf20 | Pofut1 |  | Gmip |  |  |  |  | Sowahb |  |  | Bub3 | Trp53bp1 |
| Glis3 | Ankrd50 | Pomgnt1 |  | Il1a |  |  |  |  | Snw1 |  |  | Sash1 | Zgpat |
| Ly86 | Mtmr4 | 2410006H16Rik |  | Tmem170b |  |  |  |  | Txndc15 |  |  | Tomm40 | Ranbp6 |
| Gpr153 | Lpin2 | Brms1 |  | Pank1 |  |  |  |  | Gm9892 |  |  | 1110004F10Rik | Tmem181b-ps |
| Mettl21a | Laptm4b | Naxe |  | Rel |  |  |  |  | Snapc5 |  |  | Ppp3cb | Armc5 |
| Ccr5 | Cask | Rpl37rt |  | Snx20 |  |  |  |  | Coq5 |  |  | Ube2k | Klf16 |
| Tfpi | Creb1 | Vps72 |  | Mttp |  |  |  |  | Fam210b |  |  | Rabl6 | Pogz |
| Gpr146 | D5Ertd579e | Ggt6 |  | Mast3 |  |  |  |  | Chid1 |  |  | Nop56 | Zfp395 |
| Mterf4 | Patj | Pla2g5 |  | Nlrp3 |  |  |  |  | Mapk8ip3 |  |  | Srpk1 | Cep68 |
| Tenm3 | Glb1 | Arl5b |  | Gm5483 |  |  |  |  | Snx14 |  |  | Cacybp | Pwwp2a |
| Arhgef6 | Cacfd1 | Rps6-ps4 |  | Map4k2 |  |  |  |  | Themis3 |  |  | Wbp2 | Akap8l |
| Sdk1 | Inpp5j | Trappc6a |  | Cyfip2 |  |  |  |  | Ranbp9 |  |  | Hnrnpr | Abcc10 |
| Serpina3m | Fam117b | Chchd1 |  | Il1f9 |  |  |  |  | Bet1 |  |  | Gapvd1 | Unk |
| Fgf2 | Cutal | CAAA01118383.1 |  | Isy1 |  |  |  |  | Dpp4 |  |  | Aimp1 | Zfp317 |
| Mxra7 | Irf9 | Papd5 |  | Ikzf1 |  |  |  |  | Serhl |  |  | Eftud2 | Slc35f2 |
| Ehd3 | Myo1b | Amdhd2 |  | Lfng |  |  |  |  | Pip4p1 |  |  | Dnajc11 | Cuedc1 |
| Zdhhc8 | Tmem238l | Dcaf5 |  | Csf2rb2 |  |  |  |  | Ncald |  |  | Sec23b | Sult2b1 |
| Plcb1 | Sfr1 | Vps37a |  | Crlf3 |  |  |  |  | Bloc1s6 |  |  | Dnmt1 | Zfp160 |
| Thap6 | Prkcz | Gjb1 |  | Ggta1 |  |  |  |  | Mavs |  |  | Dars | E4f1 |
| Ssc5d | Tdrd7 | Dhrs4 |  | Wfdc21 |  |  |  |  | Cd63-ps |  |  | Wbp11 | Tfap4 |
| Inhba | Atg12 | Uqcc2 |  | Sgsm1 |  |  |  |  | Cbx7 |  |  | Dido1 | Rgcc |
| Ms4a6c | Arhgef11 | Mrpl58 |  | Slc31a2 |  |  |  |  | Tmem208 |  |  | Zc3h18 | Bsn |
| Arhgap23 | Ces1f | Romo1 |  | Gabpb1 |  |  |  |  | Tiprl |  |  | Cnppd1 | Zfp523 |
| Kcnab2 | Ceacam10 | Npc1 |  | Rnf34 |  |  |  |  | Ier3ip1 |  |  | Plin3 | Cbr4 |
| Kdelc1 | Slc37a3 | Commd7 |  | Alg6 |  |  |  |  | Ciao1 |  |  | Hras | Pigv |
| Sgce | Scamp5 | 1810022K09Rik |  | Cep120 |  |  |  |  | Slc52a3 |  |  | Gatad2a | Hbegf |
| Medag | B4galt4 | Washc2 |  | Plekho1 |  |  |  |  | Pgm3 |  |  | Slc30a9 | Ephx1 |
| Cep131 | Ptprd | Pik3c3 |  | Emb |  |  |  |  | Slc25a13 |  |  | Tnpo3 | Hyi |
| P3h4 | Prcp | Prkx |  | Gtpbp10 |  |  |  |  | Aspscr1 |  |  | Slc35b1 | Zfp512 |
| Fcna | Cited2 | Mrps16 |  | Pip4k2a |  |  |  |  | Gm13910 |  |  | Elovl5 | Ankzf1 |
| AI987944 | Spred2 | Brd9 |  | Chst11 |  |  |  |  | Lrrc26 |  |  | Pnpla8 | Twnk |
| Kctd11 | Fxr1 | Gm7536 |  | Plekhm2 |  |  |  |  | Leng9 |  |  | Mmadhc | Rassf1 |
| P3h1 | Slc16a5 | Med12 |  | Rab20 |  |  |  |  | Oxsm |  |  | Rnf7 | Ercc6 |
| Ccl7 | Rxrb | Znhit1 |  | Spns2 |  |  |  |  | Mpv17l2 |  |  | Ubr2 | Zfp715 |
| Gprc5b | Brd3 | Frrs1 |  | Arid5a |  |  |  |  | Vsp35l |  |  | Slco2a1 | Surf2 |
| Btc | Cobl | 1700123O20Rik |  | Stx11 |  |  |  |  | Bmp4 |  |  | Rbm8a | Zfp7 |
| C3ar1 | Ncoa7 | Tmem11 |  | Kdelc2 |  |  |  |  | Txndc12 |  |  | Mau2 | AC110534.5 |
| Rab3il1 | Sema5a | Rnf216 |  | Pik3ap1 |  |  |  |  | Rabif |  |  | Sec22b | Nucb2 |
| Lbp | Stx17 | Mrpl48 |  | Mical1 |  |  |  |  | Scmh1 |  |  | Trrap | Zfp608 |
| Pde1a | Tvp23b | Tnrc6c |  | Snx24 |  |  |  |  | Exoc4 |  |  | Ldah | Kiz |
| Flt4 | Maco1 | Pcnt |  | Irak3 |  |  |  |  | Triap1 |  |  | Ppp6c | S100pbp |
| Pdgfb | Kit | Rgs2 |  | Ifnar2 |  |  |  |  | Mlkl |  |  | B4galnt1 | Zfp943 |
| Shank3 | Nol4l | Sgms1 |  | Ppp1r15a |  |  |  |  | Spns1 |  |  | Hdac2 | Cep89 |
| Adgrd1 | Car12 | Upf2 |  | Cdk17 |  |  |  |  | Rnf111 |  |  | Cnih1 | Gsta1 |
| Dchs1 | Stat2 | Cnot10 |  | Fgd3 |  |  |  |  | Stard3nl |  |  | Golga7 | Fzd7 |
| Pcbp4 | Isx | G6pc3 |  | Ints12 |  |  |  |  | Zc3h13 |  |  | Aggf1 | Inpp5f |
| Jaml | Srsf9 | Tmem222 |  | Lmbr1l |  |  |  |  | Rab11fip4 |  |  | Ccdc50 | Cspp1 |
| Il2rb | Trim41 | Anapc11 |  | Hdac7 |  |  |  |  | Dcbld1 |  |  | Psmd12 | Pick1 |
| Lama1 | Arhgef28 | Nit1 |  | Dmxl2 |  |  |  |  | Dpm2 |  |  | Bpgm | Zfp942 |
| Mief2 | Pi4ka | Mindy2 |  | Fscn1 |  |  |  |  | Cd34 |  |  | Xpot | Zfp639 |
| Fam222b | Atoh1 | Smcr8 |  | Gphn |  |  |  |  | Cog5 |  |  | Dopey2 | Ppcdc |
| Aste1 | Rnft1 | Kbtbd2 |  | Nfam1 |  |  |  |  | Tex2 |  |  | Smc6 | Sbk1 |
| Ighv1-39 | Apaf1 | B4galnt3 |  | Csnk1e |  |  |  |  | Cox10 |  |  | Tmem33 | Cyp27a1 |
| Ppp3cc | Tex264 | Spty2d1 |  | Igsf6 |  |  |  |  | Ptges2 |  |  | Kpna6 | Tstd2 |
| Mapkbp1 | Kbtbd11 | Upf3a |  | Cd320 |  |  |  |  | Eapp |  |  | Mrpl20 | Ptpn13 |
| Rab34 | Mst1r | Dele1 |  | Mccc2 |  |  |  |  | Slf2 |  |  | Cse1l | Tubgcp2 |
| Npr2 | Retsat | 2210016L21Rik |  | Fabp1 |  |  |  |  | Coasy |  |  | Bcas2 | Metap1d |
| Ighv1-85 | Pnpo | Phf14 |  | Gla |  |  |  |  | Lias |  |  | Seh1l | Dguok |
| Foxf2 | Phactr4 | Senp5 |  | Heatr6 |  |  |  |  | Mtmr14 |  |  | Rbm17 | Btbd6 |
| Tmem229b | Rnd3 | Tor1a |  | Bnip3 |  |  |  |  | Prkra |  |  | Nsfl1c | Cyb561a3 |
| S1pr2 | Ptpn9 | As3mt |  | Il1rap |  |  |  |  | Lpin3 |  |  | Metap1 | Zfp618 |
| Dcbld2 | Eci1 | Cdk12 |  | Myo1f |  |  |  |  | Prdm4 |  |  | Usp1 | Mthfsd |
| Slc35e4 | Pptc7 | Rdh16 |  | Tbc1d8 |  |  |  |  | Mdn1 |  |  | Itga2 | 1110032A03Rik |
| Fgd5 | Syt7 | Spag7 |  | Cdkn2d |  |  |  |  | Fbxo8 |  |  | Egln3 | Zfp512b |
| Spry4 | Hcfc1r1 | Rab9 |  | Sorcs2 |  |  |  |  | Ticam1 |  |  | Nktr | Sp2 |
| Cadm3 | Tshz1 | Ylpm1 |  | Zfp281 |  |  |  |  | Rubcn |  |  | Tmem248 | Sertad3 |
| Wnt2b | Gm2a | Tmem65 |  | Wdfy4 |  |  |  |  | Atg2b |  |  | Tsn | Glt1d1 |
| Dusp4 | Socs6 | Arid4a |  | Samd5 |  |  |  |  | Tcf21 |  |  | Bzw2 | Msh3 |
| Nmnat1 | Parp4 | Atad2b |  | Rcsd1 |  |  |  |  | Ubr1 |  |  | Actr10 | Tmem117 |
| Samd4 | Fgd4 | Dnajc19 |  | Emp3 |  |  |  |  | Abcb10 |  |  | Smc3 | Xxylt1 |
| Cd248 | Slc4a4 | Commd8 |  | Pik3r5 |  |  |  |  | Rtca |  |  | Yars | Tbp |
| Jcad | Hps5 | Zbtb4 |  | Ripor2 |  |  |  |  | Ormdl2 |  |  | Cnp | Lrif1 |
| Iglc3 | Kat2b | Podxl |  | Stk11ip |  |  |  |  | Hccs |  |  | Gm49336 | Zfp692 |
| Slc39a6 | Gnptab | Snx25 |  | Gimap3 |  |  |  |  | Gm10053 |  |  | Sf3a1 | Phykpl |
| Syde1 | Kctd3 | Smim20 |  | B4galt3 |  |  |  |  | Arid5b |  |  | Senp3 | Yae1d1 |
| Runx3 | Ctps | Rev3l |  | Yaf2 |  |  |  |  | Vti1a |  |  | Sltm | AU022252 |
| Nsg1 | Fchsd2 | Zfp236 |  | Plcg2 |  |  |  |  | Lrrc75a |  |  | Xpo7 | Srsf4 |
| Alkbh4 | Gm44250 | Stk35 |  | Unc13d |  |  |  |  | Med16 |  |  | Ubl4a | Pomt1 |
| Dysf | Arel1 | Clpp |  | Rdh12 |  |  |  |  | Eml3 |  |  | Topbp1 | Uvssa |
| Fancl | Tfdp2 | Plxna1 |  | Lcp2 |  |  |  |  | Fars2 |  |  | Psmd11 | Hinfp |
| Ifi207 | Epb41l3 | Impdh2-ps |  | Lacc1 |  |  |  |  | Ptpmt1 |  |  | Rnps1 | Srgap1 |
| Trim6 | Hoxb9 | Fam118b |  | Fbxo30 |  |  |  |  | Gpc4 |  |  | Rbm15b | Rmi1 |
| Vasn | Ano9 | Ggact |  | Ccrl2 |  |  |  |  | Rars2 |  |  | Mthfd1 | Ppox |
| Clec4a1 | Pex6 | Lrrc8b |  | Rin3 |  |  |  |  | Plcg1 |  |  | Nudt21 | Dnah8 |
| Vstm4 | Slc35d1 | Pcbd1 |  | Ddhd2 |  |  |  |  | Mrps31 |  |  | Atp6v1h | Thap3 |
| Arhgap10 | Xrn1 | Ate1 |  | Gm26809 |  |  |  |  | Zbtb8os |  |  | Akt1s1 | Sugp2 |
| Syne3 | Sertad2 | Gna12 |  | Ptpre |  |  |  |  | Ptgr2 |  |  | Nasp | Pcid2 |
| Zfp385a | Adap1 | Slc37a4 |  | Asah2 |  |  |  |  | Commd10 |  |  | Ppp2r2a | Polm |
| Ighmbp2 | Ramp1 | Btf3l4 |  | Rnd1 |  |  |  |  | Nod1 |  |  | Ppfia1 | Zfp426 |
| Cmklr1 | Fgfr2 | Rif1 |  | Maml3 |  |  |  |  | Ankle2 |  |  | Cpsf2 | 4933431E20Rik |
| Fcgr1 | Camsap3 | Ephb3 |  | Tjap1 |  |  |  |  | Evpl |  |  | Krtcap2 | Kdm2b |
| Dnase1l3 | Pon3 | Zfx |  | Slc22a15 |  |  |  |  | Acot8 |  |  | Rb1cc1 | Bphl |
| Gpx8 | Sppl3 | Ube2q2 |  | Cxcr4 |  |  |  |  | Slc39a3 |  |  | Tcf3 | Meaf6 |
| Limk1 | Adgrg1 | Ttc4 |  | Traf1 |  |  |  |  | Mtmr11 |  |  | Cops4 | Smg8 |
| Igkv6-20 | Mier3 | Oard1 |  | R3hcc1l |  |  |  |  | Zbtb1 |  |  | Iars | Cnnm3 |
| Il3ra | Stard5 | Sesn3 |  | Jdp2 |  |  |  |  | Rufy1 |  |  | Kmt5b | Zfp120 |
| Fkbp14 | Phlpp2 | Dusp3 |  | Khk |  |  |  |  | Mustn1 |  |  | Sf3b5 | Sned1 |
| Derl3 | Arfgef3 | Gm15920 |  | Fcgr4 |  |  |  |  | Slc10a7 |  |  | Tsen34 | Tmem161b |
| Rgma | Ttc3 | Epc2 |  | Adamtsl4 |  |  |  |  | Fam160b1 |  |  | Cstf2 | Klhl26 |
| Numbl | Mgll | Sos1 |  | Il10ra |  |  |  |  | Ccdc82 |  |  | Tnfaip8 | Etfbkmt |
| Nfatc4 | Trappc6b | Rai14 |  | Dock11 |  |  |  |  | Mylip |  |  | Trim56 | mt-Nd3 |
| Rgs4 | Casp2 | Cers5 |  | Notch4 |  |  |  |  | Cav2 |  |  | Fem1a | Arhgef39 |
| Tifab | Slc1a4 | Sharpin |  | Zbtb11 |  |  |  |  | Iah1 |  |  | Gcn1l1 | Fance |
| Cd48 | Tmem246 | Pigu |  | Basp1 |  |  |  |  | Panx1 |  |  | Pes1 | Mpc1 |
| Lair1 | Bace2 | Trim30a |  | Fyb |  |  |  |  | Flad1 |  |  | Gm7964 | Zfp629 |
| Pla1a | Ank3 | Ubxn8 |  | Tlr13 |  |  |  |  | Rce1 |  |  | Gna14 | Btrc |
| Clec4a2 | Mocs2 | Tlk2 |  | Cwc25 |  |  |  |  | Dgcr6 |  |  | Dera | Styx |
| Csf3 | Rb1 | Dnajc1 |  | Esam |  |  |  |  | B3galnt2 |  |  | Sf3b6 | Maob |
| Atp10a | Josd1 | Traf6 |  | Nfil3 |  |  |  |  | Kdelr3 |  |  | Mcm7 | Fam219b |
| Tmem100 | Insig2 | Cyp2c68 |  | Bst1 |  |  |  |  | Apool |  |  | Egln1 | Stra6l |
| Cyyr1 | Foxd2 | Fnip1 |  | Crlf2 |  |  |  |  | Nudt14 |  |  | Rfwd3 | Zfp948 |
| Dio2 | Agtrap | Clic6 |  | Enpep |  |  |  |  | Mdc1 |  |  | Traf4 | Repin1 |
| Tubb3 | Narf | Gm5854 |  | Ncf4 |  |  |  |  | Dip2b |  |  | Eif2b5 | Zfp143 |
| Intu | Zdhhc9 | Tnni1 |  | Osgin1 |  |  |  |  | Tm7sf2 |  |  | Mafk | Zdhhc1 |
| Prickle1 | Urgcp | Samd8 |  | Sesn2 |  |  |  |  | 5-Sep |  |  | Ctr9 | Gm13648 |
| Lpxn | Tor3a | Ppil4 |  | Rhobtb2 |  |  |  |  | Nsmaf |  |  | E2f4 | Inpp5e |
| Chrdl1 | 4930539E08Rik | Gm13841 |  | Slc16a6 |  |  |  |  | Slc9a6 |  |  | Ero1l | Rhbdd2 |
| Prickle2 | Zmym2 | Bloc1s2 |  | Sis |  |  |  |  | Pikfyve |  |  | Gtf2f1 | Hhip |
| Aoah | Sema4b | Sap30l |  | Procr |  |  |  |  | Gm4737 |  |  | Rnf13 | Ap5z1 |
| Gtdc1 | Arhgap18 | Gsta3 |  | Tcp11l2 |  |  |  |  | Dcp1a |  |  | Plek2 | Tbc1d24 |
| Scara3 | Igsf9 | Med9 |  | Ppm1m |  |  |  |  | Foxo1 |  |  | Ddx24 | Ccdc28a |
| Lif | Tppp | Tmem205 |  | Rassf5 |  |  |  |  | Ppcs |  |  | Txndc11 | Per3 |
| Ptpn22 | Cgnl1 | Lrrc45 |  | Epb41l5 |  |  |  |  | Stx8 |  |  | Snx6 | Nrbf2 |
| Pcdhgc3 | Wipf2 | AI837181 |  | Rbm38 |  |  |  |  | Dmtf1 |  |  | Pik3ca | Zfp628 |
| Fam219a | Lrrc58 | Ptpn18 |  | Fmnl3 |  |  |  |  | Washc3 |  |  | Qrich1 | Zbtb21 |
| Ptprn | Tsc2 | Mfap3 |  | Egfl7 |  |  |  |  | Trim39 |  |  | Klhl24 | Polr3gl |
| Tmem177 | Aig1 | Tlr4 |  | Slc15a4 |  |  |  |  | Micall2 |  |  | Elavl1 | Mmp28 |
| Armcx2 | Naprt | Ift52 |  | Pnpla7 |  |  |  |  | Trim11 |  |  | Nolc1 | Zfp101 |
| Zfp651 | Ifi27 | Ccdc117 |  | Nsun3 |  |  |  |  | Emsy |  |  | Met | Ripk2 |
| Nes | Rnf126 | Mrps6 |  | Prkch |  |  |  |  | Nqo2 |  |  | Raph1 | Zfp553 |
| Slamf7 | Cmbl | Zfand2b |  | Ssh1 |  |  |  |  | Prelid2 |  |  | Polr2b | Eif4e3 |
| Dusp22 | Abca3 | Clcc1 |  | Ccl5 |  |  |  |  | Mtx1 |  |  | Mtx2 | Qpctl |
| Gypc | Fbxo9 | Dus3l |  | Ogfrl1 |  |  |  |  | Eid1 |  |  | Ckap5 | Pcgf2 |
| Bmp6 | Rdx | Dpm3 |  | Tbc1d9 |  |  |  |  | Tada3 |  |  | U2af1 | Vamp1 |
| Dennd2a | Atg13 | Tmem126a |  | Tmem185b |  |  |  |  | Rab12 |  |  | Gfm1 | Slc19a1 |
| Sema3g | Plekha3 | Pih1d1 |  | Inpp4a |  |  |  |  | Phf21a |  |  | Scyl1 | Nemp1 |
| Kcnmb1 | Ces1d | Zfp787 |  | Mtmr10 |  |  |  |  | Cd36 |  |  | Nsd2 | Carnmt1 |
| Chrdl2 | Smchd1 | Snhg18 |  | Il6ra |  |  |  |  | Plekhh3 |  |  | Sfxn1 | Ddx55 |
| Clec4a3 | Btnl4 | Impdh1 |  | Cdr2 |  |  |  |  | Tmem129 |  |  | Ndufb6 | Zfp212 |
| Npl | Gabarapl1 | Unc119 |  | Ltb |  |  |  |  | Gm10076 |  |  | Pik3c2a | Spsb2 |
| C1qtnf1 | Plxna2 | Gm15772 |  | Osm |  |  |  |  | Nav1 |  |  | Nfat5 | Rmnd1 |
| Pald1 | Slc39a5 | Amz2 |  | Arid3a |  |  |  |  | Umad1 |  |  | H2afx | Slx1b |
| P4ha2 | Oas3 | Phax |  | Nfkbie |  |  |  |  | Arl6ip6 |  |  | Lrpprc | Ercc4 |
| Hhipl1 | Snx7 | Bmp2 |  | Rsph3a |  |  |  |  | Lemd3 |  |  | Ncbp1 | Igip |
| Clip3 | Nsmf | C330007P06Rik |  | Fam199x |  |  |  |  | Cep250 |  |  | Tbc1d14 | Wdfy1 |
| Ighv1-69 | Ank | Brcc3 |  | 1600014C10Rik |  |  |  |  | Bet1l |  |  | Dnajb1 | Stoml1 |
| Parvb | Ppp1r16a | Trappc1 |  | Enox2 |  |  |  |  | Pdss2 |  |  | Fnta | Coq8b |
| Spire1 | Kif16b | Exoc2 |  | Fas |  |  |  |  | Wdr37 |  |  | Vps53 | Pbld2 |
| Reck | Map3k21 | Tmem5 |  | Klf7 |  |  |  |  | Fbxo6 |  |  | Got1 | Gm10031 |
| Dedd | Zbtb22 | Zhx1 |  | Cyp4f18 |  |  |  |  | Igsf5 |  |  | Gps1 | Ap4m1 |
| Ly9 | Tle1 | Fam96b |  | Tcf7 |  |  |  |  | Fbxo31 |  |  | Tbcb | Mthfd2l |
| Rassf8 | Foxn3 | Ndufaf8 |  | AC160637.1 |  |  |  |  | Pigc |  |  | Rrp7a | Wfs1 |
| Ahi1 | Kcnk5 | Ift172 |  | Mapk7 |  |  |  |  | Hacd1 |  |  | Babam1 | Ttr |
| Erg | Slc10a2 | Klhl22 |  | Cyp4v3 |  |  |  |  | Ascc1 |  |  | Timm23 | Ttyh2 |
| Cacng7 | Espn | Dalrd3 |  | C9orf72 |  |  |  |  | Ahr |  |  | Afg3l1 | Ckap2 |
| Ldlrad4 | Dennd1b | Sppl2b |  | Arhgap9 |  |  |  |  | Mical3 |  |  | Ubqln4 | Taf6 |
| Mr1 | Myo19 | Plch2 |  | Map3k8 |  |  |  |  | Oxnad1 |  |  | Derl2 | Swt1 |
| Zfp414 | Ppp1r13b | Rbsn |  | Gpatch2l |  |  |  |  | Fam83g |  |  | Dctn4 | Prdm15 |
| Rab7b | Ufd1 | Pdrg1 |  | Slc2a3 |  |  |  |  | Cgrrf1 |  |  | Asna1 | Ppp4r1l-ps |
| Nr4a2 | Foxo4 | Gm10250 |  | Rasd1 |  |  |  |  | Bicdl2 |  |  | Cdca3 | Cchcr1 |
| Ttc7b | Epb41l1 | Commd4 |  | Donson |  |  |  |  | Mtif3 |  |  | Pclaf | Rnpc3 |
| 4931414P19Rik | Erlec1 | Hdhd2 |  | Sh2d3c |  |  |  |  | Hr |  |  | Mpzl1 | Zfp738 |
| Gpr171 | Cpq | Capn13 |  | Dok3 |  |  |  |  | Srd5a3 |  |  | Rcc1 | Zfp362 |
| Il1rl2 | Cyp4f16 | Gcdh |  | Myo1g |  |  |  |  | Bcam |  |  | Ncapd2 | Ano8 |
| Atp1b2 | Nr5a2 | Eri3 |  | Tnip2 |  |  |  |  | Tcaf1 |  |  | Dcun1d5 | Rcor3 |
| Itpripl1 | Mid2 | Mapk8 |  | Lrrc25 |  |  |  |  | Tmem150b |  |  | Slfn9 | Fbf1 |
| Rerg | Magi1 | Rdh13 |  | Ccdc134 |  |  |  |  | Ankrd9 |  |  | Ccar2 | Sec22c |
| Serpina3f | Zkscan1 | Acot13 |  | Vps33b |  |  |  |  | Smug1 |  |  | Smurf2 | Zfp945 |
| Cadm4 | Thumpd3 | Stk19 |  | Bcl6 |  |  |  |  | Psd |  |  | Mogs | Lrrcc1 |
| Slc9a9 | Nqo1 | Washc1 |  | Fntb |  |  |  |  | Tceanc2 |  |  | Mxi1 | Krba1 |
| Plekhf1 | Rtp4 | Tab1 |  | Ggt1 |  |  |  |  | Nr2f2 |  |  | Snrpg | Gm20300 |
| Glis2 | Optn | Rbfa |  | Ace2 |  |  |  |  | Tmem131l |  |  | Kmt2b | Tspan33 |
| Pfn2 | Ttc38 | Gpr89 |  | Palm |  |  |  |  | Zfp451 |  |  | Cd2bp2 | Fam173b |
| Nxpe5 | Slc39a8 | Cox19 |  | Mefv |  |  |  |  | Bloc1s3 |  |  | Zmpste24 | Snap47 |
| Plxdc2 | Enpp5 | Ttc5 |  | Aldh5a1 |  |  |  |  | Capn10 |  |  | Gtf2h1 | Rfxank |
| Snta1 | Dgkh | Nf1 |  | Aldh3b1 |  |  |  |  | Mien1 |  |  | Creld2 | Ing2 |
| Ighv3-5 | Gpatch8 | Tcf7l1 |  | Ift140 |  |  |  |  | Prkrip1 |  |  | Minpp1 | Snapc3 |
| Apbb1 | Gstk1 | Kctd2 |  | Tkfc |  |  |  |  | Cep19 |  |  | Cbx1 | Camta1 |
| Samd14 | Fbxw5 | Fam53a |  | Stfa2l1 |  |  |  |  | Secisbp2 |  |  | Rraga | Hunk |
| Mt2 | Dhrs7b | Ssbp1 |  | Trub2 |  |  |  |  | Fmc1 |  |  | Mcm5 | 2310022A10Rik |
| Gfpt2 | Arhgef38 | Gstt3 |  | Ada |  |  |  |  | Cfap36 |  |  | Anapc16 | Zfp747 |
| Ak1 | Mon1a | Txndc16 |  | Ap1s2 |  |  |  |  | Enpp2 |  |  | Smarcc1 | Zfp944 |
| Enho | Cracr2a | Idnk |  | Fpr2 |  |  |  |  | Htra2 |  |  | Ebna1bp2 | 2810013P06Rik |
| Serpinf1 | Dnajb12 | Acbd6 |  | Sash3 |  |  |  |  | Oser1 |  |  | Usp14 | Lrrc8e |
| Epdr1 | Rbm22 | Rpl9-ps4 |  | Cd300ld |  |  |  |  | Fam172a |  |  | Heca | Cnpy4 |
| Gja4 | Pkig | Rab40c |  | Cfap43 |  |  |  |  | Tmub1 |  |  | Cfdp1 | 2410131K14Rik |
| Mrgprf | St5 | Camk1 |  | Ubtd1 |  |  |  |  | Gm5805 |  |  | Yipf5 | Fzd6 |
| Afap1l2 | Tmem115 | Urod |  | Ifnlr1 |  |  |  |  | Zfp35 |  |  | Mrpl17 | Tnfrsf14 |
| Galnt18 | Osbpl7 | Togaram1 |  | Slco3a1 |  |  |  |  | Rabepk |  |  | Mrpl28 | Dhx34 |
| Pard6g | Rbl1 | Trnau1ap |  | Btbd10 |  |  |  |  | Zfp335 |  |  | Ralgps2 | Leng1 |
| Fosb | Mroh1 | Gcc1 |  | Fam213a |  |  |  |  | Alg14 |  |  | Hectd4 | Slc10a3 |
| Tubb2b | Acot11 | Elp5 |  | Slc12a9 |  |  |  |  | Tk2 |  |  | Dr1 | Odf2l |
| Rgs16 | Ifit2 | Gm9493 |  | Apbb1ip |  |  |  |  | Mad2l1bp |  |  | Slc23a2 | Tagap1 |
| Igkv12-98 | Ammecr1l | Slc7a6os |  | Igsf23 |  |  |  |  | Gtf3c3 |  |  | Rbm10 | Cdk10 |
| Srpx | Neurl4 | Thap11 |  | Arg1 |  |  |  |  | Abi3bp |  |  | Dap3 | Htr4 |
| Zfp125 | Phkb | Smim22 |  | Flvcr1 |  |  |  |  | Fbxw7 |  |  | Hars | Mfap1b |
| Tshz3 | Zfyve1 | Mcrip2 |  | Sh3bp2 |  |  |  |  | Tmem68 |  |  | Tmem214 | Alkbh1 |
| Renbp | Uckl1 | Bbip1 |  | Card6 |  |  |  |  | Gm7367 |  |  | Trim27 | 2510002D24Rik |
| Mmp23 | Pex7 | Mrpl32 |  | Pacs1 |  |  |  |  | Cblb |  |  | C1d | Spats2 |
| Adamts7 | Pgpep1 | Rrp36 |  | Hmces |  |  |  |  | Mcat |  |  | Vps29 | Trim23 |
| Trpa1 | Oasl1 | Fads3 |  | F10 |  |  |  |  | Slc5a9 |  |  | Atp6v1d | Snx21 |
| Ctla4 | Oas1g | Dctpp1 |  | Bin2 |  |  |  |  | Tpcn2 |  |  | Atf7ip | Brat1 |
| Gpx7 | Trp53bp2 | Prr14l |  | Mcemp1 |  |  |  |  | Zfp318 |  |  | Atad3a | Rida |
| Lmcd1 | Pdgfa | Sav1 |  | Supt7l |  |  |  |  | Nudt22 |  |  | Wdr43 | Tmem80 |
| Gjc1 | Naa30 | Maml1 |  | Stat5a |  |  |  |  | Cyp2d10 |  |  | Bccip | Hdac6 |
| Calhm2 | Foxj3 | Hopx |  | Angptl4 |  |  |  |  | Hdhd3 |  |  | Tdg | Lyrm9 |
| Ptgis | Nhlrc2 | Atp11c |  | Gbp5 |  |  |  |  | Fam160a1 |  |  | Ralgapa1 | Whrn |
| 5730409E04Rik | Ilvbl | Rpl36a-ps2 |  | Rgs19 |  |  |  |  | Aldoc |  |  | Kcnn4 | Ggcx |
| Ccdc102a | Btnl1 | BC003965 |  | Slfn1 |  |  |  |  | Nudt7 |  |  | Dda1 | Ybx2 |
| B3galnt1 | Ttc17 | Gstm5 |  | Arap3 |  |  |  |  | Zfp276 |  |  | Lrpap1 | Gm3608 |
| Tgfb3 | Uba7 | Ebag9 |  | Arg2 |  |  |  |  | Iba57 |  |  | Rpa1 | Usp20 |
| B4galt2 | Cldn23 | Mcoln2 |  | Tmc8 |  |  |  |  | Impact |  |  | Gm10039 | Sfi1 |
| Ighv1-59 | Eci2 | Nenf |  | Hcar2 |  |  |  |  | Tmlhe |  |  | Cdc27 | Xpa |
| Rspo3 | Crebrf | Hint2 |  | Dagla |  |  |  |  | Paox |  |  | Cpsf1 | Bicdl1 |
| Ifi205 | Sox13 | Ngrn |  | Pde1b |  |  |  |  | Prorsd1 |  |  | Phrf1 | Arrdc2 |
| Milr1 | Card10 | Tmem263 |  | Ing3 |  |  |  |  | Bicra |  |  | Bap1 | Dclre1a |
| Trem2 | Rabgap1 | Svbp |  | Slc39a13 |  |  |  |  | Ptbp2 |  |  | Ints3 | Macrod1 |
| Tgfb2 | Smndc1 | Lrrc40 |  | Sp140 |  |  |  |  | Atg4c |  |  | Alyref | Frat1 |
| Icos | Mal | Ccdc85b |  | Map2k6 |  |  |  |  | Armh3 |  |  | Plekha1 | Cc2d2a |
| Gm4149 | Scaf4 | Enoph1 |  | Cmtm7 |  |  |  |  | Capn9 |  |  | Kif11 | Nfkbil1 |
| Asap3 | Cebpz | Atg9a |  | Dusp5 |  |  |  |  | Mzt2 |  |  | Prep | Mex3d |
| Hebp1 | Stim2 | Inafm2 |  | 1-Sep |  |  |  |  | Kazn |  |  | Sdf2l1 | Ganc |
| Wisp1 | Msantd4 | Pnkd |  | Kbtbd7 |  |  |  |  | Lifr |  |  | Ccdc124 | Snhg17 |
| Serpinb6b | Klhl5 | Srek1ip1 |  | Acap1 |  |  |  |  | Mlst8 |  |  | Racgap1 | Mlxipl |
| Prx | Pard3 | Cep63 |  | Prap1 |  |  |  |  | Rusc2 |  |  | Frg1 | Bcl7a |
| Rnase6 | Nde1 | Gm10132 |  | Lipe |  |  |  |  | Yipf2 |  |  | Gm10282 | Otud3 |
| Cbx2 | Ccm2 | Slc15a1 |  | Ppm1d |  |  |  |  | Ndufab1-ps |  |  | Fzr1 | Sap30 |
| Bdkrb1 | Cnst | Snrnp27 |  | Sema6b |  |  |  |  | Tspan5 |  |  | Scap | Zfp65 |
| Tlr7 | Epn3 | Peak1 |  | Gimap8 |  |  |  |  | Ubxn6 |  |  | Nup88 | Tha1 |
| Cysltr1 | Rab3ip | Commd9 |  | Hilpda |  |  |  |  | Klhl20 |  |  | Vezf1 | Nek4 |
| Arhgap28 | Decr2 | Pds5b |  | Dram1 |  |  |  |  | Mosmo |  |  | Ubap2 | Rab10os |
| Mras | Xab2 | Cfap20 |  | Gimap1 |  |  |  |  | Mkrn2 |  |  | Ash2l | Slc35e3 |
| Loxl3 | Pml | Gstp-ps |  | Ankrd22 |  |  |  |  | Fam241b |  |  | Axin1 | Dclre1b |
| Abcg3 | Etv5 | Ulk2 |  | Napepld |  |  |  |  | Nt5c3b |  |  | Zfp292 | Zfp398 |
| Apold1 | Plpp6 | Polr2h |  | Map3k14 |  |  |  |  | Zfp954 |  |  | Syvn1 | Cep162 |
| Tbkbp1 | Tmem135 | Patl1 |  | Dcun1d3 |  |  |  |  | Slc35e2 |  |  | Gm13835 | Hottip |
| Gm49331 | Paf1 | Adamts15 |  | Atpaf1 |  |  |  |  | Ccdc90b |  |  | Ranbp3 | Camk2b |
| Iglv3 | Hagh | Dcaf10 |  | Gpat3 |  |  |  |  | Ndufaf2 |  |  | Parp1 | Nek8 |
| Npr1 | Ap5m1 | Chrac1 |  | Evl |  |  |  |  | Tubgcp6 |  |  | Ankrd52 | Klhdc7a |
| Fbxo10 | Rbl2 | Chchd7 |  | Gsap |  |  |  |  | Gtf3a |  |  | Ilf2 | Wdr59 |
| Apcdd1 | Abhd6 | Hars2 |  | Vill |  |  |  |  | Mnat1 |  |  | Zswim8 | Fer |
| Hspa12b | Frs2 | 0610037L13Rik |  | Dtx2 |  |  |  |  | Zbtb8a |  |  | Phf5a | Zfp994 |
| Gimap7 | Ptpn2 | Tmem160 |  | Mocos |  |  |  |  | Trim3 |  |  | Ifrd2 | Tarbp1 |
| Slc16a2 | Zfp110 | Osbpl11 |  | Cdc42ep2 |  |  |  |  | Cutc |  |  | Arpc5l | Ice2 |
| Rasl11b | Gpt | Zbtb41 |  | Cd84 |  |  |  |  | Coq3 |  |  | Get4 | Rrnad1 |
| Arsi | Fam13a | Tpgs1 |  | Trim47 |  |  |  |  | Tmem62 |  |  | Farsb | Ccdc28b |
| Cyp7b1 | Bmp3 | AI854703 |  | AB124611 |  |  |  |  | Ghdc |  |  | Tpp1 | Spidr |
| Aldh1a2 | Dym | Cacna1h |  | Lax1 |  |  |  |  | Tbc1d30 |  |  | Cdkn2aipnl | Lsm11 |
| Rnf144b | Lrrc66 | Rpa3 |  | Gpr20 |  |  |  |  | Dis3l2 |  |  | Eif3j1 | Zfp408 |
| Frzb | Zcchc2 | Acpp |  | Dleu2 |  |  |  |  | Bloc1s5 |  |  | Kpna3 | Zfp329 |
| Fst | Ptger4 | Foxa2 |  | Siglece |  |  |  |  | Zfp503 |  |  | Rras2 | Trub1 |
| Gpr55 | Ipo9 | Stambp |  | Xylt1 |  |  |  |  | Gramd1b |  |  | AI661453 | Snapc4 |
| Stbd1 | Kdm5d | Gm3362 |  | Nsun4 |  |  |  |  | Inpp4b |  |  | Mrps23 | Hoxa5 |
| Pth1r | Rit1 | Osbpl3 |  | Slc7a8 |  |  |  |  | Ndufaf1 |  |  | Prpf6 | Lrsam1 |
| Nrtn | Ergic2 | Zcchc9 |  | Jmjd8 |  |  |  |  | Pigf |  |  | Usp16 | Ces2g |
| Igkv4-70 | Acad8 | Rnaseh2a |  | Il16 |  |  |  |  | Pllp |  |  | Cdk1 | Hdac10 |
| Loxl4 | Foxd2os | Narfl |  | Gbp9 |  |  |  |  | 1110008L16Rik |  |  | Itpa | Zup1 |
| Il6 | Hltf | Ptgs1 |  | Slc2a6 |  |  |  |  | Dtx3 |  |  | Vamp2 | Gm12715 |
| Prr16 | Slc30a1 | Fgf1 |  | Ifi203 |  |  |  |  | Alg1 |  |  | Ddx47 | Plekhg5 |
| Ackr1 | Fpgt | Cmc1 |  | Snai1 |  |  |  |  | Pyroxd1 |  |  | Zfp638 | Tinag |
| Ptx3 | Nmi | Trappc2l |  | Rasgrp2 |  |  |  |  | Afap1 |  |  | Denr | Zfp113 |
| Fdxacb1 | 9930021J03Rik | Mrps33 |  | Gtf2ird2 |  |  |  |  | Dll1 |  |  | Med22 | Eno1b |
| Zfp873 | Trmt2b | Steap3 |  | Rnf144a |  |  |  |  | Tldc1 |  |  | Nup62 | Coq10a |
| Dact1 | Sh3kbp1 | Taf6l |  | Eva1b |  |  |  |  | Zhx3 |  |  | Drg1 | Rfx5 |
| Reep1 | Rnf31 | Med21 |  | Gm1966 |  |  |  |  | Adamts10 |  |  | Ythdf1 | Phka2 |
| C1qtnf7 | Prkag2 | Sgf29 |  | Lck |  |  |  |  | Adgrl1 |  |  | Plce1 | Usp49 |
| Cercam | Slc25a16 | Gab2 |  | Ramp2 |  |  |  |  | Apbb2 |  |  | Uba5 | Pde8b |
| Tnfsf11 | Gmcl1 | Slc35a5 |  | Spn |  |  |  |  | Polk |  |  | Gmppb | Zfp626 |
| Nnmt | Psd3 | Sh3d21 |  | F630028O10Rik |  |  |  |  | Cyp2d9 |  |  | Lars | Prdm16 |
| Gm14295 | Prepl | Mnt |  | Dennd4b |  |  |  |  | Gm12251 |  |  | Ranbp1 | Ankrd13b |
| Azin2 | Cnpy3 | Ankmy2 |  | Gm49172 |  |  |  |  | Evi5l |  |  | Hspa4l | Ahsa2 |
| Gm14698 | Dvl3 | Dnajc4 |  | Gm7665 |  |  |  |  | Tomm40l |  |  | Foxk2 | Zmym6 |
| Trim72 | Fam13b | Parp2 |  | Tarm1 |  |  |  |  | Trmt11 |  |  | Snrpd1 | Hspa12a |
| Tlr8 | Car8 | Zfyve19 |  | Vwa1 |  |  |  |  | Emc1 |  |  | Setd7 | Zfp707 |
| H2afy2 | Prrg2 | Naa10 |  | Pdlim4 |  |  |  |  | Pex12 |  |  | Fcgrt | Mettl27 |
| Gpr65 | Trim30d | Enpp1 |  | Pi16 |  |  |  |  | Dusp18 |  |  | Tor4a | Zfp369 |
| Mfap2 | Adra2a | Smg9 |  | Cyp3a11 |  |  |  |  | Lpar5 |  |  | Dhx16 | H2-K2 |
| Efs | Sh3yl1 | Mcts1 |  | Zc3h10 |  |  |  |  | Sdhaf4 |  |  | Rnf38 | Hexdc |
| Lat2 | Dopey1 | Rybp |  | Krt23 |  |  |  |  | Zfp12 |  |  | Ttc9c | Il17re |
| Fam20a | Tcea3 | Tsc22d2 |  | Arhgap4 |  |  |  |  | Zmat5 |  |  | Trappc3 | Snhg12 |
| Pcbp3 | Mbtd1 | Fam168a |  | Ppp1r16b |  |  |  |  | Gnb4 |  |  | Ncoa4 | Sfxn2 |
| Slc10a6 | Gal3st1 | Mfsd13a |  | Tbc1d10c |  |  |  |  | Zfp37 |  |  | Cabin1 | Zcchc4 |
| mt-Tl2 | Igsf8 | Gatd1 |  | Il2rg |  |  |  |  | Gm3571 |  |  | Pgrmc2 | Lrrc20 |
| Tmem237 | Ccs | Osgep |  | Rasal3 |  |  |  |  | Zfp346 |  |  | Kansl1 | C87436 |
| Upk1b | Tsc1 | Snrnp25 |  | Nfkbid |  |  |  |  | Ppm1j |  |  | Dock7 | Zfp606 |
| Tspyl3 | Gse1 | Gnptg |  | Gpsm3 |  |  |  |  | Trib2 |  |  | Npm3 | Osbpl6 |
| C430042M11Rik | Lcorl | Wdr24 |  | Mndal |  |  |  |  | Tom1 |  |  | C2cd2l | Trit1 |
| Rrad | Tmtc2 | Ift46 |  | Sox18 |  |  |  |  | Rundc3a |  |  | Larp1b | Ap5b1 |
| Fjx1 | Ehhadh | Babam2 |  | Apobr |  |  |  |  | Cdkl2 |  |  | Mrpl18 | Cntrob |
| Ramp3 | Daxx | Pin4 |  | Ntng2 |  |  |  |  | Stmn2 |  |  | Snrk | Pex1 |
| Kcns3 | Fads1 | Exosc5 |  | Gm37416 |  |  |  |  | 4930523C07Rik |  |  | Bckdk | Mrm1 |
| Eid2 | Hpd | Sprtn |  | Prok2 |  |  |  |  | Swsap1 |  |  | Ptpra | Dgke |
| Lag3 | Nceh1 | N4bp2 |  | Cish |  |  |  |  | Lmo2 |  |  | Rell1 | Nprl3 |
| Gpr37l1 | Nuak2 | Atf5 |  | Tmem154 |  |  |  |  | Cox11 |  |  | Noc2l | Fsd1l |
| Slc41a3 | Zfp687 | Uqcc3 |  | Armc7 |  |  |  |  | 9930104L06Rik |  |  | Tmem147 | Snhg20 |
| Layn | Jade1 | Tm2d3 |  | Il18rap |  |  |  |  | Asb3 |  |  | Rpe | Phf21b |
| Homer3 | Tlr3 | Sesn1 |  | Fam122a |  |  |  |  | Cox16 |  |  | Ube2a | Hoxa3 |
| Arl11 | Akr1e1 | Fam208b |  | Slc28a2 |  |  |  |  | Dclre1c |  |  | Mcm3 | Cttnbp2 |
| Ch25h | Psip1 | Glrx2 |  | Elp4 |  |  |  |  | Zfp142 |  |  | Pygo2 | Xrcc3 |
| Rtl8c | Cdc14a | Phlpp1 |  | Dtx1 |  |  |  |  | Lyrm2 |  |  | Fam214b | Phkg2 |
| Fam171a2 | Vps11 | Ogfod2 |  | Pfkm |  |  |  |  | Coq4 |  |  | Skiv2l2 | Zkscan5 |
| C7 | Cdc40 | 1700021F05Rik |  | Ctla2a |  |  |  |  | Fgd2 |  |  | Ppp1r11 | Pbx3 |
| Il13ra2 | Sidt1 | Naglu |  | Il34 |  |  |  |  | Brms1l |  |  | Ccnb1 | Zfp810 |
| Ighv3-1 | Irs2 | Mrpl55 |  | Was |  |  |  |  | Pik3ip1 |  |  | Aldh18a1 | Cep70 |
| Gpr34 | Crybg2 | Ctps2 |  | Pstpip1 |  |  |  |  | Arl3 |  |  | Trp53 | Zfp511 |
| Tigit | Zfp24 | Traf3ip2 |  | Napsa |  |  |  |  | Gm11545 |  |  | Tmem245 | Fam120aos |
| Tmem120b | Ranbp10 | Cdpf1 |  | Hk3 |  |  |  |  | Rab19 |  |  | Cdc20 | Rab24 |
| Twist1 | Vdac3-ps1 | 3830406C13Rik |  | Fcho1 |  |  |  |  | Yod1 |  |  | Rcbtb1 | Apex1 |
| Rtn4rl2 | Tcta | Fam43a |  | Anxa13 |  |  |  |  | Gm4459 |  |  | Mrpl36 | Gvin1 |
| Artn | Pcca | Snhg6 |  | Slc16a10 |  |  |  |  | Prkab2 |  |  | Stag1 | Pyroxd2 |
| F2rl3 | Spryd7 | Gm11361 |  | H2-Q10 |  |  |  |  | B3galt4 |  |  | Timm50 | Zfp952 |
| Ripply3 | Mlycd | Zfp277 |  | Trim30b |  |  |  |  | Erich1 |  |  | Zc3h14 | Capn8 |
| Klrb1b | Fer1l6 | Casp8ap2 |  | Trpv2 |  |  |  |  | Tspyl2 |  |  | Pitpnb | Prickle3 |
| Lrrn4cl | Topors | Vezt |  | Tmem158 |  |  |  |  | 1500009L16Rik |  |  | Gnpat | Fbxl12 |
| Olfml1 | Rsf1 | Enc1 |  | Ifi204 |  |  |  |  | 4933427D14Rik |  |  | Mtmr2 | Ccdc84 |
| Cxcr3 | Atxn7 | Polr2j |  | Ifi209 |  |  |  |  | Pou6f1 |  |  | Ctbp2 | Wdr90 |
| H2-M2 | Btbd2 | P2ry2 |  | Ankrd33b |  |  |  |  | Cideb |  |  | Cmah | Tada2a |
| AU021092 | 1700025G04Rik | Prpsap2 |  | Rinl |  |  |  |  | Patz1 |  |  | Klf9 | 2610044O15Rik8 |
| Gm16685 | Gpatch2 | Smim11 |  | Hlx |  |  |  |  | Trmt10b |  |  | Mrps7 | Zfp280c |
| Sfrp5 | Pcgf5 | Robo2 |  | Cpxm2 |  |  |  |  | Tefm |  |  | Irf2bp1 | BC029722 |
| Irak1bp1 | Ppp1r8 | Coa6 |  | AI467606 |  |  |  |  | Cbx4 |  |  | Gmps | Sec61a2 |
| Sostdc1 | Usp33 | Ldlrad3 |  | Pou2f2 |  |  |  |  | Adck1 |  |  | Srp19 | Ofd1 |
| Fam216a | Aspn | Chm |  | Slc25a45 |  |  |  |  | Pot1b |  |  | Cnot2 | Znrf3 |
| Rpusd3 | Asap2 | Rpap1 |  | Lyz1 |  |  |  |  | Ubox5 |  |  | Brd7 | Gal3st2 |
| Mdk | Inafm1 | Caml |  | Tbx2 |  |  |  |  | Dnajc18 |  |  | Rfc1 | 4921524J17Rik |
| Ccl12 | Mon1b | Adprhl2 |  | Spp1 |  |  |  |  | Zswim5 |  |  | Bag3 | Slc22a21 |
| Prg2 | Cramp1l | Ring1 |  | Atg16l2 |  |  |  |  | Zbtb34 |  |  | Ccnb2 | Mroh2a |
| Ighv2-4 | Pign | Exd2 |  | Ccl25 |  |  |  |  | Strada |  |  | Mrpl3 | Bbc3 |
| Gm6658 | Cul5 | Ten1 |  | Dnm1 |  |  |  |  | Bckdha |  |  | Zfp652 | Primpol |
| Vtn | Rcan3 | Bnip1 |  | Dusp2 |  |  |  |  | Gas1 |  |  | Cdc5l | Eef1akmt2 |
| Gm49395 | Cdk2 | Fkrp |  | Arhgap25 |  |  |  |  | Echdc1 |  |  | Ypel3 | Msantd2 |
| mt-Tl1 | Gpr137b | Coa4 |  | Emcn |  |  |  |  | Rilp |  |  | Ube2g2 | Ccne2 |
| Gm7901 | Tuft1 | Isca2 |  | Zfp719 |  |  |  |  | Gm5529 |  |  | Med14 | D930016D06Rik |
| mt-Th | Pmm1 | Ebpl |  | Cxcl10 |  |  |  |  | Gm2962 |  |  | Polg | Zfp729b |
| Fbxo2 | Zfp518a | Mtpap |  | Chst12 |  |  |  |  | Ddah2 |  |  | Stk39 | Cep95 |
| Ighv1-77 | Sh2b3 | Peg13 |  | Taf7 |  |  |  |  | Tnfsf13 |  |  | Lsr | Tbc1d12 |
| Hist1h4k | Stat1 | Lrmp |  | Fam20c |  |  |  |  | Nrap |  |  | Timm17a | Gm5884 |
| mt-Ts2 | Cdkn2b | Zdhhc4 |  | Rap2b |  |  |  |  | Exoc3l2 |  |  | Dtymk | Zfp932 |
| Rpl7-ps7 | Pdk2 | Glod5 |  | Tnfrsf10b |  |  |  |  | Abi2 |  |  | Cdc16 | Dusp28 |
| Gm15590 | Spg11 | Ppp2r3c |  | Igfbp6 |  |  |  |  | Gm10705 |  |  | Brwd1 | B930095G15Rik |
| Gm15429 | Pdlim2 | Nfatc1 |  | Tnfrsf23 |  |  |  |  | Tmed10-ps |  |  | Stoml2 | Zc2hc1a |
| Gm7664 | Irgq | Klhl21 |  | Hspbap1 |  |  |  |  | Gpsm1 |  |  | Zfp710 | Clca3a2 |
| Igkj1 | Shisa2 | Ing5 |  | Clec5a |  |  |  |  | Hdac8 |  |  | Fam136a | Wfdc1 |
| Ighj3 | Igkv3-4 | Fam171a1 |  | Tarsl2 |  |  |  |  | A230050P20Rik |  |  | Tcerg1 | Hist2h2be |
| Gm2541 | Ctdp1 | Cdk5 |  | Robo4 |  |  |  |  | Ccp110 |  |  | Cdc37l1 | E2f1 |
| 9130015L21Rik | Gm13889 | Tango2 |  | Zfp472 |  |  |  |  | Dph1 |  |  | Ide | St7 |
| Fxyd7 | Igf2bp2 | Scn7a |  | Tm6sf2 |  |  |  |  | Zfp597 |  |  | Smc2 | Lhpp |
| Gm38317 | Tgfa | Uba6 |  | Fli1 |  |  |  |  | Ndufaf6 |  |  | Sec23ip | Ccdc77 |
| Gm40922 | Recql5 | Usp28 |  | Prkcb |  |  |  |  | Dusp19 |  |  | Dnajb9 | Zfp874a |
| Gm8034 | Rorc | Sinhcaf |  | Mllt11 |  |  |  |  | Prkar2b |  |  | Mrpl13 | Ccdc125 |
| Gm15610 | Zdhhc17 | Fam49a |  | Dmwd |  |  |  |  | Tmem107 |  |  | Strip1 | Rbm4b |
| Gm45380 | Vps39 | Zscan21 |  | Trbc2 |  |  |  |  | Grasp |  |  | Dnttip2 | Mbip |
| Gm19774 | Slc17a4 | Rnf138 |  | Zfp52 |  |  |  |  | Fkbp7 |  |  | Tmem189 | Spice1 |
| Mir6973a | Morc2a | Rpp25l |  | Pilra |  |  |  |  | Taf1b |  |  | Mrpl15 | Slc39a7 |
|  | Smoc2 | Atp7a |  | Mctp1 |  |  |  |  | Omp |  |  | Mrpl34 | Pdzd7 |
|  | Pi4kb | Med30 |  | Abtb2 |  |  |  |  | Gm1821 |  |  | Dnajc2 | Zfp579 |
|  | Acvr2a | Mplkip |  | Igkv8-28 |  |  |  |  | Slc38a6 |  |  | Mpp6 | Zfp688 |
|  | Ppfia3 | Lcmt1 |  | Il21r |  |  |  |  | Snap25 |  |  | Cenpa | Cep152 |
|  | Slfn5 | Dxo |  | C030006K11Rik |  |  |  |  | Glt28d2 |  |  | Psmd5 | Proser2 |
|  | Rad50 | Cry1 |  | Rhov |  |  |  |  | Insl6 |  |  | Cks2 | 1190007I07Rik |
|  | Cachd1 | Lage3 |  | Inhbb |  |  |  |  | Nsun6 |  |  | Supt16 | 1300002E11Rik |
|  | Gid4 | Pou2f1 |  | H2-Q5 |  |  |  |  | Cited4 |  |  | Chmp7 | Fancc |
|  | Rsu1 | Acer2 |  | Cd300a |  |  |  |  | Elk1 |  |  | Abcf2 | Large2 |
|  | Ddx58 | Diaph2 |  | Creb3l3 |  |  |  |  | Pars2 |  |  | AW549877 | Nmnat3 |
|  | Ptdss2 | Ankrd49 |  | Ptpn7 |  |  |  |  | 9930111J21Rik2 |  |  | Tcof1 | Snhg4 |
|  | Galnt5 | Eef2kmt |  | D16Ertd472e |  |  |  |  | Plp1 |  |  | Ufsp2 | Oxld1 |
|  | Ptprn2 | Vps37b |  | Tnfaip8l2 |  |  |  |  | Trdmt1 |  |  | Otud6b | Nat6 |
|  | Slc22a5 | Epb41l4aos |  | Cnnm2 |  |  |  |  | Kank3 |  |  | Incenp | Pla2g6 |
|  | Fam149b | Cln8 |  | Rgs12 |  |  |  |  | Gm8566 |  |  | Hsph1 | Zfp60 |
|  | Fam110c | 1810055G02Rik |  | Gpr132 |  |  |  |  | Gm9917 |  |  | Birc5 | Zfp839 |
|  | Ifi35 | Dnttip1 |  | Arid3b |  |  |  |  | Gm8121 |  |  | Bud31 | Bvht |
|  | Nadk2 | Lancl1 |  | P2ry13 |  |  |  |  | Clcn6 |  |  | Nudcd2 | Rbak |
|  | Mertk | Mier2 |  | Rab44 |  |  |  |  | Jam3 |  |  | Cbx3 | Neil1 |
|  | Igdcc4 | Gm12338 |  | Praf2 |  |  |  |  | Sncg |  |  | Baz2b | Zfp182 |
|  | A4gnt | Josd2 |  | Stx2 |  |  |  |  | Slc26a11 |  |  | Mrpl37 | Izumo4 |
|  | Pisd | Mmgt2 |  | Gngt2 |  |  |  |  | Aldh3b2 |  |  | Mrpl4 | Abhd10 |
|  | Parp3 | Pdp2 |  | Tnfrsf11b |  |  |  |  | Gm14253 |  |  | Tpx2 | Clca3a1 |
|  | Cdc25b | Pam16 |  | Klhl6 |  |  |  |  | Bglap3 |  |  | Sf3a3 | Rbm12b2 |
|  | Lmf2 | Rab11fip3 |  | Rin1 |  |  |  |  | Prr3 |  |  | Dcaf13 | Setd4 |
|  | Shb | 1110038F14Rik |  | N4bp2l1 |  |  |  |  | Pex11g |  |  | 2310011J03Rik | Rad1 |
|  | Tmem106a | Slc30a6 |  | Cd244a |  |  |  |  | Cldn5 |  |  | Ints1 | Ift88 |
|  | Steap2 | Ap5s1 |  | Ubash3b |  |  |  |  | Zfp971 |  |  | Utp6 | Zfp799 |
|  | Uap1l1 | Tsen54 |  | Grap2 |  |  |  |  | Rnf8 |  |  | Itsn1 | Gm8822 |
|  | Cobll1 | Apopt1 |  | Parvg |  |  |  |  | Ngfr |  |  | Mcm2 | Zfp788 |
|  | Pxmp4 | Aars2 |  | 2310015A10Rik |  |  |  |  | Senp8 |  |  | Cnot7 | Harbi1 |
|  | Vopp1 | Cracr2b |  | Fam189b |  |  |  |  | Grcc10 |  |  | Btaf1 | Gm12940 |
|  | B3gnt6 | Timm21 |  | Il5ra |  |  |  |  | Cox17 |  |  | Frmd8 | Gm14410 |
|  | Cbfa2t3 | Dcps |  | Jag2 |  |  |  |  | Rilpl1 |  |  | Thop1 | Taf4b |
|  | Wdr20 | 4930402H24Rik |  | Bmp8b |  |  |  |  | Atoh8 |  |  | Smarcb1 | Kctd6 |
|  | 1190002N15Rik | Crtc3 |  | Nab2 |  |  |  |  | Kctd15 |  |  | Ythdf2 | Slc25a42 |
|  | Usp36 | Tmem219 |  | Madcam1 |  |  |  |  | Hmgn3 |  |  | Kalrn | Gal3st2c |
|  | Stc2 | Ccdc174 |  | Rasgrp4 |  |  |  |  | 1810034E14Rik |  |  | Cdk2ap1 | 1600020E01Rik |
|  | Ppp2r3d | Rpp21 |  | Ajuba |  |  |  |  | Pisd-ps1 |  |  | Rps6kb1 | Mill2 |
|  | Ceacam2 | Enah |  | Grap |  |  |  |  | Eid2b |  |  | Vegfa | Xrcc4 |
|  | Boc | Best2 |  | Lonrf1 |  |  |  |  | Gm14121 |  |  | Pak1ip1 | Ppm1k |
|  | Slc25a35 | Dglucy |  | Smoc1 |  |  |  |  | Gm9826 |  |  | Uhrf1 | Zfp930 |
|  | Zfp644 | Mycl |  | Angel1 |  |  |  |  | Tnfsf12 |  |  | Unc45a | Polg2 |
|  | Hsdl2 | Tmem110 |  | Eepd1 |  |  |  |  | Eif4ebp3 |  |  | Gm5518 | Gm15682 |
|  | Epha1 | Lxn |  | Ier5l |  |  |  |  | Scg5 |  |  | Rnf167 | Taf1a |
|  | Ptprr | Wdr83 |  | Pcnx4 |  |  |  |  | Mapk12 |  |  | Trappc13 | Zfp958 |
|  | Slc17a5 | Vav3 |  | Slc6a19 |  |  |  |  | Rem1 |  |  | Mbd6 | Ptpru |
|  | Ccdc68 | Tyw1 |  | Sh2b2 |  |  |  |  | Xkr9 |  |  | Shtn1 | Bckdhb |
|  | Tpk1 | Gstt1 |  | Btk |  |  |  |  | Gm26617 |  |  | Eif4enif1 | Asrgl1 |
|  | Fuom | Galt |  | Ptger3 |  |  |  |  | Ap3s1-ps1 |  |  | Pcnx | Hps6 |
|  | Gnpda1 | 1700037H04Rik |  | Nod2 |  |  |  |  | Gm26782 |  |  | Tmem120a | mt-Tc |
|  | Nob1 | 2310039H08Rik |  | Meox1 |  |  |  |  | Gm14038 |  |  | Chordc1 | Trim68 |
|  | Agl | Nlk |  | Bbs7 |  |  |  |  | Gap43 |  |  | Llph | Rtkn |
|  | Slc51b | Ino80b |  | Lat |  |  |  |  | Mfsd4b4 |  |  | Ampd3 | Kif12 |
|  | Epc1 | Impa2 |  | Fam78a |  |  |  |  | Tubb4a |  |  | Acy1 | Eri2 |
|  | Zbtb43 | Rapgef2 |  | Def6 |  |  |  |  | Krt8-ps |  |  | Rrm2 | Vstm5 |
|  | Tmem56 | Gm11273 |  | Lgals2 |  |  |  |  | Plppr2 |  |  | Fip1l1 | Sft2d3 |
|  | Clybl | Hddc2 |  | Evi2a |  |  |  |  | Neurod1 |  |  | Cc2d1a | 2810402E24Rik |
|  | Mctp2 | Gpatch1 |  | Adrb2 |  |  |  |  | Gm17275 |  |  | Ppil1 | Tfcp2 |
|  | Axin2 | Tmem9 |  | Kcnj2 |  |  |  |  | Aoc2 |  |  | St6galnac4 | D3Ertd751e |
|  | Plcd1 | Rpl10a-ps1 |  | Gm14117 |  |  |  |  | Gm10704 |  |  | Aqr | Zfp612 |
|  | Ctso | Rint1 |  | Mmp25 |  |  |  |  | Gm9780 |  |  | Fam98a | Phf7 |
|  | Paqr5 | Arl2 |  | Traf3ip3 |  |  |  |  | Gm12231 |  |  | Vcp-rs | Gm17066 |
|  | H2-T3 | Polb |  | Adgrg3 |  |  |  |  | Setmar |  |  | Mrps5 | Zfp870 |
|  | Zfp422 | Borcs8 |  | Ccr7 |  |  |  |  | Porcn |  |  | Slbp | 2010016I18Rik |
|  | Cnksr1 | Smim12 |  | Aspa |  |  |  |  | Gm8325 |  |  | Wrnip1 | A530020G20Rik |
|  | Ube2w | Nudt2 |  | Cd8a |  |  |  |  | Pxmp2 |  |  | Fam192a | Rtn4rl1 |
|  | Prkd2 | Erc1 |  | Pacsin3 |  |  |  |  | Dync2li1 |  |  | Cnot9 | Zfp933 |
|  | Trim36 | Ube2o |  | Nfe2 |  |  |  |  | Gm18541 |  |  | Hbs1l | Zbtb48 |
|  | Ppp1r21 | Mtfmt |  | Msln |  |  |  |  | Slc29a2 |  |  | Sf3b4 | Smyd3 |
|  | Rab4a | Kyat1 |  | Il7r |  |  |  |  | AW011738 |  |  | Nup205 | Helq |
|  | Nup210 | Rps19-ps6 |  | Grrp1 |  |  |  |  | Bbs12 |  |  | Cad | Rab3a |
|  | Slc10a5 | Dnal4 |  | Agap2 |  |  |  |  | Gm10080 |  |  | Mars | Tlcd1 |
|  | Miga2 | Wdr7 |  | Treml2 |  |  |  |  | 2210011C24Rik |  |  | Pbx1 | Rad52 |
|  | Auts2 | Trim46 |  | Padi4 |  |  |  |  | Ndufa4l2 |  |  | Ift20 | Atat1 |
|  | Oma1 | Zfp444 |  | Rnf125 |  |  |  |  | Gm48496 |  |  | Lig1 | Gnal |
|  | Slc7a5 | Mrps18c |  | Pld2 |  |  |  |  | Fbxo17 |  |  | Tln2 | Hemk1 |
|  | Pigx | Fbxw9 |  | Camkmt |  |  |  |  | Apoo-ps |  |  | Armc1 | 4632404H12Rik |
|  | Ccnyl1 | Def8 |  | Trappc2 |  |  |  |  | Gm16089 |  |  | Gtpbp4 | Acad12 |
|  | P2ry6 | Adcy5 |  | Camkk1 |  |  |  |  | Tbxa2r |  |  | Cpsf3 | Nphp3 |
|  | Acer1 | 3110040N11Rik |  | Paqr7 |  |  |  |  | Rbp7 |  |  | Pum3 | Dusp23 |
|  | Pold4 | Scnm1 |  | Chst1 |  |  |  |  | Snx32 |  |  | Gcnt1 | Fahd2a |
|  | Nup188 | Ms4a12 |  | Trac |  |  |  |  | Gm10297 |  |  | Atp6v0c | Zfp760 |
|  | Creb3l4 | Dapk1 |  | Trp53i13 |  |  |  |  | Gm6395 |  |  | Elp3 | Gm10093 |
|  | Mfhas1 | Coq6 |  | Lin52 |  |  |  |  | Zbtb3 |  |  | Kank1 | Gm46430 |
|  | Ciz1 | Ntpcr |  | Rgs14 |  |  |  |  | Gm14388 |  |  | Rfc2 | Ccdc163 |
|  | Eif1b | Zfp275 |  | Btnl2 |  |  |  |  | Ndufb4c |  |  | Lemd2 | Tmem35b |
|  | Rdh14 | Abcc5 |  | Arhgef15 |  |  |  |  | Reep2 |  |  | Ubn2 | B4galnt4 |
|  | Ston2 | Pgm2l1 |  | Arsg |  |  |  |  | Igkj5 |  |  | Eif1ad | Zfp157 |
|  | Aldh6a1 | Rpl19-ps11 |  | AI662270 |  |  |  |  | Gm10145 |  |  | Ripk3 | Nkiras1 |
|  | Neurl1b | Zfp871 |  | Gpatch3 |  |  |  |  | Gm10382 |  |  | Tk1 | B3gntl1 |
|  | Cyb561d1 | Gm8430 |  | Alox5 |  |  |  |  | Zfp580 |  |  | Ern1 | Gm12966 |
|  | Tmem18 | Mettl23 |  | Slc27a2 |  |  |  |  | Gm37963 |  |  | Creg1 | Abo |
|  | Lpcat1 | Nudt16l1 |  | Enpp3 |  |  |  |  | Gm4705 |  |  | Akap7 | Sirt5 |
|  | Tmem39a | Cars2 |  | Rasip1 |  |  |  |  | A530030E21Rik |  |  | Tnip3 | Hspb6 |
|  | Ces1g | Cyp39a1 |  | Adm |  |  |  |  | Igkj4 |  |  | Sdc2 | Atn1 |
|  | Map3k20 | 4931406P16Rik |  | Krt36 |  |  |  |  | Gm23935 |  |  | Hdac5 | Wdr47 |
|  | Clec16a | Rft1 |  | Gal |  |  |  |  | BC028528 |  |  | Gch1 | Gm27149 |
|  | Crbn | Cbr2 |  | Iffo1 |  |  |  |  | Gm5457 |  |  | Kif2a | Gm5617 |
|  | Fam111a | Ccdc61 |  | Mogat2 |  |  |  |  | Gm10736 |  |  | Ruvbl2 | Abraxas1 |
|  | Uty | Hspa1b |  | Fosl1 |  |  |  |  | Aard |  |  | Wiz | D930048N14Rik |
|  | Bmp2k | Mynn |  | AI504432 |  |  |  |  | Gm48194 |  |  | Mcm4 | Ccdc116 |
|  | Rhbdd1 | Hdhd5 |  | Adssl1 |  |  |  |  | Gm13203 |  |  | Phc3 | Rfesd |
|  | Dnajc16 | Zfp568 |  | Ppp1r3d |  |  |  |  | Igkj3 |  |  | Pkp1 | Asnsd1 |
|  | Tpsg1 | Magohb |  | Dubr |  |  |  |  | Ltc4s |  |  | Usp10 | Bcl2l12 |
|  | Map4k5 | Khdrbs3 |  | Batf |  |  |  |  | Igkj2 |  |  | Ino80d | Zfp764 |
|  | Traf2 | Gm13456 |  | Cd86 |  |  |  |  | Rap1gapos |  |  | Atg101 | E430018J23Rik |
|  | Usp45 | Gpn2 |  | Ms4a4b |  |  |  |  | Gm14321 |  |  | Mrps26 | Gm28439 |
|  | Dtnbp1 | Gpatch11 |  | I830077J02Rik |  |  |  |  | Neurl2 |  |  | Memo1 | Gm15446 |
|  | Pphln1 | Gm11703 |  | 2010106E10Rik |  |  |  |  | Gm29787 |  |  | Cdk19 | Zfp862-ps |
|  | Ipo13 | Dmap1 |  | Lct |  |  |  |  | Rtl10 |  |  | Snrpa | Sirt4 |
|  | Isoc2a | Zfp771 |  | Mdm1 |  |  |  |  | Gm5560 |  |  | Wdr36 | Vegfc |
|  | Phf8 | Zfpl1 |  | Hspa1a |  |  |  |  | Gm19366 |  |  | Taf5l | AW554918 |
|  | Bche | Nit2 |  | Tmem132a |  |  |  |  | Gm14286 |  |  | Snrnp40 | Zfp959 |
|  | D430042O09Rik | Pigyl |  | 4930503L19Rik |  |  |  |  | 9230111E07Rik |  |  | Ccdc34 | Gm10516 |
|  | 2810459M11Rik | Nsmce2 |  | Klra2 |  |  |  |  | Gm12345 |  |  | Asns | Rsad1 |
|  | Enpp4 | D230025D16Rik |  | Slfn8 |  |  |  |  | Mrpl23-ps1 |  |  | Thra | Gm609 |
|  | Snx15 | Cby1 |  | Nr4a3 |  |  |  |  | Gm15344 |  |  | Adsl | Tmem218 |
|  | Lrrk2 | Ift43 |  | Trbc1 |  |  |  |  | Gm44199 |  |  | Arhgef10l | Tmem39b |
|  | Tbc1d22b | Rfxap |  | Angpt2 |  |  |  |  | Glud-ps |  |  | Ubfd1 | BC024978 |
|  | Casp9 | Ctns |  | Zfpm1 |  |  |  |  | Atp5l2-ps |  |  | Bop1 | Mapk1ip1 |
|  | Tent4a | Atxn7l1 |  | 4833407H14Rik |  |  |  |  | Gm14541 |  |  | Fubp3 | Ccdc130 |
|  | Phlda1 | Commd1 |  | Morc4 |  |  |  |  | Ndufs6b |  |  | Parl | Gm9860 |
|  | Tmem260 | Tmem192 |  | Aplnr |  |  |  |  | Lsmem2 |  |  | Cacnb3 | Mks1 |
|  | Plcb4 | Ppp1r35 |  | Stfa2 |  |  |  |  | Gm13268 |  |  | Wdr5 | Rnf32 |
|  | Pex16 | Homer1 |  | 6530402F18Rik |  |  |  |  | Gm6020 |  |  | Dpagt1 | Zfp956 |
|  | Oas2 | Rps10-ps1 |  | Soat2 |  |  |  |  | Gm22753 |  |  | Ikbkg | Spaca4 |
|  | Dio1 | N6amt1 |  | Cyp2b10 |  |  |  |  | Gm16161 |  |  | Rpia | Gm5182 |
|  | E2f5 | Ovol2 |  | Ltb4r1 |  |  |  |  | Gm26387 |  |  | Cul2 | 1700001L05Rik |
|  | Ikbip | Tgs1 |  | Tm6sf1 |  |  |  |  | Snord49a |  |  | Gpaa1 | Bcdin3d |
|  | Pard3b | Cox7a1 |  | Adh6a |  |  |  |  | Eif3s6-ps2 |  |  | Pcsk9 | Nemp2 |
|  | Mospd2 | Churc1 |  | Tm4sf5 |  |  |  |  | Gm22935 |  |  | Pole3 | 3-Mar |
|  | Nsmce3 | Rpusd4 |  | Olr1 |  |  |  |  | Gm22362 |  |  | Tapt1 | Zfp825 |
|  | Aadac | Slc1a1 |  | Sec14l2 |  |  |  |  | Snora21 |  |  | Snrpc | Grik5 |
|  | Sdr42e1 | Rsph3b |  | Nos3 |  |  |  |  | Mir339 |  |  | Cdc73 | Ddb2 |
|  | Ube2l6 | Alkbh6 |  | Slc25a48 |  |  |  |  | Gm24644 |  |  | Ttc13 | Nek3 |
|  | Abca12 | Pla2g4f |  | Gm9392 |  |  |  |  | Mir7679 |  |  | Shmt2 | Rab11b-ps2 |
|  | Slc25a34 | Qrsl1 |  | Snai2 |  |  |  |  | Mir3091 |  |  | Dus1l | Gm12183 |
|  | Sgsm2 | Txnl4a |  | Apoc3 |  |  |  |  | Gm26202 |  |  | Tppp3 | AW146154 |
|  | Elmod2 | Nkap |  | Adcy4 |  |  |  |  | Gm22933 |  |  | Tbc1d2 | BC002059 |
|  | Iqsec2 | Zdhhc12 |  | Rasgrp1 |  |  |  |  |  |  |  | Eya3 | Zfp775 |
|  | Tmem184a | Pex14 |  | Msantd3 |  |  |  |  |  |  |  | Mrpl45 | Kifc2 |
|  | Abhd14b | Fitm2 |  | Adamtsl5 |  |  |  |  |  |  |  | Gramd4 | Naip3-ps1 |
|  | Apol9a | Tec |  | Tgm1 |  |  |  |  |  |  |  | Mad2l1 | Ankle1 |
|  | Cpsf4 | Slc7a6 |  | Gm6377 |  |  |  |  |  |  |  | Ppp1r7 | Mpg |
|  | Slc2a13 | Rpl34-ps1 |  | Nipa1 |  |  |  |  |  |  |  | Mmgt1 | Zfp983 |
|  | Prss23 | Ss18l2 |  | Relt |  |  |  |  |  |  |  | Ddx27 | Ttll10 |
|  | Akap10 | 1500011K16Rik |  | Mfng |  |  |  |  |  |  |  | Tmub2 | Dph7 |
|  | Ano7 | Wdsub1 |  | Havcr2 |  |  |  |  |  |  |  | Drosha | Cluap1 |
|  | Fam83b | Faap20 |  | Cd40 |  |  |  |  |  |  |  | Pold3 | Cryzl2 |
|  | Fmo5 | Tab3 |  | Cbx8 |  |  |  |  |  |  |  | Dhrs1 | Zfp27 |
|  | Hkdc1 | Polr3a |  | Cd80 |  |  |  |  |  |  |  | Prmt5 | Gm21781 |
|  | Ffar2 | Nrde2 |  | Ifi211 |  |  |  |  |  |  |  | Dut | Npy1r |
|  | Tnfsf10 | Ddhd1 |  | Ltf |  |  |  |  |  |  |  | Fbxw8 | Fuz |
|  | Fam98c | R3hcc1 |  | Mapk11 |  |  |  |  |  |  |  | Pepd | Tbc1d19 |
|  | Gbp3 | Dnase2a |  | Cdk5r1 |  |  |  |  |  |  |  | Arid4b | Fmo1 |
|  | Abcc4 | Lipo3 |  | Slc6a20a |  |  |  |  |  |  |  | Mkl2 | Gm12411 |
|  | Dcaf6 | Zfp46 |  | Zap70 |  |  |  |  |  |  |  | Ptch1 | Tmem267 |
|  | Adtrp | Eral1 |  | Gimap5 |  |  |  |  |  |  |  | Trim12c | 1810032O08Rik |
|  | Arhgef19 | Spef1 |  | Gm12854 |  |  |  |  |  |  |  | Dapk2 | Foxq1 |
|  | Gpr160 | Dclk1 |  | Ldb2 |  |  |  |  |  |  |  | Kif23 | Bbs2 |
|  | Helb | Pik3cb |  | Cnr2 |  |  |  |  |  |  |  | Tyms | Tmem206 |
|  | Fbxo21 | Map1b |  | Cd3d |  |  |  |  |  |  |  | Gnl3 | Gm44223 |
|  | Col8a1 | Gm9843 |  | Usp35 |  |  |  |  |  |  |  | Hmcn2 | Rpgr |
|  | Baiap2 | Hps1 |  | Gm37691 |  |  |  |  |  |  |  | Cdc42bpa | Edn2 |
|  | Zfyve21 | Dtd2 |  | Ap3m2 |  |  |  |  |  |  |  | Arl6ip4 | Syn2 |
|  | Wnk4 | Hscb |  | Nt5dc2 |  |  |  |  |  |  |  | Mepce | Fn3krp |
|  | Mfsd10 | Pdcd2l |  | Bcl2a1d |  |  |  |  |  |  |  | Tor2a | Phf19 |
|  | Rasgef1b | Col6a4 |  | Cd6 |  |  |  |  |  |  |  | Csnk2a2 | Zfp867 |
|  | Hint3 | Tmem106c |  | Itgb2l |  |  |  |  |  |  |  | Gart | Gm7694 |
|  | Nat2 | Acd |  | Adora2a |  |  |  |  |  |  |  | Vps52 | Klf12 |
|  | Tmem173 | Pramef8 |  | Rnd2 |  |  |  |  |  |  |  | Tmem41b | 2700097O09Rik |
|  | Nup54 | Snhg8 |  | Lst1 |  |  |  |  |  |  |  | E2f2 | Poli |
|  | Pskh1 | Ppt2 |  | Mrgpra2b |  |  |  |  |  |  |  | Cetn3 | Diablo |
|  | Ppp6r2 | Nat9 |  | Prss22 |  |  |  |  |  |  |  | Wdr18 | Gm37261 |
|  | Homer2 | Megf9 |  | Dok2 |  |  |  |  |  |  |  | Trim44 | Serf1 |
|  | Arntl | Gucy1b1 |  | Tlr6 |  |  |  |  |  |  |  | Tacc3 | Zfp595 |
|  | Stk3 | Mpv17 |  | Ms4a10 |  |  |  |  |  |  |  | Tmem109 | Slc16a13 |
|  | Ly75 | Sgcb |  | Nmrk1 |  |  |  |  |  |  |  | Dennd6a | Wdr5b |
|  | Nudt16 | Atp6v0e2 |  | Fam217b |  |  |  |  |  |  |  | Clk3 | Depdc1b |
|  | Rsrc1 | Fam58b |  | Ttll1 |  |  |  |  |  |  |  | Polr2c | Eme2 |
|  | Nkapd1 | C2cd2 |  | S1pr4 |  |  |  |  |  |  |  | Mrps15 | Ufsp1 |
|  | Kdm4c | Alkbh7 |  | Rasl11a |  |  |  |  |  |  |  | Hmgb3 | Gm8494 |
|  | L2hgdh | Pdzrn3 |  | Ift57 |  |  |  |  |  |  |  | Crot | Tatdn3 |
|  | Usp40 | Rab13 |  | Rtl8a |  |  |  |  |  |  |  | Pno1 | Mir17hg |
|  | Fam189a2 | Zfp407 |  | Ms4a4c |  |  |  |  |  |  |  | Nfyc | Dennd6b |
|  | Zfyve16 | Pet100 |  | Vamp5 |  |  |  |  |  |  |  | Pnpt1 | 9130019O22Rik |
|  | Atp8a2 | Kin |  | Clcf1 |  |  |  |  |  |  |  | Bms1 | AI480526 |
|  | Wdr44 | Smim8 |  | Krt14 |  |  |  |  |  |  |  | Kdm5b | Zfp59 |
|  | Asb6 | Rsbn1 |  | Marco |  |  |  |  |  |  |  | Agap1 | Zswim3 |
|  | Hsd3b2 | Nepro |  | Olfm4 |  |  |  |  |  |  |  | Nelfcd | Bbs4 |
|  | E2f6 | Gm10335 |  | Cd300lb |  |  |  |  |  |  |  | Prdx4 | Gm17491 |
|  | Mfap3l | Micall1 |  | Slc25a29 |  |  |  |  |  |  |  | Gorasp1 | 2810410L24Rik |
|  | Shroom2 | Sirt6 |  | Tnfaip6 |  |  |  |  |  |  |  | Dpy30 | AC098880.2 |
|  | Mccc1 | Kat8 |  | Arl5c |  |  |  |  |  |  |  | Zfp260 | mt-Tn |
|  | Kctd9 | Dexi |  | Klhl8 |  |  |  |  |  |  |  | Ppm1h | 2310007B03Rik |
|  | Ppargc1a | Igkv16-104 |  | Hcst |  |  |  |  |  |  |  | Commd3 | Gm15542 |
|  | Zfp768 | Dcp1b |  | Gm42547 |  |  |  |  |  |  |  | Mtss1 | Gm15947 |
|  | Clec14a | Alkbh3 |  | Akr1b7 |  |  |  |  |  |  |  | Sephs1 | Plppr3 |
|  | Pip4k2b | Itfg2 |  | Ccdc126 |  |  |  |  |  |  |  | Med8 | Gmpr2 |
|  | Gpt2 | Cryz |  | Acsbg1 |  |  |  |  |  |  |  | Nelfe | 3110056K07Rik |
|  | Sult1c2 | 1110059G10Rik |  | Tnfrsf9 |  |  |  |  |  |  |  | Sart3 | Zc3h6 |
|  | Slc22a1 | Rpsa-ps2 |  | Fhl3 |  |  |  |  |  |  |  | Ilf3 | 9130023H24Rik |
|  | Uevld | Gm10154 |  | Trim13 |  |  |  |  |  |  |  | Sertad1 | Mir196a-1 |
|  | Sh3gl2 | Nudt1 |  | Gm43737 |  |  |  |  |  |  |  | Eif2b2 | Gm12454 |
|  | Pttg1 | Mettl17 |  | Gfra2 |  |  |  |  |  |  |  | Utp3 | Psrc1 |
|  | Utp14b | Pkmyt1 |  | Mdfi |  |  |  |  |  |  |  | Eri1 | Atg10 |
|  | E2f3 | Pdcd5-ps |  | Fignl2 |  |  |  |  |  |  |  | Htatsf1 | Cd200r2 |
|  | Dgkq | Gm6169 |  | St3gal5 |  |  |  |  |  |  |  | Bud23 | Zfp772 |
|  | P2ry1 | Det1 |  | Tyw3 |  |  |  |  |  |  |  | Vamp7 | Zfp938 |
|  | Pex11a | Lipt2 |  | Igkv14-126 |  |  |  |  |  |  |  | Cuta | Platr25 |
|  | Polr3f | Pla2g12a |  | BC100530 |  |  |  |  |  |  |  | Magoh | Prdm9 |
|  | Dhx33 | Taf1c |  | Tmem71 |  |  |  |  |  |  |  | Knop1 | Zscan12 |
|  | Socs5 | Zfp623 |  | Card9 |  |  |  |  |  |  |  | Cactin | Zfp976 |
|  | Stambpl1 | Asf1a |  | Il27ra |  |  |  |  |  |  |  | Exosc10 | AC129328.1 |
|  | Ppfibp2 | Uros |  | Cib2 |  |  |  |  |  |  |  | Sh3bp5l | Zfp970 |
|  | Zfp282 | Usp31 |  | Tctex1d2 |  |  |  |  |  |  |  | Ube2e3 | Pdgfd |
|  | Cmpk2 | Rnf113a2 |  | Cd5 |  |  |  |  |  |  |  | Tgif1 | Rexo5 |
|  | Exph5 | Pitpnm2 |  | Adora2b |  |  |  |  |  |  |  | Eed | Zfp653 |
|  | Igkv6-25 | Tmem101 |  | C1rl |  |  |  |  |  |  |  | Hmbs | Ccdc191 |
|  | Sh3tc1 | Pde4d |  | Ccl22 |  |  |  |  |  |  |  | Arhgap11a | Bnip3l-ps |
|  | Pus7 | Pcgf6 |  | Tmem88 |  |  |  |  |  |  |  | Gtf3c6 | Sms-ps |
|  | Slc3a1 | Spg20 |  | Igkv4-58 |  |  |  |  |  |  |  | Trappc4 | Spsb3 |
|  | Tmem230 | Gatb |  | Tm4sf4 |  |  |  |  |  |  |  | Crls1 | 4933439C10Rik |
|  | Dpy19l1 | Grk3 |  | Stfa1 |  |  |  |  |  |  |  | Ect2 | Gm16536 |
|  | Oraov1 | Wdyhv1 |  | Ighv1-5 |  |  |  |  |  |  |  | Tmem252 | 1110051M20Rik |
|  | Pigg | Ccdc122 |  | Adgrg5 |  |  |  |  |  |  |  | Mecp2 | Mtcp1 |
|  | Atg4a | Zc3h12d |  | Sh3bp1 |  |  |  |  |  |  |  | Ppp2r1b | Smyd4 |
|  | Cnksr3 | Scrn3 |  | Entpd3 |  |  |  |  |  |  |  | Btbd3 | Zfp40 |
|  | Bahcc1 | Sdsl |  | Trem3 |  |  |  |  |  |  |  | Zcchc8 | A730017L22Rik |
|  | Agfg2 | Pex11b |  | Sirpb1c |  |  |  |  |  |  |  | Ppid | Myl7 |
|  | Hnf4g | Fam118a |  | Stx1a |  |  |  |  |  |  |  | Rbm27 | Nbdy |
|  | Reps1 | Nudt13 |  | Tmem86b |  |  |  |  |  |  |  | Gm12960 | Sdhaf3 |
|  | Prkce | Tmed8 |  | Slc18a2 |  |  |  |  |  |  |  | Ncbp2 | Zfp991 |
|  | Hcfc2 | Gm4294 |  | Treh |  |  |  |  |  |  |  | Snrpb2 | Mterf2 |
|  | Aamdc | Slc5a6 |  | Gpr18 |  |  |  |  |  |  |  | Klhdc2 | AI606181 |
|  | Mettl6 | Thnsl2 |  | Fam19a5 |  |  |  |  |  |  |  | Herpud2 | Glmn |
|  | Tbc1d10a | Tmem69 |  | A530040E14Rik |  |  |  |  |  |  |  | Ambra1 | Zfp763 |
|  | Slc41a2 | Dnajc30 |  | Mex3b |  |  |  |  |  |  |  | Mvk | Gm4673 |
|  | Apol7a | Saysd1 |  | Slc2a5 |  |  |  |  |  |  |  | Ftsj3 | Pnmal2 |
|  | Cables1 | Rab29 |  | Gpr84 |  |  |  |  |  |  |  | Dkc1 | Slc9a5 |
|  | Gm6548 | Chchd5 |  | Gm19951 |  |  |  |  |  |  |  | Kansl2 | Tmem42 |
|  | Hnf1a | Pter |  | Il10 |  |  |  |  |  |  |  | Cdca8 | Zfp119a |
|  | Poglut1 | Zfp637 |  | Cd8b1 |  |  |  |  |  |  |  | Tmem268 | Gm16136 |
|  | D17H6S53E | Pstk |  | Rdh7 |  |  |  |  |  |  |  | Vps13a | Gli1 |
|  | Rnf152 | Tmeff1 |  | Gm8399 |  |  |  |  |  |  |  | Rbmxl1 | B9d1 |
|  | Flrt3 | Psmg4 |  | Gm15832 |  |  |  |  |  |  |  | Anapc4 | Cep41 |
|  | Amot | Xpnpep3 |  | Sgtb |  |  |  |  |  |  |  | Gtf2h5 | Zfp229 |
|  | Ikbkap | Phka1 |  | D430020J02Rik |  |  |  |  |  |  |  | Fam104a | Kbtbd4 |
|  | Sirt1 | Paxx |  | Bmx |  |  |  |  |  |  |  | Ercc3 | Fmo4 |
|  | Il22ra1 | AC115954.1 |  | Tnfsf14 |  |  |  |  |  |  |  | Nabp2 | AC126457.1 |
|  | Zfyve9 | Smim19 |  | Shisa4 |  |  |  |  |  |  |  | Ass1 | Fam161a |
|  | Snapc1 | Rpl21-ps8 |  | Ccm2l |  |  |  |  |  |  |  | Wdr61 | Oas1c |
|  | Vmac | Aldh4a1 |  | Castor1 |  |  |  |  |  |  |  | Thumpd1 | Gm37969 |
|  | Lzic | Polr2k |  | Pced1b |  |  |  |  |  |  |  | Gnl3l | 2010001A14Rik |
|  | Reps2 | Ap4e1 |  | Mir142hg |  |  |  |  |  |  |  | Crim1 | Zfp97 |
|  | Tex10 | Mtg2 |  | Pilrb2 |  |  |  |  |  |  |  | Zdhhc6 | Mfsd3 |
|  | Ttc33 | Srd5a2 |  | Gm14548 |  |  |  |  |  |  |  | Nek2 | Lyplal1 |
|  | Kif1bp | Tsr3 |  | Susd3 |  |  |  |  |  |  |  | Tob2 | Dusp12 |
|  | Phf11d | Dpp7 |  | Ebi3 |  |  |  |  |  |  |  | Alad | Timm10b |
|  | Fam83f | 2310030G06Rik |  | Gm47283 |  |  |  |  |  |  |  | Slc11a2 | Cck |
|  | Plscr3 | Ap4s1 |  | Cyp3a25 |  |  |  |  |  |  |  | Abcf3 | Gm21399 |
|  | Cdk18 | Tyro3 |  | Gm30414 |  |  |  |  |  |  |  | Gcsh | Eldr |
|  | Itga7 | Rpl17-ps5 |  | Purg |  |  |  |  |  |  |  | Dhx30 | Zfp61 |
|  | Tmem139 | Rnaseh1 |  | Prr7 |  |  |  |  |  |  |  | Ddx19a | Casq2 |
|  | Yif1b | Sh3rf2 |  | Cyp2c66 |  |  |  |  |  |  |  | Mrpl11 | Gm15417 |
|  | Fgfr4 | 9430038I01Rik |  | Gm17586 |  |  |  |  |  |  |  | Sestd1 | Gm12258 |
|  | Rcl1 | Abhd14a |  | Mreg |  |  |  |  |  |  |  | Mindy3 | Armcx5 |
|  | Samd12 | Fdxr |  | Gm5150 |  |  |  |  |  |  |  | Rc3h2 | Zfp341 |
|  | Ifit3 | Gm13050 |  | Tmem182 |  |  |  |  |  |  |  | Med24 | 2610507I01Rik |
|  | Icosl | Fbxl8 |  | Gm43181 |  |  |  |  |  |  |  | Slc33a1 | Tceanc |
|  | Srgap2 | Gmpr |  | Tnfsf9 |  |  |  |  |  |  |  | Ndufa12 | Gm6542 |
|  | Cyb5r1 | Maip1 |  | Gm14269 |  |  |  |  |  |  |  | Mrpl49 | Ccdc167 |
|  | Cdkn2aip | B3gnt9 |  | Fcrlb |  |  |  |  |  |  |  | Prpf38a | Gdf15 |
|  | Camsap2 | Slf1 |  | Gpr27 |  |  |  |  |  |  |  | Dhdds | Airn |
|  | Zfp367 | Dohh |  | Cd27 |  |  |  |  |  |  |  | Ptcd3 | Ugt1a6a |
|  | Ugt2b35 | Ift27 |  | Gm14719 |  |  |  |  |  |  |  | Rprd1b | AC154232.2 |
|  | Rnf170 | Dynlt1b |  | Treml4 |  |  |  |  |  |  |  | Edc4 | Echdc3 |
|  | Rsad2 | Nt5m |  | Sult6b2 |  |  |  |  |  |  |  | Xpo5 | Ccdc17 |
|  | Pear1 | Pcbd2 |  | Pla2g12b |  |  |  |  |  |  |  | Nifk | 2700099C18Rik |
|  | Chml | Trpv3 |  | Erich4 |  |  |  |  |  |  |  | Cherp | Gm13375 |
|  | Eif2ak4 | Tmem60 |  | Prrt1 |  |  |  |  |  |  |  | Cpox | Lmntd2 |
|  | Tmem170 | Bcs1l |  | Tnfrsf4 |  |  |  |  |  |  |  | Pold2 | 2210039B01Rik |
|  | Fam126b | Echdc2 |  | Klk8 |  |  |  |  |  |  |  | Mtmr12 | Gm16185 |
|  | Dhx58 | Gm44950 |  | Tmem40 |  |  |  |  |  |  |  | Pycr2 | 2410022M11Rik |
|  | Tnk1 | Kptn |  | Pilrb1 |  |  |  |  |  |  |  | Ino80c | 9030622O22Rik |
|  | Clec2e | Scg2 |  | Ncmap |  |  |  |  |  |  |  | Ccndbp1 | Bex2 |
|  | Sema6a | Sh3bgr |  | Bcl2a1a |  |  |  |  |  |  |  | Ncoa5 | Rps10 |
|  | Tox | Tmem104 |  | Defa24 |  |  |  |  |  |  |  | Aip | Gpr19 |
|  | Aga | Mvb12b |  | Ighv1-20 |  |  |  |  |  |  |  | Gm9800 | Hoxd10 |
|  | Slc51a | Rrm2b |  | Stfa3 |  |  |  |  |  |  |  | Mrpl38 | Gm38244 |
|  | Ercc5 | Elmod3 |  | Gm10499 |  |  |  |  |  |  |  | Ublcp1 | Dancr |
|  | Ctnnbip1 | Ttc32 |  | Gstm2-ps1 |  |  |  |  |  |  |  | Lsm12 | Zfp931 |
|  | Siae | Rhod |  | Gm36161 |  |  |  |  |  |  |  | Hspa14 | 1810014B01Rik |
|  | Zbtb10 | Hebp2 |  | Bcl2a1b |  |  |  |  |  |  |  | Smarca2 | A530017D24Rik |
|  | Zbtb39 | Rps4l |  | 1700058P15Rik |  |  |  |  |  |  |  | Ddx52 | Rom1 |
|  | Mitd1 | Them4 |  | Cend1 |  |  |  |  |  |  |  | Pin1 | 9330151L19Rik |
|  | AA986860 | Syt11 |  | Kcna3 |  |  |  |  |  |  |  | Unc13b | Eci3 |
|  | Slc52a2 | Ret |  | Cartpt |  |  |  |  |  |  |  | Pmvk | Gm15912 |
|  | Ric8b | Zfp790 |  | Rsph9 |  |  |  |  |  |  |  | Nop10 | Pelo |
|  | Igkv8-27 | Rpap2 |  | Leap2 |  |  |  |  |  |  |  | Lyar | Gm15401 |
|  | Xaf1 | Thbs2 |  | Tnni2 |  |  |  |  |  |  |  | Lsm6 | Hpn |
|  | Lmbr1 | 2810025M15Rik |  | D630033A02Rik |  |  |  |  |  |  |  | Ccdc71 | Gm12663 |
|  | Whamm | Tatdn1 |  | Fev |  |  |  |  |  |  |  | Rrs1 | Lrrc17 |
|  | Map3k10 | 8030462N17Rik |  | Gm16091 |  |  |  |  |  |  |  | Rhoq | 2810408A11Rik |
|  | Nxt2 | Ogg1 |  | Gm26740 |  |  |  |  |  |  |  | Med17 | Plekhd1 |
|  | Aqp11 | Dcaf4 |  | AI839979 |  |  |  |  |  |  |  | Mfsd5 | 6720427I07Rik |
|  | Cdon | Gnao1 |  | Tubg2 |  |  |  |  |  |  |  | Nck1 | Immp2l |
|  | Svip | Ptpro |  | Snai3 |  |  |  |  |  |  |  | Phf10 | Slc25a47 |
|  | Myzap | Gstm4 |  | I830127L07Rik |  |  |  |  |  |  |  | Ubac1 | Zfp975 |
|  | Rph3al | Ttc21b |  | Hrct1 |  |  |  |  |  |  |  | Kank2 | Mir5125 |
|  | Ifit1bl2 | Lin37 |  | Ly6i |  |  |  |  |  |  |  | Nom1 | Gm20735 |
|  | Xk | 1810019D21Rik |  | 2010005H15Rik |  |  |  |  |  |  |  | Eif2d | Slc2a4rg-ps |
|  | Ffar4 | Nprl2 |  | Gjb4 |  |  |  |  |  |  |  | Lsm8 | Gm830 |
|  | Cyp2d26 | C1qtnf12 |  | Tmem190 |  |  |  |  |  |  |  | Taf2 | Msrb2 |
|  | Psen2 | AC121151.1 |  | Cst7 |  |  |  |  |  |  |  | Prpf31 | Tmem81 |
|  | Ifi44 | Prph |  | Fam167b |  |  |  |  |  |  |  | Mrpl40 | Sp3os |
|  | Ptcd1 | Cspg4 |  | Gm9115 |  |  |  |  |  |  |  | Dapk3 | AC174678.1 |
|  | Vldlr | Rhbdd3 |  | Gm13056 |  |  |  |  |  |  |  | Gtf2a1 | Gm14305 |
|  | Smco4 | Cyb5d1 |  | Gm20186 |  |  |  |  |  |  |  | Vps33a | Gm14584 |
|  | Zbtb14 | Rps13-ps2 |  | Gm16174 |  |  |  |  |  |  |  | Cenpe | 9230116L04Rik |
|  | Lztfl1 | Atp5s |  | Defa30 |  |  |  |  |  |  |  | Cc2d1b | Gm4285 |
|  | Ecscr | Arl15 |  | Nat8 |  |  |  |  |  |  |  | Ciapin1 | Gm14207 |
|  | Herc3 | Tstd3 |  | Gm9733 |  |  |  |  |  |  |  | Desi1 | Kazald1 |
|  | Ciita | Ube2d-ps |  | 2210406H18Rik |  |  |  |  |  |  |  | Brix1 | E130311K13Rik |
|  | Elac1 | Dvl2 |  | CR974586.5 |  |  |  |  |  |  |  | Fbxl20 | Slc16a12 |
|  | Gsdme | Rpl9-ps7 |  | Zfp422-ps |  |  |  |  |  |  |  | Mrpl21 | Thumpd2 |
|  | Pgap3 | Rnf146 |  | Gm16071 |  |  |  |  |  |  |  | Naa40 | Smpx |
|  | Camsap1 | Usp54 |  | Gm7019 |  |  |  |  |  |  |  | Zbtb38 | Ccpg1os |
|  | Miga1 | Myl12b |  | Gm27010 |  |  |  |  |  |  |  | Pom121 | A130014A01Rik |
|  | Dusp7 | Lrrc31 |  | Hmgb1-ps8 |  |  |  |  |  |  |  | Sema6d | Mterf1a |
|  | Rundc3b | Rnf219 |  | Bambi-ps1 |  |  |  |  |  |  |  | Exoc6b | Znrd1as |
|  | Slc25a19 | Tmem141 |  | U90926 |  |  |  |  |  |  |  | Rfx7 | Zfp599 |
|  | Ang | Aplf |  | Wfdc12 |  |  |  |  |  |  |  | Fez2 | 8430429K09Rik |
|  | 10-Sep | Rps27a-ps2 |  | Camp |  |  |  |  |  |  |  | Rab28 | BC051226 |
|  | Gadd45g | Mmachc |  | Gm17024 |  |  |  |  |  |  |  | Rhot1 | 2810030D12Rik |
|  | Cry2 | Cd59a |  | E230014E18Rik |  |  |  |  |  |  |  | Acox3 | Smim5 |
|  | Phf13 | Mtrf1l |  | Scrg1 |  |  |  |  |  |  |  | Srm | Hist3h2a |
|  | Hace1 | Mthfsl |  | Gm14851 |  |  |  |  |  |  |  | Trnt1 | Slc16a11 |
|  | Xpc | Mob3a |  | CR974586.3 |  |  |  |  |  |  |  | Senp1 | Gm48768 |
|  | Pdzd3 | Hspb11 |  | Gm43525 |  |  |  |  |  |  |  | Ubqln2 | 5830428M24Rik |
|  | Slc25a40 | 2010320M18Rik |  | Orm1 |  |  |  |  |  |  |  | Crebbp | 4930522L14Rik |
|  | Dact2 | Rnf24 |  | Gm20234 |  |  |  |  |  |  |  | Las1l | Slc6a7 |
|  | Afg1l | Ndufb2 |  | Gm10134 |  |  |  |  |  |  |  | Pi4k2a | 2900076A07Rik |
|  | Fam133b | Borcs7 |  | Phf2os1 |  |  |  |  |  |  |  | Cops8 | 9330162012Rik |
|  | Klhl42 | Arl16 |  | Rpl13-ps6 |  |  |  |  |  |  |  | Slc7a7 | 1010001N08Rik |
|  | Parp16 | Trmu |  | Gm28913 |  |  |  |  |  |  |  | Trappc5 | Dnajb13 |
|  | Thrb | 2310009A05Rik |  | Gm36551 |  |  |  |  |  |  |  | Pnp | 1700052K11Rik |
|  | Fam220a | Uxt |  | Gm43555 |  |  |  |  |  |  |  | Nusap1 | Hoxa6 |
|  | Osr2 | Gm6055 |  | Snord89 |  |  |  |  |  |  |  | Sos2 | Gm16973 |
|  | Fbxl18 | Sst |  | Gm15347 |  |  |  |  |  |  |  | Cars | Il22ra2 |
|  | Vav2 | Tsen15 |  | Ighv12-3 |  |  |  |  |  |  |  | Gatad2b | Tmem191c |
|  | Cep295 | Borcs5 |  | Gm13092 |  |  |  |  |  |  |  | Ngdn | 4930481A15Rik |
|  | Cwf19l2 | Hacd4 |  | Mir7-1 |  |  |  |  |  |  |  | Eif2b4 | Ssc4d |
|  | Pitx1 | Ror2 |  | Gm20689 |  |  |  |  |  |  |  | Megf8 | Pcsk4 |
|  | Nek1 | Akr1b10 |  | Iglj3p |  |  |  |  |  |  |  | Slc25a12 | Nhej1 |
|  | Ppargc1b | Ift74 |  | CT571247.2 |  |  |  |  |  |  |  | Nop2 | Zfp3 |
|  | Bag4 | Dnajc17 |  | Snora41 |  |  |  |  |  |  |  | Timm29 | Gm15545 |
|  | Plekhg6 | 2610021A01Rik |  | Snora69 |  |  |  |  |  |  |  | Plrg1 | E230025N22Rik |
|  | A1cf | Zfp239 |  | Mir5107 |  |  |  |  |  |  |  | Snx19 | Gm19605 |
|  | Tmtc4 | 2310033P09Rik |  | Mir7678 |  |  |  |  |  |  |  | Szt2 | 1500015A07Rik |
|  | Pde6d | 1600002K03Rik |  | Mir142b |  |  |  |  |  |  |  | Bcor | K230010J24Rik |
|  | Rian | Ctif |  | Gm23966 |  |  |  |  |  |  |  | Nubp2 | Zfp947 |
|  | Vegfb | Gm15710 |  | Snord32a |  |  |  |  |  |  |  | Dph3 | Zfp939 |
|  | Hps3 | Sclt1 |  | Trbj1-2 |  |  |  |  |  |  |  | Actl6a | Rnls |
|  | Dhx35 | Poll |  | Trbj2-1 |  |  |  |  |  |  |  | Cnot3 | 4930579G18Rik |
|  | Prkg2 | Nkd1 |  | Gm26115 |  |  |  |  |  |  |  | Slc25a44 | Cmc4 |
|  | Snhg5 | Slc28a3 |  | Mir1957a |  |  |  |  |  |  |  | Gnl2 | Gm6457 |
|  | Stau2 | Mapt |  | Mir7031 |  |  |  |  |  |  |  | Nup85 | Zfp13 |
|  | Bmf | Slc2a8 |  | Trbj1-6 |  |  |  |  |  |  |  | Fyco1 | Mroh3 |
|  | Adprm | Fam117a |  | Ighd4-1 |  |  |  |  |  |  |  | Ppat | Gm43667 |
|  | N4bp3 | Castor2 |  |  |  |  |  |  |  |  |  | 0610030E20Rik | B330016D10Rik |
|  | Ppp2r3a | Bag2 |  |  |  |  |  |  |  |  |  | Drg2 | Klhdc1 |
|  | Gca | Uchl1 |  |  |  |  |  |  |  |  |  | Dnajc15 | Gm16675 |
|  | Cyp4b1 | Gm10073 |  |  |  |  |  |  |  |  |  | Tmem51 | 4933406C10Rik |
|  | Map3k13 | Zfp750 |  |  |  |  |  |  |  |  |  | Slc5a3 | 3930402G23Rik |
|  | Chn2 | Cep44 |  |  |  |  |  |  |  |  |  | Hax1 | Ywhaq-ps3 |
|  | Arhgap20 | 5730480H06Rik |  |  |  |  |  |  |  |  |  | Golt1b | Gm1976 |
|  | Dok1 | Asb1 |  |  |  |  |  |  |  |  |  | Bahd1 | Gemin8 |
|  | Gk5 | Tmem181c-ps |  |  |  |  |  |  |  |  |  | Trim12a | Gm15519 |
|  | Cnot4 | Nsg2 |  |  |  |  |  |  |  |  |  | Mrps10 | Gm28370 |
|  | Setbp1 | Fam83c |  |  |  |  |  |  |  |  |  | Rarres2 | Atp6v1g2 |
|  | Dpyd | 2310009B15Rik |  |  |  |  |  |  |  |  |  | Fam98b | Gm2999 |
|  | Sccpdh | Gm4617 |  |  |  |  |  |  |  |  |  | Pbk | 2300009A05Rik |
|  | Spata6 | Dsel |  |  |  |  |  |  |  |  |  | Dhrs3 | Zfp960 |
|  | Usp30 | Slc46a1 |  |  |  |  |  |  |  |  |  | Pdcd11 | Gm2981 |
|  | Lmf1 | Smim4 |  |  |  |  |  |  |  |  |  | Tusc3 | Lipt1 |
|  | Kcnh3 | Gm11430 |  |  |  |  |  |  |  |  |  | Ddx41 | Gm20219 |
|  | Smarcal1 | Tbc1d25 |  |  |  |  |  |  |  |  |  | Vps50 | Gm2260 |
|  | Zdhhc23 | Neil3 |  |  |  |  |  |  |  |  |  | Nox1 | 2610306M01Rik |
|  | Hps4 | Gstm7 |  |  |  |  |  |  |  |  |  | Mrpl41 | 5033404E19Rik |
|  | Gfod2 | Il7 |  |  |  |  |  |  |  |  |  | Dnmt3a | Fcamr |
|  | Fam204a | Ficd |  |  |  |  |  |  |  |  |  | Acad9 | Hoxa2 |
|  | Rnf214 | Slc6a4 |  |  |  |  |  |  |  |  |  | Rictor | 5330438D12Rik |
|  | Tigd2 | Zfp54 |  |  |  |  |  |  |  |  |  | Rbm28 | Gm49284 |
|  | Lrp12 | Rims1 |  |  |  |  |  |  |  |  |  | Cstf2t | Lrrc51 |
|  | Slc8a1 | D11Wsu47e |  |  |  |  |  |  |  |  |  | Tsr1 | Krt10 |
|  | Zfp869 | Osgepl1 |  |  |  |  |  |  |  |  |  | Cep83 | Frg2f1 |
|  | Pitpnm3 | BC052040 |  |  |  |  |  |  |  |  |  | Map3k1 | Gm49359 |
|  | Scoc | Wdr53 |  |  |  |  |  |  |  |  |  | Ptgr1 | Agtr1a |
|  | Txlng | Iqcc |  |  |  |  |  |  |  |  |  | Asb8 | 1700086O06Rik |
|  | Ovol1 | Cd1d1 |  |  |  |  |  |  |  |  |  | Rbbp8 | Gm19391 |
|  | Atg14 | Pstpip2 |  |  |  |  |  |  |  |  |  | Sap30bp | Gm31718 |
|  | Apol9b | Bfsp1 |  |  |  |  |  |  |  |  |  | Knstrn | Zfp58 |
|  | Zfp84 | Gin1 |  |  |  |  |  |  |  |  |  | Paxip1 | Zscan2 |
|  | Pdxk | Gstm6 |  |  |  |  |  |  |  |  |  | Pole4 | Gm28417 |
|  | Gorab | Rab6b |  |  |  |  |  |  |  |  |  | Rexo4 | Gm16907 |
|  | Acap3 | Kif3a |  |  |  |  |  |  |  |  |  | Atmin | Gm48604 |
|  | Trim14 | Hoxa11 |  |  |  |  |  |  |  |  |  | Gm6563 | Gm11423 |
|  | Pibf1 | Tma7-ps |  |  |  |  |  |  |  |  |  | Rpf1 | Mnd1 |
|  | Zxdc | Pex10 |  |  |  |  |  |  |  |  |  | Mcm3ap | Tcea2 |
|  | Spindoc | Gm18194 |  |  |  |  |  |  |  |  |  | Tbc1d23 | Gm16011 |
|  | Map3k6 | Hand2 |  |  |  |  |  |  |  |  |  | Nol9 | Camk2n2 |
|  | Herc6 | Ccdc166 |  |  |  |  |  |  |  |  |  | Nae1 | Ccnjl |
|  | Naaladl1 | Mettl8 |  |  |  |  |  |  |  |  |  | Bub1b | Gm26982 |
|  | Stradb | Gtpbp6 |  |  |  |  |  |  |  |  |  | Tarbp2 | Gm28438 |
|  | 1110012L19Rik | C4bp |  |  |  |  |  |  |  |  |  | Prc1 | A430046D13Rik |
|  | 1700019D03Rik | Rusc1 |  |  |  |  |  |  |  |  |  | Ndufaf4 | Gm6712 |
|  | Selenoo | Tnfsf13os |  |  |  |  |  |  |  |  |  | Rpl13a | Smim27 |
|  | Parp11 | Ssbp2 |  |  |  |  |  |  |  |  |  | Pwp1 | Gm43031 |
|  | Usp18 | Gm10709 |  |  |  |  |  |  |  |  |  | Emc8 | L3hypdh |
|  | Gm5431 | Haghl |  |  |  |  |  |  |  |  |  | Pde12 | Gm36266 |
|  | Garem1 | Ccdc181 |  |  |  |  |  |  |  |  |  | Kat2a | Cabcoco1 |
|  | Shf | Plin4 |  |  |  |  |  |  |  |  |  | Dhx8 | Gm20632 |
|  | Ighv10-1 | Rab11fip2 |  |  |  |  |  |  |  |  |  | Ubr7 | Cyp2d37-ps |
|  | Farp2 | Sp4 |  |  |  |  |  |  |  |  |  | Atad2 | Gm27817 |
|  | Tbcc | H2-T24 |  |  |  |  |  |  |  |  |  | Cdca4 | AI463229 |
|  | Map1s | Ddt |  |  |  |  |  |  |  |  |  | Ecd | Gm47071 |
|  | Bivm | Trmt12 |  |  |  |  |  |  |  |  |  | Jmjd6 | Fhit |
|  | Prodh | Cebpzos |  |  |  |  |  |  |  |  |  | Isg20l2 | Hexim2 |
|  | Avpi1 | Mettl5 |  |  |  |  |  |  |  |  |  | Ccdc97 | Gm43071 |
|  | Gm12191 | Zfp9 |  |  |  |  |  |  |  |  |  | Nop9 | N4bp2os |
|  | Mef2c | Col23a1 |  |  |  |  |  |  |  |  |  | Parp10 | Gm45769 |
|  | Hs1bp3 | Ly6g6e |  |  |  |  |  |  |  |  |  | Cdc26 | Gm11821 |
|  | Rapgef3 | Rnf135 |  |  |  |  |  |  |  |  |  | Slc35c2 | Gm28229 |
|  | Lnx1 | Gm10288 |  |  |  |  |  |  |  |  |  | Map1lc3a | Mapk15 |
|  | Sytl4 | Gdpd5 |  |  |  |  |  |  |  |  |  | Odf2 | Nudt6 |
|  | Gprin3 | Smim26 |  |  |  |  |  |  |  |  |  | Dynlt1-ps1 | Scel |
|  | Dynlt1c | Gm17828 |  |  |  |  |  |  |  |  |  | Hook3 | Gm9856 |
|  | Tbx3 | 2310079G19Rik |  |  |  |  |  |  |  |  |  | Psat1 | Gm9923 |
|  | Gbe1 | Ndrg4 |  |  |  |  |  |  |  |  |  | Nfyb | Rprm |
|  | Mapre3 | Cyp2d12 |  |  |  |  |  |  |  |  |  | Dbf4 | Gm14399 |
|  | Npas2 | Phtf2 |  |  |  |  |  |  |  |  |  | Trmt2a | Gm43254 |
|  | Zhx2 | Gm45133 |  |  |  |  |  |  |  |  |  | Mrpl39 | 2310010J17Rik |
|  | Phf11b | Klhl36 |  |  |  |  |  |  |  |  |  | Pprc1 | Gm32391 |
|  | Hoxb6 | Nupl2 |  |  |  |  |  |  |  |  |  | Plekhj1 | Gm9762 |
|  | Srr | Brf2 |  |  |  |  |  |  |  |  |  | Acot9 | Dcst2 |
|  | Slc25a23 | 2700062C07Rik |  |  |  |  |  |  |  |  |  | Sap130 | Gm7285 |
|  | Zbtb7c | Snhg15 |  |  |  |  |  |  |  |  |  | Rad23a | 2900052L18Rik |
|  | Slc13a1 | Tmem203 |  |  |  |  |  |  |  |  |  | Mesd | Gm10767 |
|  | Mtr | Sergef |  |  |  |  |  |  |  |  |  | Farsa | 9130221H12Rik |
|  | Tiam2 | Ovca2 |  |  |  |  |  |  |  |  |  | Pitrm1 | Gm36738 |
|  | Chic1 | Slc25a33 |  |  |  |  |  |  |  |  |  | Slc45a3 | Gm45495 |
|  | Kctd14 | Amigo2 |  |  |  |  |  |  |  |  |  | Poc1b | A630052C17Rik |
|  | Pcsk1 | Igkv3-7 |  |  |  |  |  |  |  |  |  | Kat14 | Mylpf |
|  | Gm12250 | Mcts2 |  |  |  |  |  |  |  |  |  | Nmd3 | Nthl1 |
|  | Lig4 | Spock2 |  |  |  |  |  |  |  |  |  | Msra | Tssk4 |
|  | C330018D20Rik | Flywch2 |  |  |  |  |  |  |  |  |  | Ttc1 | 4632428C04Rik |
|  | Phtf1 | Efna4 |  |  |  |  |  |  |  |  |  | Lrch1 | Gm19680 |
|  | Adck2 | Rpl7a-ps11 |  |  |  |  |  |  |  |  |  | Rbm45 | Zfp963 |
|  | Rgs17 | Cradd |  |  |  |  |  |  |  |  |  | Smad2 | Gm14292 |
|  | Osbpl10 | 2810001G20Rik |  |  |  |  |  |  |  |  |  | Hells | Akt2-ps |
|  | Rab17 | Mxd3 |  |  |  |  |  |  |  |  |  | Snrpf | Gm16015 |
|  | Zfp827 | A430033K04Rik |  |  |  |  |  |  |  |  |  | Ezh2 | Gm2223 |
|  | Dyrk1b | Snrpert |  |  |  |  |  |  |  |  |  | Msh2 | Tmem255b |
|  | Acnat1 | Filip1 |  |  |  |  |  |  |  |  |  | Thoc1 | Gm14287 |
|  | Tox3 | Zfp235 |  |  |  |  |  |  |  |  |  | Heatr1 | Gm20275 |
|  | Rdm1 | Acyp1 |  |  |  |  |  |  |  |  |  | Rrn3 | Gm10399 |
|  | Zfp868 | Tldc2 |  |  |  |  |  |  |  |  |  | Hs2st1 | Mgl2 |
|  | Tbc1d32 | Ascl2 |  |  |  |  |  |  |  |  |  | Hbp1 | C920006O11Rik |
|  | Gm14494 | Hoga1 |  |  |  |  |  |  |  |  |  | Mrps12 | Gm43681 |
|  | Tmem184c | Mettl4 |  |  |  |  |  |  |  |  |  | Fbxo34 | Gm5555 |
|  | Ston1 | Ndn |  |  |  |  |  |  |  |  |  | Crnkl1 | Cyp2f2 |
|  | Shpk | Tusc1 |  |  |  |  |  |  |  |  |  | Ddx18 | Gm17251 |
|  | Phlda3 | Hs3st3b1 |  |  |  |  |  |  |  |  |  | Fbxo22 | D730003I15Rik |
|  | Ccser1 | Pcgf1 |  |  |  |  |  |  |  |  |  | Rae1 | Gm29008 |
|  | Tma16 | Fabp6 |  |  |  |  |  |  |  |  |  | Nvl | Gm20257 |
|  | Tspan17 | Itgb3bp |  |  |  |  |  |  |  |  |  | Slc25a22 | 1810017P11Rik |
|  | Sstr1 | Ighv9-1 |  |  |  |  |  |  |  |  |  | Zfp62 | 5830448L01Rik |
|  | Penk | Lefty1 |  |  |  |  |  |  |  |  |  | Ints14 | 1300014J16Rik |
|  | Etfb | Gm12918 |  |  |  |  |  |  |  |  |  | Ints7 | 2510046G10Rik |
|  | Dixdc1 | Ly96 |  |  |  |  |  |  |  |  |  | Pdzd11 | Cyp2w1 |
|  | Tpmt | Slain1 |  |  |  |  |  |  |  |  |  | Plch1 | Alyref2 |
|  | Trim21 | Tacr1 |  |  |  |  |  |  |  |  |  | Men1 | Gm27898 |
|  | Entpd2 | Iyd |  |  |  |  |  |  |  |  |  | Parg | 9130214F15Rik |
|  | Tmem143 | 9030619P08Rik |  |  |  |  |  |  |  |  |  | Dnajc21 | Capsl |
|  | Wars2 | Casp14 |  |  |  |  |  |  |  |  |  | Lrfn4 | 4933440N22Rik |
|  | Ntn4 | Rtn1 |  |  |  |  |  |  |  |  |  | Lnx2 | Gm38102 |
|  | Fgfrl1 | Fam234b |  |  |  |  |  |  |  |  |  | Itpr1 | Ctf1 |
|  | D2hgdh | Dok7 |  |  |  |  |  |  |  |  |  | Gtf2b | Gm20457 |
|  | Arl14 | Snx16 |  |  |  |  |  |  |  |  |  | 2810004N23Rik | Gm43745 |
|  | Gdpgp1 | Plekhb1 |  |  |  |  |  |  |  |  |  | Tars2 | C8g |
|  | Ophn1 | 9230105E05Rik |  |  |  |  |  |  |  |  |  | Pmf1 | Gm16046 |
|  | Rbp4 | Avil |  |  |  |  |  |  |  |  |  | Hat1 | Stxbp3-ps |
|  | 4632415L05Rik | Rpl35a-ps2 |  |  |  |  |  |  |  |  |  | Mrpl46 | E330034L11Rik |
|  | Urb1 | Gm14586 |  |  |  |  |  |  |  |  |  | Socs2 | A930024E05Rik |
|  | Rmdn1 | Arhgap8 |  |  |  |  |  |  |  |  |  | Timm22 | Myl6b |
|  | Naip3 | Tfb1m |  |  |  |  |  |  |  |  |  | Zfyve27 | Gm1943 |
|  | Wnk2 | Pla2g10os |  |  |  |  |  |  |  |  |  | Smad7 | Gm44509 |
|  | Pla2g3 | Cldn14 |  |  |  |  |  |  |  |  |  | Aplp1 | Gm36298 |
|  | Thada | Gm14539 |  |  |  |  |  |  |  |  |  | Zpr1 | Fgfbp3 |
|  | Zdhhc24 | 1700096K18Rik |  |  |  |  |  |  |  |  |  | Fgd6 | Gm15884 |
|  | Shank2 | Prkar1b |  |  |  |  |  |  |  |  |  | Asl | Ribc1 |
|  | Rasd2 | 4732471J01Rik |  |  |  |  |  |  |  |  |  | Nutf2 | Gm14508 |
|  | Natd1 | CT025731.4 |  |  |  |  |  |  |  |  |  | Kctd17 | Cfap300 |
|  | Prmt2 | Adap2 |  |  |  |  |  |  |  |  |  | Cdk6 | 5033430I15Rik |
|  | Itgb1bp1 | 9530018H14Rik |  |  |  |  |  |  |  |  |  | Timm17b | Tssk6 |
|  | Slc25a30 | Mapk8ip1 |  |  |  |  |  |  |  |  |  | Gar1 | Gm9958 |
|  | Trmt61a | Zfp524 |  |  |  |  |  |  |  |  |  | Sf3a2 | Sat2 |
|  | Nrm | Mab21l2 |  |  |  |  |  |  |  |  |  | Krr1 | Gm39469 |
|  | Myo7a | Gm9385 |  |  |  |  |  |  |  |  |  | Ddx10 | Bex1 |
|  | Sp110 | Epop |  |  |  |  |  |  |  |  |  | Urm1 | Gm40348 |
|  | Mfsd8 | 9-Mar |  |  |  |  |  |  |  |  |  | Igkv4-59 | Gm16580 |
|  | Gm21885 | Rims4 |  |  |  |  |  |  |  |  |  | Sfxn3 | 2810405F17Rik |
|  | Plxnb1 | Rab7-ps1 |  |  |  |  |  |  |  |  |  | Nme1 | Cox20 |
|  | Lpin1 | B230307C23Rik |  |  |  |  |  |  |  |  |  | Nop16 | Gm14648 |
|  | Ighv1-72 | Spata2l |  |  |  |  |  |  |  |  |  | Nup93 | Gm28192 |
|  | Lysmd4 | Gal3st2b |  |  |  |  |  |  |  |  |  | Dnajc9 | 2510016D11Rik |
|  | Slx4ip | Eef1akmt4 |  |  |  |  |  |  |  |  |  | Ncapg2 | Gm7327 |
|  | Mfsd12 | Nme4 |  |  |  |  |  |  |  |  |  | Rwdd1 | Rsph1 |
|  | Syde2 | Eno2 |  |  |  |  |  |  |  |  |  | Ggps1 | Gm28901 |
|  | Aldh1a7 | Hap1 |  |  |  |  |  |  |  |  |  | Usp39 | E030042O20Rik |
|  | Ppp1ccb | Gm11942 |  |  |  |  |  |  |  |  |  | Sdad1 | Gm42732 |
|  | Ftl1-ps1 | Sdhaf1 |  |  |  |  |  |  |  |  |  | Shkbp1 | 1810044D09Rik |
|  | AI464131 | Syngr1 |  |  |  |  |  |  |  |  |  | Cip2a | 4930516B21Rik |
|  | Sirt3 | Htr3a |  |  |  |  |  |  |  |  |  | Sys1 | Gm15777 |
|  | 9130008F23Rik | Ubxn10 |  |  |  |  |  |  |  |  |  | Dffa | Gm17112 |
|  | Apol6 | Comtd1 |  |  |  |  |  |  |  |  |  | Cstf1 | 0610009L18Rik |
|  | Ugt2b5 | Zmynd15 |  |  |  |  |  |  |  |  |  | Inpp5k | Gm4130 |
|  | Ppl | Gm48809 |  |  |  |  |  |  |  |  |  | Rnf41 | Gm12418 |
|  | Actr5 | Fdps |  |  |  |  |  |  |  |  |  | Nip7 | Gm13414 |
|  | Rita1 | 2010008C14Rik |  |  |  |  |  |  |  |  |  | Tmem37 | Gm14597 |
|  | Ttc23 | Pirt |  |  |  |  |  |  |  |  |  | Tbl2 | Mterf1b |
|  | Slc7a4 | Ppp1r3c |  |  |  |  |  |  |  |  |  | Gm4204 | Angptl8 |
|  | D3Ertd254e | Gm12989 |  |  |  |  |  |  |  |  |  | Brwd3 | Gm12860 |
|  | Tubal3 | Fkbpl |  |  |  |  |  |  |  |  |  | Gm10241 | Gm29650 |
|  | Cir1 | Rpl39-ps |  |  |  |  |  |  |  |  |  | Ints13 | 1810021B22Rik |
|  | Fzd8 | 2500004C02Rik |  |  |  |  |  |  |  |  |  | Cnot11 | Gm16008 |
|  | A130010J15Rik | Gm7666 |  |  |  |  |  |  |  |  |  | Thyn1 | Cox4i2 |
|  | Extl2 | Gm16418 |  |  |  |  |  |  |  |  |  | Abcd1 | Inca1 |
|  | Amer1 | E030030I06Rik |  |  |  |  |  |  |  |  |  | Mrpl16 | 9130604C24Rik |
|  | Ddx28 | Rpl10-ps3 |  |  |  |  |  |  |  |  |  | Mrps34 | Gm8162 |
|  | Nlrp9b | Hoxd11 |  |  |  |  |  |  |  |  |  | Pdcd5 | Hist1h4h |
|  | Agbl5 | Gm20604 |  |  |  |  |  |  |  |  |  | Ubald1 | Far1os |
|  | Isoc2b | Gm10243 |  |  |  |  |  |  |  |  |  | Mrps25 | Colca2 |
|  | Cpn1 | Gm10384 |  |  |  |  |  |  |  |  |  | Trim37 | Gm18733 |
|  | Otc | Gm8730 |  |  |  |  |  |  |  |  |  | Zfp326 | 4933431K14Rik |
|  | Ipp | Tspyl4 |  |  |  |  |  |  |  |  |  | Coq7 | Gm4714 |
|  | Hsd17b13 | Shld1 |  |  |  |  |  |  |  |  |  | Rfc5 | Gm36371 |
|  | 2610035D17Rik | Gm27605 |  |  |  |  |  |  |  |  |  | Rhof | Retnla |
|  | Rogdi | Col28a1 |  |  |  |  |  |  |  |  |  | Melk | Gm10226 |
|  | Kcnf1 | Gm15706 |  |  |  |  |  |  |  |  |  | Zfp655 | Pgk1-rs7 |
|  | Bud13 | Gm10263 |  |  |  |  |  |  |  |  |  | Ltv1 | Hotairm1 |
|  | Ighv1-15 | Lrp11 |  |  |  |  |  |  |  |  |  | Taf13 | Gm45091 |
|  | 5033406O09Rik | Gm16477 |  |  |  |  |  |  |  |  |  | Mov10 | Spa17 |
|  | Acy3 | 3110001I22Rik |  |  |  |  |  |  |  |  |  | Mrpl35 | Gm27042 |
|  | B230354K17Rik | Gm13192 |  |  |  |  |  |  |  |  |  | Pfdn2 | Gm2423 |
|  | Creld1 | Gm340 |  |  |  |  |  |  |  |  |  | Ints6 | Bex4 |
|  | Wrb | Fam222a |  |  |  |  |  |  |  |  |  | Hspbp1 | Gm45453 |
|  | Mus81 | Kbtbd3 |  |  |  |  |  |  |  |  |  | Cdc23 | 2900093K20Rik |
|  | Arl10 | Scg3 |  |  |  |  |  |  |  |  |  | 1810030O07Rik | 1810062G17Rik |
|  | Gm45871 | P3h2 |  |  |  |  |  |  |  |  |  | Cwc22 | Gm45756 |
|  | Igkv8-21 | Gm11518 |  |  |  |  |  |  |  |  |  | Mrps2 | Gm5406 |
|  | Sct | Fndc10 |  |  |  |  |  |  |  |  |  | Rpp14 | Gm18113 |
|  | Nr2c1 | Gjb5 |  |  |  |  |  |  |  |  |  | Tmem243 | Gm35189 |
|  | Slc2a10 | Mblac1 |  |  |  |  |  |  |  |  |  | Ccdc59 | Gm7162 |
|  | Ccdc149 | Bbs5 |  |  |  |  |  |  |  |  |  | Ccnc | Zfp972 |
|  | Top3a | Csta1 |  |  |  |  |  |  |  |  |  | Nol6 | Gm49066 |
|  | Galnt15 | Lppos |  |  |  |  |  |  |  |  |  | Rbbp5 | Gm12276 |
|  | Nckipsd | Znf41-ps |  |  |  |  |  |  |  |  |  | Ints10 | Hist1h2bg |
|  | Tti2 | Gm45397 |  |  |  |  |  |  |  |  |  | Mrps28 | Gm15265 |
|  | Slc34a2 | 7530428D23Rik |  |  |  |  |  |  |  |  |  | Chchd4 | C230035I16Rik |
|  | Ache | Rpl24 |  |  |  |  |  |  |  |  |  | Nrf1 | Gm14167 |
|  | Nlrp1b | Nhlrc1 |  |  |  |  |  |  |  |  |  | Hmbox1 | Gm44066 |
|  | Tmem175 | Pimreg |  |  |  |  |  |  |  |  |  | Ttll12 | Gm15122 |
|  | Fam69a | Mettl15 |  |  |  |  |  |  |  |  |  | Piga | mt-Ty |
|  | Nts | Rps3a2 |  |  |  |  |  |  |  |  |  | Jagn1 | Gm27454 |
|  | Med7 | 1700030K09Rik |  |  |  |  |  |  |  |  |  | Bcl7b | Hist1h2bj |
|  | Ctbs | Supt3 |  |  |  |  |  |  |  |  |  | Bola3 | Gm9515 |
|  | Ercc1 | Ccdc112 |  |  |  |  |  |  |  |  |  | Larp7 | Gm44706 |
|  | Zfp462 | 4921531C22Rik |  |  |  |  |  |  |  |  |  | Lsm7 | Gm13091 |
|  | Nudt12 | Gm11353 |  |  |  |  |  |  |  |  |  | Cog8 | Chrna1os |
|  | Setdb2 | Gm18588 |  |  |  |  |  |  |  |  |  | Hsd17b7 | Gm48265 |
|  | Rpain | Cdh19 |  |  |  |  |  |  |  |  |  | Ints8 | Tcf15 |
|  | Jade3 | Tmem14a |  |  |  |  |  |  |  |  |  | Aurkb | mt-Te |
|  | Btla | Gm7331 |  |  |  |  |  |  |  |  |  | Ip6k2 | Hist3h2ba |
|  | Gfi1 | Nap1l5 |  |  |  |  |  |  |  |  |  | Anln | Gm44432 |
|  | Zfp141 | Magee1 |  |  |  |  |  |  |  |  |  | Thoc3 | Rpl36a-ps1 |
|  | 0610005C13Rik | 4930415O20Rik |  |  |  |  |  |  |  |  |  | Nup160 | Hist1h4j |
|  | Acad10 | Tmem53 |  |  |  |  |  |  |  |  |  | Clcn5 | Gm11535 |
|  | Cd72 | Neu3 |  |  |  |  |  |  |  |  |  | Ddx56 | Gm29237 |
|  | Stn1 | Gm12174 |  |  |  |  |  |  |  |  |  | Edc3 | Gm8292 |
|  | Tmem82 | Fbxo36 |  |  |  |  |  |  |  |  |  | 1810026B05Rik | Gm32031 |
|  | Trim34a | Gm37768 |  |  |  |  |  |  |  |  |  | Vac14 | Gm26944 |
|  | Cidec | Eef1a2 |  |  |  |  |  |  |  |  |  | Ncdn | Gm28529 |
|  | Ankrd26 | Pdzd4 |  |  |  |  |  |  |  |  |  | Psmf1 | 1110025M09Rik |
|  | 9130024F11Rik | Zfp119b |  |  |  |  |  |  |  |  |  | Selenoh | 2610318M16Rik |
|  | Med26 | Gm15848 |  |  |  |  |  |  |  |  |  | Med19 | Snora31 |
|  | Pm20d1 | Bnipl |  |  |  |  |  |  |  |  |  | Hacd2 | Snhg10 |
|  | Pomgnt2 | Mgmt |  |  |  |  |  |  |  |  |  | Exosc3 | 1110020A21Rik |
|  | Fancg | Zfp273 |  |  |  |  |  |  |  |  |  | Mrps27 | Gm11240 |
|  | Tead4 | Alkbh2 |  |  |  |  |  |  |  |  |  | Ccdc115 | 8430422M14Rik |
|  | Zfp770 | Spata24 |  |  |  |  |  |  |  |  |  | Qsox2 | Gm9320 |
|  | Pusl1 | Enkd1 |  |  |  |  |  |  |  |  |  | Jmy | Gm4032 |
|  | Osgin2 | Gm13436 |  |  |  |  |  |  |  |  |  | Dnlz | Gm27003 |
|  | Cdc7 | Klhl11 |  |  |  |  |  |  |  |  |  | Ppan | Gm22270 |
|  | Gimap9 | Tent5b |  |  |  |  |  |  |  |  |  | Wdr75 | Gm17530 |
|  | Amacr | Mrgpre |  |  |  |  |  |  |  |  |  | Rsbn1l | Gm16574 |
|  | Gas2l3 | Bean1 |  |  |  |  |  |  |  |  |  | Kif22 | Gm29397 |
|  | Pycr1 | Gm10036 |  |  |  |  |  |  |  |  |  | Ankrd12 | Gm26779 |
|  | Zfp1 | Rpl36-ps12 |  |  |  |  |  |  |  |  |  | Rassf4 | Gm15050 |
|  | Pqlc2 | Nccrp1 |  |  |  |  |  |  |  |  |  | Rbm14 | Hist1h4c |
|  | Taf5 | S100g |  |  |  |  |  |  |  |  |  | Ccnk | Smbd1 |
|  | Tex30 | Sult4a1 |  |  |  |  |  |  |  |  |  | Ptprs | Gm15727 |
|  | Pdk4 | Defb45 |  |  |  |  |  |  |  |  |  | Flcn | Gm7180 |
|  | Ogfod3 | Ubtd2 |  |  |  |  |  |  |  |  |  | Twf2 | Gm29093 |
|  | Ccdc66 | Gng4 |  |  |  |  |  |  |  |  |  | Alg2 | Gm10155 |
|  | Gnpda2 | Rps15a-ps6 |  |  |  |  |  |  |  |  |  | Mrto4 | 1810012K08Rik |
|  | Ces1e | Apobec2 |  |  |  |  |  |  |  |  |  | Slc35b4 | Gm30934 |
|  | Tulp3 | Bik |  |  |  |  |  |  |  |  |  | 4930453N24Rik | Gm11724 |
|  | Fam149a | Gm6274 |  |  |  |  |  |  |  |  |  | 2510009E07Rik | Gm15361 |
|  | Malrd1 | 1700003E16Rik |  |  |  |  |  |  |  |  |  | Zcchc17 | Gm44040 |
|  | Fbxw17 | Stmn3 |  |  |  |  |  |  |  |  |  | Gm45855 | Gm6012 |
|  | Srgap3 | 1700037C18Rik |  |  |  |  |  |  |  |  |  | Ruvbl1 | Rn7sk |
|  | Acot4 | Hoxd12 |  |  |  |  |  |  |  |  |  | Inpp1 | Zfp286os |
|  | Abcg1 | Gpc3 |  |  |  |  |  |  |  |  |  | Msh6 | Hnf1aos2 |
|  | Abcb9 | Gm8129 |  |  |  |  |  |  |  |  |  | Rwdd4a | Snora17 |
|  | Nicn1 | Rpl21-ps10 |  |  |  |  |  |  |  |  |  | Thoc5 | Rpl10-ps6 |
|  | Itpka | Zfp85 |  |  |  |  |  |  |  |  |  | Elp2 | CT033749.1 |
|  | Taf9b | Bend5 |  |  |  |  |  |  |  |  |  | Gle1 | AA465934 |
|  | 2610301B20Rik | Gm15657 |  |  |  |  |  |  |  |  |  | Uspl1 | Gm27572 |
|  | Zfp995 | Gm5436 |  |  |  |  |  |  |  |  |  | Umps | 2210417A02Rik |
|  | Sdccag8 | Rpl27-ps3 |  |  |  |  |  |  |  |  |  | Mief1 | 9130230N09Rik |
|  | Txnl4b | Ccdc92 |  |  |  |  |  |  |  |  |  | Tmem64 | Gm11767 |
|  | Cdk5rap1 | Morn2 |  |  |  |  |  |  |  |  |  | Gtf2h3 | Gm8741 |
|  | Cd300lg | 1700001O22Rik |  |  |  |  |  |  |  |  |  | Naa38 | Gm17197 |
|  | Slc2a9 | Rps15a-ps7 |  |  |  |  |  |  |  |  |  | Ormdl3 | Gm48667 |
|  | Cand2 | Rps3a3 |  |  |  |  |  |  |  |  |  | Ints5 | mt-Tr |
|  | A630001G21Rik | Cfap157 |  |  |  |  |  |  |  |  |  | Kdm6a | Gm24119 |
|  | Gm15163 | Fkbp1b |  |  |  |  |  |  |  |  |  | Polr3e | mt-Tg |
|  | Tmem220 | Gm3325 |  |  |  |  |  |  |  |  |  | Crcp | Gm16938 |
|  | Tmppe | Gm10269 |  |  |  |  |  |  |  |  |  | Gtf2e2 | Gm48079 |
|  | Ppih | Zcchc12 |  |  |  |  |  |  |  |  |  | Rabep1 | mt-Atp8 |
|  | Disp1 | Ighv14-1 |  |  |  |  |  |  |  |  |  | Rai1 | Gm13205 |
|  | Zfp820 | Gm6665 |  |  |  |  |  |  |  |  |  | Kif20b | Hist1h4m |
|  | Zfp668 | Gm47483 |  |  |  |  |  |  |  |  |  | Ankrd46 | Rpl36-ps2 |
|  | Gramd1c | Gm12320 |  |  |  |  |  |  |  |  |  | Bok | Gm16238 |
|  | Cpxm1 | Ptges3l |  |  |  |  |  |  |  |  |  | Zfp410 | 4930557K07Rik |
|  | Dusp8 | Syt5 |  |  |  |  |  |  |  |  |  | Zmynd19 | Gm31462 |
|  | Lysmd2 | Vtcn1 |  |  |  |  |  |  |  |  |  | Carm1 | Gm27028 |
|  | Pkia | Gm2614 |  |  |  |  |  |  |  |  |  | Trmt6 | Mup-ps23 |
|  | Gm36640 | Ly6g6d |  |  |  |  |  |  |  |  |  | Hdgfl2 | AC151284.1 |
|  | Gcnt4 | 6330403K07Rik |  |  |  |  |  |  |  |  |  | Tfb2m | Gm7153 |
|  | AC164088.2 | Mettl7a3 |  |  |  |  |  |  |  |  |  | Xpo4 | Gm49539 |
|  | Triqk | Gm7658 |  |  |  |  |  |  |  |  |  | Foxred1 | Snord123 |
|  | Ppara | Gm5526 |  |  |  |  |  |  |  |  |  | Orc4 | Gm16378 |
|  | Pms2 | H3f3c |  |  |  |  |  |  |  |  |  | Hmox2 | Gm17195 |
|  | Pigh | Gm11263 |  |  |  |  |  |  |  |  |  | Cptp | Gm49432 |
|  | Lins1 | Hoxd9 |  |  |  |  |  |  |  |  |  | Itpr2 | Terc |
|  | S1pr3 | 4833445I07Rik |  |  |  |  |  |  |  |  |  | Carmil1 | Gm16143 |
|  | Pcyox1l | 2310040G24Rik |  |  |  |  |  |  |  |  |  | Mrps18a | Gm47814 |
|  | Pigl | Gm17025 |  |  |  |  |  |  |  |  |  | Mrpl19 | 5730437C11Rik |
|  | Tmem254c | Gm10136 |  |  |  |  |  |  |  |  |  | Nmt2 | Gm22574 |
|  | Hcn2 | Gm11478 |  |  |  |  |  |  |  |  |  | Hmmr | Scarna2 |
|  | Kctd21 | Gm16206 |  |  |  |  |  |  |  |  |  | Zdhhc2 | Snord55 |
|  | Edn3 | Gm6807 |  |  |  |  |  |  |  |  |  | Snrpa1 | Rps13-ps4 |
|  | Klf11 | Rgn |  |  |  |  |  |  |  |  |  | Polr2l | Gm22806 |
|  | Chrm1 | Gm2225 |  |  |  |  |  |  |  |  |  | Cstf3 | Gm24494 |
|  | Mpv17l | Gm17619 |  |  |  |  |  |  |  |  |  | Lin54 | Gm10095 |
|  | Nrarp | Guca1a |  |  |  |  |  |  |  |  |  | Tsfm | Gm25082 |
|  | BC048403 | Gm7932 |  |  |  |  |  |  |  |  |  | Rnf123 | Gm22009 |
|  | Ttc30b | Gm8242 |  |  |  |  |  |  |  |  |  | Cavin2 | 4930439D14Rik |
|  | Tcaim | Rps11-ps2 |  |  |  |  |  |  |  |  |  | Slc39a10 | Gm37116 |
|  | Pigw | Gm49326 |  |  |  |  |  |  |  |  |  | Taf12 | Gm5781 |
|  | Gas8 | Gm4262 |  |  |  |  |  |  |  |  |  | Fam208a | Gm27326 |
|  | Calb2 | Rpl10-ps1 |  |  |  |  |  |  |  |  |  | Apba3 | Gm22478 |
|  | Acot1 | Gm13991 |  |  |  |  |  |  |  |  |  | Pip4p2 | Gm15536 |
|  | Scp2-ps2 | Syngr3 |  |  |  |  |  |  |  |  |  | Wdr81 | Rnu73b |
|  | Homez | Stk32c |  |  |  |  |  |  |  |  |  | Rnf2 | Gm20703 |
|  | Btbd19 | A630001O12Rik |  |  |  |  |  |  |  |  |  | Ctnnbl1 | Mir1898 |
|  | Slc35d2 | Gm8451 |  |  |  |  |  |  |  |  |  | Wipi1 | Gm12279 |
|  | 2610005L07Rik | Gm12254 |  |  |  |  |  |  |  |  |  | Psmd10 | Gm23301 |
|  | Amigo3 | Map2k3os |  |  |  |  |  |  |  |  |  | Exosc2 | Gm28706 |
|  | Lrfn3 | Gm4956 |  |  |  |  |  |  |  |  |  | Cep57 | Gm9057 |
|  | Tmem241 | Snord104 |  |  |  |  |  |  |  |  |  | Nat10 | Gm47797 |
|  | Pim2 | Gm15899 |  |  |  |  |  |  |  |  |  | Tac1 | Gm22121 |
|  | Sectm1a | C1qtnf4 |  |  |  |  |  |  |  |  |  | Mis12 | Gm23136 |
|  | Amt | Gm12166 |  |  |  |  |  |  |  |  |  | Cinp | Gm45738 |
|  | Hmga2 | Rps8-ps1 |  |  |  |  |  |  |  |  |  | Aurka | Gm11962 |
|  | Zkscan14 | Gm13408 |  |  |  |  |  |  |  |  |  | Gmnn | Gm10563 |
|  | Padi2 | Grp |  |  |  |  |  |  |  |  |  | Fam160b2 | Snord91a |
|  | Ighv6-6 | D330020A13Rik |  |  |  |  |  |  |  |  |  | Cep192 | Gm13245 |
|  | Gm28512 | Gm15421 |  |  |  |  |  |  |  |  |  | Acer3 | Gm27477 |
|  | Rttn | Gm8355 |  |  |  |  |  |  |  |  |  | Timm8a1 | Snord53 |
|  | Mkx | Rpl17-ps8 |  |  |  |  |  |  |  |  |  | Atxn1 | Snord83b |
|  | Hoxb7 | Gm20149 |  |  |  |  |  |  |  |  |  | Wdr46 | Gm23027 |
|  | AC113595.1 | Gm15972 |  |  |  |  |  |  |  |  |  | Pcsk5 | Gm22771 |
|  | Chrnb4 | Vkorc1 |  |  |  |  |  |  |  |  |  | Cetn2 | AC119957.1 |
|  | Il17d | K230015D01Rik |  |  |  |  |  |  |  |  |  | Tirap | Gm25945 |
|  | Mx1 | Gm16341 |  |  |  |  |  |  |  |  |  | Mphosph10 | Snord88c |
|  | Ror1 | Rps12-ps9 |  |  |  |  |  |  |  |  |  | Csnk1g1 | Gm22572 |
|  | Gfap | Rps19-ps3 |  |  |  |  |  |  |  |  |  | Vta1 | Snord73a |
|  | Acta1 | Tmem17 |  |  |  |  |  |  |  |  |  | Fcf1 | Gm11429 |
|  | Hoxaas3 | Rps11-ps1 |  |  |  |  |  |  |  |  |  | Pla2g2a | Gm23849 |
|  | Hrh1 | Rpl35a-ps3 |  |  |  |  |  |  |  |  |  | Bex3 | Gm16024 |
|  | Sybu | Rab26os |  |  |  |  |  |  |  |  |  | Clpb | Mir466i |
|  | Klhdc8a | Gm5561 |  |  |  |  |  |  |  |  |  | Pus10 | Mir192 |
|  | Sass6 | Rpl17-ps9 |  |  |  |  |  |  |  |  |  | Rapgef5 | Gm22858 |
|  | Celf5 | Gm5451 |  |  |  |  |  |  |  |  |  | Slc9a3r2 | Gm23119 |
|  | Slc43a1 | Nrgn |  |  |  |  |  |  |  |  |  | Fiz1 | Gm24610 |
|  | Upk1a | Gm2810 |  |  |  |  |  |  |  |  |  | Phgdh | Gm25328 |
|  | Ighv1-74 | Gm7765 |  |  |  |  |  |  |  |  |  | Polr1a | Gm24500 |
|  | Hoxb8 | Ighj4 |  |  |  |  |  |  |  |  |  | Rnmt | Snord65 |
|  | Igkv4-80 | Gm12551 |  |  |  |  |  |  |  |  |  | Psmd9 | Gm22079 |
|  | Gm2115 | Gm14018 |  |  |  |  |  |  |  |  |  | Abraxas2 | Gm25301 |
|  | Vsig2 | Gm4065 |  |  |  |  |  |  |  |  |  | Tmtc3 | Mir7025 |
|  | A330035P11Rik | Zfp335os |  |  |  |  |  |  |  |  |  | Xrcc5 | Gm24091 |
|  | Traf5 | A930003A15Rik |  |  |  |  |  |  |  |  |  | Fkbp3 | Gm23318 |
|  | Ighv5-9-1 | Rpl15-ps3 |  |  |  |  |  |  |  |  |  | Osbpl5 | Mir7052 |
|  | Cyb5d2 | Gm9908 |  |  |  |  |  |  |  |  |  | Trmt112 | Snord7 |
|  | Snrpn | 3110045C21Rik |  |  |  |  |  |  |  |  |  | Mipep | Gm24400 |
|  | Thnsl1 | Gm47708 |  |  |  |  |  |  |  |  |  | Lamb2 | Gm22573 |
|  | Nkain1 | Hist1h2ae |  |  |  |  |  |  |  |  |  | Uchl5 | Gm26175 |
|  | Trpt1 | Gm19610 |  |  |  |  |  |  |  |  |  | Orai1 | Gm25361 |
|  | Mfsd6l | Gm7206 |  |  |  |  |  |  |  |  |  | Uba52 | Gm11084 |
|  | Zfp953 | Gm49376 |  |  |  |  |  |  |  |  |  | Fig4 | Mir697 |
|  | Gpld1 | Gm1673 |  |  |  |  |  |  |  |  |  | Lsg1 | Gm25108 |
|  | Fam69b | Gm29560 |  |  |  |  |  |  |  |  |  | Srprb | Gm24867 |
|  | Ugt1a1 | Rpl28-ps3 |  |  |  |  |  |  |  |  |  | Wdr4 | Mir6955 |
|  | B9d2 | Gm4045 |  |  |  |  |  |  |  |  |  | Ccdc43 | Mir7049 |
|  | Gm5113 | Gm11963 |  |  |  |  |  |  |  |  |  | Manbal | Gm27616 |
|  | Ccnj | Gm10257 |  |  |  |  |  |  |  |  |  | Vrk1 | Gm24996 |
|  | Zswim7 | Rpl21-ps11 |  |  |  |  |  |  |  |  |  | Xrcc6 | Gm24776 |
|  | 4732465J04Rik | Gm9294 |  |  |  |  |  |  |  |  |  | Ppie | Mir7026 |
|  | 6820431F20Rik | Gm7600 |  |  |  |  |  |  |  |  |  | Aen | Gm27280 |
|  | Ifit3b | Gm6204 |  |  |  |  |  |  |  |  |  | Ado | Gm24261 |
|  | Arl4d | H3f3a-ps2 |  |  |  |  |  |  |  |  |  | Cdt1 | Gm22200 |
|  | 9130401M01Rik | Gm42067 |  |  |  |  |  |  |  |  |  | Zrsr2 | Gm26109 |
|  | Osr1 | Plekhd1os |  |  |  |  |  |  |  |  |  | Kat5 | Gm24379 |
|  | Hsh2d | Rps4x-ps |  |  |  |  |  |  |  |  |  | Fam45a | Gm25632 |
|  | Scnn1b | Gm31544 |  |  |  |  |  |  |  |  |  | Pop5 | Mir5119 |
|  | Slc16a9 | Gm13669 |  |  |  |  |  |  |  |  |  | Rufy3 | Gm10663 |
|  | Cdkn1c | Cbfa2t2-ps1 |  |  |  |  |  |  |  |  |  | Yrdc | mt-Ta |
|  | Ankrd37 | Gm10130 |  |  |  |  |  |  |  |  |  | Cpm | Mir7682 |
|  | Slco4a1 | Gm10051 |  |  |  |  |  |  |  |  |  | Smpd4 | Gm25772 |
|  | Nyap1 | C130046K22Rik |  |  |  |  |  |  |  |  |  | Fam193a | Mir6909 |
|  | Arhgef37 | Rps15a-ps3 |  |  |  |  |  |  |  |  |  | Kif4 | Gm17622 |
|  | Dus2 | Apoa2 |  |  |  |  |  |  |  |  |  | Otulin | Mir7041 |
|  | Mfsd9 | Gm8172 |  |  |  |  |  |  |  |  |  | Gtf3c5 | Gm17720 |
|  | Lrat | Gm6180 |  |  |  |  |  |  |  |  |  | Stard13 |  |
|  | 2810021J22Rik | Gm8226 |  |  |  |  |  |  |  |  |  | Gzf1 |  |
|  | Rnf180 | Gm10060 |  |  |  |  |  |  |  |  |  | Rhbdf1 |  |
|  | Gpr183 | Gm9396 |  |  |  |  |  |  |  |  |  | Exosc7 |  |
|  | Tpd52l1 | Gm11830 |  |  |  |  |  |  |  |  |  | Polr2d |  |
|  | Ces2b | Gm7099 |  |  |  |  |  |  |  |  |  | Usp46 |  |
|  | Ankrd39 | Gm12112 |  |  |  |  |  |  |  |  |  | Hsf1 |  |
|  | Lypd6b | Mir703 |  |  |  |  |  |  |  |  |  | Foxm1 |  |
|  | Zfp955b | Hist1h3g |  |  |  |  |  |  |  |  |  | Tesk2 |  |
|  | Slc35g2 | Gm8319 |  |  |  |  |  |  |  |  |  | Bysl |  |
|  | Cd2 | Gm10012 |  |  |  |  |  |  |  |  |  | Atpaf2 |  |
|  | Srl | Gm2830 |  |  |  |  |  |  |  |  |  | Bicral |  |
|  | Tmem144 | Gm27463 |  |  |  |  |  |  |  |  |  | Ippk |  |
|  | Tril | Rpl18-ps2 |  |  |  |  |  |  |  |  |  | Mysm1 |  |
|  | Gm43672 | Gm7392 |  |  |  |  |  |  |  |  |  | Nelfa |  |
|  | Zbtb16 | Rps15a-ps4 |  |  |  |  |  |  |  |  |  | Fam124a |  |
|  | H2-T10 | Rpl30-ps1 |  |  |  |  |  |  |  |  |  | Elac2 |  |
|  | Cdhr1 | Hist1h4n |  |  |  |  |  |  |  |  |  | Ndufaf7 |  |
|  | Oas1b | Gm13611 |  |  |  |  |  |  |  |  |  | Spc24 |  |
|  | Rpl30-ps10 | Rps12-ps10 |  |  |  |  |  |  |  |  |  | Rsl24d1 |  |
|  | Anks6 | Rps19-ps7 |  |  |  |  |  |  |  |  |  | Pdhx |  |
|  | Gm4951 | Rpl35a-ps4 |  |  |  |  |  |  |  |  |  | Fundc1 |  |
|  | Myrip | Gm48684 |  |  |  |  |  |  |  |  |  | Trmt10c |  |
|  | Zfp955a | 1810063I02Rik |  |  |  |  |  |  |  |  |  | Nup107 |  |
|  | Ephx4 | Rpl30-ps8 |  |  |  |  |  |  |  |  |  | Wdr3 |  |
|  | Gm11695 | AC122481.2 |  |  |  |  |  |  |  |  |  | Zw10 |  |
|  | Nacad | Gm47791 |  |  |  |  |  |  |  |  |  | Rbm18 |  |
|  | 9230112E08Rik | Ighj2 |  |  |  |  |  |  |  |  |  | Cdk7 |  |
|  | Gm37844 | Gm37503 |  |  |  |  |  |  |  |  |  | Heatr3 |  |
|  | 5430427M07Rik | Gm22107 |  |  |  |  |  |  |  |  |  | Ptges3-ps |  |
|  | Nt5c1a | Gm37313 |  |  |  |  |  |  |  |  |  | Arhgap39 |  |
|  | Fyb2 | Mir8091 |  |  |  |  |  |  |  |  |  | Nfxl1 |  |
|  | Cmtm8 | Gm24616 |  |  |  |  |  |  |  |  |  | Zkscan17 |  |
|  | Pbld1 | Trbj1-5 |  |  |  |  |  |  |  |  |  | Zfp64 |  |
|  | Gm7591 | Mir210 |  |  |  |  |  |  |  |  |  | Inpp5b |  |
|  | Fah | Trbj1-4 |  |  |  |  |  |  |  |  |  | Prpf4 |  |
|  | Serac1 | Trbj2-7 |  |  |  |  |  |  |  |  |  | Tssc4 |  |
|  | Dnajc28 | Mir343 |  |  |  |  |  |  |  |  |  | Ncapd3 |  |
|  | Zbtb25 | Gm22464 |  |  |  |  |  |  |  |  |  | Prmt7 |  |
|  | Lrrc61 |  |  |  |  |  |  |  |  |  |  | Snx11 |  |
|  | Icam2 |  |  |  |  |  |  |  |  |  |  | 1700066B19Rik |  |
|  | mt-Nd4l |  |  |  |  |  |  |  |  |  |  | Ddx49 |  |
|  | Scgn |  |  |  |  |  |  |  |  |  |  | Nol12 |  |
|  | Gm1965 |  |  |  |  |  |  |  |  |  |  | Phf6 |  |
|  | Chst14 |  |  |  |  |  |  |  |  |  |  | Prps1l3 |  |
|  | Rtl8b |  |  |  |  |  |  |  |  |  |  | Polr2g |  |
|  | Olfr56 |  |  |  |  |  |  |  |  |  |  | Smpd1 |  |
|  | Tcf23 |  |  |  |  |  |  |  |  |  |  | Gtf2f2 |  |
|  | Zfp759 |  |  |  |  |  |  |  |  |  |  | Polr3b |  |
|  | Acot2 |  |  |  |  |  |  |  |  |  |  | Pcp4l1 |  |
|  | Gpr39 |  |  |  |  |  |  |  |  |  |  | Bri3bp |  |
|  | D330045A20Rik |  |  |  |  |  |  |  |  |  |  | Ndc1 |  |
|  | Rmdn2 |  |  |  |  |  |  |  |  |  |  | Pex26 |  |
|  | Vnn1 |  |  |  |  |  |  |  |  |  |  | Tysnd1 |  |
|  | Tmem138 |  |  |  |  |  |  |  |  |  |  | Ipo11 |  |
|  | Hoxb4 |  |  |  |  |  |  |  |  |  |  | Mthfd1l |  |
|  | Hoxb5 |  |  |  |  |  |  |  |  |  |  | Nr2c2 |  |
|  | 9430091E24Rik |  |  |  |  |  |  |  |  |  |  | Fgfr1op |  |
|  | 1700020L24Rik |  |  |  |  |  |  |  |  |  |  | Ocrl |  |
|  | Dnase1l2 |  |  |  |  |  |  |  |  |  |  | Uchl3 |  |
|  | Bend7 |  |  |  |  |  |  |  |  |  |  | Mospd3 |  |
|  | Arl6 |  |  |  |  |  |  |  |  |  |  | Efl1 |  |
|  | Rai2 |  |  |  |  |  |  |  |  |  |  | Ttc19 |  |
|  | 2610524H06Rik |  |  |  |  |  |  |  |  |  |  | E130309D02Rik |  |
|  | Mt3 |  |  |  |  |  |  |  |  |  |  | Wdr83os |  |
|  | Klf15 |  |  |  |  |  |  |  |  |  |  | Naa25 |  |
|  | Zbtb32 |  |  |  |  |  |  |  |  |  |  | Faah |  |
|  | 2810002D19Rik |  |  |  |  |  |  |  |  |  |  | Utp14a |  |
|  | Cnbd2 |  |  |  |  |  |  |  |  |  |  | Ints11 |  |
|  | Prkcz2 |  |  |  |  |  |  |  |  |  |  | A430005L14Rik |  |
|  | Gm15998 |  |  |  |  |  |  |  |  |  |  | Nedd1 |  |
|  | Gm13340 |  |  |  |  |  |  |  |  |  |  | Snip1 |  |
|  | Igkv6-13 |  |  |  |  |  |  |  |  |  |  | Tbcel |  |
|  | Gm28875 |  |  |  |  |  |  |  |  |  |  | Esf1 |  |
|  | Zfp93 |  |  |  |  |  |  |  |  |  |  | Fam207a |  |
|  | Neurl1a |  |  |  |  |  |  |  |  |  |  | Nup155 |  |
|  | Efcab2 |  |  |  |  |  |  |  |  |  |  | Exosc9 |  |
|  | Gdf11 |  |  |  |  |  |  |  |  |  |  | Ckap2l |  |
|  | Otud1 |  |  |  |  |  |  |  |  |  |  | Fpgs |  |
|  | Slc13a2os |  |  |  |  |  |  |  |  |  |  | Nup214 |  |
|  | Ighv10-3 |  |  |  |  |  |  |  |  |  |  | Casc4 |  |
|  | Sox21 |  |  |  |  |  |  |  |  |  |  | Pus1 |  |
|  | Nags |  |  |  |  |  |  |  |  |  |  | Wwc2 |  |
|  | Nradd |  |  |  |  |  |  |  |  |  |  | Dmac1 |  |
|  | Foxl1 |  |  |  |  |  |  |  |  |  |  | Zbtb6 |  |
|  | Btnl7-ps |  |  |  |  |  |  |  |  |  |  | Lsm2 |  |
|  | Selenbp2 |  |  |  |  |  |  |  |  |  |  | Cdkn2c |  |
|  | Afmid |  |  |  |  |  |  |  |  |  |  | Bag5 |  |
|  | 1810010D01Rik |  |  |  |  |  |  |  |  |  |  | Hpf1 |  |
|  | Tmem150a |  |  |  |  |  |  |  |  |  |  | Foxj2 |  |
|  | Bmp8a |  |  |  |  |  |  |  |  |  |  | Prps1 |  |
|  | Hoxd8 |  |  |  |  |  |  |  |  |  |  | Leo1 |  |
|  | Amn |  |  |  |  |  |  |  |  |  |  | Fam50a |  |
|  | Gm37254 |  |  |  |  |  |  |  |  |  |  | Slc25a38 |  |
|  | Fndc5 |  |  |  |  |  |  |  |  |  |  | Nubp1 |  |
|  | Depp1 |  |  |  |  |  |  |  |  |  |  | Utp18 |  |
|  | Zc3h8 |  |  |  |  |  |  |  |  |  |  | Paqr8 |  |
|  | Fam151b |  |  |  |  |  |  |  |  |  |  | Tbl3 |  |
|  | 1110019D14Rik |  |  |  |  |  |  |  |  |  |  | Rcc1l |  |
|  | Slc23a3 |  |  |  |  |  |  |  |  |  |  | Gpn3 |  |
|  | Tmem25 |  |  |  |  |  |  |  |  |  |  | Inip |  |
|  | Rgs13 |  |  |  |  |  |  |  |  |  |  | Polr1b |  |
|  | Tnfrsf18 |  |  |  |  |  |  |  |  |  |  | Usb1 |  |
|  | Acaa1b |  |  |  |  |  |  |  |  |  |  | Tut4 |  |
|  | Gm9776 |  |  |  |  |  |  |  |  |  |  | Pelp1 |  |
|  | Zfp383 |  |  |  |  |  |  |  |  |  |  | Mfsd11 |  |
|  | Esm1 |  |  |  |  |  |  |  |  |  |  | Riok2 |  |
|  | Gm48529 |  |  |  |  |  |  |  |  |  |  | Pold1 |  |
|  | Fv1 |  |  |  |  |  |  |  |  |  |  | Hmgxb4 |  |
|  | AU041133 |  |  |  |  |  |  |  |  |  |  | Mettl16 |  |
|  | Gm11437 |  |  |  |  |  |  |  |  |  |  | Ncaph |  |
|  | 1700010I14Rik |  |  |  |  |  |  |  |  |  |  | Kifc1 |  |
|  | Gm11496 |  |  |  |  |  |  |  |  |  |  | Zfand2a |  |
|  | 9930012K11Rik |  |  |  |  |  |  |  |  |  |  | Ptk6 |  |
|  | Zcchc3 |  |  |  |  |  |  |  |  |  |  | Bbx |  |
|  | Zfp563 |  |  |  |  |  |  |  |  |  |  | Nbn |  |
|  | Rtn4r |  |  |  |  |  |  |  |  |  |  | Ptrh2 |  |
|  | Dusp14 |  |  |  |  |  |  |  |  |  |  | Ecsit |  |
|  | AI427809 |  |  |  |  |  |  |  |  |  |  | Prune1 |  |
|  | Ckm |  |  |  |  |  |  |  |  |  |  | Itgb6 |  |
|  | P2ry12 |  |  |  |  |  |  |  |  |  |  | Pknox1 |  |
|  | Gm5547 |  |  |  |  |  |  |  |  |  |  | Fbxo45 |  |
|  | Gm6485 |  |  |  |  |  |  |  |  |  |  | Utp4 |  |
|  | Odf3b |  |  |  |  |  |  |  |  |  |  | Dars2 |  |
|  | E230001N04Rik |  |  |  |  |  |  |  |  |  |  | Depdc5 |  |
|  | Gm6741 |  |  |  |  |  |  |  |  |  |  | Galns |  |
|  | Sh2d7 |  |  |  |  |  |  |  |  |  |  | Tsku |  |
|  | Mfsd4b3 |  |  |  |  |  |  |  |  |  |  | Spag5 |  |
|  | Sh2d6 |  |  |  |  |  |  |  |  |  |  | Lsm1 |  |
|  | Gm10222 |  |  |  |  |  |  |  |  |  |  | Gbp7 |  |
|  | Hoxb5os |  |  |  |  |  |  |  |  |  |  | Fam53b |  |
|  | Cyp2c69 |  |  |  |  |  |  |  |  |  |  | Gpx4-ps2 |  |
|  | Batf3 |  |  |  |  |  |  |  |  |  |  | Tut1 |  |
|  | Cfap52 |  |  |  |  |  |  |  |  |  |  | Commd2 |  |
|  | Ciart |  |  |  |  |  |  |  |  |  |  | Dnajb4 |  |
|  | Gm16437 |  |  |  |  |  |  |  |  |  |  | Cd99l2 |  |
|  | Smim10l2a |  |  |  |  |  |  |  |  |  |  | Mrps22 |  |
|  | Efna3 |  |  |  |  |  |  |  |  |  |  | Daglb |  |
|  | AC125351.1 |  |  |  |  |  |  |  |  |  |  | Gm9824 |  |
|  | 5730507C01Rik |  |  |  |  |  |  |  |  |  |  | Taf11 |  |
|  | Gm43197 |  |  |  |  |  |  |  |  |  |  | Taf8 |  |
|  | Prss27 |  |  |  |  |  |  |  |  |  |  | Utp15 |  |
|  | Golt1a |  |  |  |  |  |  |  |  |  |  | Pqbp1 |  |
|  | Gna15 |  |  |  |  |  |  |  |  |  |  | Tubgcp4 |  |
|  | Ccdc69 |  |  |  |  |  |  |  |  |  |  | Tubb2a |  |
|  | Rnf183 |  |  |  |  |  |  |  |  |  |  | Tead3 |  |
|  | CAAA01147332.1 |  |  |  |  |  |  |  |  |  |  | Gm10131 |  |
|  | Msl3l2 |  |  |  |  |  |  |  |  |  |  | Bcl9 |  |
|  | Gm14097 |  |  |  |  |  |  |  |  |  |  | Nop14 |  |
|  | Pgam2 |  |  |  |  |  |  |  |  |  |  | Wdr55 |  |
|  | 2700038G22Rik |  |  |  |  |  |  |  |  |  |  | Blzf1 |  |
|  | Ckmt2 |  |  |  |  |  |  |  |  |  |  | Ogfod1 |  |
|  | Akr1c18 |  |  |  |  |  |  |  |  |  |  | Vsig1 |  |
|  | Gm28588 |  |  |  |  |  |  |  |  |  |  | Pym1 |  |
|  | Igkv8-18 |  |  |  |  |  |  |  |  |  |  | Habp4 |  |
|  | Snora57 |  |  |  |  |  |  |  |  |  |  | Noc4l |  |
|  | Gm16589 |  |  |  |  |  |  |  |  |  |  | Nol11 |  |
|  | 0610040F04Rik |  |  |  |  |  |  |  |  |  |  | Kif15 |  |
|  | Gm15368 |  |  |  |  |  |  |  |  |  |  | Nlrx1 |  |
|  | Ighv1-36 |  |  |  |  |  |  |  |  |  |  | Xrcc1 |  |
|  | Gm33424 |  |  |  |  |  |  |  |  |  |  | Cep55 |  |
|  | Gm16464 |  |  |  |  |  |  |  |  |  |  | Nif3l1 |  |
|  | Itln1 |  |  |  |  |  |  |  |  |  |  | Exosc8 |  |
|  | 9130410C08Rik |  |  |  |  |  |  |  |  |  |  | Calcoco1 |  |
|  | Gm13657 |  |  |  |  |  |  |  |  |  |  | Myg1 |  |
|  | Gm49282 |  |  |  |  |  |  |  |  |  |  | Bcap29 |  |
|  | Gm15133 |  |  |  |  |  |  |  |  |  |  | Supv3l1 |  |
|  | 4930447F24Rik |  |  |  |  |  |  |  |  |  |  | Mre11a |  |
|  | Gm13803 |  |  |  |  |  |  |  |  |  |  | Lmod1 |  |
|  | Gm5611 |  |  |  |  |  |  |  |  |  |  | Mrpl1 |  |
|  | Proc |  |  |  |  |  |  |  |  |  |  | Mrpl47 |  |
|  | 1700016K19Rik |  |  |  |  |  |  |  |  |  |  | Pola2 |  |
|  | Gm8069 |  |  |  |  |  |  |  |  |  |  | Gpatch4 |  |
|  | Gm10522 |  |  |  |  |  |  |  |  |  |  | Mettl1 |  |
|  | Zbtb11os1 |  |  |  |  |  |  |  |  |  |  | Rpa2 |  |
|  | Gm11739 |  |  |  |  |  |  |  |  |  |  | Auh |  |
|  | Gm39556 |  |  |  |  |  |  |  |  |  |  | Zer1 |  |
|  | Gm9887 |  |  |  |  |  |  |  |  |  |  | Zmat3 |  |
|  | Gm13552 |  |  |  |  |  |  |  |  |  |  | Wdr77 |  |
|  | Gm9001 |  |  |  |  |  |  |  |  |  |  | Trim59 |  |
|  | Gm12352 |  |  |  |  |  |  |  |  |  |  | Pop4 |  |
|  | Mir8116 |  |  |  |  |  |  |  |  |  |  | Znhit2 |  |
|  | Gm45552 |  |  |  |  |  |  |  |  |  |  | Mrpl22 |  |
|  | Gm9790 |  |  |  |  |  |  |  |  |  |  | Cks1b |  |
|  | AC122217.2 |  |  |  |  |  |  |  |  |  |  | Tedc1 |  |
|  | Gm8221 |  |  |  |  |  |  |  |  |  |  | E2f8 |  |
|  | Gm13160 |  |  |  |  |  |  |  |  |  |  | Gm11847 |  |
|  | Urad |  |  |  |  |  |  |  |  |  |  | Twistnb |  |
|  | Appbp2os |  |  |  |  |  |  |  |  |  |  | Pspc1 |  |
|  | Gm6483 |  |  |  |  |  |  |  |  |  |  | Terf2ip |  |
|  | Gm6419 |  |  |  |  |  |  |  |  |  |  | Mpp7 |  |
|  | Gm16124 |  |  |  |  |  |  |  |  |  |  | Pbdc1 |  |
|  | Hist1h2ao |  |  |  |  |  |  |  |  |  |  | Cln3 |  |
|  | Nmb |  |  |  |  |  |  |  |  |  |  | Shprh |  |
|  | Gm26964 |  |  |  |  |  |  |  |  |  |  | Nt5dc1 |  |
|  | Gm36107 |  |  |  |  |  |  |  |  |  |  | Riok1 |  |
|  | Ripply1 |  |  |  |  |  |  |  |  |  |  | Mtap |  |
|  | Gm10651 |  |  |  |  |  |  |  |  |  |  | Dhfr |  |
|  | Defb37 |  |  |  |  |  |  |  |  |  |  | Gpr137 |  |
|  | Gm12506 |  |  |  |  |  |  |  |  |  |  | 0610010F05Rik |  |
|  | 3100003L05Rik |  |  |  |  |  |  |  |  |  |  | Mycbp |  |
|  | 2010001M07Rik |  |  |  |  |  |  |  |  |  |  | Mbtps2 |  |
|  | Gm12928 |  |  |  |  |  |  |  |  |  |  | Dis3 |  |
|  | Gm18284 |  |  |  |  |  |  |  |  |  |  | Lsm3 |  |
|  | Gm31363 |  |  |  |  |  |  |  |  |  |  | Gemin5 |  |
|  | Gm10478 |  |  |  |  |  |  |  |  |  |  | Rnaseh2b |  |
|  | Gm15502 |  |  |  |  |  |  |  |  |  |  | Gins4 |  |
|  | Tpd52-ps |  |  |  |  |  |  |  |  |  |  | Grhpr |  |
|  | Gm6969 |  |  |  |  |  |  |  |  |  |  | Arhgap19 |  |
|  | Gm13412 |  |  |  |  |  |  |  |  |  |  | Polrmt |  |
|  | Gm39132 |  |  |  |  |  |  |  |  |  |  | Dhps |  |
|  | 9030616G12Rik |  |  |  |  |  |  |  |  |  |  | Dis3l |  |
|  | Gm12252 |  |  |  |  |  |  |  |  |  |  | Nudt5 |  |
|  | Fam183b |  |  |  |  |  |  |  |  |  |  | Nup133 |  |
|  | Gm21769 |  |  |  |  |  |  |  |  |  |  | Aven |  |
|  | Gm9027 |  |  |  |  |  |  |  |  |  |  | Fadd |  |
|  | Gm15432 |  |  |  |  |  |  |  |  |  |  | Zswim6 |  |
|  | Gm10157 |  |  |  |  |  |  |  |  |  |  | Mettl14 |  |
|  | S100a4 |  |  |  |  |  |  |  |  |  |  | Gadd45gip1 |  |
|  | Gm11964 |  |  |  |  |  |  |  |  |  |  | Tubg1 |  |
|  | Gm8066 |  |  |  |  |  |  |  |  |  |  | Iqgap3 |  |
|  | Scgb2b15 |  |  |  |  |  |  |  |  |  |  | Ccnh |  |
|  | Gm30613 |  |  |  |  |  |  |  |  |  |  | Blvra |  |
|  | Gm11752 |  |  |  |  |  |  |  |  |  |  | Eipr1 |  |
|  | Gm10240 |  |  |  |  |  |  |  |  |  |  | Mtmr7 |  |
|  | Xcl1 |  |  |  |  |  |  |  |  |  |  | Ddx20 |  |
|  | Gm8508 |  |  |  |  |  |  |  |  |  |  | Cyp20a1 |  |
|  | Gm44507 |  |  |  |  |  |  |  |  |  |  | Aldh7a1 |  |
|  | AC122821.1 |  |  |  |  |  |  |  |  |  |  | Timmdc1 |  |
|  | Gm21297 |  |  |  |  |  |  |  |  |  |  | Polr3d |  |
|  | Gm13071 |  |  |  |  |  |  |  |  |  |  | Flywch1 |  |
|  | Scgb1b3 |  |  |  |  |  |  |  |  |  |  | Rad51 |  |
|  | Gm14567 |  |  |  |  |  |  |  |  |  |  | Etfrf1 |  |
|  | Gm4540 |  |  |  |  |  |  |  |  |  |  | Fastkd2 |  |
|  | AC125149.1 |  |  |  |  |  |  |  |  |  |  | Rbm34 |  |
|  | Gm17330 |  |  |  |  |  |  |  |  |  |  | Grwd1 |  |
|  | Gm24727 |  |  |  |  |  |  |  |  |  |  | Tcf19 |  |
|  | Gm25596 |  |  |  |  |  |  |  |  |  |  | Hikeshi |  |
|  | Gm46515 |  |  |  |  |  |  |  |  |  |  | Klhl12 |  |
|  | Gm44216 |  |  |  |  |  |  |  |  |  |  | Samd1 |  |
|  | Gm44954 |  |  |  |  |  |  |  |  |  |  | Ube2d1 |  |
|  | Gm26424 |  |  |  |  |  |  |  |  |  |  | Noa1 |  |
|  | Mir7027 |  |  |  |  |  |  |  |  |  |  | Pias3 |  |
|  | Gm23123 |  |  |  |  |  |  |  |  |  |  | Ankrd54 |  |
|  | Gm26254 |  |  |  |  |  |  |  |  |  |  | Gm5527 |  |
|  | Trgj4 |  |  |  |  |  |  |  |  |  |  | Haus4 |  |
|  | Gm23264 |  |  |  |  |  |  |  |  |  |  | Exoc8 |  |
|  | Gm25965 |  |  |  |  |  |  |  |  |  |  | Knl1 |  |
|  | Gm23564 |  |  |  |  |  |  |  |  |  |  | Tiam1 |  |
|  | Mir3058 |  |  |  |  |  |  |  |  |  |  | Ndufaf3 |  |
|  | mt-Ti |  |  |  |  |  |  |  |  |  |  | Pwwp2b |  |
|  | Traj1 |  |  |  |  |  |  |  |  |  |  | Lrig2 |  |
|  |  |  |  |  |  |  |  |  |  |  |  | Orai3 |  |
|  |  |  |  |  |  |  |  |  |  |  |  | Prpf3 |  |
|  |  |  |  |  |  |  |  |  |  |  |  | Terf1 |  |
|  |  |  |  |  |  |  |  |  |  |  |  | Nagpa |  |
|  |  |  |  |  |  |  |  |  |  |  |  | Smpd2 |  |
|  |  |  |  |  |  |  |  |  |  |  |  | St7l |  |
|  |  |  |  |  |  |  |  |  |  |  |  | Rab33b |  |
|  |  |  |  |  |  |  |  |  |  |  |  | Phactr2 |  |
|  |  |  |  |  |  |  |  |  |  |  |  | Gmeb1 |  |
|  |  |  |  |  |  |  |  |  |  |  |  | Ncapg |  |
|  |  |  |  |  |  |  |  |  |  |  |  | Rtel1 |  |
|  |  |  |  |  |  |  |  |  |  |  |  | Gatc |  |
|  |  |  |  |  |  |  |  |  |  |  |  | BC025446 |  |
|  |  |  |  |  |  |  |  |  |  |  |  | Hirip3 |  |
|  |  |  |  |  |  |  |  |  |  |  |  | Siah1a |  |
|  |  |  |  |  |  |  |  |  |  |  |  | Dbr1 |  |
|  |  |  |  |  |  |  |  |  |  |  |  | Dlgap5 |  |
|  |  |  |  |  |  |  |  |  |  |  |  | Rfc3 |  |
|  |  |  |  |  |  |  |  |  |  |  |  | Champ1 |  |
|  |  |  |  |  |  |  |  |  |  |  |  | Fbxo4 |  |
|  |  |  |  |  |  |  |  |  |  |  |  | L3mbtl2 |  |
|  |  |  |  |  |  |  |  |  |  |  |  | Stx18 |  |
|  |  |  |  |  |  |  |  |  |  |  |  | Gng11 |  |
|  |  |  |  |  |  |  |  |  |  |  |  | Fam241a |  |
|  |  |  |  |  |  |  |  |  |  |  |  | Haus2 |  |
|  |  |  |  |  |  |  |  |  |  |  |  | Cdk5rap2 |  |
|  |  |  |  |  |  |  |  |  |  |  |  | Med4 |  |
|  |  |  |  |  |  |  |  |  |  |  |  | Mthfr |  |
|  |  |  |  |  |  |  |  |  |  |  |  | Rrp9 |  |
|  |  |  |  |  |  |  |  |  |  |  |  | Hipk2 |  |
|  |  |  |  |  |  |  |  |  |  |  |  | Zmym4 |  |
|  |  |  |  |  |  |  |  |  |  |  |  | Arsa |  |
|  |  |  |  |  |  |  |  |  |  |  |  | 2410004B18Rik |  |
|  |  |  |  |  |  |  |  |  |  |  |  | Rps19bp1 |  |
|  |  |  |  |  |  |  |  |  |  |  |  | Crebl2 |  |
|  |  |  |  |  |  |  |  |  |  |  |  | Cfap97 |  |
|  |  |  |  |  |  |  |  |  |  |  |  | Ccnf |  |
|  |  |  |  |  |  |  |  |  |  |  |  | Mthfd2 |  |
|  |  |  |  |  |  |  |  |  |  |  |  | Ino80e |  |
|  |  |  |  |  |  |  |  |  |  |  |  | Galnt11 |  |
|  |  |  |  |  |  |  |  |  |  |  |  | Lnpk |  |
|  |  |  |  |  |  |  |  |  |  |  |  | Jag1 |  |
|  |  |  |  |  |  |  |  |  |  |  |  | Rfng |  |
|  |  |  |  |  |  |  |  |  |  |  |  | Armt1 |  |
|  |  |  |  |  |  |  |  |  |  |  |  | Gtpbp3 |  |
|  |  |  |  |  |  |  |  |  |  |  |  | Eef1e1 |  |
|  |  |  |  |  |  |  |  |  |  |  |  | Mios |  |
|  |  |  |  |  |  |  |  |  |  |  |  | Ppp1r14a |  |
|  |  |  |  |  |  |  |  |  |  |  |  | Nol8 |  |
|  |  |  |  |  |  |  |  |  |  |  |  | Aasdhppt |  |
|  |  |  |  |  |  |  |  |  |  |  |  | Exosc4 |  |
|  |  |  |  |  |  |  |  |  |  |  |  | Spc25 |  |
|  |  |  |  |  |  |  |  |  |  |  |  | Grem2 |  |
|  |  |  |  |  |  |  |  |  |  |  |  | Znrd1 |  |
|  |  |  |  |  |  |  |  |  |  |  |  | Polr2i |  |
|  |  |  |  |  |  |  |  |  |  |  |  | Slc4a1ap |  |
|  |  |  |  |  |  |  |  |  |  |  |  | Vps45 |  |
|  |  |  |  |  |  |  |  |  |  |  |  | Dhx29 |  |
|  |  |  |  |  |  |  |  |  |  |  |  | Zfp800 |  |
|  |  |  |  |  |  |  |  |  |  |  |  | Katnbl1 |  |
|  |  |  |  |  |  |  |  |  |  |  |  | Rwdd2b |  |
|  |  |  |  |  |  |  |  |  |  |  |  | Clp1 |  |
|  |  |  |  |  |  |  |  |  |  |  |  | Asb13 |  |
|  |  |  |  |  |  |  |  |  |  |  |  | Suv39h1 |  |
|  |  |  |  |  |  |  |  |  |  |  |  | Dcakd |  |
|  |  |  |  |  |  |  |  |  |  |  |  | Ints9 |  |
|  |  |  |  |  |  |  |  |  |  |  |  | Tigar |  |
|  |  |  |  |  |  |  |  |  |  |  |  | Rngtt |  |
|  |  |  |  |  |  |  |  |  |  |  |  | Asf1b |  |
|  |  |  |  |  |  |  |  |  |  |  |  | Pfas |  |
|  |  |  |  |  |  |  |  |  |  |  |  | Ahdc1 |  |
|  |  |  |  |  |  |  |  |  |  |  |  | Dnase1l1 |  |
|  |  |  |  |  |  |  |  |  |  |  |  | Ago3 |  |
|  |  |  |  |  |  |  |  |  |  |  |  | Aspm |  |
|  |  |  |  |  |  |  |  |  |  |  |  | Nxt1 |  |
|  |  |  |  |  |  |  |  |  |  |  |  | Ints2 |  |
|  |  |  |  |  |  |  |  |  |  |  |  | Cbfa2t2 |  |
|  |  |  |  |  |  |  |  |  |  |  |  | Pdp1 |  |
|  |  |  |  |  |  |  |  |  |  |  |  | Mis18a |  |
|  |  |  |  |  |  |  |  |  |  |  |  | Ntmt1 |  |
|  |  |  |  |  |  |  |  |  |  |  |  | Gtf2h2 |  |
|  |  |  |  |  |  |  |  |  |  |  |  | Gm5641 |  |
|  |  |  |  |  |  |  |  |  |  |  |  | Plk4 |  |
|  |  |  |  |  |  |  |  |  |  |  |  | Kif20a |  |
|  |  |  |  |  |  |  |  |  |  |  |  | Urb2 |  |
|  |  |  |  |  |  |  |  |  |  |  |  | Sap18b |  |
|  |  |  |  |  |  |  |  |  |  |  |  | Haus8 |  |
|  |  |  |  |  |  |  |  |  |  |  |  | Orc2 |  |
|  |  |  |  |  |  |  |  |  |  |  |  | Cops7b |  |
|  |  |  |  |  |  |  |  |  |  |  |  | 1110008P14Rik |  |
|  |  |  |  |  |  |  |  |  |  |  |  | Med11 |  |
|  |  |  |  |  |  |  |  |  |  |  |  | Ung |  |
|  |  |  |  |  |  |  |  |  |  |  |  | Mospd1 |  |
|  |  |  |  |  |  |  |  |  |  |  |  | Gab1 |  |
|  |  |  |  |  |  |  |  |  |  |  |  | Suox |  |
|  |  |  |  |  |  |  |  |  |  |  |  | Mcm10 |  |
|  |  |  |  |  |  |  |  |  |  |  |  | Yars2 |  |
|  |  |  |  |  |  |  |  |  |  |  |  | Itpkc |  |
|  |  |  |  |  |  |  |  |  |  |  |  | Rrp1b |  |
|  |  |  |  |  |  |  |  |  |  |  |  | Gm17018 |  |
|  |  |  |  |  |  |  |  |  |  |  |  | Med6 |  |
|  |  |  |  |  |  |  |  |  |  |  |  | Espl1 |  |
|  |  |  |  |  |  |  |  |  |  |  |  | Bcl2 |  |
|  |  |  |  |  |  |  |  |  |  |  |  | Mphosph6 |  |
|  |  |  |  |  |  |  |  |  |  |  |  | Tmem185a |  |
|  |  |  |  |  |  |  |  |  |  |  |  | Rarg |  |
|  |  |  |  |  |  |  |  |  |  |  |  | Mlh1 |  |
|  |  |  |  |  |  |  |  |  |  |  |  | Mrps11 |  |
|  |  |  |  |  |  |  |  |  |  |  |  | Riox2 |  |
|  |  |  |  |  |  |  |  |  |  |  |  | B4gat1 |  |
|  |  |  |  |  |  |  |  |  |  |  |  | Rpf2 |  |
|  |  |  |  |  |  |  |  |  |  |  |  | Sms |  |
|  |  |  |  |  |  |  |  |  |  |  |  | Myef2 |  |
|  |  |  |  |  |  |  |  |  |  |  |  | Zbed3 |  |
|  |  |  |  |  |  |  |  |  |  |  |  | Bola1 |  |
|  |  |  |  |  |  |  |  |  |  |  |  | Me1 |  |
|  |  |  |  |  |  |  |  |  |  |  |  | Ypel2 |  |
|  |  |  |  |  |  |  |  |  |  |  |  | Acacb |  |
|  |  |  |  |  |  |  |  |  |  |  |  | Psmg1 |  |
|  |  |  |  |  |  |  |  |  |  |  |  | Chd1l |  |
|  |  |  |  |  |  |  |  |  |  |  |  | Gnai1 |  |
|  |  |  |  |  |  |  |  |  |  |  |  | Tmem251 |  |
|  |  |  |  |  |  |  |  |  |  |  |  | Vps9d1 |  |
|  |  |  |  |  |  |  |  |  |  |  |  | Bub1 |  |
|  |  |  |  |  |  |  |  |  |  |  |  | Ftsj1 |  |
|  |  |  |  |  |  |  |  |  |  |  |  | Wfdc18 |  |
|  |  |  |  |  |  |  |  |  |  |  |  | Chaf1a |  |
|  |  |  |  |  |  |  |  |  |  |  |  | Ctu2 |  |
|  |  |  |  |  |  |  |  |  |  |  |  | Wdr12 |  |
|  |  |  |  |  |  |  |  |  |  |  |  | Slc36a4 |  |
|  |  |  |  |  |  |  |  |  |  |  |  | Wdhd1 |  |
|  |  |  |  |  |  |  |  |  |  |  |  | Myo9a |  |
|  |  |  |  |  |  |  |  |  |  |  |  | Tmem125 |  |
|  |  |  |  |  |  |  |  |  |  |  |  | Gpn1 |  |
|  |  |  |  |  |  |  |  |  |  |  |  | Aaas |  |
|  |  |  |  |  |  |  |  |  |  |  |  | Ormdl1 |  |
|  |  |  |  |  |  |  |  |  |  |  |  | Mob3c |  |
|  |  |  |  |  |  |  |  |  |  |  |  | Tamm41 |  |
|  |  |  |  |  |  |  |  |  |  |  |  | Zfp319 |  |
|  |  |  |  |  |  |  |  |  |  |  |  | B4galt7 |  |
|  |  |  |  |  |  |  |  |  |  |  |  | Gucy1a1 |  |
|  |  |  |  |  |  |  |  |  |  |  |  | Poc1a |  |
|  |  |  |  |  |  |  |  |  |  |  |  | Lepr |  |
|  |  |  |  |  |  |  |  |  |  |  |  | Arhgap24 |  |
|  |  |  |  |  |  |  |  |  |  |  |  | Gm10052 |  |
|  |  |  |  |  |  |  |  |  |  |  |  | Ctc1 |  |
|  |  |  |  |  |  |  |  |  |  |  |  | Proser1 |  |
|  |  |  |  |  |  |  |  |  |  |  |  | Oplah |  |
|  |  |  |  |  |  |  |  |  |  |  |  | Arfip1 |  |
|  |  |  |  |  |  |  |  |  |  |  |  | Pola1 |  |
|  |  |  |  |  |  |  |  |  |  |  |  | Gm4366 |  |
|  |  |  |  |  |  |  |  |  |  |  |  | Smarcd1 |  |
|  |  |  |  |  |  |  |  |  |  |  |  | Tipin |  |
|  |  |  |  |  |  |  |  |  |  |  |  | Ccdc86 |  |
|  |  |  |  |  |  |  |  |  |  |  |  | Klhl7 |  |
|  |  |  |  |  |  |  |  |  |  |  |  | Zfp866 |  |
|  |  |  |  |  |  |  |  |  |  |  |  | 5031425E22Rik |  |
|  |  |  |  |  |  |  |  |  |  |  |  | Snx33 |  |
|  |  |  |  |  |  |  |  |  |  |  |  | Pwp2 |  |
|  |  |  |  |  |  |  |  |  |  |  |  | Smyd5 |  |
|  |  |  |  |  |  |  |  |  |  |  |  | Orc5 |  |
|  |  |  |  |  |  |  |  |  |  |  |  | Slco2b1 |  |
|  |  |  |  |  |  |  |  |  |  |  |  | Thap7 |  |
|  |  |  |  |  |  |  |  |  |  |  |  | Hs3st1 |  |
|  |  |  |  |  |  |  |  |  |  |  |  | Eef1akmt1 |  |
|  |  |  |  |  |  |  |  |  |  |  |  | Zdhhc16 |  |
|  |  |  |  |  |  |  |  |  |  |  |  | Spout1 |  |
|  |  |  |  |  |  |  |  |  |  |  |  | F8a |  |
|  |  |  |  |  |  |  |  |  |  |  |  | Smn1 |  |
|  |  |  |  |  |  |  |  |  |  |  |  | Wdr92 |  |
|  |  |  |  |  |  |  |  |  |  |  |  | Timm9 |  |
|  |  |  |  |  |  |  |  |  |  |  |  | Ptpn21 |  |
|  |  |  |  |  |  |  |  |  |  |  |  | Siva1 |  |
|  |  |  |  |  |  |  |  |  |  |  |  | Rps6kb2 |  |
|  |  |  |  |  |  |  |  |  |  |  |  | Prim1 |  |
|  |  |  |  |  |  |  |  |  |  |  |  | Snapc2 |  |
|  |  |  |  |  |  |  |  |  |  |  |  | Nup35 |  |
|  |  |  |  |  |  |  |  |  |  |  |  | Card14 |  |
|  |  |  |  |  |  |  |  |  |  |  |  | Lrwd1 |  |
|  |  |  |  |  |  |  |  |  |  |  |  | Nuf2 |  |
|  |  |  |  |  |  |  |  |  |  |  |  | Rhno1 |  |
|  |  |  |  |  |  |  |  |  |  |  |  | Pogk |  |
|  |  |  |  |  |  |  |  |  |  |  |  | Aatf |  |
|  |  |  |  |  |  |  |  |  |  |  |  | H2-Q1 |  |
|  |  |  |  |  |  |  |  |  |  |  |  | Kif2c |  |
|  |  |  |  |  |  |  |  |  |  |  |  | Dcun1d4 |  |
|  |  |  |  |  |  |  |  |  |  |  |  | Ubiad1 |  |
|  |  |  |  |  |  |  |  |  |  |  |  | Kifap3 |  |
|  |  |  |  |  |  |  |  |  |  |  |  | Katnb1 |  |
|  |  |  |  |  |  |  |  |  |  |  |  | Faap100 |  |
|  |  |  |  |  |  |  |  |  |  |  |  | Orc6 |  |
|  |  |  |  |  |  |  |  |  |  |  |  | Smyd2 |  |
|  |  |  |  |  |  |  |  |  |  |  |  | Exosc1 |  |
|  |  |  |  |  |  |  |  |  |  |  |  | Peli2 |  |
|  |  |  |  |  |  |  |  |  |  |  |  | Qtrt1 |  |
|  |  |  |  |  |  |  |  |  |  |  |  | Pop7 |  |
|  |  |  |  |  |  |  |  |  |  |  |  | Mrrf |  |
|  |  |  |  |  |  |  |  |  |  |  |  | Trp53rka |  |
|  |  |  |  |  |  |  |  |  |  |  |  | Sac3d1 |  |
|  |  |  |  |  |  |  |  |  |  |  |  | Mmd |  |
|  |  |  |  |  |  |  |  |  |  |  |  | Reep6 |  |
|  |  |  |  |  |  |  |  |  |  |  |  | D030056L22Rik |  |
|  |  |  |  |  |  |  |  |  |  |  |  | Zbed4 |  |
|  |  |  |  |  |  |  |  |  |  |  |  | Rrp12 |  |
|  |  |  |  |  |  |  |  |  |  |  |  | Urah |  |
|  |  |  |  |  |  |  |  |  |  |  |  | Ipo4 |  |
|  |  |  |  |  |  |  |  |  |  |  |  | Abt1 |  |
|  |  |  |  |  |  |  |  |  |  |  |  | Xylt2 |  |
|  |  |  |  |  |  |  |  |  |  |  |  | Cmc2 |  |
|  |  |  |  |  |  |  |  |  |  |  |  | Fam214a |  |
|  |  |  |  |  |  |  |  |  |  |  |  | Spryd4 |  |
|  |  |  |  |  |  |  |  |  |  |  |  | Tti1 |  |
|  |  |  |  |  |  |  |  |  |  |  |  | Entpd7 |  |
|  |  |  |  |  |  |  |  |  |  |  |  | Pfdn4 |  |
|  |  |  |  |  |  |  |  |  |  |  |  | Gins2 |  |
|  |  |  |  |  |  |  |  |  |  |  |  | Timm10 |  |
|  |  |  |  |  |  |  |  |  |  |  |  | Noc3l |  |
|  |  |  |  |  |  |  |  |  |  |  |  | Scly |  |
|  |  |  |  |  |  |  |  |  |  |  |  | Psmg2 |  |
|  |  |  |  |  |  |  |  |  |  |  |  | Dtl |  |
|  |  |  |  |  |  |  |  |  |  |  |  | Dnaaf5 |  |
|  |  |  |  |  |  |  |  |  |  |  |  | Cped1 |  |
|  |  |  |  |  |  |  |  |  |  |  |  | 5730455P16Rik |  |
|  |  |  |  |  |  |  |  |  |  |  |  | Lman2l |  |
|  |  |  |  |  |  |  |  |  |  |  |  | Senp7 |  |
|  |  |  |  |  |  |  |  |  |  |  |  | Pnp2 |  |
|  |  |  |  |  |  |  |  |  |  |  |  | Mphosph8 |  |
|  |  |  |  |  |  |  |  |  |  |  |  | Ccdc9b |  |
|  |  |  |  |  |  |  |  |  |  |  |  | Rbm12 |  |
|  |  |  |  |  |  |  |  |  |  |  |  | Tecpr2 |  |
|  |  |  |  |  |  |  |  |  |  |  |  | Fignl1 |  |
|  |  |  |  |  |  |  |  |  |  |  |  | Wdr73 |  |
|  |  |  |  |  |  |  |  |  |  |  |  | Chek2 |  |
|  |  |  |  |  |  |  |  |  |  |  |  | Shcbp1 |  |
|  |  |  |  |  |  |  |  |  |  |  |  | Gm8991 |  |
|  |  |  |  |  |  |  |  |  |  |  |  | Nrep |  |
|  |  |  |  |  |  |  |  |  |  |  |  | Cdca2 |  |
|  |  |  |  |  |  |  |  |  |  |  |  | Gtf2h4 |  |
|  |  |  |  |  |  |  |  |  |  |  |  | Fbxl4 |  |
|  |  |  |  |  |  |  |  |  |  |  |  | Naf1 |  |
|  |  |  |  |  |  |  |  |  |  |  |  | Sfmbt1 |  |
|  |  |  |  |  |  |  |  |  |  |  |  | Trim32 |  |
|  |  |  |  |  |  |  |  |  |  |  |  | Amigo1 |  |
|  |  |  |  |  |  |  |  |  |  |  |  | Prmt3 |  |
|  |  |  |  |  |  |  |  |  |  |  |  | Crtc1 |  |
|  |  |  |  |  |  |  |  |  |  |  |  | Chaf1b |  |
|  |  |  |  |  |  |  |  |  |  |  |  | Klhl25 |  |
|  |  |  |  |  |  |  |  |  |  |  |  | Alkbh8 |  |
|  |  |  |  |  |  |  |  |  |  |  |  | Zfp809 |  |
|  |  |  |  |  |  |  |  |  |  |  |  | Rrp15 |  |
|  |  |  |  |  |  |  |  |  |  |  |  | Lancl2 |  |
|  |  |  |  |  |  |  |  |  |  |  |  | Recql |  |
|  |  |  |  |  |  |  |  |  |  |  |  | Lrrc14 |  |
|  |  |  |  |  |  |  |  |  |  |  |  | Zfp26 |  |
|  |  |  |  |  |  |  |  |  |  |  |  | Rps2-ps10 |  |
|  |  |  |  |  |  |  |  |  |  |  |  | Dynlt1f |  |
|  |  |  |  |  |  |  |  |  |  |  |  | Zfp729a |  |
|  |  |  |  |  |  |  |  |  |  |  |  | Ttc27 |  |
|  |  |  |  |  |  |  |  |  |  |  |  | Tnfaip8l1 |  |
|  |  |  |  |  |  |  |  |  |  |  |  | Rfc4 |  |
|  |  |  |  |  |  |  |  |  |  |  |  | Gm5424 |  |
|  |  |  |  |  |  |  |  |  |  |  |  | Lsm5 |  |
|  |  |  |  |  |  |  |  |  |  |  |  | Tbc1d16 |  |
|  |  |  |  |  |  |  |  |  |  |  |  | Mybl2 |  |
|  |  |  |  |  |  |  |  |  |  |  |  | Pdcd7 |  |
|  |  |  |  |  |  |  |  |  |  |  |  | Rbm19 |  |
|  |  |  |  |  |  |  |  |  |  |  |  | Lactb |  |
|  |  |  |  |  |  |  |  |  |  |  |  | Kti12 |  |
|  |  |  |  |  |  |  |  |  |  |  |  | Srbd1 |  |
|  |  |  |  |  |  |  |  |  |  |  |  | Smad6 |  |
|  |  |  |  |  |  |  |  |  |  |  |  | Slc38a7 |  |
|  |  |  |  |  |  |  |  |  |  |  |  | Gabbr1 |  |
|  |  |  |  |  |  |  |  |  |  |  |  | Ddx51 |  |
|  |  |  |  |  |  |  |  |  |  |  |  | Zkscan8 |  |
|  |  |  |  |  |  |  |  |  |  |  |  | Klhl28 |  |
|  |  |  |  |  |  |  |  |  |  |  |  | Clspn |  |
|  |  |  |  |  |  |  |  |  |  |  |  | Mad1l1 |  |
|  |  |  |  |  |  |  |  |  |  |  |  | Nup37 |  |
|  |  |  |  |  |  |  |  |  |  |  |  | Kifc3 |  |
|  |  |  |  |  |  |  |  |  |  |  |  | Msto1 |  |
|  |  |  |  |  |  |  |  |  |  |  |  | Mrps18b |  |
|  |  |  |  |  |  |  |  |  |  |  |  | Papolg |  |
|  |  |  |  |  |  |  |  |  |  |  |  | Cyp2d22 |  |
|  |  |  |  |  |  |  |  |  |  |  |  | Dbp |  |
|  |  |  |  |  |  |  |  |  |  |  |  | Smpdl3b |  |
|  |  |  |  |  |  |  |  |  |  |  |  | Rabl3 |  |
|  |  |  |  |  |  |  |  |  |  |  |  | Map2k5 |  |
|  |  |  |  |  |  |  |  |  |  |  |  | Setd1a |  |
|  |  |  |  |  |  |  |  |  |  |  |  | Cxxc5 |  |
|  |  |  |  |  |  |  |  |  |  |  |  | Arl13b |  |
|  |  |  |  |  |  |  |  |  |  |  |  | Gstcd |  |
|  |  |  |  |  |  |  |  |  |  |  |  | Alg8 |  |
|  |  |  |  |  |  |  |  |  |  |  |  | AW209491 |  |
|  |  |  |  |  |  |  |  |  |  |  |  | Bcl11b |  |
|  |  |  |  |  |  |  |  |  |  |  |  | Kremen2 |  |
|  |  |  |  |  |  |  |  |  |  |  |  | Fkbp11 |  |
|  |  |  |  |  |  |  |  |  |  |  |  | Rrp8 |  |
|  |  |  |  |  |  |  |  |  |  |  |  | Srp54c |  |
|  |  |  |  |  |  |  |  |  |  |  |  | Gba2 |  |
|  |  |  |  |  |  |  |  |  |  |  |  | Prmt9 |  |
|  |  |  |  |  |  |  |  |  |  |  |  | Pfkfb2 |  |
|  |  |  |  |  |  |  |  |  |  |  |  | Alg12 |  |
|  |  |  |  |  |  |  |  |  |  |  |  | Arl14ep |  |
|  |  |  |  |  |  |  |  |  |  |  |  | Slx4 |  |
|  |  |  |  |  |  |  |  |  |  |  |  | Mak16 |  |
|  |  |  |  |  |  |  |  |  |  |  |  | Rbbp9 |  |
|  |  |  |  |  |  |  |  |  |  |  |  | Hira |  |
|  |  |  |  |  |  |  |  |  |  |  |  | Fam83d |  |
|  |  |  |  |  |  |  |  |  |  |  |  | Tmem79 |  |
|  |  |  |  |  |  |  |  |  |  |  |  | Cdc42ep3 |  |
|  |  |  |  |  |  |  |  |  |  |  |  | Mettl2 |  |
|  |  |  |  |  |  |  |  |  |  |  |  | Ak6 |  |
|  |  |  |  |  |  |  |  |  |  |  |  | Etaa1 |  |
|  |  |  |  |  |  |  |  |  |  |  |  | Thoc6 |  |
|  |  |  |  |  |  |  |  |  |  |  |  | Coa7 |  |
|  |  |  |  |  |  |  |  |  |  |  |  | Akip1 |  |
|  |  |  |  |  |  |  |  |  |  |  |  | Ndc80 |  |
|  |  |  |  |  |  |  |  |  |  |  |  | Ttf2 |  |
|  |  |  |  |  |  |  |  |  |  |  |  | Jrkl |  |
|  |  |  |  |  |  |  |  |  |  |  |  | Nars2 |  |
|  |  |  |  |  |  |  |  |  |  |  |  | Aifm2 |  |
|  |  |  |  |  |  |  |  |  |  |  |  | Saal1 |  |
|  |  |  |  |  |  |  |  |  |  |  |  | Sgo1 |  |
|  |  |  |  |  |  |  |  |  |  |  |  | Zfp821 |  |
|  |  |  |  |  |  |  |  |  |  |  |  | Haus6 |  |
|  |  |  |  |  |  |  |  |  |  |  |  | Gtse1 |  |
|  |  |  |  |  |  |  |  |  |  |  |  | Fam206a |  |
|  |  |  |  |  |  |  |  |  |  |  |  | Tmem253 |  |
|  |  |  |  |  |  |  |  |  |  |  |  | Siah2 |  |
|  |  |  |  |  |  |  |  |  |  |  |  | Zbtb40 |  |
|  |  |  |  |  |  |  |  |  |  |  |  | Fam110a |  |
|  |  |  |  |  |  |  |  |  |  |  |  | Mto1 |  |
|  |  |  |  |  |  |  |  |  |  |  |  | Haus7 |  |
|  |  |  |  |  |  |  |  |  |  |  |  | Endog |  |
|  |  |  |  |  |  |  |  |  |  |  |  | Mtg1 |  |
|  |  |  |  |  |  |  |  |  |  |  |  | Dhodh |  |
|  |  |  |  |  |  |  |  |  |  |  |  | Taco1 |  |
|  |  |  |  |  |  |  |  |  |  |  |  | Fbxo5 |  |
|  |  |  |  |  |  |  |  |  |  |  |  | Cdkn3 |  |
|  |  |  |  |  |  |  |  |  |  |  |  | Dhx37 |  |
|  |  |  |  |  |  |  |  |  |  |  |  | Aimp2 |  |
|  |  |  |  |  |  |  |  |  |  |  |  | Chac2 |  |
|  |  |  |  |  |  |  |  |  |  |  |  | Plekhg1 |  |
|  |  |  |  |  |  |  |  |  |  |  |  | Cda |  |
|  |  |  |  |  |  |  |  |  |  |  |  | Atp1a2 |  |
|  |  |  |  |  |  |  |  |  |  |  |  | Zkscan6 |  |
|  |  |  |  |  |  |  |  |  |  |  |  | Nufip1 |  |
|  |  |  |  |  |  |  |  |  |  |  |  | Tmem199 |  |
|  |  |  |  |  |  |  |  |  |  |  |  | Sgsh |  |
|  |  |  |  |  |  |  |  |  |  |  |  | Zfp53 |  |
|  |  |  |  |  |  |  |  |  |  |  |  | Wdr70 |  |
|  |  |  |  |  |  |  |  |  |  |  |  | Mpzl2 |  |
|  |  |  |  |  |  |  |  |  |  |  |  | Esco2 |  |
|  |  |  |  |  |  |  |  |  |  |  |  | Trip13 |  |
|  |  |  |  |  |  |  |  |  |  |  |  | Map3k9 |  |
|  |  |  |  |  |  |  |  |  |  |  |  | Snx30 |  |
|  |  |  |  |  |  |  |  |  |  |  |  | Spry1 |  |
|  |  |  |  |  |  |  |  |  |  |  |  | Sapcd2 |  |
|  |  |  |  |  |  |  |  |  |  |  |  | Fastkd3 |  |
|  |  |  |  |  |  |  |  |  |  |  |  | Ercc6l |  |
|  |  |  |  |  |  |  |  |  |  |  |  | Mb21d2 |  |
|  |  |  |  |  |  |  |  |  |  |  |  | Lrrc57 |  |
|  |  |  |  |  |  |  |  |  |  |  |  | Dll4 |  |
|  |  |  |  |  |  |  |  |  |  |  |  | Lrp8 |  |
|  |  |  |  |  |  |  |  |  |  |  |  | Polr3h |  |
|  |  |  |  |  |  |  |  |  |  |  |  | Tubgcp5 |  |
|  |  |  |  |  |  |  |  |  |  |  |  | Fhdc1 |  |
|  |  |  |  |  |  |  |  |  |  |  |  | Zbtb45 |  |
|  |  |  |  |  |  |  |  |  |  |  |  | Mphosph9 |  |
|  |  |  |  |  |  |  |  |  |  |  |  | Mettl3 |  |
|  |  |  |  |  |  |  |  |  |  |  |  | Slc19a2 |  |
|  |  |  |  |  |  |  |  |  |  |  |  | Ndufaf5 |  |
|  |  |  |  |  |  |  |  |  |  |  |  | Rpap3 |  |
|  |  |  |  |  |  |  |  |  |  |  |  | Trim65 |  |
|  |  |  |  |  |  |  |  |  |  |  |  | Lcmt2 |  |
|  |  |  |  |  |  |  |  |  |  |  |  | Mettl13 |  |
|  |  |  |  |  |  |  |  |  |  |  |  | D6Wsu163e |  |
|  |  |  |  |  |  |  |  |  |  |  |  | Thg1l |  |
|  |  |  |  |  |  |  |  |  |  |  |  | Ttk |  |
|  |  |  |  |  |  |  |  |  |  |  |  | Eif2b3 |  |
|  |  |  |  |  |  |  |  |  |  |  |  | Col4a5 |  |
|  |  |  |  |  |  |  |  |  |  |  |  | Gprasp1 |  |
|  |  |  |  |  |  |  |  |  |  |  |  | Mms22l |  |
|  |  |  |  |  |  |  |  |  |  |  |  | Tfpt |  |
|  |  |  |  |  |  |  |  |  |  |  |  | Ppm1l |  |
|  |  |  |  |  |  |  |  |  |  |  |  | Hgh1 |  |
|  |  |  |  |  |  |  |  |  |  |  |  | Poc5 |  |
|  |  |  |  |  |  |  |  |  |  |  |  | Slc24a3 |  |
|  |  |  |  |  |  |  |  |  |  |  |  | Gm48583 |  |
|  |  |  |  |  |  |  |  |  |  |  |  | Gtf2e1 |  |
|  |  |  |  |  |  |  |  |  |  |  |  | Rbm43 |  |
|  |  |  |  |  |  |  |  |  |  |  |  | Scaper |  |
|  |  |  |  |  |  |  |  |  |  |  |  | Gatm |  |
|  |  |  |  |  |  |  |  |  |  |  |  | Riox1 |  |
|  |  |  |  |  |  |  |  |  |  |  |  | Gm11249 |  |
|  |  |  |  |  |  |  |  |  |  |  |  | Jmjd4 |  |
|  |  |  |  |  |  |  |  |  |  |  |  | Prr11 |  |
|  |  |  |  |  |  |  |  |  |  |  |  | Cenpl |  |
|  |  |  |  |  |  |  |  |  |  |  |  | Ift22 |  |
|  |  |  |  |  |  |  |  |  |  |  |  | Hus1 |  |
|  |  |  |  |  |  |  |  |  |  |  |  | Wrap73 |  |
|  |  |  |  |  |  |  |  |  |  |  |  | Mtfp1 |  |
|  |  |  |  |  |  |  |  |  |  |  |  | Mtrr |  |
|  |  |  |  |  |  |  |  |  |  |  |  | Unc5cl |  |
|  |  |  |  |  |  |  |  |  |  |  |  | Aoc3 |  |
|  |  |  |  |  |  |  |  |  |  |  |  | Phc1 |  |
|  |  |  |  |  |  |  |  |  |  |  |  | Zwilch |  |
|  |  |  |  |  |  |  |  |  |  |  |  | Tmem201 |  |
|  |  |  |  |  |  |  |  |  |  |  |  | Tgds |  |
|  |  |  |  |  |  |  |  |  |  |  |  | Cdca7l |  |
|  |  |  |  |  |  |  |  |  |  |  |  | Raet1e |  |
|  |  |  |  |  |  |  |  |  |  |  |  | Ptrh1 |  |
|  |  |  |  |  |  |  |  |  |  |  |  | Gjb2 |  |
|  |  |  |  |  |  |  |  |  |  |  |  | Depdc1a |  |
|  |  |  |  |  |  |  |  |  |  |  |  | Stil |  |
|  |  |  |  |  |  |  |  |  |  |  |  | Mastl |  |
|  |  |  |  |  |  |  |  |  |  |  |  | Bcas3 |  |
|  |  |  |  |  |  |  |  |  |  |  |  | Pomk |  |
|  |  |  |  |  |  |  |  |  |  |  |  | Zc3h3 |  |
|  |  |  |  |  |  |  |  |  |  |  |  | Zfp984 |  |
|  |  |  |  |  |  |  |  |  |  |  |  | Eefsec |  |
|  |  |  |  |  |  |  |  |  |  |  |  | Tmem47 |  |
|  |  |  |  |  |  |  |  |  |  |  |  | Dtd1 |  |
|  |  |  |  |  |  |  |  |  |  |  |  | Tnfrsf8 |  |
|  |  |  |  |  |  |  |  |  |  |  |  | Mgme1 |  |
|  |  |  |  |  |  |  |  |  |  |  |  | 2610008E11Rik |  |
|  |  |  |  |  |  |  |  |  |  |  |  | Tmed1 |  |
|  |  |  |  |  |  |  |  |  |  |  |  | Srd5a1 |  |
|  |  |  |  |  |  |  |  |  |  |  |  | Prim2 |  |
|  |  |  |  |  |  |  |  |  |  |  |  | Edn1 |  |
|  |  |  |  |  |  |  |  |  |  |  |  | Cwc27 |  |
|  |  |  |  |  |  |  |  |  |  |  |  | Fanca |  |
|  |  |  |  |  |  |  |  |  |  |  |  | Gm9816 |  |
|  |  |  |  |  |  |  |  |  |  |  |  | Gm13736 |  |
|  |  |  |  |  |  |  |  |  |  |  |  | Mocs3 |  |
|  |  |  |  |  |  |  |  |  |  |  |  | Ppwd1 |  |
|  |  |  |  |  |  |  |  |  |  |  |  | Cps1 |  |
|  |  |  |  |  |  |  |  |  |  |  |  | Telo2 |  |
|  |  |  |  |  |  |  |  |  |  |  |  | Aph1b |  |
|  |  |  |  |  |  |  |  |  |  |  |  | Nme6 |  |
|  |  |  |  |  |  |  |  |  |  |  |  | Wdr76 |  |
|  |  |  |  |  |  |  |  |  |  |  |  | Fra10ac1 |  |
|  |  |  |  |  |  |  |  |  |  |  |  | Brca1 |  |
|  |  |  |  |  |  |  |  |  |  |  |  | Agt |  |
|  |  |  |  |  |  |  |  |  |  |  |  | Kif14 |  |
|  |  |  |  |  |  |  |  |  |  |  |  | Ears2 |  |
|  |  |  |  |  |  |  |  |  |  |  |  | Mbnl3 |  |
|  |  |  |  |  |  |  |  |  |  |  |  | Zfp758 |  |
|  |  |  |  |  |  |  |  |  |  |  |  | Mis18bp1 |  |
|  |  |  |  |  |  |  |  |  |  |  |  | Polh |  |
|  |  |  |  |  |  |  |  |  |  |  |  | Mcm9 |  |
|  |  |  |  |  |  |  |  |  |  |  |  | Ercc2 |  |
|  |  |  |  |  |  |  |  |  |  |  |  | Toe1 |  |
|  |  |  |  |  |  |  |  |  |  |  |  | Fam185a |  |
|  |  |  |  |  |  |  |  |  |  |  |  | Dnajc25 |  |
|  |  |  |  |  |  |  |  |  |  |  |  | Rad54l |  |
|  |  |  |  |  |  |  |  |  |  |  |  | Cdc45 |  |
|  |  |  |  |  |  |  |  |  |  |  |  | Tek |  |
|  |  |  |  |  |  |  |  |  |  |  |  | Zc3h11a |  |
|  |  |  |  |  |  |  |  |  |  |  |  | Dph6 |  |
|  |  |  |  |  |  |  |  |  |  |  |  | Erfe |  |
|  |  |  |  |  |  |  |  |  |  |  |  | Cdca5 |  |
|  |  |  |  |  |  |  |  |  |  |  |  | Focad |  |
|  |  |  |  |  |  |  |  |  |  |  |  | Ppy |  |
|  |  |  |  |  |  |  |  |  |  |  |  | Bcorl1 |  |
|  |  |  |  |  |  |  |  |  |  |  |  | Spdl1 |  |
|  |  |  |  |  |  |  |  |  |  |  |  | Polr3g |  |
|  |  |  |  |  |  |  |  |  |  |  |  | Faap24 |  |
|  |  |  |  |  |  |  |  |  |  |  |  | Nme7 |  |
|  |  |  |  |  |  |  |  |  |  |  |  | Rad9a |  |
|  |  |  |  |  |  |  |  |  |  |  |  | Haus3 |  |
|  |  |  |  |  |  |  |  |  |  |  |  | Kntc1 |  |
|  |  |  |  |  |  |  |  |  |  |  |  | Large1 |  |
|  |  |  |  |  |  |  |  |  |  |  |  | Zfp51 |  |
|  |  |  |  |  |  |  |  |  |  |  |  | Lin9 |  |
|  |  |  |  |  |  |  |  |  |  |  |  | Wrap53 |  |
|  |  |  |  |  |  |  |  |  |  |  |  | Nol10 |  |
|  |  |  |  |  |  |  |  |  |  |  |  | Hpgds |  |
|  |  |  |  |  |  |  |  |  |  |  |  | Cep76 |  |
|  |  |  |  |  |  |  |  |  |  |  |  | Pir |  |
|  |  |  |  |  |  |  |  |  |  |  |  | Il15 |  |
|  |  |  |  |  |  |  |  |  |  |  |  | Nmral1 |  |
|  |  |  |  |  |  |  |  |  |  |  |  | Sarnp |  |
|  |  |  |  |  |  |  |  |  |  |  |  | Bend3 |  |
|  |  |  |  |  |  |  |  |  |  |  |  | Ddit3 |  |
|  |  |  |  |  |  |  |  |  |  |  |  | 9530082P21Rik |  |
|  |  |  |  |  |  |  |  |  |  |  |  | Setd6 |  |
|  |  |  |  |  |  |  |  |  |  |  |  | Zdhhc15 |  |
|  |  |  |  |  |  |  |  |  |  |  |  | Rbks |  |
|  |  |  |  |  |  |  |  |  |  |  |  | Mfsd2a |  |
|  |  |  |  |  |  |  |  |  |  |  |  | Haus1 |  |
|  |  |  |  |  |  |  |  |  |  |  |  | Wdr45 |  |
|  |  |  |  |  |  |  |  |  |  |  |  | Zrsr1 |  |
|  |  |  |  |  |  |  |  |  |  |  |  | Rufy2 |  |
|  |  |  |  |  |  |  |  |  |  |  |  | Cdan1 |  |
|  |  |  |  |  |  |  |  |  |  |  |  | Banp |  |
|  |  |  |  |  |  |  |  |  |  |  |  | Copz2 |  |
|  |  |  |  |  |  |  |  |  |  |  |  | Pradc1 |  |
|  |  |  |  |  |  |  |  |  |  |  |  | Znhit6 |  |
|  |  |  |  |  |  |  |  |  |  |  |  | 4-Sep |  |
|  |  |  |  |  |  |  |  |  |  |  |  | Spata5 |  |
|  |  |  |  |  |  |  |  |  |  |  |  | Ccne1 |  |
|  |  |  |  |  |  |  |  |  |  |  |  | Gstp2 |  |
|  |  |  |  |  |  |  |  |  |  |  |  | Pole |  |
|  |  |  |  |  |  |  |  |  |  |  |  | Agk |  |
|  |  |  |  |  |  |  |  |  |  |  |  | Arsb |  |
|  |  |  |  |  |  |  |  |  |  |  |  | Cyb561d2 |  |
|  |  |  |  |  |  |  |  |  |  |  |  | Ccdc120 |  |
|  |  |  |  |  |  |  |  |  |  |  |  | 1110065P20Rik |  |
|  |  |  |  |  |  |  |  |  |  |  |  | Pgam5 |  |
|  |  |  |  |  |  |  |  |  |  |  |  | Sars2 |  |
|  |  |  |  |  |  |  |  |  |  |  |  | Rad18 |  |
|  |  |  |  |  |  |  |  |  |  |  |  | Diaph3 |  |
|  |  |  |  |  |  |  |  |  |  |  |  | Haspin |  |
|  |  |  |  |  |  |  |  |  |  |  |  | Igsf10 |  |
|  |  |  |  |  |  |  |  |  |  |  |  | Rpp30 |  |
|  |  |  |  |  |  |  |  |  |  |  |  | Psph |  |
|  |  |  |  |  |  |  |  |  |  |  |  | Taf3 |  |
|  |  |  |  |  |  |  |  |  |  |  |  | Wnt4 |  |
|  |  |  |  |  |  |  |  |  |  |  |  | Scfd2 |  |
|  |  |  |  |  |  |  |  |  |  |  |  | Idua |  |
|  |  |  |  |  |  |  |  |  |  |  |  | Dph2 |  |
|  |  |  |  |  |  |  |  |  |  |  |  | Fxyd4 |  |
|  |  |  |  |  |  |  |  |  |  |  |  | Caap1 |  |
|  |  |  |  |  |  |  |  |  |  |  |  | Fxn |  |
|  |  |  |  |  |  |  |  |  |  |  |  | Hmga1b |  |
|  |  |  |  |  |  |  |  |  |  |  |  | Cplx2 |  |
|  |  |  |  |  |  |  |  |  |  |  |  | Ppil3 |  |
|  |  |  |  |  |  |  |  |  |  |  |  | Gm4739 |  |
|  |  |  |  |  |  |  |  |  |  |  |  | Gm11585 |  |
|  |  |  |  |  |  |  |  |  |  |  |  | Cenpw |  |
|  |  |  |  |  |  |  |  |  |  |  |  | Plcd3 |  |
|  |  |  |  |  |  |  |  |  |  |  |  | Mcub |  |
|  |  |  |  |  |  |  |  |  |  |  |  | Polr1e |  |
|  |  |  |  |  |  |  |  |  |  |  |  | 1700066M21Rik |  |
|  |  |  |  |  |  |  |  |  |  |  |  | Nup43 |  |
|  |  |  |  |  |  |  |  |  |  |  |  | Prmt6 |  |
|  |  |  |  |  |  |  |  |  |  |  |  | Adgrl4 |  |
|  |  |  |  |  |  |  |  |  |  |  |  | Gm9531 |  |
|  |  |  |  |  |  |  |  |  |  |  |  | Znhit3 |  |
|  |  |  |  |  |  |  |  |  |  |  |  | Zc3hc1 |  |
|  |  |  |  |  |  |  |  |  |  |  |  | Yju2 |  |
|  |  |  |  |  |  |  |  |  |  |  |  | Rad51ap1 |  |
|  |  |  |  |  |  |  |  |  |  |  |  | Atrip |  |
|  |  |  |  |  |  |  |  |  |  |  |  | Ncam1 |  |
|  |  |  |  |  |  |  |  |  |  |  |  | Cep135 |  |
|  |  |  |  |  |  |  |  |  |  |  |  | Ddx11 |  |
|  |  |  |  |  |  |  |  |  |  |  |  | Zfp358 |  |
|  |  |  |  |  |  |  |  |  |  |  |  | Vcpkmt |  |
|  |  |  |  |  |  |  |  |  |  |  |  | Kif18b |  |
|  |  |  |  |  |  |  |  |  |  |  |  | Dph5 |  |
|  |  |  |  |  |  |  |  |  |  |  |  | Gm6768 |  |
|  |  |  |  |  |  |  |  |  |  |  |  | Osbpl1a |  |
|  |  |  |  |  |  |  |  |  |  |  |  | Fxyd1 |  |
|  |  |  |  |  |  |  |  |  |  |  |  | Slc2a4 |  |
|  |  |  |  |  |  |  |  |  |  |  |  | Atad5 |  |
|  |  |  |  |  |  |  |  |  |  |  |  | Skp2 |  |
|  |  |  |  |  |  |  |  |  |  |  |  | Traf3ip1 |  |
|  |  |  |  |  |  |  |  |  |  |  |  | Dffb |  |
|  |  |  |  |  |  |  |  |  |  |  |  | Nfatc2ip |  |
|  |  |  |  |  |  |  |  |  |  |  |  | Prpf40b |  |
|  |  |  |  |  |  |  |  |  |  |  |  | 3110082I17Rik |  |
|  |  |  |  |  |  |  |  |  |  |  |  | Mfsd7a |  |
|  |  |  |  |  |  |  |  |  |  |  |  | Il15ra |  |
|  |  |  |  |  |  |  |  |  |  |  |  | Gnb5 |  |
|  |  |  |  |  |  |  |  |  |  |  |  | Dna2 |  |
|  |  |  |  |  |  |  |  |  |  |  |  | Pinx1 |  |
|  |  |  |  |  |  |  |  |  |  |  |  | Armc6 |  |
|  |  |  |  |  |  |  |  |  |  |  |  | Tead2 |  |
|  |  |  |  |  |  |  |  |  |  |  |  | Gstp3 |  |
|  |  |  |  |  |  |  |  |  |  |  |  | Cbwd1 |  |
|  |  |  |  |  |  |  |  |  |  |  |  | Anapc10 |  |
|  |  |  |  |  |  |  |  |  |  |  |  | Cdc6 |  |
|  |  |  |  |  |  |  |  |  |  |  |  | Pif1 |  |
|  |  |  |  |  |  |  |  |  |  |  |  | Alg3 |  |
|  |  |  |  |  |  |  |  |  |  |  |  | Cenpq |  |
|  |  |  |  |  |  |  |  |  |  |  |  | 2310001H17Rik |  |
|  |  |  |  |  |  |  |  |  |  |  |  | Apip |  |
|  |  |  |  |  |  |  |  |  |  |  |  | Bhlha15 |  |
|  |  |  |  |  |  |  |  |  |  |  |  | Gm10232 |  |
|  |  |  |  |  |  |  |  |  |  |  |  | Kansl1l |  |
|  |  |  |  |  |  |  |  |  |  |  |  | Gm5637 |  |
|  |  |  |  |  |  |  |  |  |  |  |  | Hpdl |  |
|  |  |  |  |  |  |  |  |  |  |  |  | Utp23 |  |
|  |  |  |  |  |  |  |  |  |  |  |  | Cep78 |  |
|  |  |  |  |  |  |  |  |  |  |  |  | Lims2 |  |
|  |  |  |  |  |  |  |  |  |  |  |  | Gm14681 |  |
|  |  |  |  |  |  |  |  |  |  |  |  | Pamr1 |  |
|  |  |  |  |  |  |  |  |  |  |  |  | Cfap298 |  |
|  |  |  |  |  |  |  |  |  |  |  |  | Zdhhc14 |  |
|  |  |  |  |  |  |  |  |  |  |  |  | Mcph1 |  |
|  |  |  |  |  |  |  |  |  |  |  |  | Med29 |  |
|  |  |  |  |  |  |  |  |  |  |  |  | Traip |  |
|  |  |  |  |  |  |  |  |  |  |  |  | Hmgb1-ps2 |  |
|  |  |  |  |  |  |  |  |  |  |  |  | Top1mt |  |
|  |  |  |  |  |  |  |  |  |  |  |  | Cmtr2 |  |
|  |  |  |  |  |  |  |  |  |  |  |  | Gm13680 |  |
|  |  |  |  |  |  |  |  |  |  |  |  | Mad2l2 |  |
|  |  |  |  |  |  |  |  |  |  |  |  | Dnajc24 |  |
|  |  |  |  |  |  |  |  |  |  |  |  | Macc1 |  |
|  |  |  |  |  |  |  |  |  |  |  |  | Ehbp1 |  |
|  |  |  |  |  |  |  |  |  |  |  |  | Nsun5 |  |
|  |  |  |  |  |  |  |  |  |  |  |  | Zfp324 |  |
|  |  |  |  |  |  |  |  |  |  |  |  | Cwf19l1 |  |
|  |  |  |  |  |  |  |  |  |  |  |  | Trmt5 |  |
|  |  |  |  |  |  |  |  |  |  |  |  | 9430015G10Rik |  |
|  |  |  |  |  |  |  |  |  |  |  |  | Rnf39 |  |
|  |  |  |  |  |  |  |  |  |  |  |  | Stard8 |  |
|  |  |  |  |  |  |  |  |  |  |  |  | Gemin6 |  |
|  |  |  |  |  |  |  |  |  |  |  |  | Thap1 |  |
|  |  |  |  |  |  |  |  |  |  |  |  | Ldhb |  |
|  |  |  |  |  |  |  |  |  |  |  |  | Ska2 |  |
|  |  |  |  |  |  |  |  |  |  |  |  | Tpgs2 |  |
|  |  |  |  |  |  |  |  |  |  |  |  | Nexn |  |
|  |  |  |  |  |  |  |  |  |  |  |  | Med27 |  |
|  |  |  |  |  |  |  |  |  |  |  |  | Coil |  |
|  |  |  |  |  |  |  |  |  |  |  |  | Gm7336 |  |
|  |  |  |  |  |  |  |  |  |  |  |  | Pced1a |  |
|  |  |  |  |  |  |  |  |  |  |  |  | Cenpt |  |
|  |  |  |  |  |  |  |  |  |  |  |  | Plscr4 |  |
|  |  |  |  |  |  |  |  |  |  |  |  | Sssca1 |  |
|  |  |  |  |  |  |  |  |  |  |  |  | Orc1 |  |
|  |  |  |  |  |  |  |  |  |  |  |  | Sdr39u1 |  |
|  |  |  |  |  |  |  |  |  |  |  |  | Cox18 |  |
|  |  |  |  |  |  |  |  |  |  |  |  | Gemin2 |  |
|  |  |  |  |  |  |  |  |  |  |  |  | Snrnp35 |  |
|  |  |  |  |  |  |  |  |  |  |  |  | B3galt6 |  |
|  |  |  |  |  |  |  |  |  |  |  |  | Pop1 |  |
|  |  |  |  |  |  |  |  |  |  |  |  | Tdp1 |  |
|  |  |  |  |  |  |  |  |  |  |  |  | Fan1 |  |
|  |  |  |  |  |  |  |  |  |  |  |  | Gm11560 |  |
|  |  |  |  |  |  |  |  |  |  |  |  | Cep83os |  |
|  |  |  |  |  |  |  |  |  |  |  |  | C1qtnf3 |  |
|  |  |  |  |  |  |  |  |  |  |  |  | Zfp81 |  |
|  |  |  |  |  |  |  |  |  |  |  |  | Txnrd3 |  |
|  |  |  |  |  |  |  |  |  |  |  |  | Gm10146 |  |
|  |  |  |  |  |  |  |  |  |  |  |  | Zfp949 |  |
|  |  |  |  |  |  |  |  |  |  |  |  | Cmss1 |  |
|  |  |  |  |  |  |  |  |  |  |  |  | Kbtbd8 |  |
|  |  |  |  |  |  |  |  |  |  |  |  | Igfbp2 |  |
|  |  |  |  |  |  |  |  |  |  |  |  | Mrm3 |  |
|  |  |  |  |  |  |  |  |  |  |  |  | Bambi |  |
|  |  |  |  |  |  |  |  |  |  |  |  | Ska3 |  |
|  |  |  |  |  |  |  |  |  |  |  |  | Ralgps1 |  |
|  |  |  |  |  |  |  |  |  |  |  |  | Mrm2 |  |
|  |  |  |  |  |  |  |  |  |  |  |  | Srfbp1 |  |
|  |  |  |  |  |  |  |  |  |  |  |  | Pkd2l2 |  |
|  |  |  |  |  |  |  |  |  |  |  |  | Nsl1 |  |
|  |  |  |  |  |  |  |  |  |  |  |  | Blm |  |
|  |  |  |  |  |  |  |  |  |  |  |  | Sumf2 |  |
|  |  |  |  |  |  |  |  |  |  |  |  | Ighv5-9 |  |
|  |  |  |  |  |  |  |  |  |  |  |  | Gm9118 |  |
|  |  |  |  |  |  |  |  |  |  |  |  | Cenpo |  |
|  |  |  |  |  |  |  |  |  |  |  |  | Rev1 |  |
|  |  |  |  |  |  |  |  |  |  |  |  | Nkrf |  |
|  |  |  |  |  |  |  |  |  |  |  |  | Dsn1 |  |
|  |  |  |  |  |  |  |  |  |  |  |  | Tmem29 |  |
|  |  |  |  |  |  |  |  |  |  |  |  | Cenpm |  |
|  |  |  |  |  |  |  |  |  |  |  |  | Tada1 |  |
|  |  |  |  |  |  |  |  |  |  |  |  | Abca8a |  |
|  |  |  |  |  |  |  |  |  |  |  |  | Fam92a |  |
|  |  |  |  |  |  |  |  |  |  |  |  | Fam122b |  |
|  |  |  |  |  |  |  |  |  |  |  |  | Cdc25c |  |
|  |  |  |  |  |  |  |  |  |  |  |  | Srp54b |  |
|  |  |  |  |  |  |  |  |  |  |  |  | Gen1 |  |
|  |  |  |  |  |  |  |  |  |  |  |  | Zfp429 |  |
|  |  |  |  |  |  |  |  |  |  |  |  | Ppp1r13l |  |
|  |  |  |  |  |  |  |  |  |  |  |  | Adgrf1 |  |
|  |  |  |  |  |  |  |  |  |  |  |  | Mtbp |  |
|  |  |  |  |  |  |  |  |  |  |  |  | Tmem204 |  |
|  |  |  |  |  |  |  |  |  |  |  |  | Lyrm4 |  |
|  |  |  |  |  |  |  |  |  |  |  |  | Snupn |  |
|  |  |  |  |  |  |  |  |  |  |  |  | Pecr |  |
|  |  |  |  |  |  |  |  |  |  |  |  | Cenpi |  |
|  |  |  |  |  |  |  |  |  |  |  |  | Thbs3 |  |
|  |  |  |  |  |  |  |  |  |  |  |  | Hist1h4i |  |
|  |  |  |  |  |  |  |  |  |  |  |  | Exog |  |
|  |  |  |  |  |  |  |  |  |  |  |  | Psmg3 |  |
|  |  |  |  |  |  |  |  |  |  |  |  | Trim5 |  |
|  |  |  |  |  |  |  |  |  |  |  |  | Hyls1 |  |
|  |  |  |  |  |  |  |  |  |  |  |  | Slc23a4 |  |
|  |  |  |  |  |  |  |  |  |  |  |  | Shq1 |  |
|  |  |  |  |  |  |  |  |  |  |  |  | Hspd1-ps3 |  |
|  |  |  |  |  |  |  |  |  |  |  |  | Tspan6 |  |
|  |  |  |  |  |  |  |  |  |  |  |  | Tpi-rs11 |  |
|  |  |  |  |  |  |  |  |  |  |  |  | Cenpj |  |
|  |  |  |  |  |  |  |  |  |  |  |  | Bclaf3 |  |
|  |  |  |  |  |  |  |  |  |  |  |  | BC005561 |  |
|  |  |  |  |  |  |  |  |  |  |  |  | Ercc8 |  |
|  |  |  |  |  |  |  |  |  |  |  |  | Ctsf |  |
|  |  |  |  |  |  |  |  |  |  |  |  | Alg13 |  |
|  |  |  |  |  |  |  |  |  |  |  |  | Gins1 |  |
|  |  |  |  |  |  |  |  |  |  |  |  | Rac3 |  |
|  |  |  |  |  |  |  |  |  |  |  |  | Fendrr |  |
|  |  |  |  |  |  |  |  |  |  |  |  | Epb41l4a |  |
|  |  |  |  |  |  |  |  |  |  |  |  | Fer1l4 |  |
|  |  |  |  |  |  |  |  |  |  |  |  | Exo1 |  |
|  |  |  |  |  |  |  |  |  |  |  |  | Trmt44 |  |
|  |  |  |  |  |  |  |  |  |  |  |  | Gm8186 |  |
|  |  |  |  |  |  |  |  |  |  |  |  | Dctd |  |
|  |  |  |  |  |  |  |  |  |  |  |  | Parpbp |  |
|  |  |  |  |  |  |  |  |  |  |  |  | Gm13597 |  |
|  |  |  |  |  |  |  |  |  |  |  |  | Ldhd |  |
|  |  |  |  |  |  |  |  |  |  |  |  | Arsk |  |
|  |  |  |  |  |  |  |  |  |  |  |  | Chek1 |  |
|  |  |  |  |  |  |  |  |  |  |  |  | Gm7730 |  |
|  |  |  |  |  |  |  |  |  |  |  |  | Gm37305 |  |
|  |  |  |  |  |  |  |  |  |  |  |  | Ddx31 |  |
|  |  |  |  |  |  |  |  |  |  |  |  | Art3 |  |
|  |  |  |  |  |  |  |  |  |  |  |  | Wdr34 |  |
|  |  |  |  |  |  |  |  |  |  |  |  | Fbxl15 |  |
|  |  |  |  |  |  |  |  |  |  |  |  | Adal |  |
|  |  |  |  |  |  |  |  |  |  |  |  | Slc25a26 |  |
|  |  |  |  |  |  |  |  |  |  |  |  | C2cd4a |  |
|  |  |  |  |  |  |  |  |  |  |  |  | Bco2 |  |
|  |  |  |  |  |  |  |  |  |  |  |  | Kcnh2 |  |
|  |  |  |  |  |  |  |  |  |  |  |  | Nudc-ps1 |  |
|  |  |  |  |  |  |  |  |  |  |  |  | 4933421O10Rik |  |
|  |  |  |  |  |  |  |  |  |  |  |  | Dnph1 |  |
|  |  |  |  |  |  |  |  |  |  |  |  | Zfp316 |  |
|  |  |  |  |  |  |  |  |  |  |  |  | Gm8540 |  |
|  |  |  |  |  |  |  |  |  |  |  |  | Steap1 |  |
|  |  |  |  |  |  |  |  |  |  |  |  | Brip1 |  |
|  |  |  |  |  |  |  |  |  |  |  |  | Elp6 |  |
|  |  |  |  |  |  |  |  |  |  |  |  | Ago4 |  |
|  |  |  |  |  |  |  |  |  |  |  |  | Arvcf |  |
|  |  |  |  |  |  |  |  |  |  |  |  | 2810006K23Rik |  |
|  |  |  |  |  |  |  |  |  |  |  |  | Nubpl |  |
|  |  |  |  |  |  |  |  |  |  |  |  | Chtf18 |  |
|  |  |  |  |  |  |  |  |  |  |  |  | Trmt10a |  |
|  |  |  |  |  |  |  |  |  |  |  |  | Gm6560 |  |
|  |  |  |  |  |  |  |  |  |  |  |  | Vrk2 |  |
|  |  |  |  |  |  |  |  |  |  |  |  | Gm11451 |  |
|  |  |  |  |  |  |  |  |  |  |  |  | Dtwd1 |  |
|  |  |  |  |  |  |  |  |  |  |  |  | Gm9770 |  |
|  |  |  |  |  |  |  |  |  |  |  |  | Ccdc157 |  |
|  |  |  |  |  |  |  |  |  |  |  |  | Rpusd1 |  |
|  |  |  |  |  |  |  |  |  |  |  |  | Anapc15 |  |
|  |  |  |  |  |  |  |  |  |  |  |  | Tubd1 |  |
|  |  |  |  |  |  |  |  |  |  |  |  | Rhobtb3 |  |
|  |  |  |  |  |  |  |  |  |  |  |  | Tmem38a |  |
|  |  |  |  |  |  |  |  |  |  |  |  | Rsl1 |  |
|  |  |  |  |  |  |  |  |  |  |  |  | Naif1 |  |
|  |  |  |  |  |  |  |  |  |  |  |  | Insm1 |  |
|  |  |  |  |  |  |  |  |  |  |  |  | Zswim1 |  |
|  |  |  |  |  |  |  |  |  |  |  |  | Mamdc2 |  |
|  |  |  |  |  |  |  |  |  |  |  |  | Gm4202 |  |
|  |  |  |  |  |  |  |  |  |  |  |  | Atp23 |  |
|  |  |  |  |  |  |  |  |  |  |  |  | Siah1b |  |
|  |  |  |  |  |  |  |  |  |  |  |  | Mb21d1 |  |
|  |  |  |  |  |  |  |  |  |  |  |  | Syt8 |  |
|  |  |  |  |  |  |  |  |  |  |  |  | Gm9761 |  |
|  |  |  |  |  |  |  |  |  |  |  |  | Wdr62 |  |
|  |  |  |  |  |  |  |  |  |  |  |  | Gm17201 |  |
|  |  |  |  |  |  |  |  |  |  |  |  | Cenph |  |
|  |  |  |  |  |  |  |  |  |  |  |  | Glp2r |  |
|  |  |  |  |  |  |  |  |  |  |  |  | Gm7887 |  |
|  |  |  |  |  |  |  |  |  |  |  |  | 2310034G01Rik |  |
|  |  |  |  |  |  |  |  |  |  |  |  | Rab43 |  |
|  |  |  |  |  |  |  |  |  |  |  |  | Tmc7 |  |
|  |  |  |  |  |  |  |  |  |  |  |  | Zcchc10 |  |
|  |  |  |  |  |  |  |  |  |  |  |  | Ptrhd1 |  |
|  |  |  |  |  |  |  |  |  |  |  |  | Muc1 |  |
|  |  |  |  |  |  |  |  |  |  |  |  | Suv39h2 |  |
|  |  |  |  |  |  |  |  |  |  |  |  | Ulbp1 |  |
|  |  |  |  |  |  |  |  |  |  |  |  | Mageh1 |  |
|  |  |  |  |  |  |  |  |  |  |  |  | Mcm8 |  |
|  |  |  |  |  |  |  |  |  |  |  |  | Dusp10 |  |
|  |  |  |  |  |  |  |  |  |  |  |  | Rps12-ps4 |  |
|  |  |  |  |  |  |  |  |  |  |  |  | Gm6421 |  |
|  |  |  |  |  |  |  |  |  |  |  |  | Rbmx2 |  |
|  |  |  |  |  |  |  |  |  |  |  |  | Chchd6 |  |
|  |  |  |  |  |  |  |  |  |  |  |  | Pidd1 |  |
|  |  |  |  |  |  |  |  |  |  |  |  | Cenpn |  |
|  |  |  |  |  |  |  |  |  |  |  |  | Tbc1d7 |  |
|  |  |  |  |  |  |  |  |  |  |  |  | Khdc1a |  |
|  |  |  |  |  |  |  |  |  |  |  |  | Syce2 |  |
|  |  |  |  |  |  |  |  |  |  |  |  | Rftn2 |  |
|  |  |  |  |  |  |  |  |  |  |  |  | Sardh |  |
|  |  |  |  |  |  |  |  |  |  |  |  | Slc25a14 |  |
|  |  |  |  |  |  |  |  |  |  |  |  | Trim62 |  |
|  |  |  |  |  |  |  |  |  |  |  |  | Ppp1r3e |  |
|  |  |  |  |  |  |  |  |  |  |  |  | Tedc2 |  |
|  |  |  |  |  |  |  |  |  |  |  |  | Tmem216 |  |
|  |  |  |  |  |  |  |  |  |  |  |  | Trf |  |
|  |  |  |  |  |  |  |  |  |  |  |  | Tbc1d31 |  |
|  |  |  |  |  |  |  |  |  |  |  |  | Cenpk |  |
|  |  |  |  |  |  |  |  |  |  |  |  | Pole2 |  |
|  |  |  |  |  |  |  |  |  |  |  |  | Ephb6 |  |
|  |  |  |  |  |  |  |  |  |  |  |  | Dhrs13 |  |
|  |  |  |  |  |  |  |  |  |  |  |  | Tagap |  |
|  |  |  |  |  |  |  |  |  |  |  |  | Ube2t |  |
|  |  |  |  |  |  |  |  |  |  |  |  | Kifc5b |  |
|  |  |  |  |  |  |  |  |  |  |  |  | Gm12184 |  |
|  |  |  |  |  |  |  |  |  |  |  |  | Armcx1 |  |
|  |  |  |  |  |  |  |  |  |  |  |  | Ttc39aos1 |  |
|  |  |  |  |  |  |  |  |  |  |  |  | Gm14325 |  |
|  |  |  |  |  |  |  |  |  |  |  |  | Meis3 |  |
|  |  |  |  |  |  |  |  |  |  |  |  | Dimt1 |  |
|  |  |  |  |  |  |  |  |  |  |  |  | Emc9 |  |
|  |  |  |  |  |  |  |  |  |  |  |  | Eps8l1 |  |
|  |  |  |  |  |  |  |  |  |  |  |  | Mtrf1 |  |
|  |  |  |  |  |  |  |  |  |  |  |  | Rhbdl3 |  |
|  |  |  |  |  |  |  |  |  |  |  |  | Resp18 |  |
|  |  |  |  |  |  |  |  |  |  |  |  | Gon7 |  |
|  |  |  |  |  |  |  |  |  |  |  |  | Gm10156 |  |
|  |  |  |  |  |  |  |  |  |  |  |  | Mark1 |  |
|  |  |  |  |  |  |  |  |  |  |  |  | Srrd |  |
|  |  |  |  |  |  |  |  |  |  |  |  | Kdm8 |  |
|  |  |  |  |  |  |  |  |  |  |  |  | Dnajc12 |  |
|  |  |  |  |  |  |  |  |  |  |  |  | Ddias |  |
|  |  |  |  |  |  |  |  |  |  |  |  | Spata5l1 |  |
|  |  |  |  |  |  |  |  |  |  |  |  | Gm15387 |  |
|  |  |  |  |  |  |  |  |  |  |  |  | Tcea1-ps1 |  |
|  |  |  |  |  |  |  |  |  |  |  |  | Cenps |  |
|  |  |  |  |  |  |  |  |  |  |  |  | Ift81 |  |
|  |  |  |  |  |  |  |  |  |  |  |  | Gm7143 |  |
|  |  |  |  |  |  |  |  |  |  |  |  | Pax8 |  |
|  |  |  |  |  |  |  |  |  |  |  |  | Lbhd1 |  |
|  |  |  |  |  |  |  |  |  |  |  |  | BC055324 |  |
|  |  |  |  |  |  |  |  |  |  |  |  | Gramd2 |  |
|  |  |  |  |  |  |  |  |  |  |  |  | Actr6 |  |
|  |  |  |  |  |  |  |  |  |  |  |  | Ddx59 |  |
|  |  |  |  |  |  |  |  |  |  |  |  | Krt13 |  |
|  |  |  |  |  |  |  |  |  |  |  |  | Fchsd1 |  |
|  |  |  |  |  |  |  |  |  |  |  |  | Thtpa |  |
|  |  |  |  |  |  |  |  |  |  |  |  | Cenpp |  |
|  |  |  |  |  |  |  |  |  |  |  |  | Gm26532 |  |
|  |  |  |  |  |  |  |  |  |  |  |  | Gm3756 |  |
|  |  |  |  |  |  |  |  |  |  |  |  | Fastkd5 |  |
|  |  |  |  |  |  |  |  |  |  |  |  | Gm5786 |  |
|  |  |  |  |  |  |  |  |  |  |  |  | Kctd7 |  |
|  |  |  |  |  |  |  |  |  |  |  |  | Gm5417 |  |
|  |  |  |  |  |  |  |  |  |  |  |  | Zfp560 |  |
|  |  |  |  |  |  |  |  |  |  |  |  | Scarf1 |  |
|  |  |  |  |  |  |  |  |  |  |  |  | Lrrc56 |  |
|  |  |  |  |  |  |  |  |  |  |  |  | Kansl2-ps |  |
|  |  |  |  |  |  |  |  |  |  |  |  | Gm8394 |  |
|  |  |  |  |  |  |  |  |  |  |  |  | Bard1 |  |
|  |  |  |  |  |  |  |  |  |  |  |  | Oip5 |  |
|  |  |  |  |  |  |  |  |  |  |  |  | Unkl |  |
|  |  |  |  |  |  |  |  |  |  |  |  | Dus4l |  |
|  |  |  |  |  |  |  |  |  |  |  |  | Gm21811 |  |
|  |  |  |  |  |  |  |  |  |  |  |  | Rpp38 |  |
|  |  |  |  |  |  |  |  |  |  |  |  | Troap |  |
|  |  |  |  |  |  |  |  |  |  |  |  | Gm20075 |  |
|  |  |  |  |  |  |  |  |  |  |  |  | Ss18l1 |  |
|  |  |  |  |  |  |  |  |  |  |  |  | Ska1 |  |
|  |  |  |  |  |  |  |  |  |  |  |  | Gm10074 |  |
|  |  |  |  |  |  |  |  |  |  |  |  | Cenpu |  |
|  |  |  |  |  |  |  |  |  |  |  |  | Kctd12b |  |
|  |  |  |  |  |  |  |  |  |  |  |  | B3gnt2 |  |
|  |  |  |  |  |  |  |  |  |  |  |  | Trmo |  |
|  |  |  |  |  |  |  |  |  |  |  |  | Eme1 |  |
|  |  |  |  |  |  |  |  |  |  |  |  | Asic1 |  |
|  |  |  |  |  |  |  |  |  |  |  |  | Rabl2 |  |
|  |  |  |  |  |  |  |  |  |  |  |  | Lonrf3 |  |
|  |  |  |  |  |  |  |  |  |  |  |  | Tctn3 |  |
|  |  |  |  |  |  |  |  |  |  |  |  | Gm3776 |  |
|  |  |  |  |  |  |  |  |  |  |  |  | Zfp874b |  |
|  |  |  |  |  |  |  |  |  |  |  |  | Gm13577 |  |
|  |  |  |  |  |  |  |  |  |  |  |  | Phlda2 |  |
|  |  |  |  |  |  |  |  |  |  |  |  | Kctd1 |  |
|  |  |  |  |  |  |  |  |  |  |  |  | Qpct |  |
|  |  |  |  |  |  |  |  |  |  |  |  | Gm14326 |  |
|  |  |  |  |  |  |  |  |  |  |  |  | Xrcc2 |  |
|  |  |  |  |  |  |  |  |  |  |  |  | C1qtnf2 |  |
|  |  |  |  |  |  |  |  |  |  |  |  | Tagln |  |
|  |  |  |  |  |  |  |  |  |  |  |  | Mettl25 |  |
|  |  |  |  |  |  |  |  |  |  |  |  | Cma1 |  |
|  |  |  |  |  |  |  |  |  |  |  |  | Mblac2 |  |
|  |  |  |  |  |  |  |  |  |  |  |  | Recql4 |  |
|  |  |  |  |  |  |  |  |  |  |  |  | Gm7353 |  |
|  |  |  |  |  |  |  |  |  |  |  |  | Trmt61b |  |
|  |  |  |  |  |  |  |  |  |  |  |  | Gm11993 |  |
|  |  |  |  |  |  |  |  |  |  |  |  | Rbm3-ps |  |
|  |  |  |  |  |  |  |  |  |  |  |  | Lysmd1 |  |
|  |  |  |  |  |  |  |  |  |  |  |  | Fcrls |  |
|  |  |  |  |  |  |  |  |  |  |  |  | Gm10175 |  |
|  |  |  |  |  |  |  |  |  |  |  |  | Gins3 |  |
|  |  |  |  |  |  |  |  |  |  |  |  | Ankdd1b |  |
|  |  |  |  |  |  |  |  |  |  |  |  | Krt87 |  |
|  |  |  |  |  |  |  |  |  |  |  |  | Ctxn1 |  |
|  |  |  |  |  |  |  |  |  |  |  |  | Adat2 |  |
|  |  |  |  |  |  |  |  |  |  |  |  | Slitrk6 |  |
|  |  |  |  |  |  |  |  |  |  |  |  | Zfp213 |  |
|  |  |  |  |  |  |  |  |  |  |  |  | Gm379 |  |
|  |  |  |  |  |  |  |  |  |  |  |  | B230118H07Rik |  |
|  |  |  |  |  |  |  |  |  |  |  |  | Trnp1 |  |
|  |  |  |  |  |  |  |  |  |  |  |  | Brsk1 |  |
|  |  |  |  |  |  |  |  |  |  |  |  | Gm5559 |  |
|  |  |  |  |  |  |  |  |  |  |  |  | Tfpi2 |  |
|  |  |  |  |  |  |  |  |  |  |  |  | Gm7846 |  |
|  |  |  |  |  |  |  |  |  |  |  |  | Gm6311 |  |
|  |  |  |  |  |  |  |  |  |  |  |  | Aunip |  |
|  |  |  |  |  |  |  |  |  |  |  |  | Gm13493 |  |
|  |  |  |  |  |  |  |  |  |  |  |  | Rmi2 |  |
|  |  |  |  |  |  |  |  |  |  |  |  | Rpusd2 |  |
|  |  |  |  |  |  |  |  |  |  |  |  | Nol3 |  |
|  |  |  |  |  |  |  |  |  |  |  |  | 2610318N02Rik |  |
|  |  |  |  |  |  |  |  |  |  |  |  | Vps51 |  |
|  |  |  |  |  |  |  |  |  |  |  |  | Gm43980 |  |
|  |  |  |  |  |  |  |  |  |  |  |  | Bmyc |  |
|  |  |  |  |  |  |  |  |  |  |  |  | Dnaja4 |  |
|  |  |  |  |  |  |  |  |  |  |  |  | Mtfr2 |  |
|  |  |  |  |  |  |  |  |  |  |  |  | Krt84 |  |
|  |  |  |  |  |  |  |  |  |  |  |  | C2cd4b |  |
|  |  |  |  |  |  |  |  |  |  |  |  | Mettl18 |  |
|  |  |  |  |  |  |  |  |  |  |  |  | Pemt |  |
|  |  |  |  |  |  |  |  |  |  |  |  | Gm10557 |  |
|  |  |  |  |  |  |  |  |  |  |  |  | Eva1a |  |
|  |  |  |  |  |  |  |  |  |  |  |  | Rgl3 |  |
|  |  |  |  |  |  |  |  |  |  |  |  | Gm48226 |  |
|  |  |  |  |  |  |  |  |  |  |  |  | Ttc30a1 |  |
|  |  |  |  |  |  |  |  |  |  |  |  | Hmgn2-ps1 |  |
|  |  |  |  |  |  |  |  |  |  |  |  | Tmem198b |  |
|  |  |  |  |  |  |  |  |  |  |  |  | Med18 |  |
|  |  |  |  |  |  |  |  |  |  |  |  | Rtn2 |  |
|  |  |  |  |  |  |  |  |  |  |  |  | Chrnb1 |  |
|  |  |  |  |  |  |  |  |  |  |  |  | Tpbg |  |
|  |  |  |  |  |  |  |  |  |  |  |  | Gm34084 |  |
|  |  |  |  |  |  |  |  |  |  |  |  | C630043F03Rik |  |
|  |  |  |  |  |  |  |  |  |  |  |  | Gm7879 |  |
|  |  |  |  |  |  |  |  |  |  |  |  | Zfp428 |  |
|  |  |  |  |  |  |  |  |  |  |  |  | Nudt15 |  |
|  |  |  |  |  |  |  |  |  |  |  |  | Tubb4b-ps1 |  |
|  |  |  |  |  |  |  |  |  |  |  |  | Psmc3ip |  |
|  |  |  |  |  |  |  |  |  |  |  |  | Myct1 |  |
|  |  |  |  |  |  |  |  |  |  |  |  | Fancb |  |
|  |  |  |  |  |  |  |  |  |  |  |  | Rnf113a1 |  |
|  |  |  |  |  |  |  |  |  |  |  |  | Gm4735 |  |
|  |  |  |  |  |  |  |  |  |  |  |  | Gm8909 |  |
|  |  |  |  |  |  |  |  |  |  |  |  | Hes2 |  |
|  |  |  |  |  |  |  |  |  |  |  |  | BC030867 |  |
|  |  |  |  |  |  |  |  |  |  |  |  | Kcnk3 |  |
|  |  |  |  |  |  |  |  |  |  |  |  | Pygm |  |
|  |  |  |  |  |  |  |  |  |  |  |  | Gm5776 |  |
|  |  |  |  |  |  |  |  |  |  |  |  | Atg9b |  |
|  |  |  |  |  |  |  |  |  |  |  |  | Zfp677 |  |
|  |  |  |  |  |  |  |  |  |  |  |  | 4833418N02Rik |  |
|  |  |  |  |  |  |  |  |  |  |  |  | 9130204K15Rik |  |
|  |  |  |  |  |  |  |  |  |  |  |  | Ccsap |  |
|  |  |  |  |  |  |  |  |  |  |  |  | Smarce1-ps1 |  |
|  |  |  |  |  |  |  |  |  |  |  |  | Gm4950 |  |
|  |  |  |  |  |  |  |  |  |  |  |  | Cox6b2 |  |
|  |  |  |  |  |  |  |  |  |  |  |  | Rnf26 |  |
|  |  |  |  |  |  |  |  |  |  |  |  | Gm6477 |  |
|  |  |  |  |  |  |  |  |  |  |  |  | Ighv1-63 |  |
|  |  |  |  |  |  |  |  |  |  |  |  | H2-T-ps |  |
|  |  |  |  |  |  |  |  |  |  |  |  | Ighv2-9 |  |
|  |  |  |  |  |  |  |  |  |  |  |  | Gm42528 |  |
|  |  |  |  |  |  |  |  |  |  |  |  | Rps13-ps1 |  |
|  |  |  |  |  |  |  |  |  |  |  |  | Fgf11 |  |
|  |  |  |  |  |  |  |  |  |  |  |  | Gm13135 |  |
|  |  |  |  |  |  |  |  |  |  |  |  | Nat14 |  |
|  |  |  |  |  |  |  |  |  |  |  |  | Thpo |  |
|  |  |  |  |  |  |  |  |  |  |  |  | Mmp11 |  |
|  |  |  |  |  |  |  |  |  |  |  |  | Gm9833 |  |
|  |  |  |  |  |  |  |  |  |  |  |  | Gm49041 |  |
|  |  |  |  |  |  |  |  |  |  |  |  | Tymp |  |
|  |  |  |  |  |  |  |  |  |  |  |  | Gfra3 |  |
|  |  |  |  |  |  |  |  |  |  |  |  | Dand5 |  |
|  |  |  |  |  |  |  |  |  |  |  |  | Gm12216 |  |
|  |  |  |  |  |  |  |  |  |  |  |  | Gm11131 |  |
|  |  |  |  |  |  |  |  |  |  |  |  | Dtwd2 |  |
|  |  |  |  |  |  |  |  |  |  |  |  | Gm12346 |  |
|  |  |  |  |  |  |  |  |  |  |  |  | Fancf |  |
|  |  |  |  |  |  |  |  |  |  |  |  | Ighv1-34 |  |
|  |  |  |  |  |  |  |  |  |  |  |  | Plscr2 |  |
|  |  |  |  |  |  |  |  |  |  |  |  | Ccdc3 |  |
|  |  |  |  |  |  |  |  |  |  |  |  | Rec8 |  |
|  |  |  |  |  |  |  |  |  |  |  |  | Rps18-ps3 |  |
|  |  |  |  |  |  |  |  |  |  |  |  | Scnn1g |  |
|  |  |  |  |  |  |  |  |  |  |  |  | Gm7160 |  |
|  |  |  |  |  |  |  |  |  |  |  |  | Gm10451 |  |
|  |  |  |  |  |  |  |  |  |  |  |  | Gm10254 |  |
|  |  |  |  |  |  |  |  |  |  |  |  | Gm5425 |  |
|  |  |  |  |  |  |  |  |  |  |  |  | Gm10819 |  |
|  |  |  |  |  |  |  |  |  |  |  |  | AC114990.3 |  |
|  |  |  |  |  |  |  |  |  |  |  |  | Particl |  |
|  |  |  |  |  |  |  |  |  |  |  |  | Gm12070 |  |
|  |  |  |  |  |  |  |  |  |  |  |  | Vstm2l |  |
|  |  |  |  |  |  |  |  |  |  |  |  | 4930579G24Rik |  |
|  |  |  |  |  |  |  |  |  |  |  |  | Gm49338 |  |
|  |  |  |  |  |  |  |  |  |  |  |  | Rpl10-ps5 |  |
|  |  |  |  |  |  |  |  |  |  |  |  | Neurog3 |  |
|  |  |  |  |  |  |  |  |  |  |  |  | 1810008I18Rik |  |
|  |  |  |  |  |  |  |  |  |  |  |  | Slc27a3 |  |
|  |  |  |  |  |  |  |  |  |  |  |  | Clba1 |  |
|  |  |  |  |  |  |  |  |  |  |  |  | Gm6363 |  |
|  |  |  |  |  |  |  |  |  |  |  |  | Ube2cbp |  |
|  |  |  |  |  |  |  |  |  |  |  |  | Gm10524 |  |
|  |  |  |  |  |  |  |  |  |  |  |  | Gm14403 |  |
|  |  |  |  |  |  |  |  |  |  |  |  | Eif3j2 |  |
|  |  |  |  |  |  |  |  |  |  |  |  | Pnck |  |
|  |  |  |  |  |  |  |  |  |  |  |  | Gm6565 |  |
|  |  |  |  |  |  |  |  |  |  |  |  | Nnat |  |
|  |  |  |  |  |  |  |  |  |  |  |  | Lrr1 |  |
|  |  |  |  |  |  |  |  |  |  |  |  | Nr2c2ap |  |
|  |  |  |  |  |  |  |  |  |  |  |  | Hnmt |  |
|  |  |  |  |  |  |  |  |  |  |  |  | Nkx3-2 |  |
|  |  |  |  |  |  |  |  |  |  |  |  | Gm11775 |  |
|  |  |  |  |  |  |  |  |  |  |  |  | E230013L22Rik |  |
|  |  |  |  |  |  |  |  |  |  |  |  | Rps26-ps1 |  |
|  |  |  |  |  |  |  |  |  |  |  |  | Optc |  |
|  |  |  |  |  |  |  |  |  |  |  |  | Gm11223 |  |
|  |  |  |  |  |  |  |  |  |  |  |  | Gm15452 |  |
|  |  |  |  |  |  |  |  |  |  |  |  | Sstr2 |  |
|  |  |  |  |  |  |  |  |  |  |  |  | Barx2 |  |
|  |  |  |  |  |  |  |  |  |  |  |  | Zc4h2 |  |
|  |  |  |  |  |  |  |  |  |  |  |  | Chad |  |
|  |  |  |  |  |  |  |  |  |  |  |  | Tnfrsf17 |  |
|  |  |  |  |  |  |  |  |  |  |  |  | Ldha-ps2 |  |
|  |  |  |  |  |  |  |  |  |  |  |  | Gm30025 |  |
|  |  |  |  |  |  |  |  |  |  |  |  | Tesc |  |
|  |  |  |  |  |  |  |  |  |  |  |  | Gm26761 |  |
|  |  |  |  |  |  |  |  |  |  |  |  | C1qtnf9 |  |
|  |  |  |  |  |  |  |  |  |  |  |  | Gm12693 |  |
|  |  |  |  |  |  |  |  |  |  |  |  | Gm3375 |  |
|  |  |  |  |  |  |  |  |  |  |  |  | Tnnt1 |  |
|  |  |  |  |  |  |  |  |  |  |  |  | Chac1 |  |
|  |  |  |  |  |  |  |  |  |  |  |  | Ube2n-ps1 |  |
|  |  |  |  |  |  |  |  |  |  |  |  | Gm11221 |  |
|  |  |  |  |  |  |  |  |  |  |  |  | Gm5873 |  |
|  |  |  |  |  |  |  |  |  |  |  |  | Gm10762 |  |
|  |  |  |  |  |  |  |  |  |  |  |  | Gm9575 |  |
|  |  |  |  |  |  |  |  |  |  |  |  | Gm10167 |  |
|  |  |  |  |  |  |  |  |  |  |  |  | Cyp4f17 |  |
|  |  |  |  |  |  |  |  |  |  |  |  | Gm47469 |  |
|  |  |  |  |  |  |  |  |  |  |  |  | Oaz1-ps |  |
|  |  |  |  |  |  |  |  |  |  |  |  | D330023K18Rik |  |
|  |  |  |  |  |  |  |  |  |  |  |  | D830050J10Rik |  |
|  |  |  |  |  |  |  |  |  |  |  |  | Dkkl1 |  |
|  |  |  |  |  |  |  |  |  |  |  |  | E130102H24Rik |  |
|  |  |  |  |  |  |  |  |  |  |  |  | Opn3 |  |
|  |  |  |  |  |  |  |  |  |  |  |  | Hsbp1l1 |  |
|  |  |  |  |  |  |  |  |  |  |  |  | Gm6382 |  |
|  |  |  |  |  |  |  |  |  |  |  |  | Dnajc19-ps |  |
|  |  |  |  |  |  |  |  |  |  |  |  | 4930413G21Rik |  |
|  |  |  |  |  |  |  |  |  |  |  |  | Gm21378 |  |
|  |  |  |  |  |  |  |  |  |  |  |  | Gm20939 |  |
|  |  |  |  |  |  |  |  |  |  |  |  | Gm11613 |  |
|  |  |  |  |  |  |  |  |  |  |  |  | Rpp25 |  |
|  |  |  |  |  |  |  |  |  |  |  |  | Gm5846 |  |
|  |  |  |  |  |  |  |  |  |  |  |  | Gm3379 |  |
|  |  |  |  |  |  |  |  |  |  |  |  | Pgam1-ps2 |  |
|  |  |  |  |  |  |  |  |  |  |  |  | Nat8f1 |  |
|  |  |  |  |  |  |  |  |  |  |  |  | 2010204K13Rik |  |
|  |  |  |  |  |  |  |  |  |  |  |  | Gm14276 |  |
|  |  |  |  |  |  |  |  |  |  |  |  | Gm21092 |  |
|  |  |  |  |  |  |  |  |  |  |  |  | Gm7332 |  |
|  |  |  |  |  |  |  |  |  |  |  |  | Gm10179 |  |
|  |  |  |  |  |  |  |  |  |  |  |  | Mrto4-ps1 |  |
|  |  |  |  |  |  |  |  |  |  |  |  | Gm16001 |  |
|  |  |  |  |  |  |  |  |  |  |  |  | Gm13394 |  |
|  |  |  |  |  |  |  |  |  |  |  |  | Gm8210 |  |
|  |  |  |  |  |  |  |  |  |  |  |  | S100b |  |
|  |  |  |  |  |  |  |  |  |  |  |  | Gm12696 |  |
|  |  |  |  |  |  |  |  |  |  |  |  | 3830403N18Rik |  |
|  |  |  |  |  |  |  |  |  |  |  |  | Gm21887 |  |
|  |  |  |  |  |  |  |  |  |  |  |  | Gm44126 |  |
|  |  |  |  |  |  |  |  |  |  |  |  | Gm47813 |  |
|  |  |  |  |  |  |  |  |  |  |  |  | Ybx1-ps2 |  |
|  |  |  |  |  |  |  |  |  |  |  |  | Gm10355 |  |
|  |  |  |  |  |  |  |  |  |  |  |  | AV356131 |  |
|  |  |  |  |  |  |  |  |  |  |  |  | Itpa-ps1 |  |
|  |  |  |  |  |  |  |  |  |  |  |  | Rwdd2a |  |
|  |  |  |  |  |  |  |  |  |  |  |  | Gm10357 |  |
|  |  |  |  |  |  |  |  |  |  |  |  | Hmgb1-ps1 |  |
|  |  |  |  |  |  |  |  |  |  |  |  | Rpl36al |  |
|  |  |  |  |  |  |  |  |  |  |  |  | Rps25-ps1 |  |
|  |  |  |  |  |  |  |  |  |  |  |  | Gm31597 |  |
|  |  |  |  |  |  |  |  |  |  |  |  | Gm6304 |  |
|  |  |  |  |  |  |  |  |  |  |  |  | Tex12 |  |
|  |  |  |  |  |  |  |  |  |  |  |  | Rpl7a-ps3 |  |
|  |  |  |  |  |  |  |  |  |  |  |  | Gm6439 |  |
|  |  |  |  |  |  |  |  |  |  |  |  | R3hdml |  |
|  |  |  |  |  |  |  |  |  |  |  |  | Igkv14-130 |  |
|  |  |  |  |  |  |  |  |  |  |  |  | BC002163 |  |
|  |  |  |  |  |  |  |  |  |  |  |  | Gm6851 |  |
|  |  |  |  |  |  |  |  |  |  |  |  | Gm4535 |  |
|  |  |  |  |  |  |  |  |  |  |  |  | H1fx |  |
|  |  |  |  |  |  |  |  |  |  |  |  | Gm20467 |  |
|  |  |  |  |  |  |  |  |  |  |  |  | Cav3 |  |
|  |  |  |  |  |  |  |  |  |  |  |  | Sprr2h |  |
|  |  |  |  |  |  |  |  |  |  |  |  | Gm7390 |  |
|  |  |  |  |  |  |  |  |  |  |  |  | Gm15331 |  |
|  |  |  |  |  |  |  |  |  |  |  |  | Tagln3 |  |
|  |  |  |  |  |  |  |  |  |  |  |  | Gm44420 |  |
|  |  |  |  |  |  |  |  |  |  |  |  | Gm8971 |  |
|  |  |  |  |  |  |  |  |  |  |  |  | Gm12319 |  |
|  |  |  |  |  |  |  |  |  |  |  |  | Gm9234 |  |
|  |  |  |  |  |  |  |  |  |  |  |  | Gm3851 |  |
|  |  |  |  |  |  |  |  |  |  |  |  | Gm49377 |  |
|  |  |  |  |  |  |  |  |  |  |  |  | Gm44686 |  |
|  |  |  |  |  |  |  |  |  |  |  |  | Gm38560 |  |
|  |  |  |  |  |  |  |  |  |  |  |  | Gm44349 |  |
|  |  |  |  |  |  |  |  |  |  |  |  | Gm13655 |  |
|  |  |  |  |  |  |  |  |  |  |  |  | Fam162b |  |
|  |  |  |  |  |  |  |  |  |  |  |  | Gm40466 |  |
|  |  |  |  |  |  |  |  |  |  |  |  | Gm6087 |  |
|  |  |  |  |  |  |  |  |  |  |  |  | C330013E15Rik |  |
|  |  |  |  |  |  |  |  |  |  |  |  | Spink6 |  |
|  |  |  |  |  |  |  |  |  |  |  |  | Sprr2b |  |
|  |  |  |  |  |  |  |  |  |  |  |  | Gm42984 |  |
|  |  |  |  |  |  |  |  |  |  |  |  | Rpl36a-ps3 |  |
|  |  |  |  |  |  |  |  |  |  |  |  | Gm42937 |  |
|  |  |  |  |  |  |  |  |  |  |  |  | Gm5340 |  |
|  |  |  |  |  |  |  |  |  |  |  |  | Gm35638 |  |
|  |  |  |  |  |  |  |  |  |  |  |  | Rnf170-ps |  |
|  |  |  |  |  |  |  |  |  |  |  |  | Gnrh1 |  |
|  |  |  |  |  |  |  |  |  |  |  |  | Hist1h3c |  |
|  |  |  |  |  |  |  |  |  |  |  |  | Gm14794 |  |
|  |  |  |  |  |  |  |  |  |  |  |  | Gm14176 |  |
|  |  |  |  |  |  |  |  |  |  |  |  | Gm20544 |  |
|  |  |  |  |  |  |  |  |  |  |  |  | Gm44899 |  |
|  |  |  |  |  |  |  |  |  |  |  |  | Gm13421 |  |
|  |  |  |  |  |  |  |  |  |  |  |  | Gm48990 |  |
|  |  |  |  |  |  |  |  |  |  |  |  | Gm13237 |  |
|  |  |  |  |  |  |  |  |  |  |  |  | Gm6104 |  |
|  |  |  |  |  |  |  |  |  |  |  |  | Gm2991 |  |
|  |  |  |  |  |  |  |  |  |  |  |  | Gm38329 |  |
|  |  |  |  |  |  |  |  |  |  |  |  | Gm26910 |  |
|  |  |  |  |  |  |  |  |  |  |  |  | Gm20056 |  |
|  |  |  |  |  |  |  |  |  |  |  |  | Snord72 |  |
|  |  |  |  |  |  |  |  |  |  |  |  | Gm13461 |  |
|  |  |  |  |  |  |  |  |  |  |  |  | Mir6386 |  |
|  |  |  |  |  |  |  |  |  |  |  |  | Gm11772 |  |
|  |  |  |  |  |  |  |  |  |  |  |  | Ccl19-ps3 |  |
|  |  |  |  |  |  |  |  |  |  |  |  | AC165079.1 |  |
|  |  |  |  |  |  |  |  |  |  |  |  | Gm12178 |  |
|  |  |  |  |  |  |  |  |  |  |  |  | Gm7774 |  |
|  |  |  |  |  |  |  |  |  |  |  |  | Gm12397 |  |
|  |  |  |  |  |  |  |  |  |  |  |  | Bloc1s2-ps |  |
|  |  |  |  |  |  |  |  |  |  |  |  | Gm32105 |  |
|  |  |  |  |  |  |  |  |  |  |  |  | Gm12424 |  |
|  |  |  |  |  |  |  |  |  |  |  |  | Gm20442 |  |
|  |  |  |  |  |  |  |  |  |  |  |  | Gm5558 |  |
|  |  |  |  |  |  |  |  |  |  |  |  | E030024N20Rik |  |
|  |  |  |  |  |  |  |  |  |  |  |  | Gm10108 |  |
|  |  |  |  |  |  |  |  |  |  |  |  | Gm43587 |  |
|  |  |  |  |  |  |  |  |  |  |  |  | Mir145a |  |
|  |  |  |  |  |  |  |  |  |  |  |  | Gm7862 |  |
|  |  |  |  |  |  |  |  |  |  |  |  | Gm13822 |  |
|  |  |  |  |  |  |  |  |  |  |  |  | Gm25047 |  |
|  |  |  |  |  |  |  |  |  |  |  |  | mt-Tw |  |
|  |  |  |  |  |  |  |  |  |  |  |  | Gm24045 |  |
|  |  |  |  |  |  |  |  |  |  |  |  | 2310026L22Rik |  |
|  |  |  |  |  |  |  |  |  |  |  |  | Gm9442 |  |
|  |  |  |  |  |  |  |  |  |  |  |  | Gm43962 |  |
|  |  |  |  |  |  |  |  |  |  |  |  | Gm37486 |  |
|  |  |  |  |  |  |  |  |  |  |  |  | Gm48398 |  |
|  |  |  |  |  |  |  |  |  |  |  |  | CT025601.1 |  |
|  |  |  |  |  |  |  |  |  |  |  |  | Gm44955 |  |
|  |  |  |  |  |  |  |  |  |  |  |  | Smt3h2-ps4 |  |
|  |  |  |  |  |  |  |  |  |  |  |  | Gm43712 |  |
|  |  |  |  |  |  |  |  |  |  |  |  | Mir186 |  |
|  |  |  |  |  |  |  |  |  |  |  |  | Gm26397 |  |
|  |  |  |  |  |  |  |  |  |  |  |  | Gm38079 |  |
|  |  |  |  |  |  |  |  |  |  |  |  | Gm11966 |  |
|  |  |  |  |  |  |  |  |  |  |  |  | Gm22581 |  |
|  |  |  |  |  |  |  |  |  |  |  |  | Mir1945 |  |
|  |  |  |  |  |  |  |  |  |  |  |  | Mir3060 |  |
|  |  |  |  |  |  |  |  |  |  |  |  | Gm23084 |  |
|  |  |  |  |  |  |  |  |  |  |  |  | Snord43 |  |
|  |  |  |  |  |  |  |  |  |  |  |  | Gm23969 |  |
|  |  |  |  |  |  |  |  |  |  |  |  | Gm48042 |  |
|  |  |  |  |  |  |  |  |  |  |  |  | Iglj3 |  |
|  |  |  |  |  |  |  |  |  |  |  |  | Gm25897 |  |
|  |  |  |  |  |  |  |  |  |  |  |  | Gm22571 |  |
|  |  |  |  |  |  |  |  |  |  |  |  | Mir199a-1 |  |
|  |  |  |  |  |  |  |  |  |  |  |  | Mir1932 |  |
|  |  |  |  |  |  |  |  |  |  |  |  | Snord42b |  |
|  |  |  |  |  |  |  |  |  |  |  |  | Ighd3-2 |  |
